# β-Turn mimetic-based stabilizers of protein–protein interactions for the study of the non-canonical roles of leucyl-tRNA synthetase†
†Electronic supplementary information (ESI) available: Detailed procedures for synthesis, bioassay, and biophysical experiments, and full characterization data of all new compounds. See DOI: 10.1039/c5sc03493k


**DOI:** 10.1039/c5sc03493k

**Published:** 2015-12-15

**Authors:** Chanwoo Kim, Jinjoo Jung, Truong T Tung, Seung Bum Park

**Affiliations:** a Department of Chemistry , Seoul National University , Seoul 151-747 , Korea . Email: sbpark@snu.ac.kr ; Fax: +82 2 884 4025; b Department of Biophysics and Chemical Biology , Seoul National University , Seoul 151-747 , Korea

## Abstract

For the systematic perturbation of protein–protein interactions, we designed and synthesized tetra-substituted hexahydro-4*H*-pyrazino[2,1-*c*][1,2,4]triazine-4,7(6*H*)-diones as β-turn mimetics. We then devised a new synthetic route to obtain β-turn mimetic scaffolds *via* tandem *N*-acyliminium cyclization and constructed a 162-member library of tetra-substituted pyrazinotriazinediones with an average purity of 90% using a solid-phase parallel synthesis platform. Each library member was subjected to ELISA-based modulator screening for the LRS–RagD interaction, which plays a pivotal role in the nutrient-dependent mTORC1 signalling pathway. Western blot analysis of phosphorylated S6K1 as well as FRET-based imaging confirmed that **5c{3,9}** stabilizes the direct interaction between LRS and RagD and activates mTORC1 in live cells under leucine-deprived conditions. Thus, **5c{3,9}** can be used as a new research tool for studying the non-canonical role of LRS.

## Abstract 2

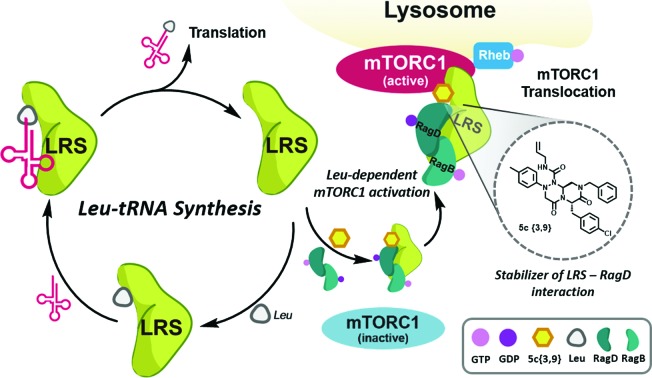
For the systematic perturbation of protein–protein interactions, we designed and synthesized tetra-substituted hexahydro-4*H*-pyrazino[2,1-*c*][1,2,4]triazine-4,7(6*H*)-diones as β-turn mimetics. **5c{3,9}** stabilizes the direct interaction between LRS and RagD and activates mTORC1 in living cells.

## Introduction

Protein–protein interactions (PPIs) play critical roles in a myriad of biological processes and many complex diseases, including cancer^1^ and metabolic and neurodegenerative diseases that are often caused by aberrant PPIs.^2^–^6^ Given their significance in biological systems, PPIs have been investigated to identify novel therapeutic targets for various diseases. Despite progress over the past 20 years, at most 40 PPIs have been targeted among the ∼650 000 pair-wise interactions in the human interactome, and a limited number of modulators have reached clinical trials.^4^–^6^ It is probably due to the fact that PPI interfaces do not typically bind to endogenous ligands that may serve as lead structures for drug discovery. PPI interfaces also present inherent physical challenges: the small-molecule binding region is likely noncontiguous to the interface between interacting proteins, and the PPI interface itself is relatively large and/or flat.^3^,^4^,^7^ In fact, few studies have reported successful and/or sufficiently potent PPI modulators because PPI modulators do not fully overlap with the criteria of drug-likeness based on FDA-approved orally available drugs.^5^,^6^,^8^ For the systematic perturbation of diverse PPIs, various synthetic strategies have been used to construct diverse molecular frameworks, such as polyheterocycles and macrocycles, with different characteristics compared to conventional inhibitors at the active site of druggable targets as substrate analogs.^9^–^14^

A recent structural analysis of PPI interfaces revealed that not all residues at the PPI interface were critical, but rather that small “hot spots” conferred most of the binding energy.^4^–^6^,^15^–^17^ A hot spot can be defined as a specific structural region that assembles at the PPI interface with conformational adaptivity.^4^,^18^ Hot spot areas are generally hydrophobic and can be comparable to the size of a small molecule, suggesting that PPIs can be modulated *via* the specific binding of small molecules at the hot spots of the protein partners.^4^,^7^ The structural elements for specific binding at hot spots include three major secondary structures such as α-helix, β-turn and β-strand, and small-molecule-based mimetics may mediate PPIs by their binding at the PPI interface.^17^,^19^ Therefore, these sites could be used for the design and synthesis of drug-like small molecules that mimic these recognition motifs for the systematic identification of novel modulators of protein–protein interactions.

Because of the conformational adaptivity of hot spots under physiological conditions, small-molecule-based mimetics of secondary structures may be useful in studies of the biological processes of PPIs with unknown structures or as potential candidates for first-in-class therapeutics. Although a few β-turn mimetics have been reported as PPI modulators, these motifs are frequently observed in the loop regions of protein domains and are thought to be important in PPIs.^19^,^20^ Therefore, β-turn mimetics should be constructed for use as PPI modulators and in studies of unexplored biological processes. It would be particularly valuable to construct polar polyheterocyclic core skeletons that can accommodate a series of hydrophobic substituents, which is critical for specific binding at hot spots, without sacrificing the solubility and membrane permeability of the resulting β-turn-mimetic-based small molecules with multiple hydrophobic substituents.^17^,^19^,^21^,^22^

**Fig. 1:**
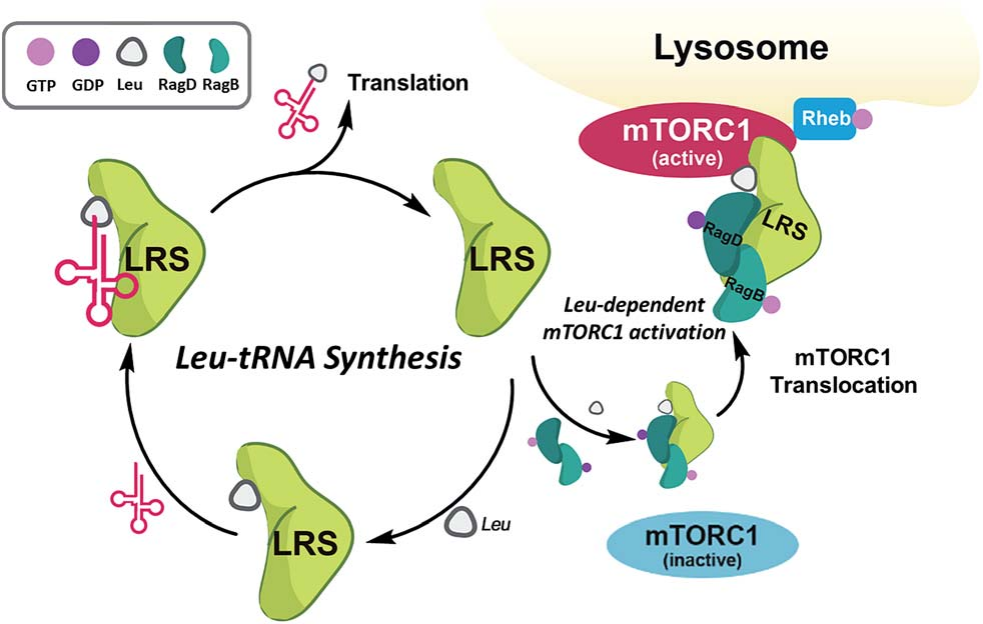
Roles of leucyl-tRNA synthetase. (i) Canonical role (left circle) of leucyl-tRNA synthetase; LRS conjugates leucine and cognate tRNA. (ii) Non-canonical role (right circle) of LRS–leucine-dependent mTORC1 activation; LRS binds to RagD and activates mTORC1 in a leucine-dependent manner.

Among the PPIs involved in various biological processes, we are interested in specific PPIs related to the mechanistic target of rapamycin complex 1 (mTORC1), which is a serine/threonine kinase that controls mRNA transcription and translation by integrating various environmental changes and signals such as growth factors, nutrients and energy status. Thus, mTORC1 modulates cell growth and proliferation and the dysregulation of mTORC1 is closely related to many diseases such as cancer and diabetes, making mTORC1 an attractive therapeutic target.^23^ A recent study demonstrated that Ras-related GTPases (Rag) mediate amino acid-based activation of mTORC1^24^ and that leucyl-tRNA synthetase (LRS) is thought to be a binding partner of Rag proteins, particularly RagD, in a leucine-dependent manner. Along with its canonical role in the conjugation of leucine to its cognate tRNA for leucyl-tRNA synthesis, LRS can also act as a leucine sensor, bind to RagD-GTP, and form a LRS–RagD complex, which translocates mTORC1 from the cytosol to the lysosome surface for subsequent activation of the mTORC1 signalling pathway (Fig. 1).^25^ The nutrient sensing mechanism of mTORC1, particularly for Leu, an essential biomarker for nutrient status in cellular systems,^26^ may be regulated by PPIs between LRS and RagD and directly mediate mTORC1 activation. Therefore, we hypothesized that small-molecule PPI modulators between LRS and RagD can be used as powerful research tools for studying the nutrient-dependent activation of mTORC1 and the subsequent biological outcomes. The protein structures of both human LRS and RagD are unknown and the binding mode of the LRS–RagD complex has not been determined. Thus, we attempted to identify PPI modulators of the LRS–RagD interaction through the systematic construction of polar molecular frameworks with limited conformational flexibility to mimic various secondary structures such as β-turns and γ-turns.

Herein, we report the design and synthesis of a β-turn mimetic hexahydro-4*H*-pyrazino[2,1-*c*][1,2,4]triazine-4,7(6*H*)-dione **1**. A small-molecule library of β-turn mimetics was constructed using a parallel synthetic strategy involving both solid- and solution-phase reactions. We also maximized the molecular diversity of our peptidomimetic library to occupy the potential chemical space for the specific modulation of protein–protein interactions. In order to determine whether the β-turn mimetics would perturb protein–protein interactions, we conducted an ELISA-based biological evaluation of our β-turn mimetic library against the specific interaction between LRS and RagD and identified a novel PPI stabilizer targeting the LRS–RagD interaction.

## Results and discussion

### Design of a tetra-substituted pyrazinotriazinedione scaffold as a β-turn mimetic

**Fig. 2:**
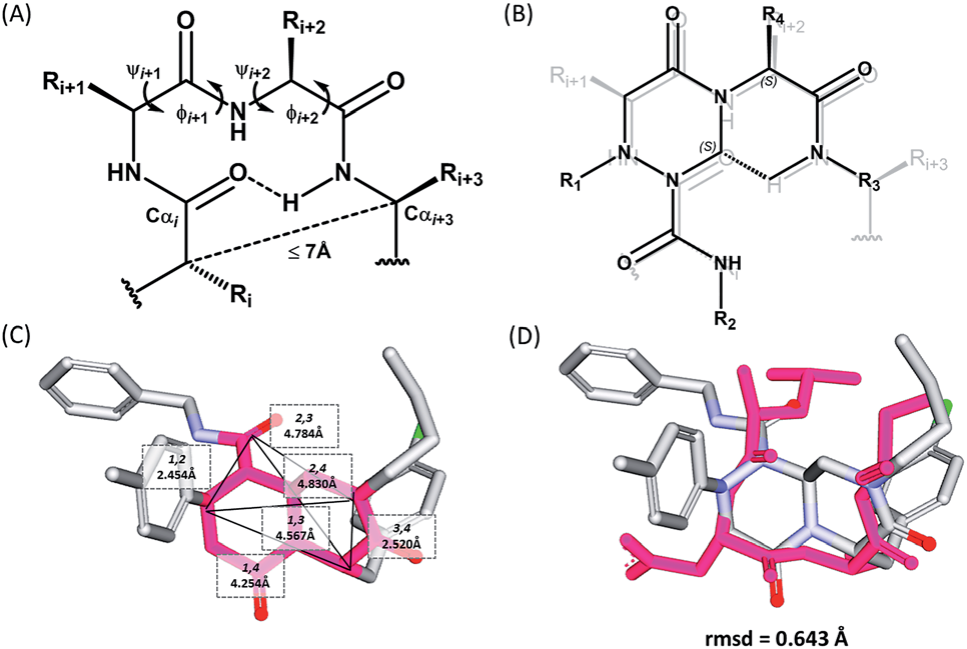
(A) Schematic diagram of a β-turn structure. (B) Overlay of a potential mode of β-turn mimicry with a tetra-substituted hexahydro-4*H*-pyrazino[2,1-*c*][1,2,4]triazine-4,7(6*H*)-dione. (C) Distance between substituents in the energy-minimized conformer of its tetra-substituted representative analog **7** containing 4-methylphenyl, benzyl, butyl, and 4-chlorophenyl at the R_1_–R_4_ positions, respectively. (D) Alignment of an energy-minimized structure of representative compound **7** with the peptide backbone structure of phospholipase A2 β-turn motif (PDB: ; 4BP2).

The β-turn is one of the three major secondary structural motifs found in proteins and peptides, particularly in the reverse direction of polypeptide strands. The peptidomimetics of β-turns have been investigated to identify small-molecule modulators that disrupt β-turn-mediated recognition events.^19^,^27^–^31^ As illustrated in Fig. 2A, the β-turn consists of four amino acid residues, designated as *i*, *i* + 1, *i* + 2, and *i* + 3. One of the important features of the β-turn structure is that the distance between the Cα_*i*_ and Cα_*i*+3_ atoms is less than 7 Å. Depending on the dihedral angles *ψ* and *φ* of the *i* + 1 and *i* + 2 residues, respectively, nine β-turn types defined by Hutchinson and Thornton are widely used: types I, I′, II, II′, VIa1, VIa2, VIb, VIII, and IV.^19^,^32^ To successfully construct β-turn structures, it is essential to mimic the back bone alignment and functionality of the side chains.^19^ Dihedral angles and the mean distances between the α-carbon centers of β-turn mimetics are considered to be geometric indicators for evaluating the mimetic capability in 3-dimensional space because of their crucial roles in β-turn structures.^5^,^6^,^33^ Using this approach, Whitby *et al.* reported a strategy for designing *trans*-pyrrolidine-3,4-dicarboxamide as a β-turn mimetic structure as well as investigating its biological activities.^29^ Since protein–protein interactions primarily occur at the hydrophobic “hot spot” region of the PPI interfaces, we also considered the general characteristics of substituents appended to the β-turn skeleton to mimic the hydrophobic side chains of the residues at hot spots.

For β-turn mimicry, we designed and synthesized a fused bicyclic scaffold, tetra-substituted hexahydro-4*H*-pyrazino[2,1-*c*][1,2,4]triazine-4,7(6*H*)-dione (**1**), as a β-turn mimetic to produce the desired conformation that could maintain its β-turn structural features with limited conformational flexibility under physiological conditions. The rigid bicyclic scaffold **1** allowed several slightly different conformers to cover the geometric β-turn structures and the resulting conformational restriction may have induced a higher binding affinity toward biopolymers because of its prepaid entropic penalty.^27^ As shown in Fig. 2B, each α-carbon of the β-turn motif matched well with each atom of our scaffold **1**. When we calculated the distances between each atom of the energy-minimized conformer of its representative analog **7**, the distance and geometry of the substituents were well-matched with the designated α-carbon of a β-turn motif, confirming that our β-turn mimetic scaffold **1** fits well into the geometric analysis data of β-turns observed in 10 245 reported structures in the protein data bank (Fig. 2C).^29^ As final confirmation, the energy-minimized 3-dimensional structure of **7** was overlapped with the X-ray crystal structure of phospholipase A2 containing the β-turn motif in its biologically active conformation.^34^,^35^ As shown in Fig. 2D, **7** overlaps with the β-turn backbone structure of phospholipase A2 (in pink) showing excellent conformational similarity. The root-mean-square deviation (rmsd) value of compound **7** compared with each α-carbon of phospholipase A2 was 0.643 Å for five atom positions (see the ESI†). We also confirmed the moderate rmsd values (0.544–0.916 Å) of compound **7** toward five different types of β-turn structures (I, II, II′, III and VIII). Based on these results, we were confident that our pyrazinotriazinedione scaffold **1** mimicked the natural β-turn structures with limited conformational flexibility. In addition, our β-turn mimetic **1** showed a high *F*_sp^3^_ value (0.67) with two stereogenic centers, which may allow selective binding toward certain target proteins to perturb PPIs.

### High-throughput library synthesis of tetra-substituted pyrazinotriazinediones as β-turn mimetics

**Fig. 3:**
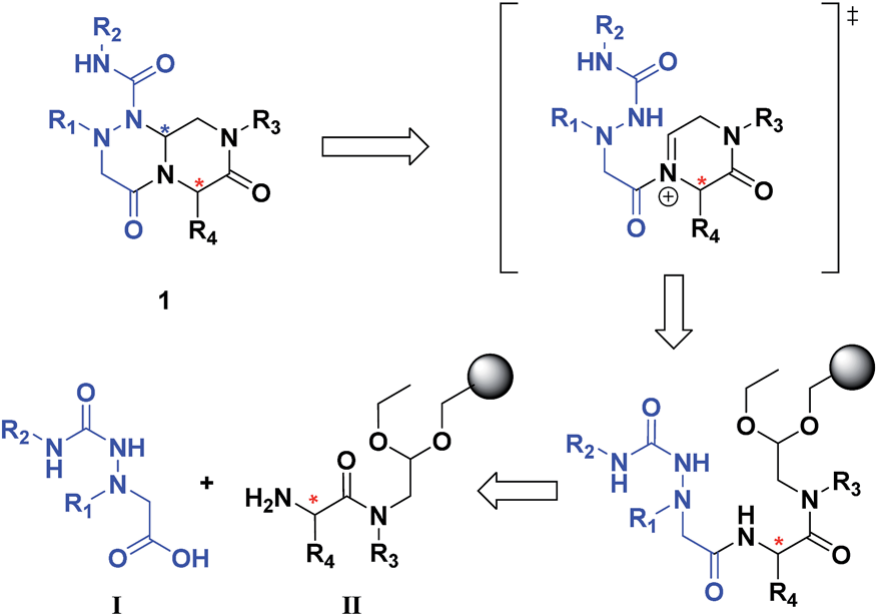
Retrosynthetic analysis of tetra-substituted hexahydro-4*H*-pyrazino[2,1-*c*][1,2,4]triazine-4,7(6*H*)-dione (**1**).

The retrosynthetic analysis of tetra-substituted hexahydro-4*H*-pyrazino[2,1-*c*][1,2,4]triazine-4,7(6*H*)-dione **1** is shown in Fig. 3. To construct the β-turn mimetic library, we designed two amide coupling partners; the carboxylic acid partner (**I**) containing the R_1_ and R_2_ groups and the amine partner (**II**) on a solid support containing the R_3_ and R_4_ groups. To ensure the efficiency of the high-throughput parallel synthesis, we synthesized the carboxylic acid partner (**I**) in the solution phase, and the subsequent amide coupling with the amine partner (**II**) allowed for the formation of the linear precursor on a solid support. The final acid-catalyzed cleavage step from the solid support was designed for the *in situ* formation of *N*-acyliminium intermediates from aldehydes and an amide nitrogen, followed by their tandem stereoselective cyclization to obtain the β-turn mimetic scaffold **1** with a new stereogenic center. This pyrazinotriazinedione **1** contained four modifiable sites to maximize the molecular diversity, which could mimic the functionalities of side-chain moieties in natural amino acids for the specific recognition events in various PPIs.

**Scheme 1:**
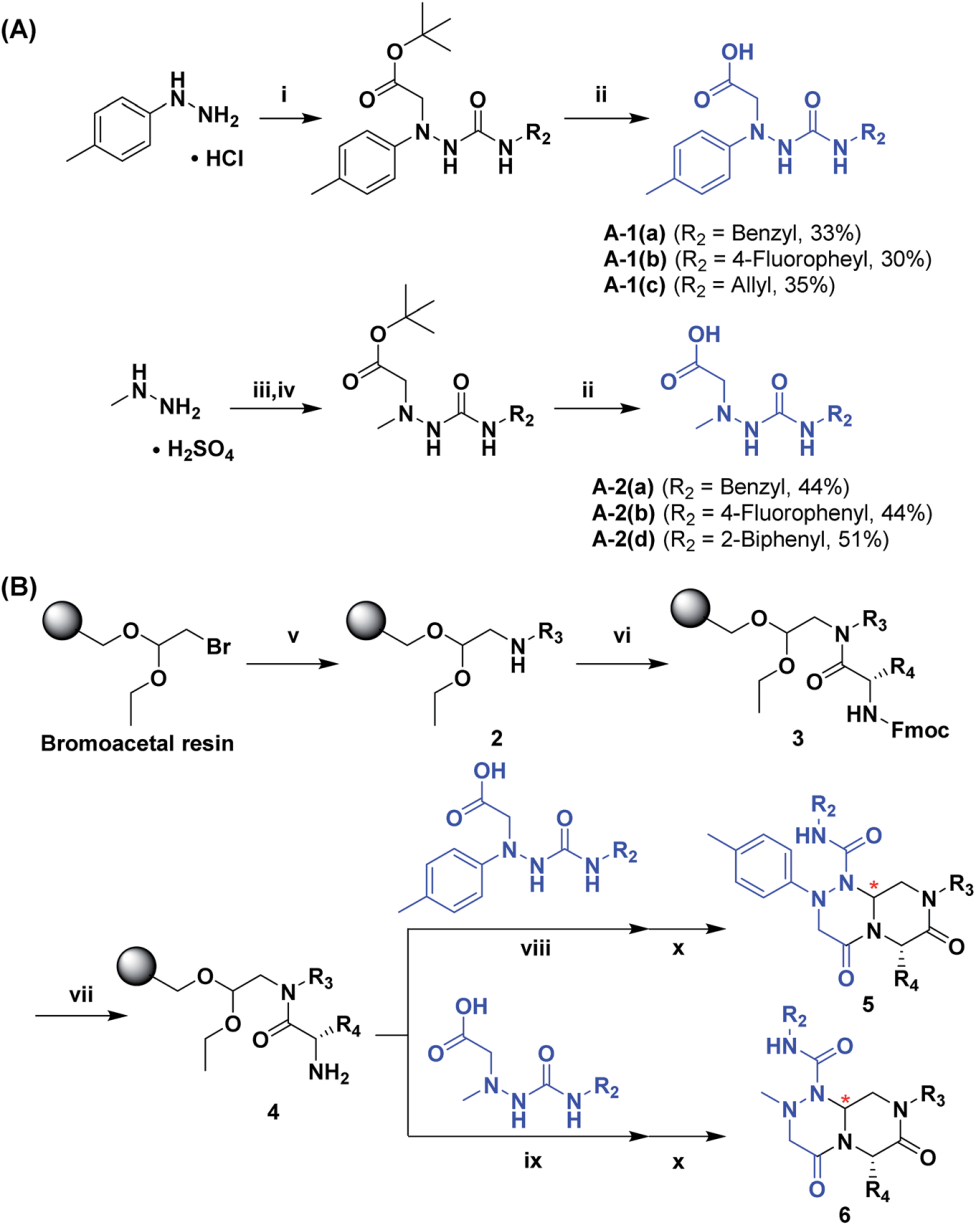
General synthesis scheme for acid partners (A) and tetra-substituted hexahydro-4*H*-pyrazino[2,1-*c*][1,2,4]triazine-4,7(6*H*)-dione (B). Reagents and conditions: (i) R_2_-isocyanate, TEA, THF, 0 °C to r.t., 5 h, then *t*-butyl bromoacetate, KHCO_3_, DMF, 80 °C, 12 h; (ii) 4 N HCl, 1,4-dioxane, r.t., overnight; (iii) Boc_2_O, NaHCO_3_, THF/H_2_O (1 : 1 v/v), r.t., overnight, then R_2_-isocyanate, TEA, THF, 0 °C to r.t., 5 h; (iv) 4 N HCl, 1,4-dioxane, r.t., then *t*-butyl bromoacetate, K_2_CO_3_, toluene/DMF (8 : 1 v/v), reflux, 12 h; (v) R_3_-NH_2_, DMSO, 60 °C, 12 h; (vi) Fmoc-amino acid, HCTU [*O*-(6-chlorobenzotriazol-1-yl)-*N*,*N*,*N*′,*N*′-tetra-methyluronium hexafluorophosphate], DIPEA, DMF, r.t., 4 h; (vii) 20% piperidine, DMF, r.t., 10 min; (viii) acid coupling partner (**A-1**), HOBt [hydroxybenzotriazole], DIC [*N*,*N*′-diisopropylcarbodiimide], DMF, r.t., 3 h; (ix) acid coupling partner (**A-2**), HOBt, DIC, DMF, r.t., 3 h; (x) formic acid, r.t., 18 h.

To generate carboxylic acid partners (**A-1** & **A-2**), we used two different hydrazines (methyl and tolyl) for diversification at the R_1_ position. Since a large quantity of acid partners is required for library construction, the synthesis of the acid partners was carried out in the solution phase (Scheme 1A). The synthetic route that we used for methylhydrazine differed from that for tolylhydrazine because methylhydrazine requires Boc protection (iii) of its internal nitrogen to differentiate the nucleophilicity of its two nitrogens. In the case of tolylhydrazine, we treated it with three different isocyanates (benzyl, 4-fluorophenyl, and allyl) *via* nucleophilic addition at 0 °C to modify the external nitrogen (i). The remaining internal nitrogen was further decorated with *t*-butyl bromoacetate *via* S_N_2 reaction in the presence of KHCO_3_ at an elevated temperature. Due to the limited nucleophilicity of the internal nitrogen in tolylhydrazine, its modification step with *t*-butyl bromoacetate suffered with the formation of a diazene moiety as a major by-product, which lowered the overall yields regardless of the types of isocyanates used. For methylhydrazine, Boc-protected methylhydrazine was modified with three different isocyanates (benzyl, 4-fluorophenyl and 2-biphenyl) (iii), followed by Boc deprotection using 4 N HCl in 1,4-dioxane (iv). After the S_N_2-type modification of the internal nitrogen with *t*-butyl bromoacetate, six different carboxylic acid partners (**A-1** & **A-2**) were obtained through acidic hydrolysis on a gram scale in 3–4 steps with moderate overall yields (30–51%).

As shown in Scheme 1B, library synthesis was initiated by preparing solid-supported amine partners **4** with various R_3_ and R_4_ substituents. First, bromoacetal resin was loaded with three different amines containing methylfuranyl, *n*-butyl and benzyl moieties at the R_3_ position *via* S_N_2 reaction (v). Next, solid-supported secondary amines **2** were coupled to various Fmoc-protected natural or unnatural amino acids [Phe, Val, Met, Ile, Tyr, Leu, Arg, Cys(bzl) and Phe(4-Cl)] under the HCTU-based amide coupling conditions (vi). After the deprotection of Fmoc amines **3** on a solid support using 20% piperidine (vii), the resulting amine partners **4** were coupled with carboxylic acid partners (**A-1** or **A-2**) under HOBt and DIC-mediated amide coupling conditions (viii or ix), respectively. After completion of the amide coupling monitored using ninhydrin testing, the final acidolysis step (x) involving neat formic acid allowed the formation of bicyclic β-turn mimetic scaffold **1** in excellent purities *via* the *N*-acyliminium intermediate shown in Fig. 3.

As shown in Table 1, eight representative compounds (**7–14**) were obtained without further purification with good purities and were fully characterized using ^1^H, ^13^C, and gCOSY NMR spectroscopy along with LC/MS and chiral HPLC analysis (see the ESI†). Because PPI interfaces generally contain some hydrophobic regions, various aromatic and hydrophobic substituents were adopted at the R_1_–R_4_ position of this β-turn mimetic scaffold **1** to target these hot spots. Each substituent at four different diversity points was selected to mimic the side-chains in the amino acids of the β-turn structures from natural to unnatural amino acids as well as to maximize the molecular diversity and drug-likeness of the resulting β-turn mimetic library *via* principal component analysis (see the ESI†). It is worth mentioning that our pyrazinotriazinedione skeleton itself is polar (clog *P* = –1.26) enough to accommodate a series of hydrophobic substituents and maintain the drug-likeness of the resulting β-turn mimetics (clog *P* range from 3.45 to 7.47).

**Table 1:** Purity and mass conformation of representative compounds

	R^1^	R^2^	R^3^	R^4^	Purity^*a*^ (%)	MS[M + H]^+^	
						Calcd	Found
**7**	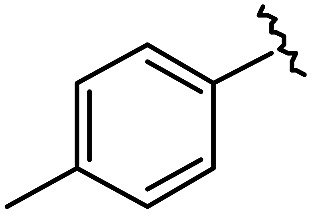	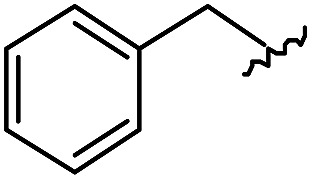	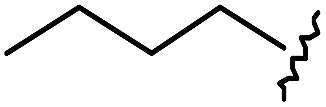	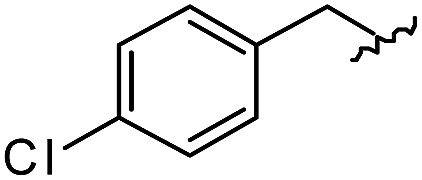	91	574.25	574.22
**8**	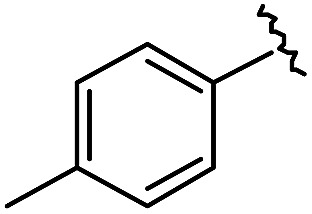	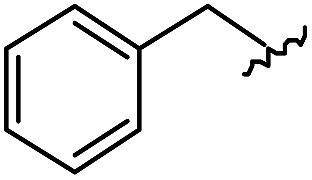	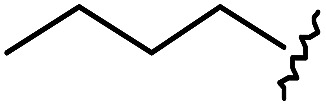	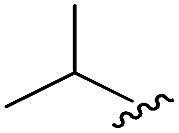	96	492.29	492.04
**9**	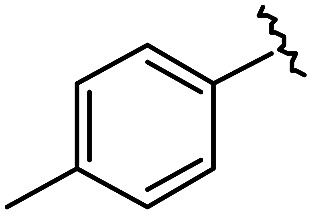	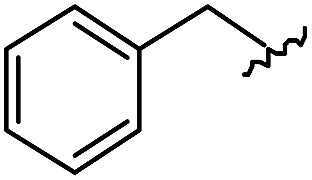	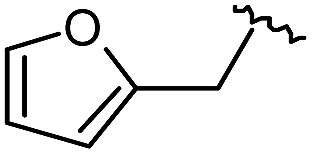	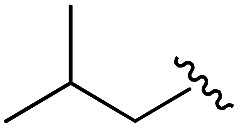	99	530.27	530.03
**10**	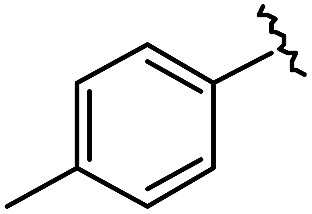	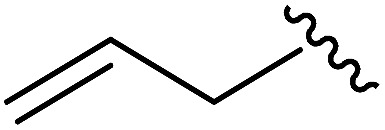	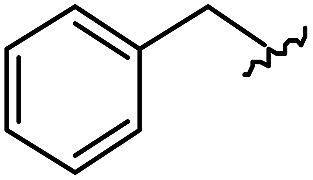	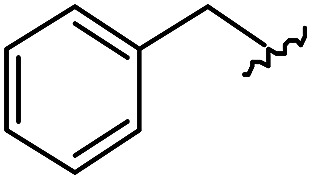	93	524.26	524.15
**11**	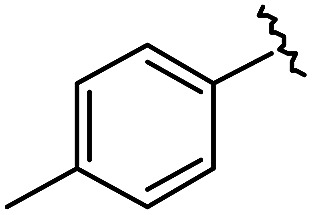	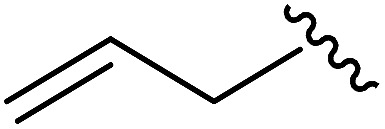	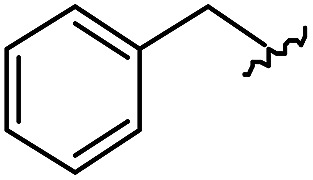	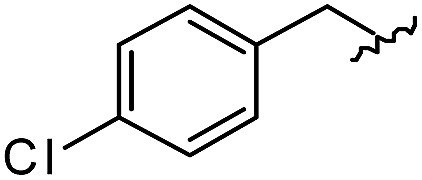	98	558.22	558.20
**12**	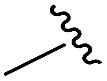	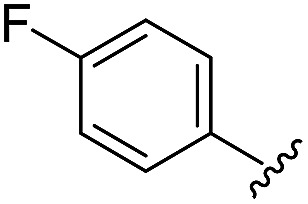	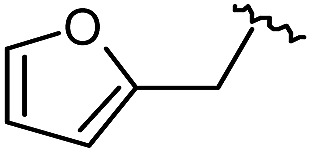	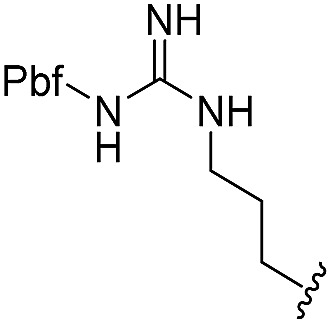	90	753.31	753.67
**13**	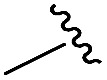	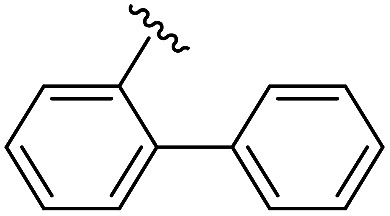	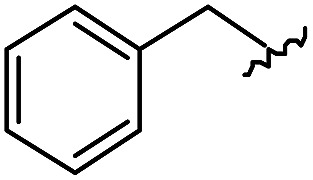	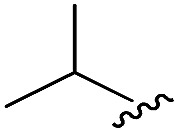	90	512.26	512.32
**14**	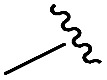	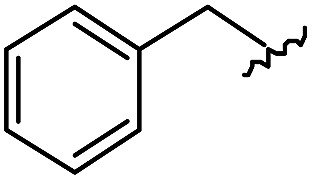	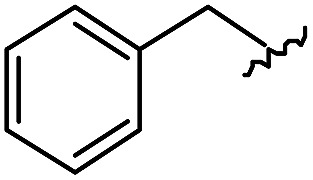	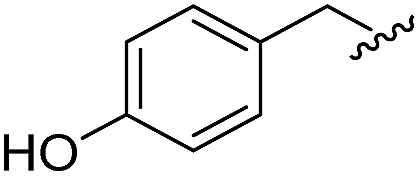	95	514.24	514.06

^*a*^Purities in percentage were obtained using PDA (photodiode array)-based LC/MS analysis of crude final compounds after a short filter column. Pbf = 2,2,4,6,7-pentamethyldihydrobenzofuran.

**Fig. 4:**
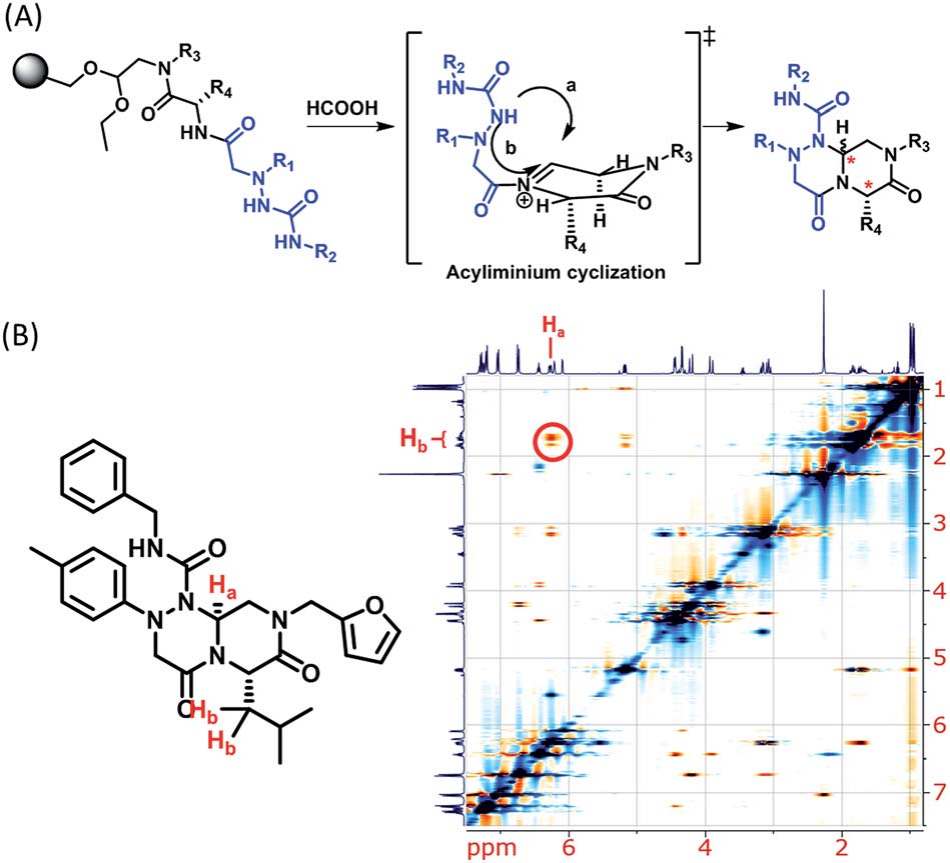
(A) Two possible trajectories of the nitrogen nucleophile (pathways a & b) *via* tandem *N*-acyliminium cyclization. (B) Representative compound **9** and its 2D-NMR NOE data.

Using this synthetic route, a new chiral center was generated in bicyclic β-turn mimetic scaffold **1** in a stereoselective manner. In fact, a new stereogenic center was generated *via* two different trajectories of the nitrogen nucleophile (pathways a and b) during the tandem *N*-acyliminium cyclization step under neat formic acid (see Fig. 4A). However, we only observed a single diastereomer in the reaction mixture, which was confirmed using crude LC/MS and crude ^1^H NMR spectroscopy. After purification of representative compound **9**, we performed a 2D nuclear Overhauser effect (NOE) NMR experiment to confirm the absolute configuration of the β-turn mimetic pyrazino-triazinedione scaffold. As shown in Fig. 4B (red color), we clearly observed NOE interactions between H_a_ at the new stereogenic center and H_b_ at the R_4_ position, and concluded that the nitrogen nucleophile attacked the top face of the *N*-acyliminium intermediate (pathway a) to avoid the steric repulsion of bulky R_4_ residues during tandem cyclization.^36^ Using a solid-phase parallel synthetic strategy, we efficiently constructed a 162-member β-turn mimetic library and the purities of all library members were measured using LC/MS equipped with a PDA detector after short silica-gel filtration (Table 2). The average purity was found to be 90%.

**Table 2:** Purity table of all final compounds^*a*^

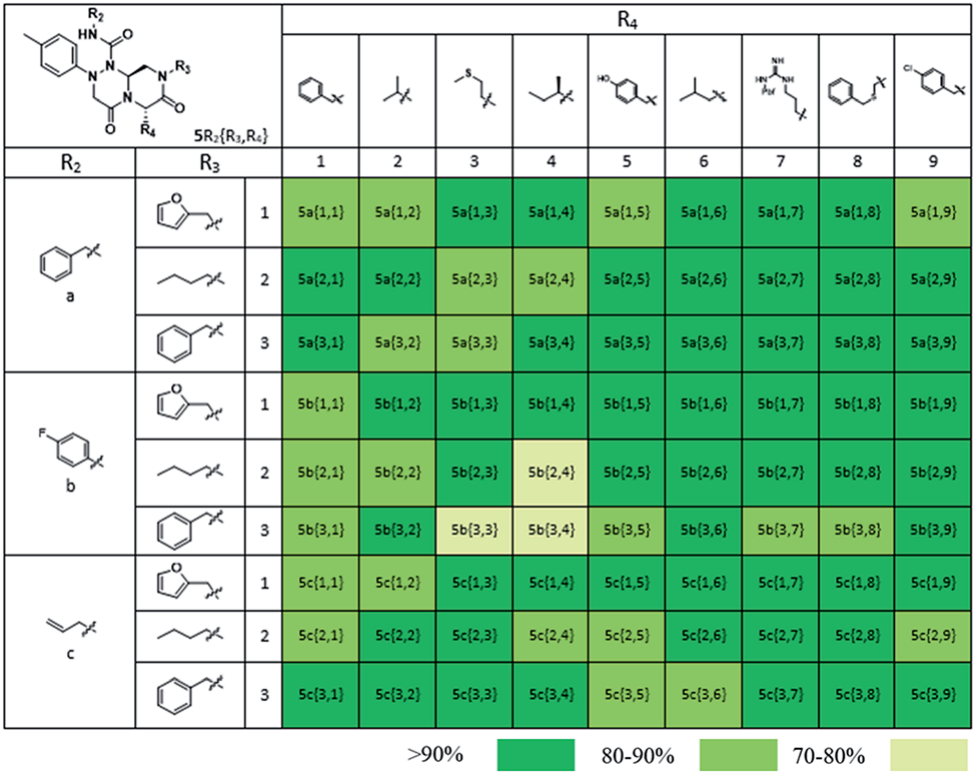
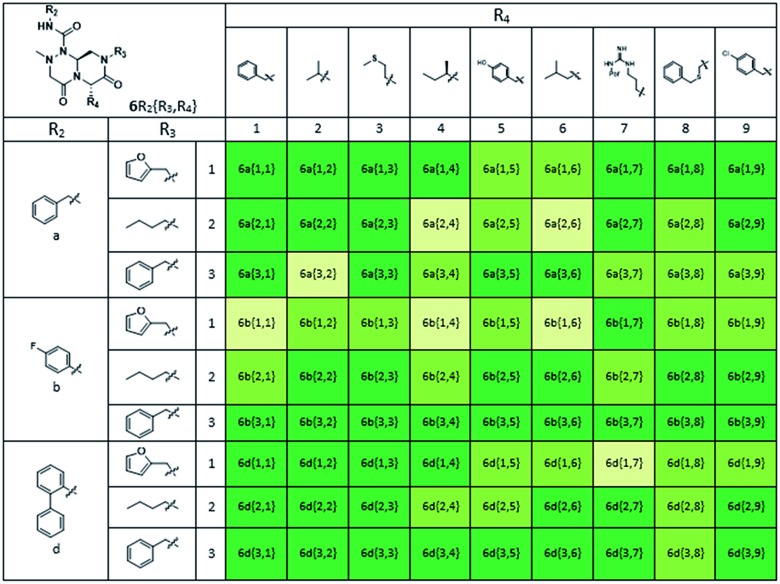

^*a*^Purities (%) were obtained using PDA-based LC-MS analysis of crude final compounds after a short filter column (average purities: 90%).

### Screening the β-turn mimetic library to identify LRS–RagD interaction modulators

To identify small-molecule modulators of the LRS–RagD interaction, we conducted ELISA-based screening with purified LRS coated on 96-well plates and each well was treated with each β-turn mimetic library member and GST (Glutathione S-transferase)-tagged RagD simultaneously for 3 h. Due to its *in vitro* instability, GST-RagD was roughly purified using a glutathione column and used immediately without freezing. The specific interaction between LRS and RagD can capture RagD-GST at the surface of each well, which was detected using anti-GST antibody. The level of small-molecule-based modulation toward the LRS–RagD interaction was quantified based on the quantity of bound RagD-GST, which was measured by determining the enzyme activity conjugated to the secondary antibody.

**Fig. 5:**
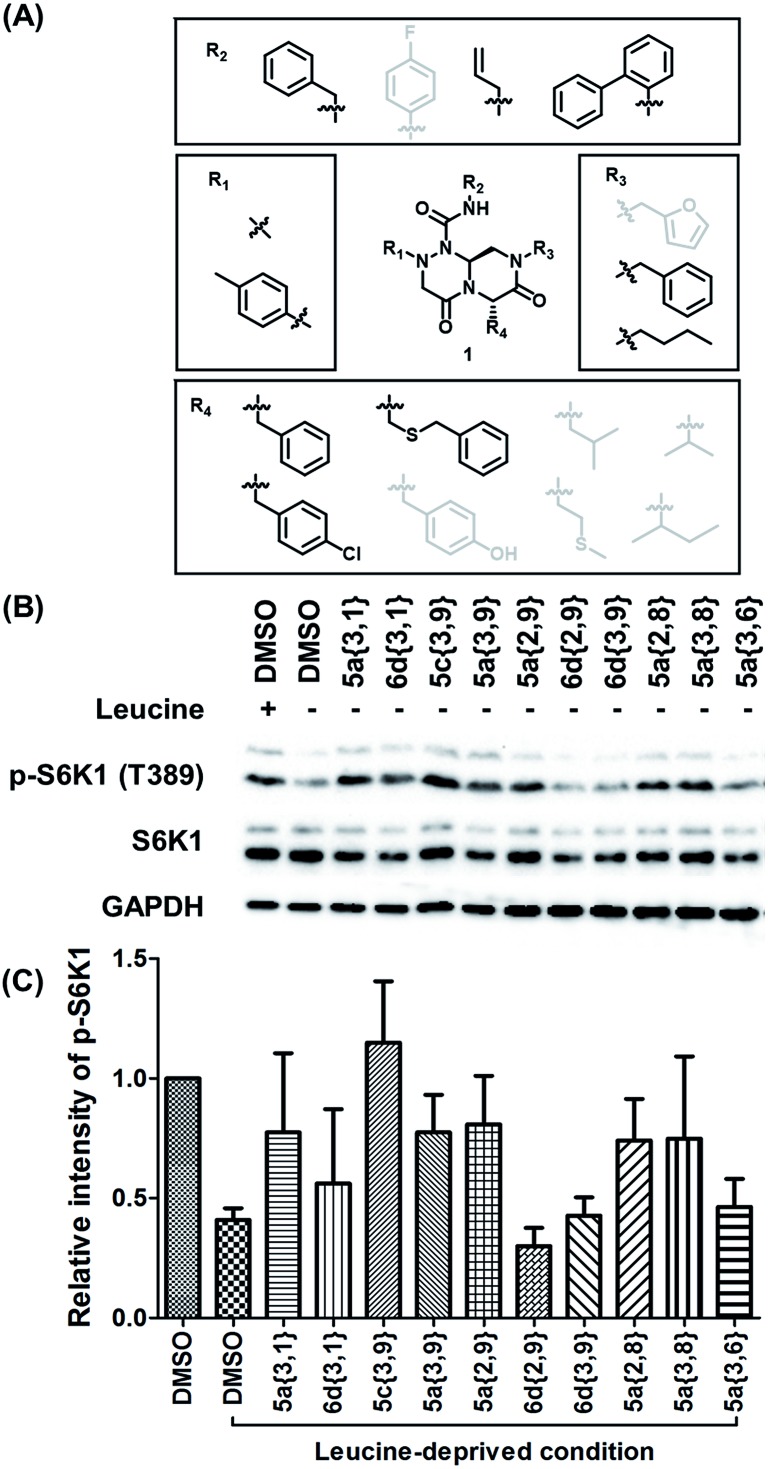
(A) Structure–activity relationship study of PPI stabilizers between LRS–RagD among our β-turn mimetic compounds. Compounds with hydrophobic and aromatic substituents (black) showed better stabilizing activity than others (grey). (B) HEK293T cells were starved of leucine for 1 h and treated with 20 μM compounds for 3 h under leucine-deprived conditions. Phospho-S6K1 (T389), S6K1, and GAPDH were determined using western blot. HEK293T cells treated with 0.6 mM leucine were used as a control. (C) Quantification of western blotting against a DMSO control.

Using ELISA-based screening, we tested all 162 compounds in the β-turn mimetic library to determine whether they could affect the interaction between LRS and RagD. We identified a series of β-turn mimetic compounds that stabilized the LRS–RagD interaction in a dose-dependent manner. It is difficult to identify small-molecule modulators of specific PPIs, but it is even more difficult to identify PPI stabilizers from a small-molecule library.^37^ Thus, our results confirm the quality and novelty of our β-turn mimetic library. Through our structure–activity relationship analysis, we clearly demonstrated that our pyrazinotriazinedione scaffold **1** with hydrophobic and aromatic substituents stabilized the LRS–RagD interaction more effectively (Fig. 5A), which is in accordance with our library design rationale, in which hydrophobic substituents on a single β-turn mimetic scaffold enhanced the binding affinity toward the hydrophobic region of hot spots at the PPI interfaces.

### Hit compound selection of LRS–RagD stabilizer and its biophysical evaluation

After selecting a series of hit compounds from our ELISA-based screening, we performed western blot analysis of phosphorylated S6K1, one of the mTORC1 substrates, to determine whether these compounds could affect the activity of mTORC1 by stabilizing the LRS–RagD interaction. As shown in Fig. 5B, the presence of leucine can initiate the LRS and RagD interaction, which is essential for the activation of mTORC1 and phosphorylation of the subsequent S6K1. Under leucine-deprived conditions, phosphorylated S6K1 (T389) decreased because of the inactivation of mTORC1. However, the phosphorylation level of S6K1 was successfully restored upon treatment with our β-turn mimetic compounds, as the LRS–RagD PPI conferred stability even under leucine-deprived conditions.

**Fig. 6:**
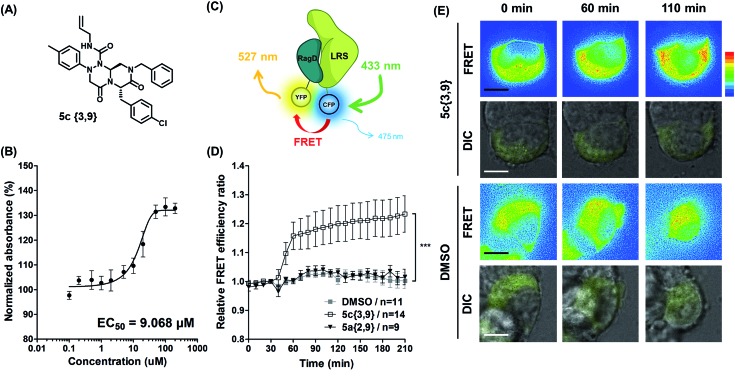
(A) Structure of the hit compound, **5c{3,9}**. (B) ELISA-based evaluation of LRS and RagD stabilization by **5c{3,9}** in a dose-dependent manner. (C) Schematic representation of FRET between LRS-CFP and RagD-YFP. When LRS directly interacts with RagD, LRS-fused CFP is physically close to RagD-fused YFP, generating a FRET signal. Fluorescence of 475 nm from CFP with excitation at 433 nm transfers to YFP and emits fluorescence at 527 nm. (D) Relative FRET efficiency ratio between LRS-CFP and RagD-YFP in HEK293T cells co-transfected with LRS-CFP and RagD-YFP. Images were taken at 10 min intervals over 3.5 h in a single cell. Cells were randomly selected from 3 independent experiments (*P* = 0.002; Mann–Whitney Test). **5c{3,9}** (clear square) or **5a{2,9}** (reverse black triangle) was treated at a 40 μM concentration after an initial 30 min of live cell imaging. Images were captured with CFP/CFP, CFP/YFP, and YFP/YFP filters (excitation/emission). Captured FRET images were analyzed using Softworks (imaging software) to exclude false-positive signal such as CFP crosstalk and YFP crosstalk. (E) Representative calculated FRET within cells. The color scale indicates the range of FRET intensity, from low (blue) to high (red). Scale bar, 20 μm.

Among the initial hit compounds, we identified the most potent compound based on the relative intensity of phosphorylated S6K1 in the Leu-deprived media compared to that in normal media (Fig. 5C) and selected **5c{3,9}** for further biophysical analysis (Fig. 6A and B). Using ELISA and western blot analysis, we confirmed that **5c{3,9}** stabilized the *in vitro* interaction between LRS and RagD and regulated the activity of mTORC1, leading to the restoration of phosphorylated S6K1 levels under leucine-deprived conditions. But, this observation does not necessarily confirm the stabilization of the direct LRS–RagD interaction. To confirm this, we established a novel biophysical assay using fluorescence resonance energy transfer (FRET) to examine the interaction between CFP (Cyan Fluorescent Protein)-fused LRS (LRS-CFP) and YFP (Yellow Fluorescent Protein)-fused RagD (RagD-YFP) in live cells: the extent of the LRS and RagD direct interaction was measured as the intensity of the FRET signal between CFP and YFP (Fig. 6C). To precisely quantify the FRET efficiency, we selected some single cells to track over time and conducted live cell imaging with CFP/CFP, CFP/YFP, and YFP/YFP filter sets (Fig. S3†). Finally, the captured images were analyzed to exclude false-positive signals such as CFP and YFP crosstalk. As shown in Fig. 6D and E, we observed that the FRET efficiency between CFP and YFP was increased over time after treatment with **5c{3,9}**, confirming the **5c{3,9}**-mediated stabilization of the LRS and RagD interaction under live cell conditions. For the less potent compound **5a{2,9}**, we observed marginal changes in the FRET efficiency that were similar to those in the DMSO control. This FRET-based biophysical experiment supported that our β-turn mimetic **5c{3,9}** stabilized the direct LRS–RagD interaction in the cellular system and modulated the activity of mTORC1.

### Small-molecule stabilizer of LRS–RagD interaction as a tool compound

Our β-turn mimetic bicyclic compound **5c{3,9}** selectively activated mTORC1, a key protein complex of cell growth and proliferation, with a novel mode of action—direct stabilization of the LRS–RagD interaction. **5c{3,9}** maintained the cells under normal conditions even in the leucine-deprived media. In fact, nutrient sensing, particularly amino acid sensing, is extremely important for cell survival, but there are no effective methods for dissecting the amino acid-mediated activation of mTORC1. Leucine is an essential amino acid in cells and plays a vital role in anabolic pathways such as skin wound healing and restoring muscle loss.^38^ Therefore, **5c{3,9}** could be an excellent research tool for activating the mTORC1 signalling pathway and used to reveal the non-canonical role of LRS in leucine-deficient conditions. Furthermore, **5c{3,9}** can be used as a novel lead compound for the selective activation of mTORC1 *via* LRS–RagD stabilization without targeting mTORC1 itself.

## Conclusions

For the systematic perturbation of PPIs, we designed and synthesized tetra-substituted hexahydro-4*H*-pyrazino[2,1-*c*][1,2,4]triazine-4,7(6*H*)-diones to mimic the β-turn structure, a key secondary structural motif. Distance calculations of energy-minimized conformers and the alignment of model compound **7** with side chains of a biologically active β-turn motif confirmed that our tetra-substituted β-turn mimetic bicycle **1** was conformationally similar to the natural β-turn structure. We then developed a robust synthetic pathway to obtain the β-turn mimetic scaffolds *via* tandem *N*-acyliminium cyclization and constructed a 162-member library of tetra-substituted pyrazinotriazinediones as β-turn mimetics with an average purity of 90% using a solid-phase parallel synthesis platform. In this synthetic route, a new chiral center was generated in a diastereoselective manner, which was confirmed using 2D NOE. After library construction, we examined whether these β-turn mimetic compounds could modulate PPIs and identified a series of compounds that could effectively stabilize the LRS–RagD interaction using ELISA-based screening. The cellular interaction between LRS and RagD directly mediated the leucine-dependent activation of mTORC1 and resulting biological outcomes. The western blot analysis of phosphorylated S6K1 and FRET imaging in live cell revealed that **5c{3,9}** stabilized the direct interaction between LRS and RagD and activated the leucine-dependent mTORC1 signalling pathway in living cells. With this mechanism of action, **5c{3,9}** can serve as an excellent research tool for studying the non-canonical role of LRS under leucine-deficient conditions and as a lead structure for examining disease models that require the selective activation of anabolic processes for wound healing or the restoration of muscle loss.

## Supplementary Material

Supplementary information
