# Supplementary material for: β-Turn mimetic-based stabilizers of protein–protein interactions for the study of the non-canonical roles of leucyl-tRNA synthetase
Source: Chem Sci. 2015 Dec 15;7(4):2753–61. doi: 10.1039/c5sc03493k (PMC5477029; doi:10.1039/c5sc03493k)
Supplement: Supplementary file 1 [file SC-007-C5SC03493K-s001.pdf]

Electronic Supplementary Material (ESI) for Chemical Science.
This journal is © The Royal Society of Chemistry 2015

< Supporting Information >

β-Turn Mimetic-based Stabilizers of Protein-Protein Interactions for Study of the Non-canonical Roles of Leucyl-tRNA Synthetase

Chanwoo Kim[a,§], Jinjoo Jung[b,§], and Seung Bum Park*[,a,b]

[a]*Department of Chemistry and* [b]*Department of Biophysics and Chemical Biology, Seoul National University, Seoul 151–747, Korea*

sbpark@snu.ac.kr

[§]*These authors contribute equally in this study*

S. No.	Content	Page No.
I	General Information	S2
II	Tetra-substituted pyrazinotriazinedione as β-turn mimetic	S5
III	Synthesis and characterization of acid partners	S6
IV	Characterization for representative compounds	S10
V	Principal component analysis (PCA) of pyrazinotriazinedione library	S27
VI	Copies of ^{1}H and ^{13}C NMR spectra	S29
VII	PDA-based LC/MS analysis data for library compounds	S43
VIII	FRET imaging	S93
IX	Experimental procedure for biological assay	S94

I. General Information

1. General information of library construction

NMR spectra were obtained on an Agilent 400-MR DD2 Magnetic Resonance System [400 MHz, Agilent, USA] or Varian/Oxford As-500 [500 MHz, Varian Assoc., Palo Alto, USA]. Chemical shifts values were recorded as parts per million (δ), referenced to tetramethylsilane (TMS) as the internal standard or to the residual solvent peak ($CDCl_3$, ^{1}H: 7.26, ^{13}C: 77.16, CD_3OD, ^{1}H: 3.31, ^{13}C: 49.00, DMSO-d_6, ^{1}H: 2.50, ^{13}C: 39.52). Multiplicities were indicated as follows: s (singlet), d (doublet), t (triplet), q (quartet); m (multiplet); dd (doublet of doublet); dt (doublet of triplet); td (triplet of doublets); br s (broad singlet) and so on. Coupling constants were reported in hertz (Hz). Low resolution mass spectra were obtained on a Finnigan Surveyor MSQ Plus LC/MS [Thermo] or LCQ LC/MS [Thermo] using the electrospray ionization (ESI) method. High resolution mass spectra were analyzed at the Mass Spectrometry Laboratory of National Instrumentation Center for Environmental Management (NICEM) in Seoul on a LCQ LC/MS [Thermo] using the electrospray ionization (ESI) method. Chiral HPLC were performed on a HP Agilent 1100 with a Chiralpack IA column (IA00CE-OG039, 4.6 mmϕ × 250 mmL, 5 μm). All commercially available reagents were used without further purification unless noted otherwise. Commercially available reagents were obtained from Sigma-Aldrich, TCI, Acros, Alfa Aesar or Beadtech. All solvents were purchased from commercial suppliers. Analytical thin-layer chromatography (TLC) was performed using Merck Kiselgel 60 F254 plates, and the components were visualized by observation under UV light (254 and 365 nm) or by treating the plates with ninhydrin followed by thermal visualization. Flash column chromatography was performed on Merck Kieselgel 60 (230–400 mesh).

General Solid-Phase Reaction Procedures.

Amine Substitution. Bromoacetal resins (100 mg, 1.8 mmol/g, 0.18 mmol) were loaded into each syringe with porous filter, and solutions of 3 different R_3-amines (20 equiv. in 2 mL of DMSO) were dispensed into each syringe. The reaction mixture was shaken at 60 ℃ in a rotating oven [Robbins Scientific] for 12 h. After the completion of the reaction which monitored by positive Chloranil test, the resin were washed extensively with DMF, MeOH and DCM sequentially (three times each) and dried in a high-vacuum desiccator.

Amino Acid Coupling. A reaction cocktail of *N*-Fmoc-AA-OH (nine different amines were used for each reaction such as Phe, Val, Met, Ile, Tyr, Leu, Arg(Pbf), Cys(Bzl) and 4-Cl-Phe, 4 equiv.), HCTU (4 equiv.), and DIPEA (6 equiv.) in DMF (2 mL per syringe) was added to each porous filter syringe charged with the resins. After the reaction mixture was shaken for 12 h at room temperature. After the completion of the reaction monitored by negative Chloranil test, the resins were washed extensively with DMF, MeOH and DCM sequentially (three times each) and dried in a high-vacuum desiccator.

Fmoc deprotection. Twenty percent piperidine in DMF was added to the resins in the syringe with porous filter, and the reaction mixture was shaken for 10 min at room temperature to unmask the Fmoc-protected primary amine. After the completion of the reaction monitored by positive ninhydrin (Kaiser) test, the resins were washed extensively with DMF, methanol, DCM and DMF, sequentially.

Acid Coupling. The activated ester for acid coupling was generated *in situ* by the activation of acid coupling partners (**methyl** or **tolyl**, 3 equiv.) with DIC (3 equiv.) and HOBt (3 equiv.) in DMF for 30 min. The resulting reaction cocktail was dispensed into the porous filter syringe charged with resins, and the reaction mixture was shaken for 12 h at room temperature. After the completion of the reaction monitored by negative ninhydrin (Kaiser) test, the resins were washed extensively with DMF, MeOH, and DCM sequentially and dried in a high-vacuum desiccator.

Cleavage and Cyclization. The resins in the porous filter syringe were first dried under high vacuum and then treated with 100% formic acid (3 mL per syringe) for 18 h at room temperature. After resin removal by filtration, the filtrate was condensed *in vacuo* to yield the desired product as an oil. The products were diluted with 50% water/acetonitrile and freeze-dried: a process that yielded a pale brown powder. The purity of the final products was observed by LC/MS without further purification

2. General information of bioassay

Reagents and materials

DMSO was purchased from Sigma-Aldrich. Micro BCA^{TM} Protein Assay Kit was purchased from PIERCE and was used for the measurement of protein concentration of cell lysate. Cell culture reagents including fatal bovine serum, culture media, and antibiotic-antimycotic solution were purchased from GIBCO, Invitrogen. The culturing dish or plates were purchased from CORNING. Developing for western blot analysis was performed by Amersham ECL Prime Western Blotting Detection System from GE Healthcare Life Science. TMB, a substrate of HRP conjugated in secondary antibody, was purchased from Invitrogen.

Antibodies, Plasmids and Proteins

Antibodies were obtained from the following sources: antibody to S6K1, phospho-S6K1 (T389), HRP-labeled anti-rabbit secondary antibodies from Abcam; anti-GST from Santa Cruz Biotechnology; antibodies to GAPDH from Cell Signaling Technology.

pAmCyan1-N1 containing LRS and pZsYellow1-N1 containing RagD plasmids for FRET imaging were generously provided by Prof. Sunghoon Kim (Seoul National University).

All proteins including his-tagged LRS, GST-tagged RagD were laboratory stocks. Transfection was performed using calcium precipitation method.

Instruments and programs

For developing of ELISA-based assay, the absorbance of 96-well plate was measured by BioTek Synergy HT Microplate reader.

Chemiluminescent signal was monitored by ChemiDoc^{TM} MP imaging system [Bio-Rad] and quantified by ImageLab 4.0 program.

FRET imaging was carried with DeltaVision Elite imaging system [GE Healthcare] equipped with a sCMOS camera. Objective lenses are supported by Olympus IX-71 [Olympus] inverted microscope equipped with Plan APO 60X/Oil (PLAPON60×O), 1.42 NA, WD 0.15 mm. DeltaVision Elite uses a solid state illumination system, InSightSSI fluorescence illumination module. Four-color standard filter set [GE Healthcare, 52-852113-003] was used to detect fluorescence signals. For live cell continuous monitoring, CO_2 supporting chamber along with an objective air heater was set and ultimatefocus hardware autofocus module was incorporated to maintain the sample z-position during timelapse imaging. FRET analysis was analyzed by SoftWorks program and all graphs were figured by GraphPad Prism 5.

II. Tetra-substituted pyrazinotriazinedione library as β-turn mimetic

FIG S1. Alignment of an energy-minimized structure of representative compound **7** with the peptide backbone structure phospholipase A2 at five atom positions of representative compound **7** (pink) versus $C\alpha_i$, $C\alpha_{i+1}$, $C\alpha_{i+2}$, $C\alpha_{i+3}$, carbons and $N_{(i+2)}$ (green) [V_{conf} interface, Discovery Studio 4.1]

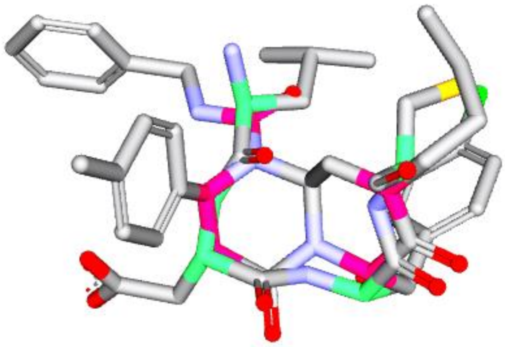

III. Synthesis and characterization of acid partners

1. General procedure for the preparation of tolyl acid partners

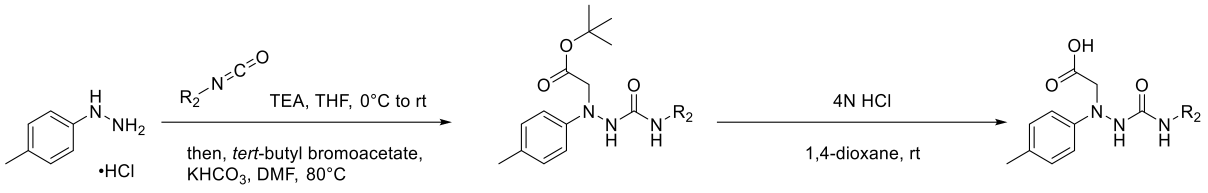

A-1

To a stirred suspension of *p*-tolylhydrazine hydrochloride in tetrahydrofuran (THF) under ice-water external bath, triethylamine was carefully added. Isocyanate (0.5 equiv.) was added dropwise to the solution. The resulting mixture was stirred at 0 °C and warmed up to room temperature for several hours. After the completion of the reaction monitored by TLC, the solvent was removed under reduced pressure. Ethyl acetate was added and the organic layer was washed with saturated NaCl (aq.) and saturated ammonium chloride (aq.). The organic layer was dried over anhydrous $MgSO_4$ (s) and filtered. The organic solvent was evaporated under vacuum. The resulting mixture was purified through the recrystallization by ethyl acetate and n-hexane to afford a desired solid. Then, the resulting solid, $KHCO_3$ and *tert*-butyl bromoacetate were dissolved in DMF and stirred at 80 °C for several hours. After completion of the reaction indicated by TLC, the reaction mixture was washed with saturated NaCl (aq.) and ammonium chloride (aq.) and extracted with ethyl acetate. The combined organic layer was condensed was dried over anhydrous $MgSO_4$ and filtered. The filtrate was condensed under reduced pressure, followed by silica-gel flash column chromatography to afford a desired solid. The resultant was dissolved in 1,4-dioxane and 4N HCl was added slowly. The reaction mixture was stirred at room temperature for several hours. After completion of the reaction indicated by TLC, the solution was concentrated under reduced pressure. The saturated $NaHCO_3$ (aq.) was added and the aqueous layer was washed with ethyl acetate. Concentrated HCl was added dropwise slowly at 0 °C (pH 2–3). The mixture was extracted with ethyl acetate (EA), and the organic layer was dried over anhydrous $MgSO_4$(s) and evaporated. The residue was purified by recrystalization with ethyl acetate and n-hexane to give the desired product **A-1** (30~35% overall yields in three steps).

2. General procedure for the preparation of methyl acid partners

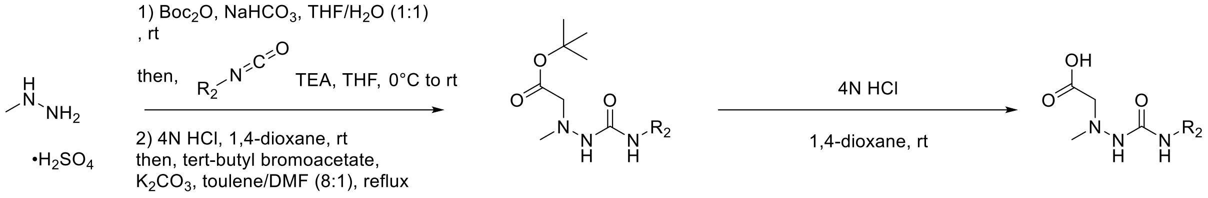

To a stirred suspension of methylhydrazine sulfate in water (200 ml) under ice-water external bath, $NaHCO_3$ was carefully added. Di-*tert*-butyl dicarbonate in tetrahydrofuran (200 mL) was added to the solution. The resulting mixture was left to stir and allowed to warm to room temperature for overnight. After completion of the reaction indicated by TLC, the organic layer was extracted with ethyl acetate. The combined solution was dried over anhydrous $MgSO_4$(s) and filtered, and evaporated *in vacuo* to afford pale yellow oily compound without further purification. The resultant was dissolved in THF and isocyanate was added dropwise. The resulting mixture was stirred at 0 °C and warmed up to room temperature for several hours. After the completion of the reaction monitored by TLC, the solvent was removed under reduced pressure. The reaction mixture was purified through the recrystallization by ethyl acetate and n-hexane to afford a desired solid. The resulting solid dissolved in 1,4-dioxane and 4N HCl was added slowly. The reaction mixture was stirred at room temperature for several hours. After completion of the reaction indicated by TLC, the solution was concentrated under reduced pressure. The saturated $NaHCO_3$ (aq.) was added slowly (pH 11–12) under ice-water external bath. The aqueous layer was extracted by ethyl acetate, and it was dried over anhydrous $MgSO_4$(s) and filtered. The residue was evaporated *in vacuo* and purified by recrystallization with ethyl acetate and n-hexane to afford a desired solid. Then, the resulting solid, K_2CO_3 and *tert*-butyl bromoacetate were dissolved in toluene/*N,N*-dimethylformamide (8:1), and stirred and refluxed for several hours. After completion of the reaction indicated by TLC, the reaction mixture was washed with saturated NaCl (aq.) and ammonium chloride (aq.) and extracted with ethyl acetate. The combined organic layer was condensed was dried over anhydrous $MgSO_4$ and filtered. The filtrate was condensed under reduced pressure, followed by silica-gel flash column chromatography to afford the desired solid. The resultant was dissolved in 1,4-dioxane and 4N HCl was added slowly. The reaction mixture was stirred at room temperature for several hours. After completion of the reaction indicated by TLC, the solution was concentrated under reduced pressure. The saturated $NaHCO_3$ (aq.) was added and the aqueous layer was washed with ethyl acetate. Concentrated HCl was added dropwise slowly at 0 °C (pH 2–3). The mixture was extracted with ethyl acetate, and the organic layer was dried over anhydrous $MgSO_4$(s) and evaporated. The residue was purified by recrystalization with ethyl acetate and n-hexane to give the desired product **A-2** (44~51% overall yields in 4 steps).

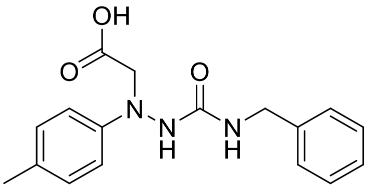

A-1(a): Yield: 33% (three steps); ^{1}H NMR (500 MHz, DMSO-*d*6) δ 13.14 (brs, 1H), 8.04 (brs, 1H), 7.39 (brs, 1H), 7.25 (m, 5H), 7.04 (d, *J* = 8.8 Hz, 2H), 6.64 (d, *J* = 8.5 Hz, 2H), 4.25 (d, *J* = 6 Hz, 2H), 4.14 (brs, 2H), 2.20 (s, 3H); ^{13}C NMR (125 MHz, DMSO-*d*6) δ 172.2, 158.5, 146.4, 140.3, 129.4, 128.2, 126.9, 126.6, 112.4, 56.8, 42.6, 29.0; LRMS (ESI+) Calcd for $C_{17}H_{20}N_3O_3$+ $[M+H]^+$: 314.36, found: 314.00.

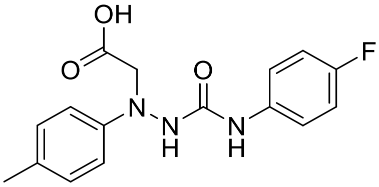

A-1(b): Yield: 30% (three steps); ^{1}H NMR (400 MHz, DMSO-*d*6) δ 13.11 (brs, 1H), 9.27 (brs, 1H), 8.42 (brs, 1H), 7.48 (dd, *J* = 8 Hz, *J* = 4.9 Hz, 2H), 7.08 (m, 4H), 6.69 (d, *J* = 8.2 Hz, 2H), 4.28 (brs, 2H), 2.20 (s, 3H); ^{13}C NMR (100 MHz, DMSO-*d*6) δ 172.9, 158.6, 156.2, 155.8 (d, $^{1}J_{C,F}$ = 44.8 Hz), 146.2, 135.9, 129.5, 128.0, 119.96, 119.89 (d, $^{3}J_{C,F}$ = 7.5 Hz), 115.3, 115.1 (d, $^{2}J_{C,F}$ = 22.1 Hz), 112.43, 56.1, 20.0; LRMS (ESI+) Calcd for $C_{16}H_{17}FN_3O_3$+ $[M+H]^+$:318.32, found: 317.93.

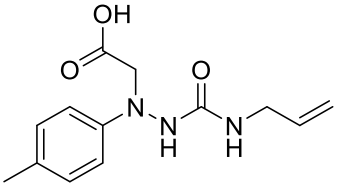

A-1(c): Yield: 35% (three steps); ^{1}H NMR (500 MHz, DMSO-d6) δ 13.12 (brs, 1H), 8.01 (brs, 1H), 7.03 (d, *J* = 8.3 Hz, 2H), 6.98 (brs, 1H), 6.62 (d, *J* = 8.3 Hz, 2H), 5.78 (m, 1H), 5.07 (dd, *J* = 17.1 Hz, *J* = 1.5 Hz, 1H), 5.00 (dd, *J* = 10.3 Hz, *J* = 1.5 Hz, 1H), 4.17 (brs, 2H), 3.68 (m, 2H), 2.20 (s, 3H); ^{13}C NMR (125 MHz, DMSO-d6) δ 172.4, 158.3, 146.3, 136.2, 129.4, 127.7, 114.5, 112.3, 55.9, 41.4, 20.0; LRMS (ESI) *m/z* calcd for $C_{13}H_{18}N_3O_3$+ [M+H]+:264.30; Found: 264.08

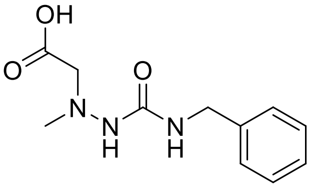

A-2(a): Yield: 44% (four steps); ^{1}H NMR (400 MHz, $CDCl_3$) δ 11.38 (brs, 1H), 7.92 (s, 1H), 7.32 (m, 5H), 6.46 (t, *J* = 6.1 Hz, 1H), 4.44 (d, *J* = 5.9 Hz, 2H), 3.66 (d, *J* = 17.2 Hz, 1H), 3.43 (d, *J* = 16.4 Hz, 1H), 2.85 (s, 3H); ^{13}C NMR (100 MHz, $CDCl_3$) δ 173.0, 159.7, 138.8, 128.7, 127.4, 57.8, 44.7, 43.6; LRMS (ESI+) Calcd for $C_{11}H_{16}N_3O_{3+}$ $[M+H]^+$:238.26, found: 238.03

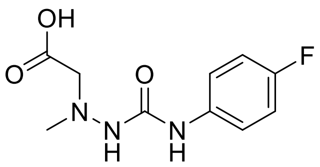

A-2(b): Yield: 44%(four steps); ^{1}H NMR (500 MHz, DMSO-*d*6) δ 12.64 (brs, 1H), 8.83 (s, 1H), 7.56 (s, 1H), 7.49 (m, 2H), 7.08 (m, 2H), 3.51 (brs, 2H), 2.61 (s, 3H); ^{13}C NMR (100 MHz, DMSO-*d*6) δ 171.5, 158.5, 156.2, 155.3, 136.05, 136.02 (d, $^{3}J_{CF}$ = 2.3 Hz) 120.1, 120.0 (d, $^{2}J_{CF}$ = 7.6Hz), 115.2, 115.0 (d, $^{1}J_{CF}$ = 22 Hz), 60.1, 45.9; LRMS (ESI+) Calcd for $C_{10}H_{13}FN_3O_3$+ $[M+H]^+$:242.22, found: 242.03

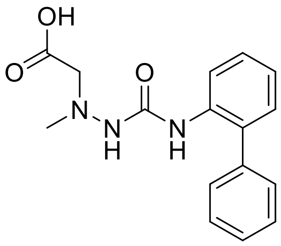

A-2(d): Yield: 51%(four steps); ^{1}H NMR (400 MHz, DMSO-*d*6) δ 12.46 (brs, 1H), 8.60 (brs, 1H), 8.14 (d, *J* = 8.2 Hz, 2H), 7.43 (m, 6H), 7.30 (t, *J* = 7.8 Hz, 1H), 7.22 (d, *J* = 7.4 Hz, 1H), 7.07 (t, *J* = 7.4 Hz, 1H), 3.33 (brs, 2H), 2.38 (s, 3H); ^{13}C NMR (100 MHz, DMSO-*d*6) δ 170.5, 154.8, 138.1, 136.0, 129.8, 129.0, 128.7, 127.9, 127.5, 122.3, 119.7, 59.4, 45.4; LRMS (ESI+) Calcd for $C_{16}H_{18}N_3O_3$+ $[M+H]^+$:300.33, found: 300.21.

IV. Characterization for representative compounds

1. Representative compound 7 (5a{2,9})

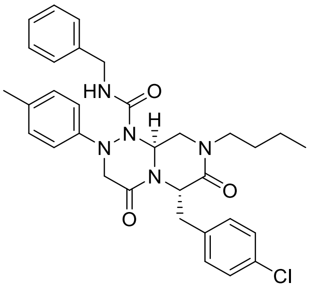

Representative Compound 7 (5a{2,9}): ^{1}H NMR (400 MHz, $CDCl_3$) δ 7.35 (m, 3H), 7.26 (m, 2H), 7.16 (m, 4H), 7.09 (d, *J* = 8.6 Hz, 2H), 6.77 (d, *J* = 8.6 Hz, 2H), 6.51 (t, *J* = 6.1 Hz, 1H), 5.99 (dd, *J* = 9.4 Hz, *J* = 5.5 Hz, 1H), 5.24 (fd, *J* = 8.6 Hz, *J* = 5.1 Hz, 1H), 4.62 (dd, *J* = 14.9 Hz, *J* = 6.7 Hz, 1H), 4.36 (dd, *J* = 14.9 Hz, *J* = 5.5 Hz, 1H), 4.1 (d, *J* = 17.2, 1H), 3.67 (d, *J* = 17.2 Hz, 1H), 3.32 (m, 2H), 3.10 (m, 4H), 2.28 (s, 3H), 1.33 (m, 2H), 1.26 (m, 2H), 0.86 (t, *J*=7.2 Hz, 3H); ^{13}C NMR (100 MHz, $CDCl_3$) δ 165.5, 162.0, 156.5, 144.7, 138.6, 134.8, 133.0, 132.4, 131.0, 130.5, 120.0, 128.7, 127.9, 127.5, 114.7, 60.2, 56.1, 50.0, 48.7, 47.1, 44.6, 36.4, 28.9, 20.6, 20.0, 13.8; HRMS (ESI) *m/z* calcd for $C_{32}H_{37}ClN_5O_3$+ $[M+H]^+$: 574.2579; Found: 574.2569.

Crude LC-MS data

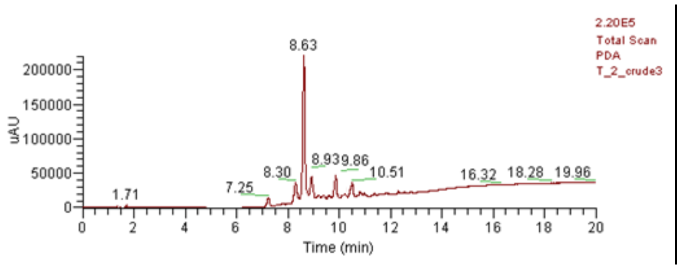

Mass spectrum

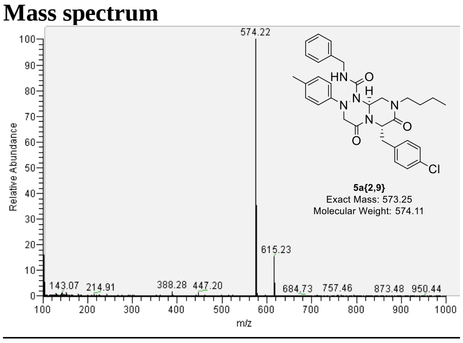

After short filter

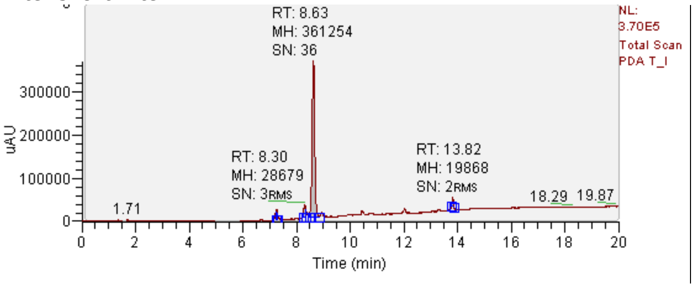

Crude ^{1}H NMR

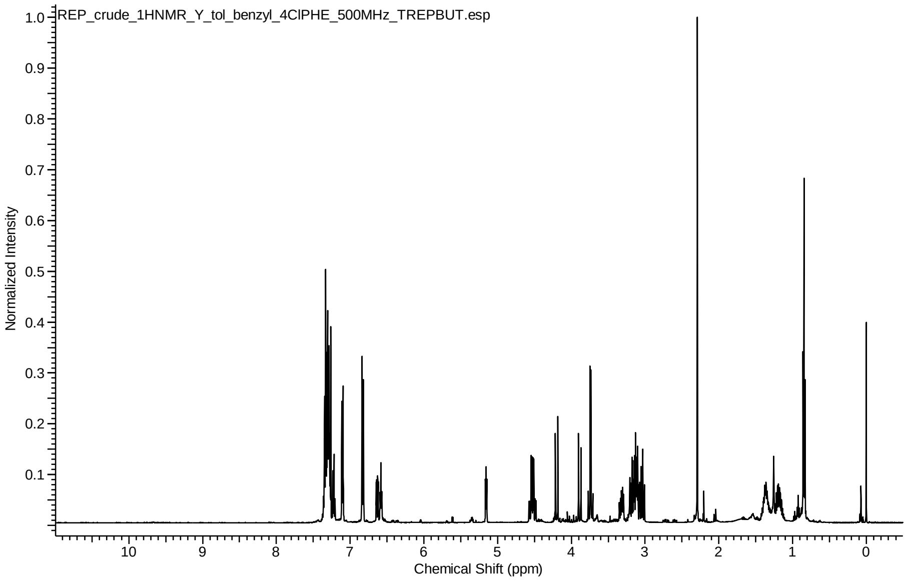

Chiral HPLC

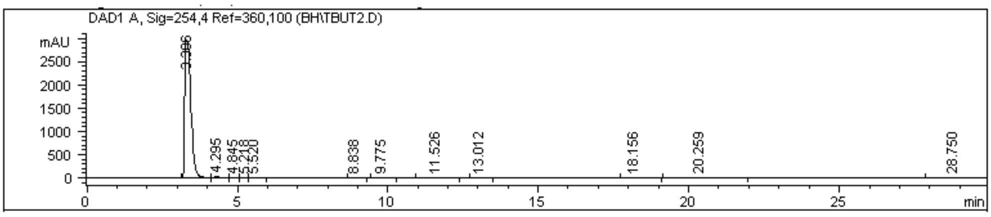

gCOSY

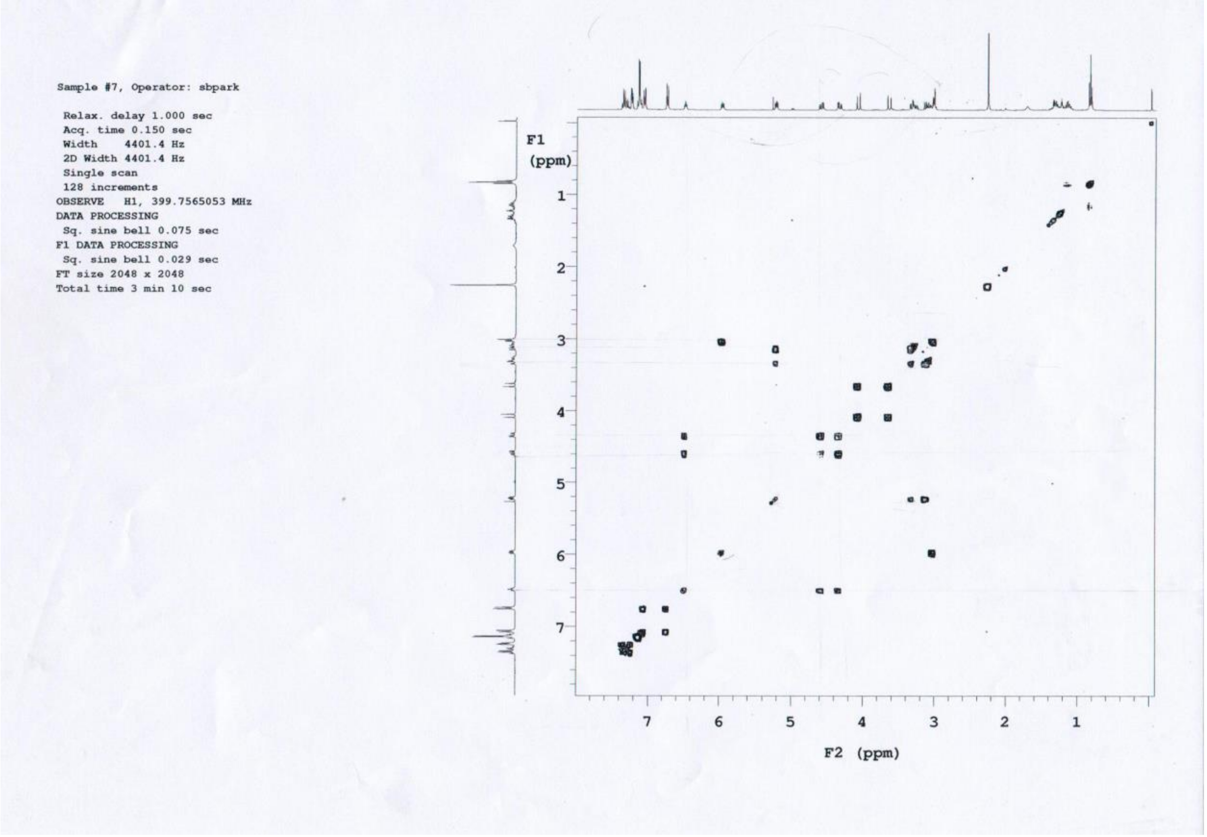

2. Representative compound 8 (5a{2,2})

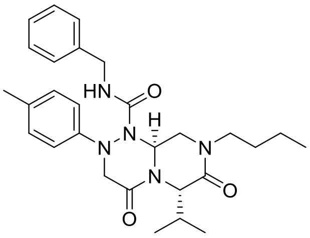

Representative Compound 8 (5a{2,2}): ^{1}H NMR (500 MHz, $CDCl_3$) δ 7.32 (m, 2H), 7.26 (m, 3H), 7.10 (d, *J* = 8.3 Hz, 2H), 6.80 (d, *J* = 8.3 Hz, 2H), 6.52 (t, *J* = 5.9 Hz, 1H), 6.40 (t, *J* = 7.3 Hz, 1H), 4.94 (d, *J* = 5.9 Hz, 1H), 4.49 (m, 2H), 4.25 (d, *J* = 17.1 Hz, 1H), 4.00 (d, *J* = 17.1 Hz, 1H), 3.35 (m, 1H), 3.14 (d, *J* = 8.7 Hz, 2H), 3.01 (ddd, *J* = 13.7 Hz, *J* = 8.1 Hz, *J* = 6.1 Hz, 1H), 2.43 (m, 1H), 2.28 (s, 3H), 1.34 (m, 2H), 1.17 (m, 2H), 1.11 (d, *J* = 8.6 Hz, 3H), 1.05 (d, *J* = 6.8 Hz, 3H), 0.85 (t, *J* = 7.1 Hz, 3H); ^{13}C NMR (100 MHz, $CDCl_3$) δ 166.0, 162.6, 156.9, 144.7, 138.3, 132.0, 130.5, 128.9, 127.7, 127.4, 114.2, 60.9, 59.8, 49.9, 48.6, 46.9, 44.7, 32.3, 29.0, 20.5, 20.03, 19.97, 19.4, 13.8; HRMS (ESI) *m/z* calcd for $C_{28}H_{38}N_5O_3$+ $[M+H]^+$: 492.2969; Found: 492.2961.

Crude LC-MS data

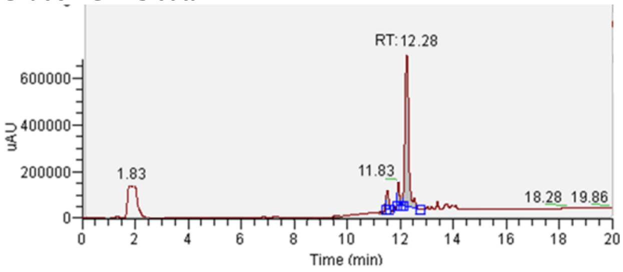

After short filter

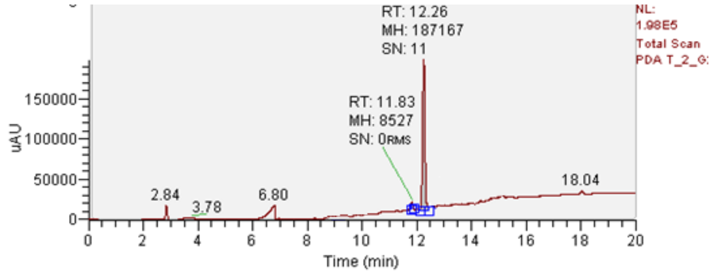

Mass spectrum

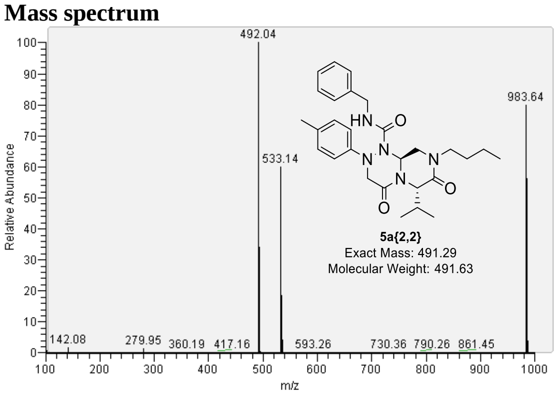

Crude ^{1}H NMR

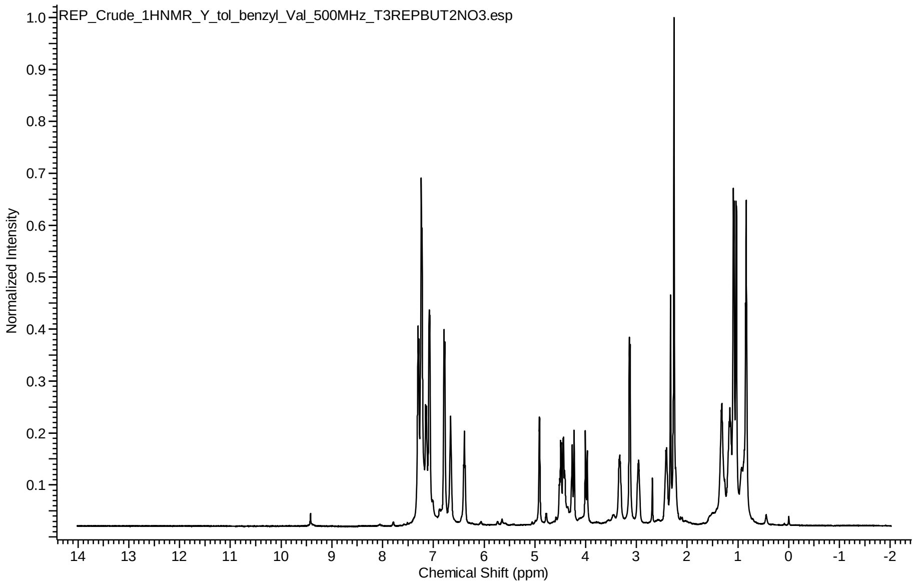

Chiral HPLC

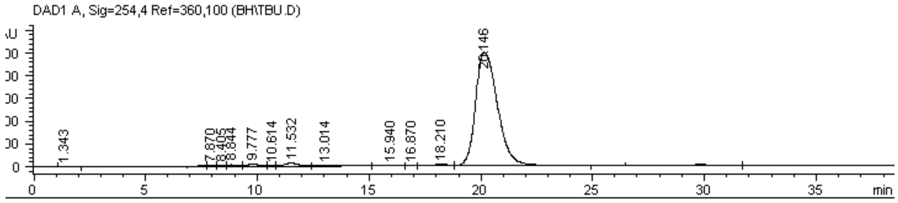

gCOSY

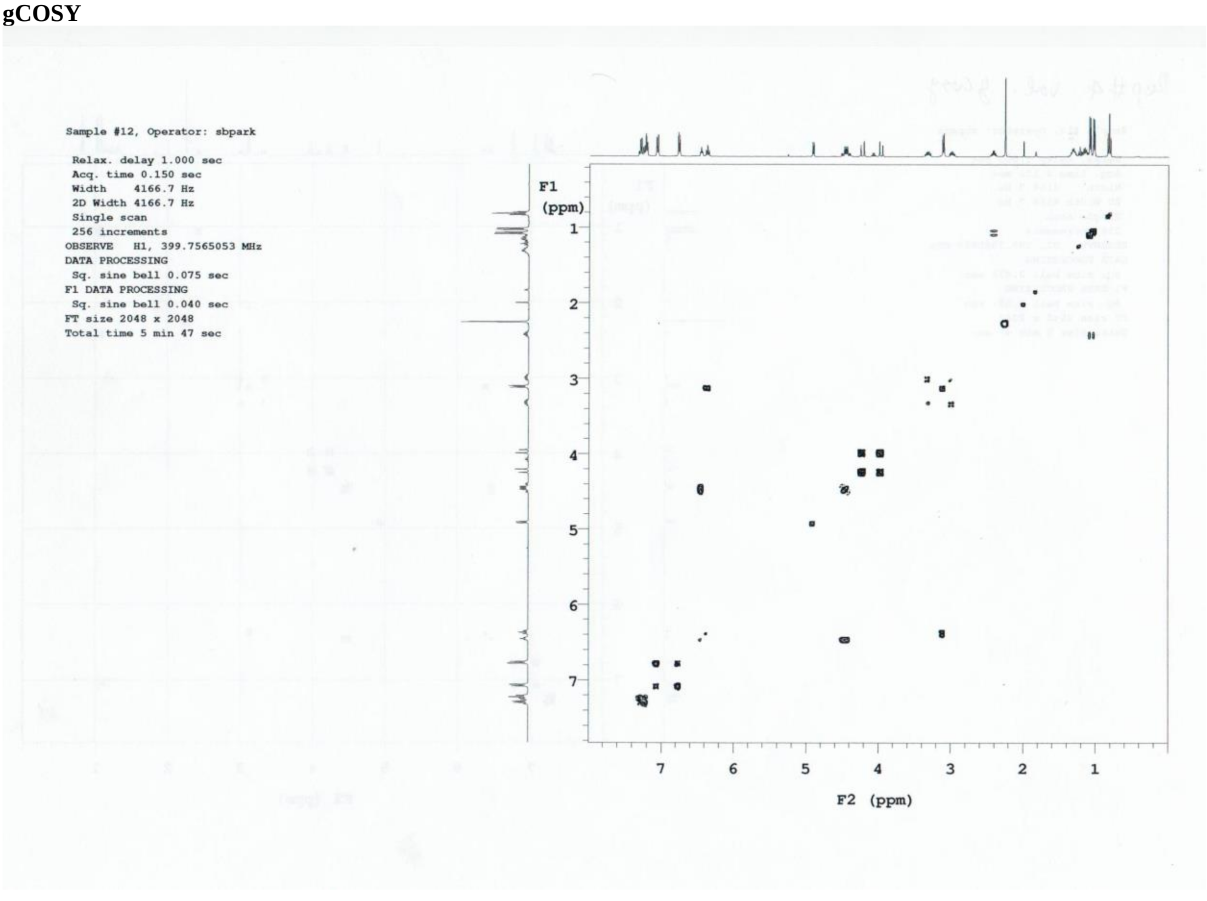

3. Representative compound 9 (5a{1,6})

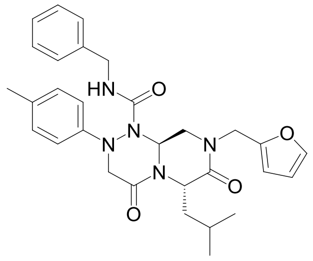

Representative Compound 9 (5a{1,6}): ^{1}H NMR (400 MHz, $CDCl_3$) δ 7.3 (m, 3H), 7.23 (d, *J* = 7.8 Hz, 3H), 7.06 (d, *J* = 8.6 Hz, 2H), 6.76 (d, *J* = 8.2 Hz, 2H), 6.46 (t, *J* = 5.9 Hz, 1H), 6.30 (dd, *J* = 11.2 Hz, *J* = 4.1 Hz, 1H), 6.24 (m, 1H), 6.11 (d, *J* = 3.1 Hz, 4H), 5.20 (dd, *J* = 9.6 Hz, *J* = 4.5 Hz, 1H), 4.48 (m, 2H), 4.38 (m, 2H), 4.23 (d, *J* = 17.2 Hz, 1H), 3.93 (d, *J* = 17.6 Hz, 1H), 3.19 (dd, *J* = 11.7 Hz, *J* = 4.3 Hz, 1H), 3.09 (t, *J* = 11.2 Hz, 1H), 2.29 (s, 3H), 1.82 (m, 2H), 1.68 (m, 1H), 1.01 (d, *J* = 6.3 Hz, 3H), 0.97 (d, *J* = 6.7 Hz, 3H); ^{13}C NMR (100 MHz, $CDCl_3$) δ 167.1, 162.1, 156.7, 149.4, 144.7, 142.6, 138.3, 132.1, 130.5, 128.9, 127.8, 127.5, 114.4, 110.4, 109.1, 59.2, 53.8, 49.6, 48.8, 44.7, 42.9, 41.0, 25.16, 23.15, 22.14, 20.6; LRMS (ESI) *m/z* calcd for $C_{30}H_{36}N_5O_4$+ [M+H]$^+$: 530.27; Found: 530.03.

Crude LC-MS data

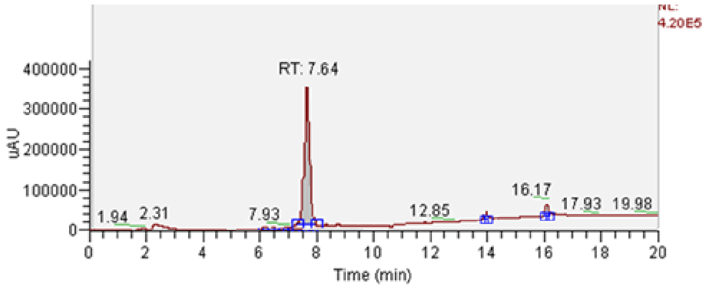

After short filter

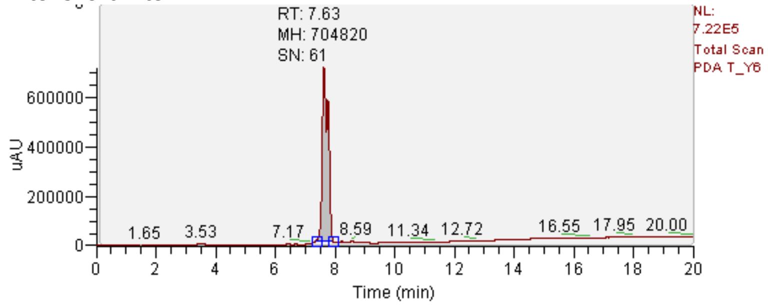

Mass spectrum

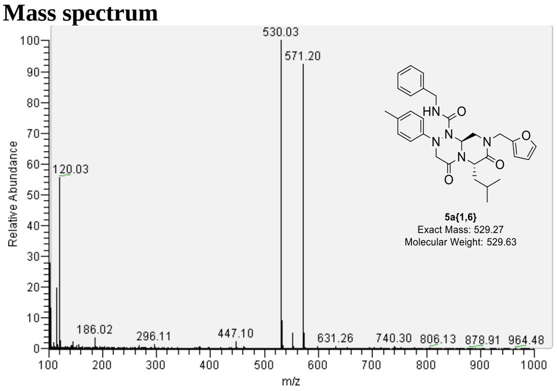

Crude ^{1}H NMR

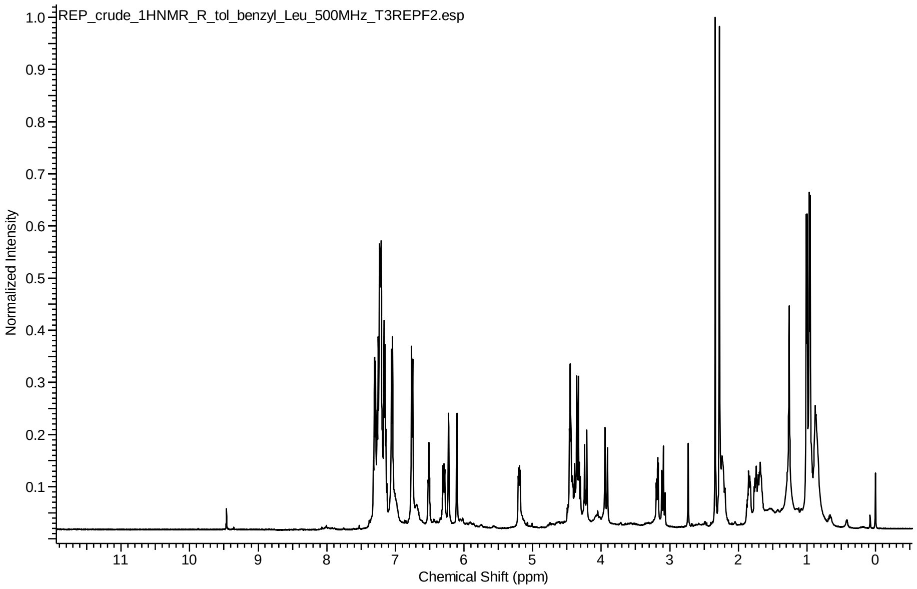

Chiral HPLC

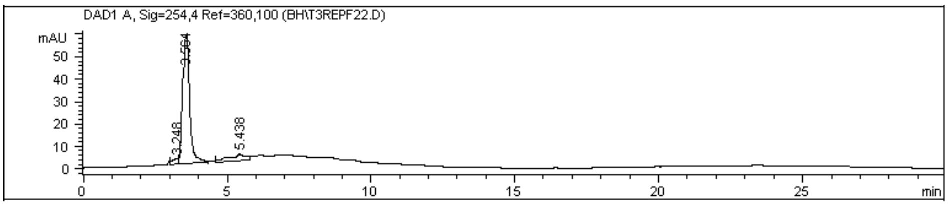

gCOSY

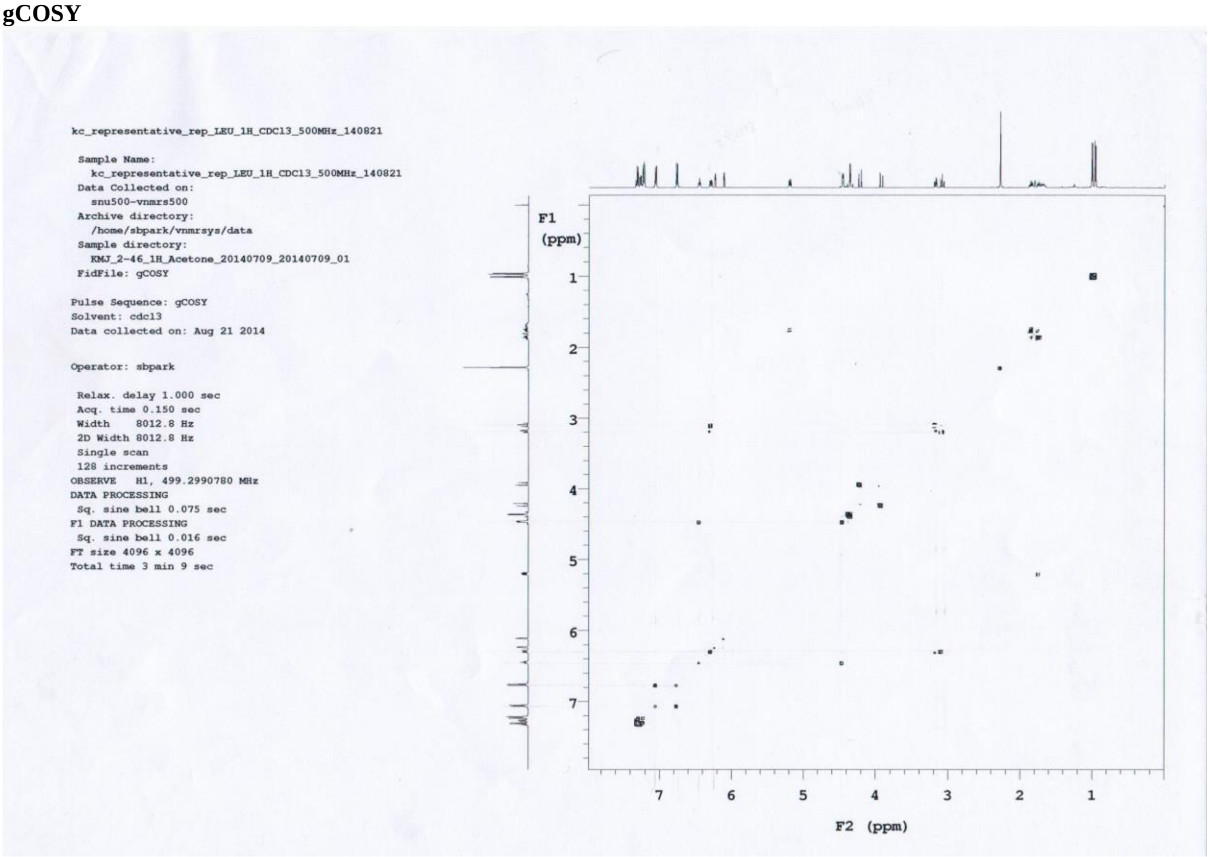

2D NOE NMR

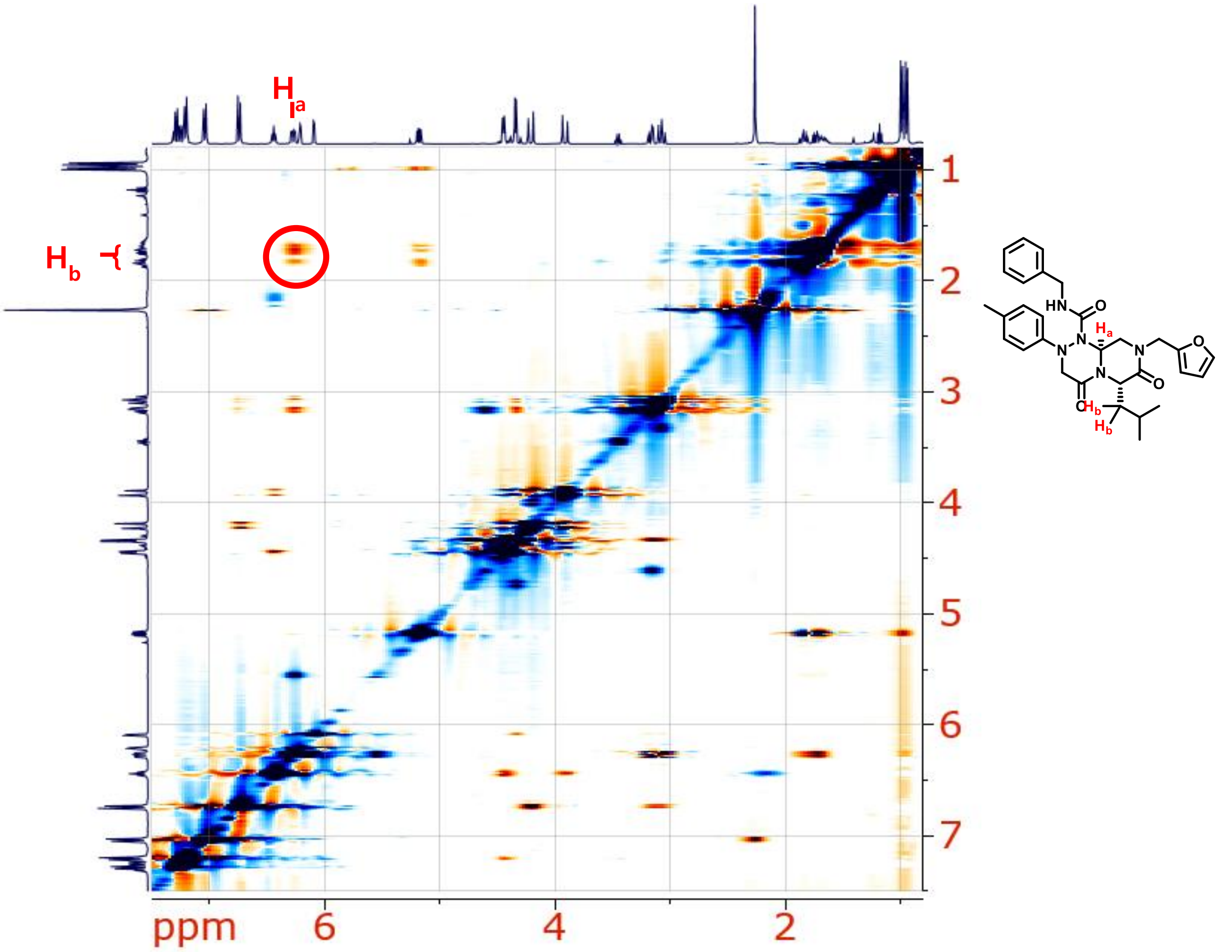

4. Representative compound 10 (5c{3,1})

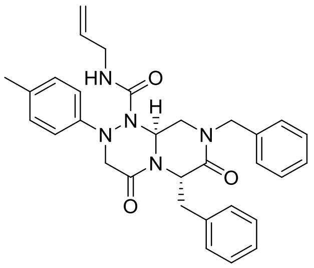

Compound 10 (5c{3,1}): ^{1}H NMR (400 MHz, CDCl3) δ 7.24 (m, 8H), 7.01 (d, *J* = 7.8 Hz, 4H), 6.71 (d, *J* = 8.6 Hz, 2H), 6.18 (t, *J* = 6.1 Hz, 1H), 5.84 (m, 2H), 5.35 (dd, *J* = 7.4 Hz, *J* = 5.1 Hz, 1H), 5.13 (m, 2H), 4.65 (d, *J* = 14.5 Hz, 1H), 4.19 (d, *J* = 14.5 Hz, 1H), 4.11 (d, J = 17.6 Hz, 1H), 3.90 (m, 2H), 3.73 (d, *J* = 17.2Hz, 1H), 3.42 (dd, *J* = 13.7 Hz, *J* = 5.1 Hz, 1H), 3.30 (dd, *J* = 13.7 Hz, *J* = 7.8 Hz, 1H), 2.91 (d, *J* = 7.4 Hz, 2H), 2.26 (s, 3H); ^{13}C NMR (100 MHz, CDCl3) δ 166.0, 162.2, 156.0, 144.8, 136.3, 135.5., 134.5, 132.5, 130.4, 129.9, 128.8, 128.6, 127.9, 127.8, 127.3, 116.2, 114.9, 60.4, 56.6, 50.2, 49.5, 48.9, 42.9, 37.0, 20.6; HRMS (ESI) *m/z* calcd for $C_{31}H_{34}N_5O_3$+ $[M+H]^+$: 524.2656; Found: 524.2656.

Crude LC-MS data

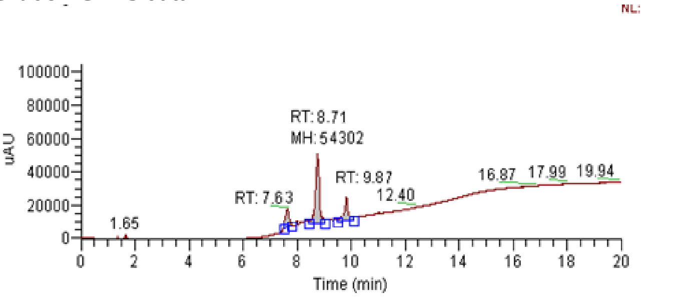

After short filter

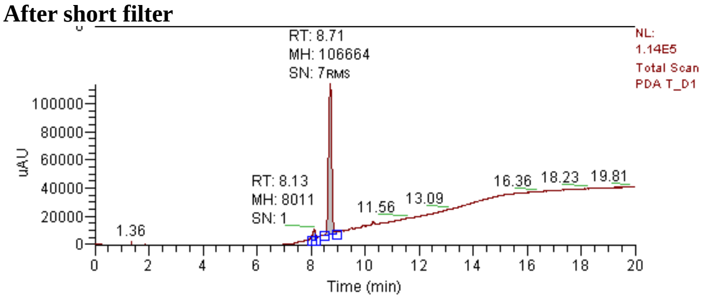

Mass spectrum

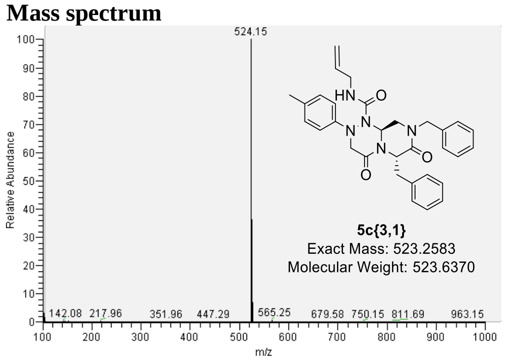

Crude ^{1}H NMR

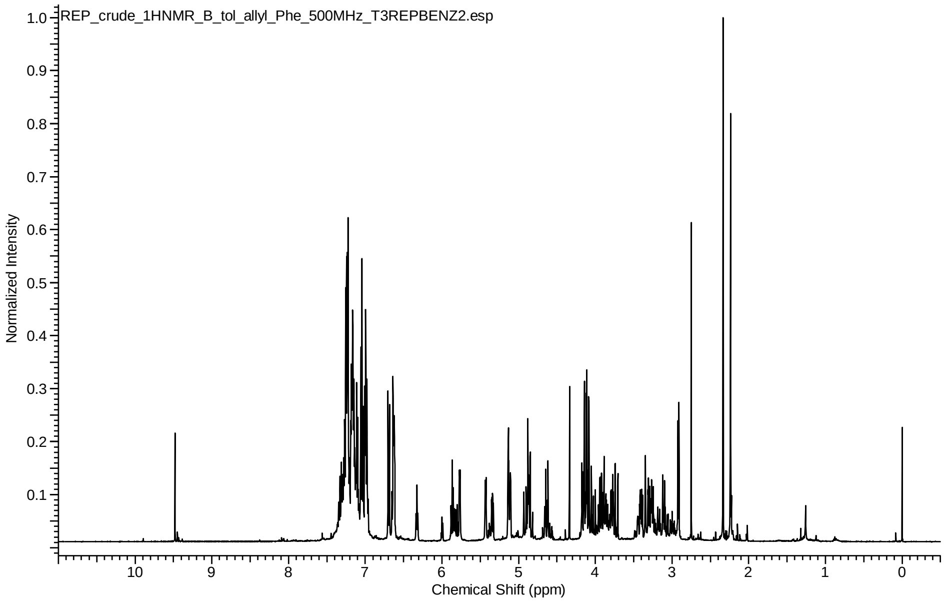

Chiral HPLC

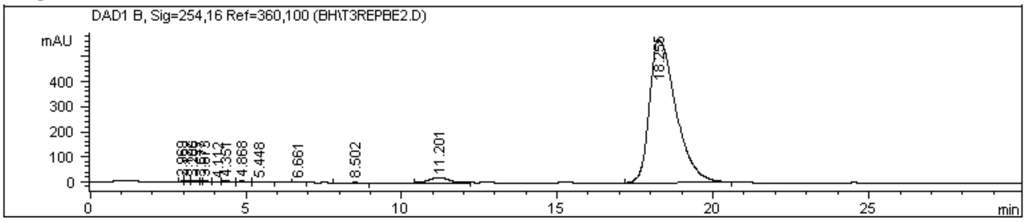

gCOSY

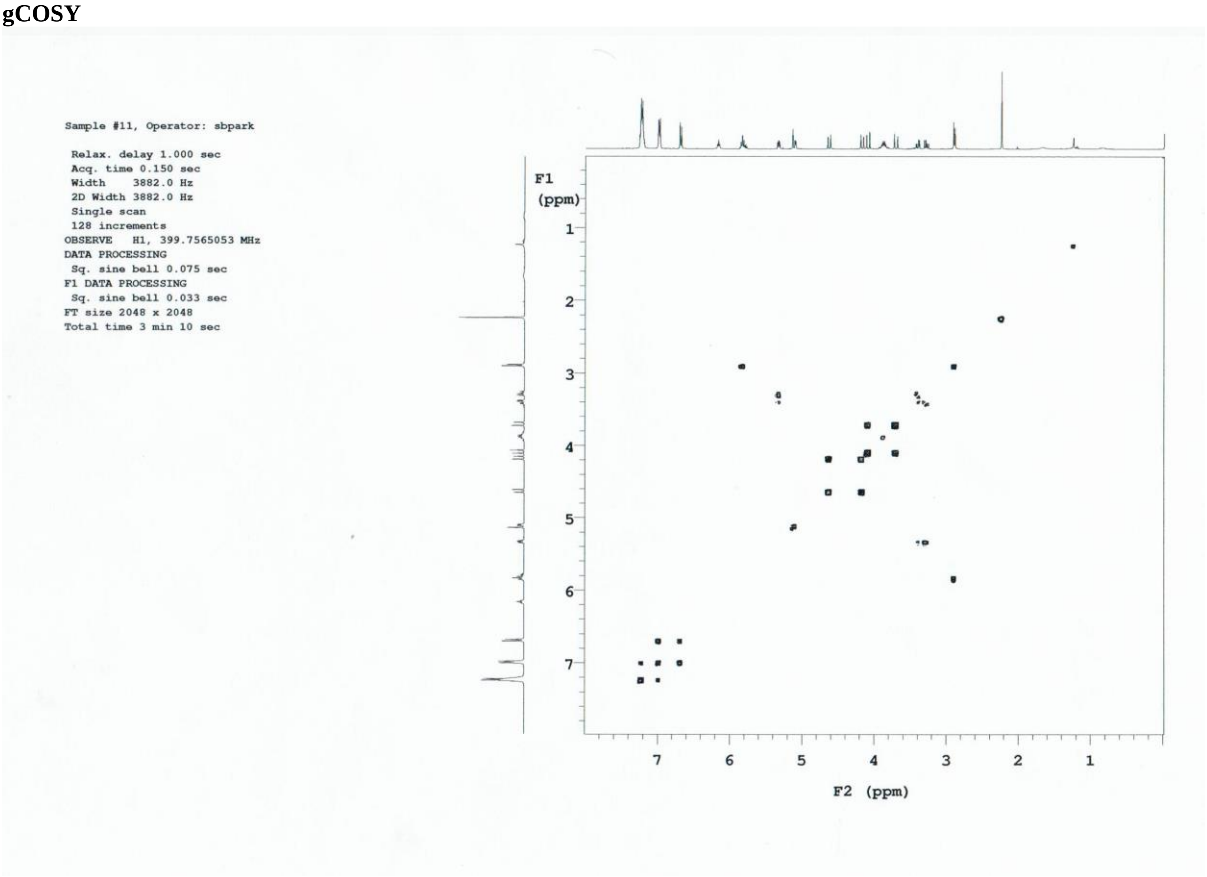

5. Representative compound 11 (5c{3,9})

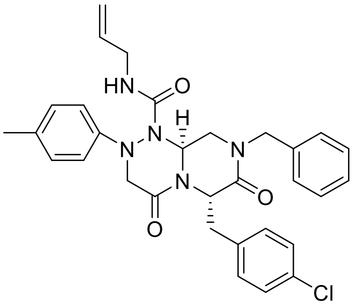

Compound 11 (5c{3,9}) : ^{1}H NMR (400 MHz, $CDCl_3$) δ 7.25 (m, 3H), 7.29 (m, 4H), 7.01 (m, 4H), 6.70 (d, J = 8.6 Hz, 2H), 6.19 (t, J = 5.9 Hz, 1H), 6.06 (dd, J = 8.6 Hz, J = 6.3 Hz, 1H), 5.64 (m, 1H), 5.36 (dd, J = 8.6 Hz, J = 4.7 Hz, 1H), 5.14 (m, 2H), 4.61 (d, J = 14.5 Hz, 1H), 4.20 (d, J = 14.9 H, 1H), 4.11 (d, J = 17.2 Hz, 1H), 3.90 (m, 2H), 3.72 (d, J = 17.6 Hz, 1H), 3.39 (dd, J = 13.7 Hz, J = 4.7 Hz, 1H), 3.19 (dd, J = 13.9 Hz, J = 8.8 Hz, 1H), 2.97 (m, 2H), 2.26 (s, 3H); ^{13}C NMR (100 MHz, $CDCl_3$) δ 165.8, 162.1, 156.1, 144.6, 135.4, 134.8, 134.3, 133.0, 132.3, 131.2, 130.4, 128.8, 128.7, 128.0, 127.9, 116.3, 114.5, 60.0, 58.1, 50.2, 49.4, 48.5, 42.9, 36.6, 20.6; LRMS (ESI) m/z calcd for $C_{31}H_{33}ClN_5O_3$+ $[M+H]^+$: 558.22; Found: 558.20.

Crude LC-MS data

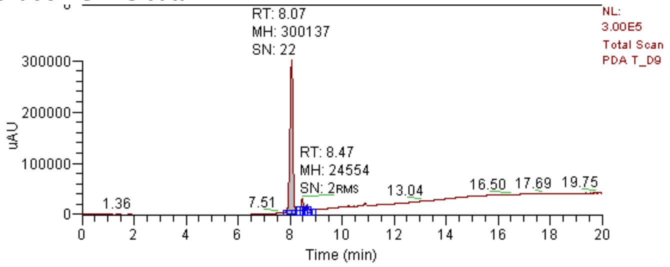

Mass spectrum

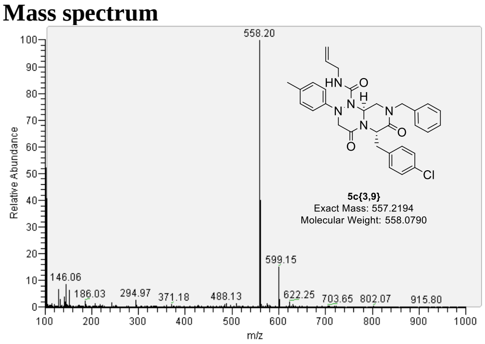

Chiral HPLC

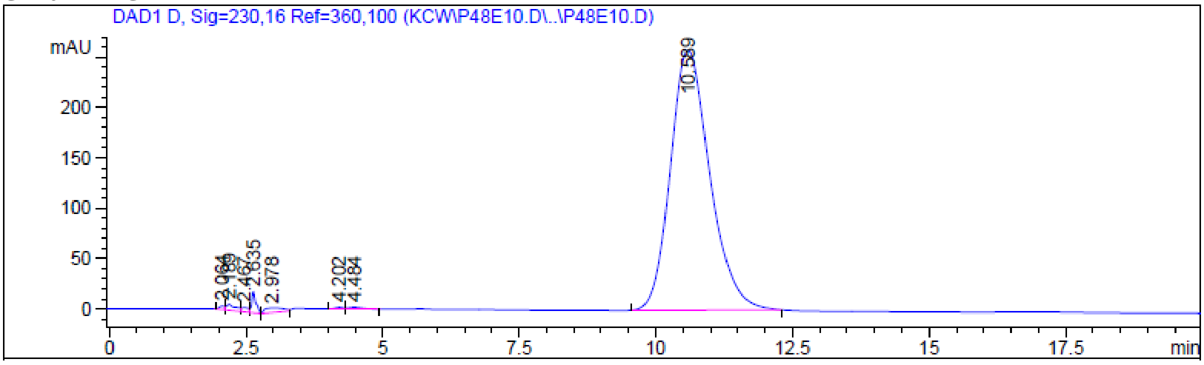

gCOSY

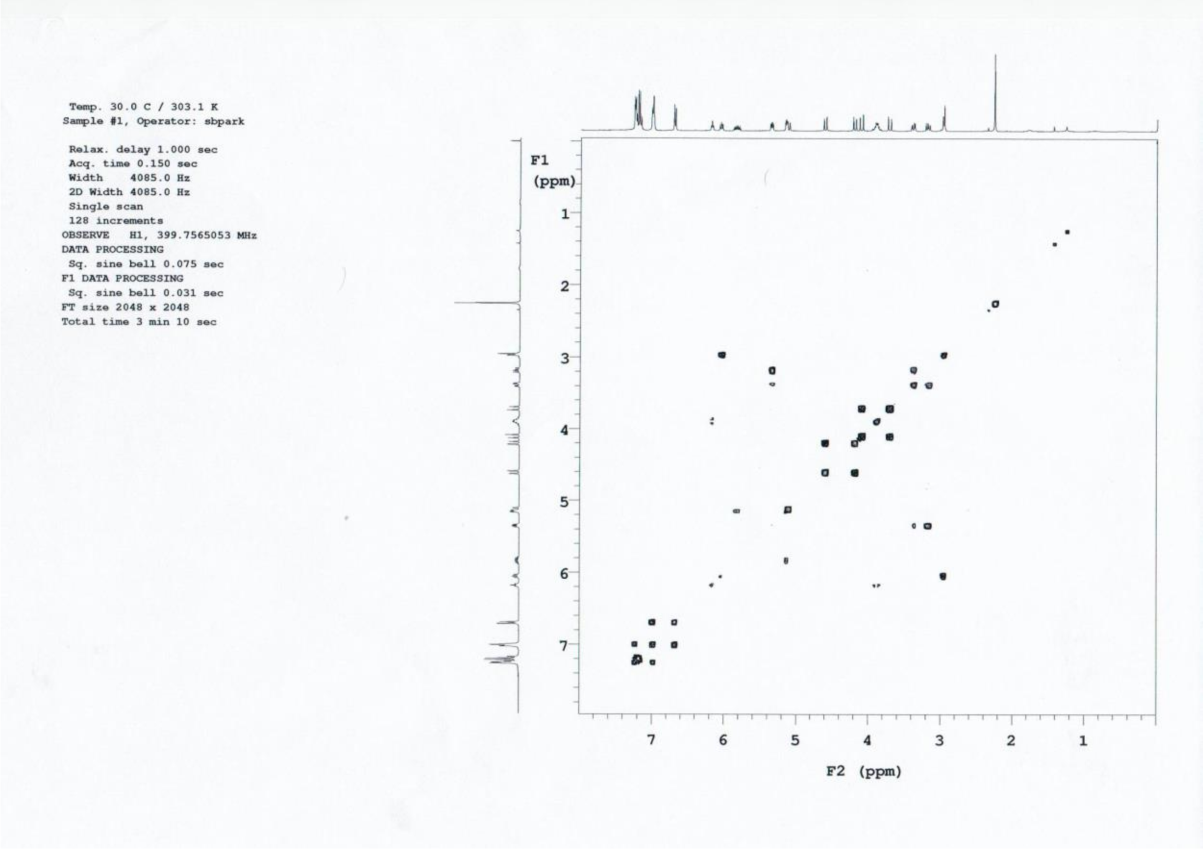
Temp. 30.0 C / 303.1 K
Sample #1, Operator: sbpark
Relax. delay 1.000 sec
Acq. time 0.150 sec
Width 4085.0 Hz
2D Width 4085.0 Hz
Single scan
128 increments
OBSERVE H1, 399.7565053 MHz
DATA PROCESSING
Sq. sine bell 0.075 sec
F1 DATA PROCESSING
Sq. sine bell 0.031 sec
FT size 2048 x 2048
Total time 3 min 10 sec
F1 (ppm)
F2 (ppm)

6. Representative compound 12 (6b{1,7})

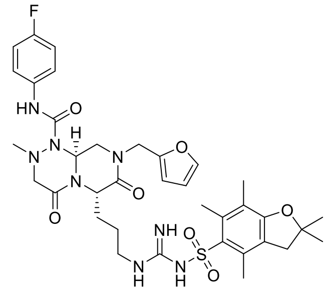

Representative Compound 12 (6b{1,7}): ^{1}H NMR (400 MHz, $CDCl_3$) δ 8.50 (s, 1H), 7.39 (m, 3H), 6.98 (t, *J* = 8.4 Hz, 2H), 6.30 (m, 5H), 6.05 (dd, J = 9.8 Hz, J = 4.3 Hz, 1H), 5.13 (t, J = 6.7 Hz, 1H), 4.77 (d, J = 15.3 Hz, 1H), 4.36 (d, J = 15.7 Hz, 1H), 3.89 (d, J = 17.2 Hz, 1H), 3.70 (m, 1H), 3.60 (m, 1H), 3.36 (d, J = 17.2 Hz, 1H), 3.22 (m, 2H), 2.89 (d, J = 13.3 Hz, 4H), 2.53 (s, 3H), 2.46 (s, 3H), 2.06 (s, 4H), 1.86 (dd, J = 13.9 Hz, J = 6.8 Hz, 2H), 1.64 (m, 2H), 1.44 (s, 6H); ^{13}C NMR (100 MHz, $CDCl_3$) δ 166.6, 163.4, 160.4, 158.7, 158.0, 156.3, 149.1, 143.0, 138.4, 133.67, 133.64 (d, $^{3}J_{C,F}$ = 3 Hz), 133.2, 132.3, 124.6, 121.43, 121.35 (d, $^{2}J_{C,F}$ = 8.3 Hz), 117.5, 115.81, 115.59 (d, $^{1}J_{C,F}$ = 22.8 Hz), 110.7, 109.6, 86.4, 59.1, 55.8, 50.6, 46.0, 43.3, 43.1, 40.7, 29.8, 29.5, 28.7, 25.7, 19.3, 18.0, 14.3, 12.6; HRMS (ESI) *m/z* calcd for $C_{36}H_{46}FN_8O_7S$+ $[M+H]^+$: 753.3189, Found: 753.3148.

Crude LC-MS data

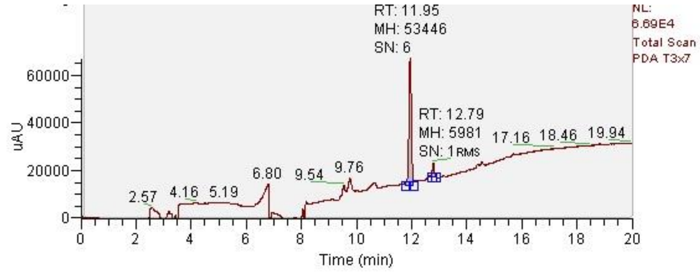

Mass spectrum

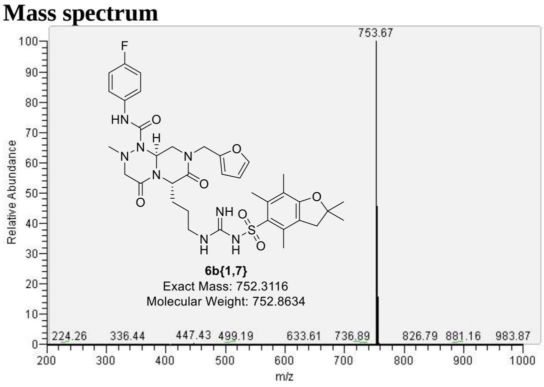

Crude ^{1}H NMR

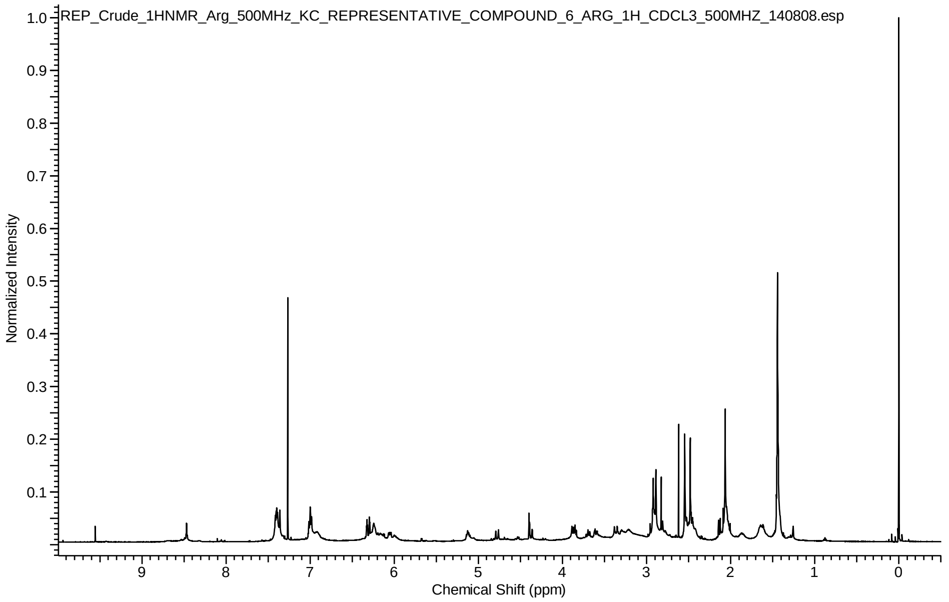

Chiral HPLC

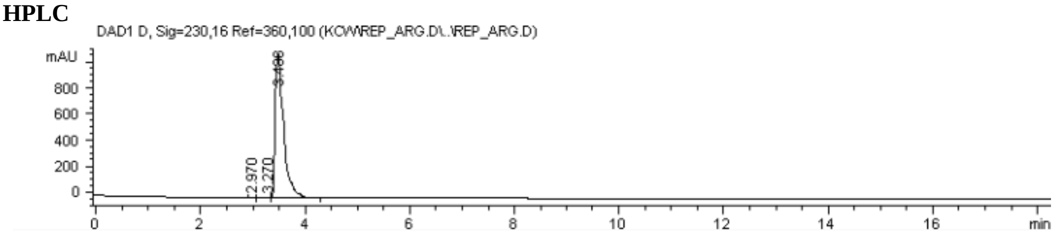

gCOSY

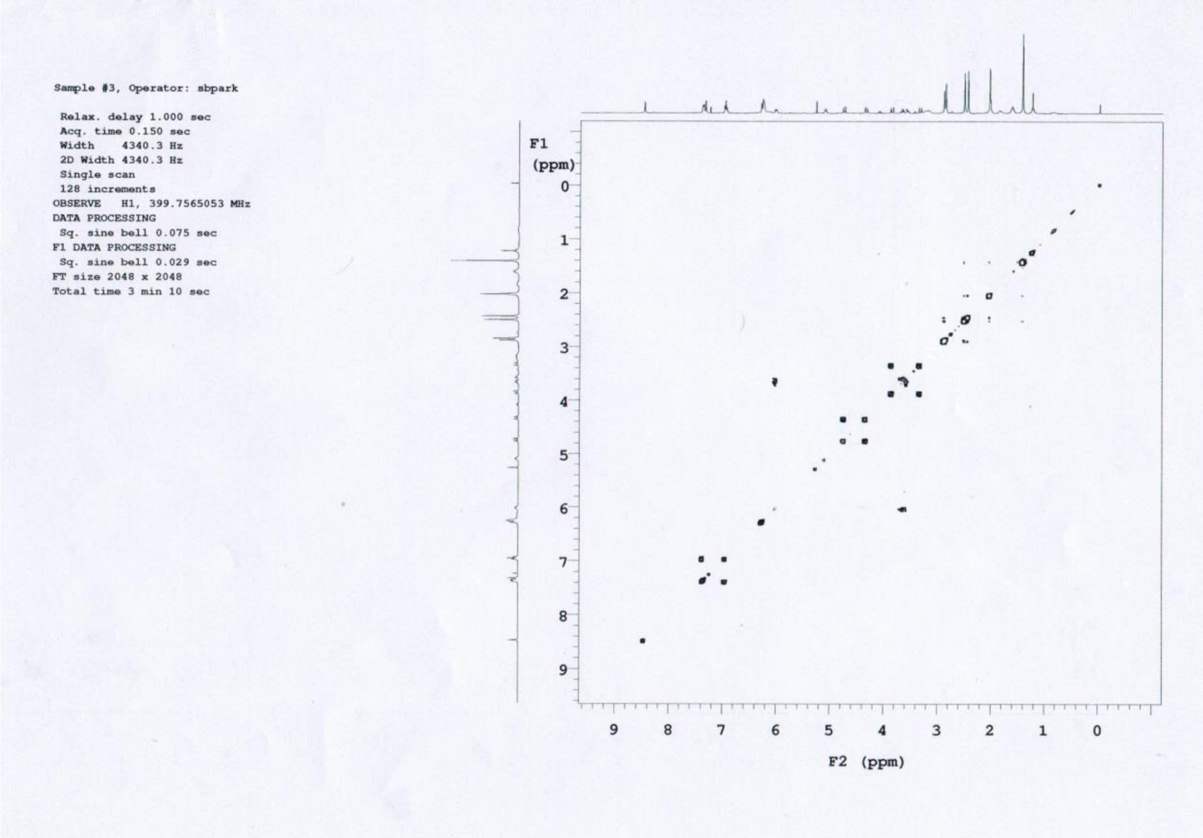

7. Representative compound 13 (6d{3,2})

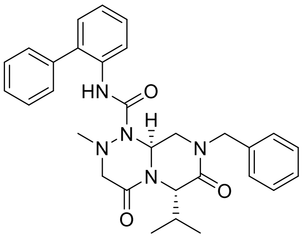

Representative Compound 13 (6d{,3,2}): ^{1}H NMR (400 MHz, $CDCl_3$) δ 8.71 (s, 1H), 8.11 (d, J = 8.2 Hz, 1H), 7.35 (m, 11H), 7.15 (t, J = 8 Hz, 2H), 6.11 (dd, J = 9.4 Hz, J = 5.1 Hz, 1H), 5.05 (d, J = 7 Hz, 1H), 4.63 (d, J = 14.5 Hz, 1H), 4.55 (d, J = 14.5 Hz, 1H), 3.49 (m, 2H), 3.43, (m, 1H), 3.00 (d, J = 16.8 Hz, 1H), 2.45 (s, 3H), 2.35 (m, 1H), 1.15 (d, J = 6.7 Hz, 3H), 1.03 (d, J = 7 Hz, 3H); ^{13}C NMR (100 MHz, $CDCl_3$) δ 166.6, 163.0, 153.3, 138.6, 136.0, 134.9, 132.3, 129.9, 129.4, 129.0, 128.9, 128.7, 128.4, 128.04, 128.00, 123.8, 119.8, 59.7, 55.3, 50.5, 50.2, 45.5, 32.2, 20.1, 20.0; LRMS (ESI) *m/z* calcd for $C_{30}H_{34}N_5O_3$+ $[M+H]^+$: 512.26, Found: 512.29.

Crude LC-MS data

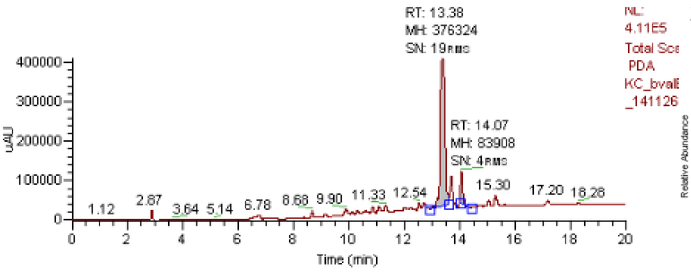

Mass spectrum

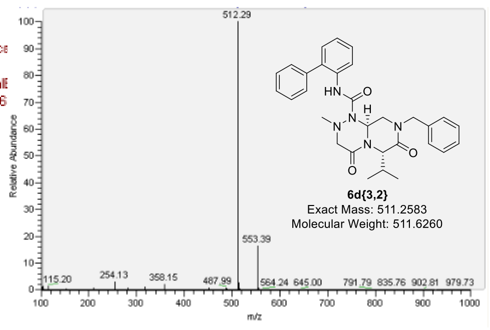

Crude ^{1}H NMR

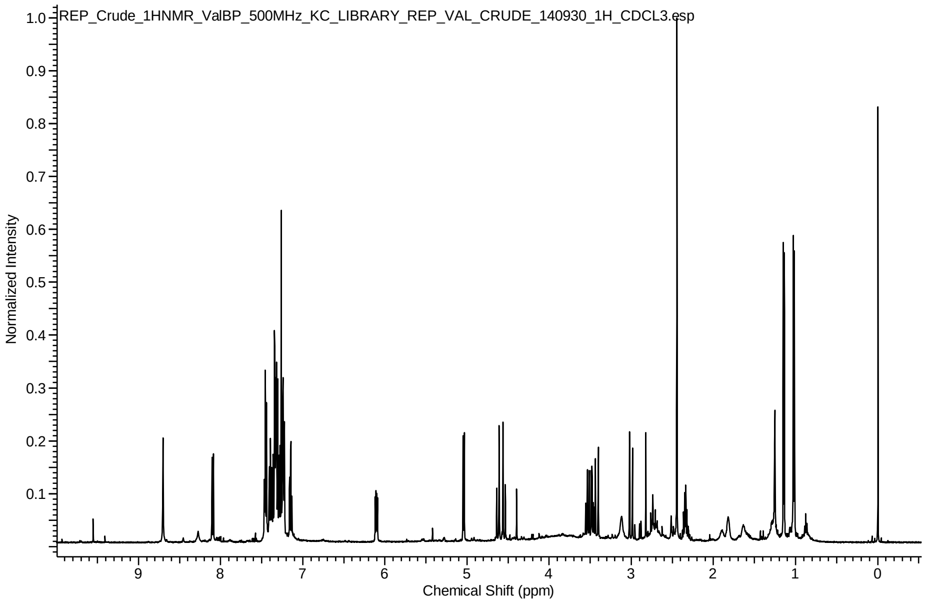

Chiral HPLC

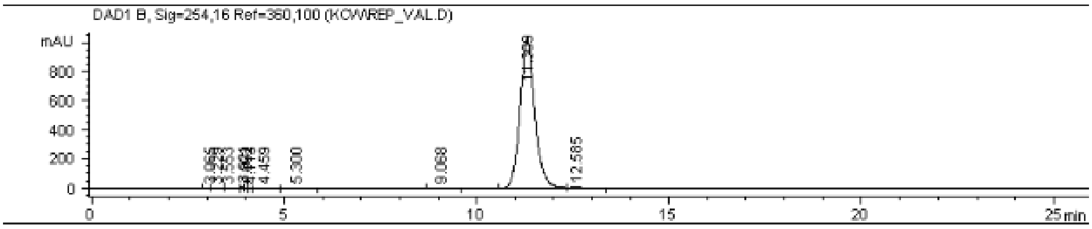

gCOSY

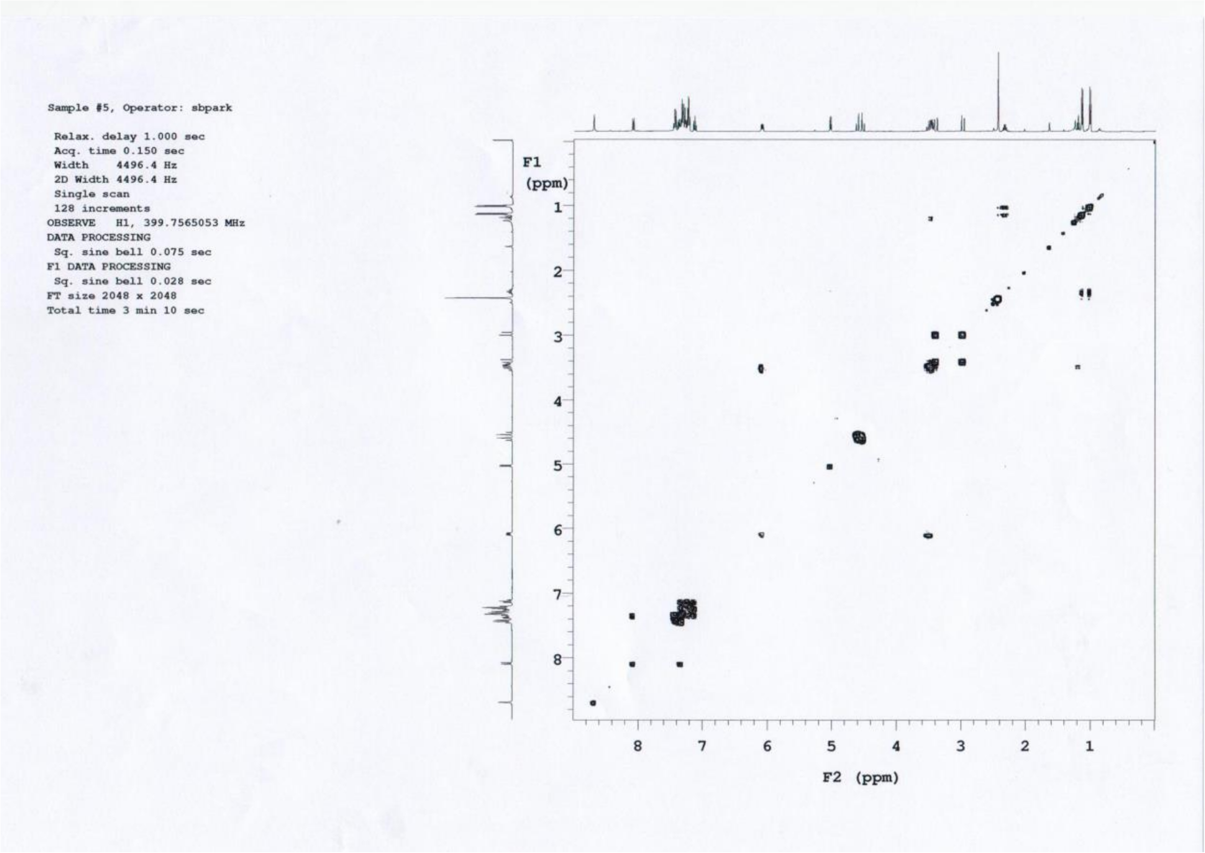

8. Representative compound 14 (6a{3,5})

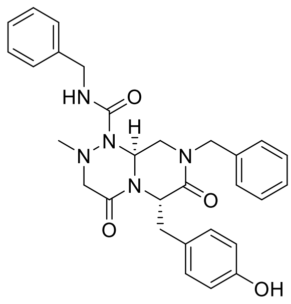

Representative Compound 14 (6a{3,5}): ^{1}H NMR (400 MHz, $CDCl_3$) δ 7.33 (m, 6H), 7.23 (m, 4H), 7.00 (d, *J* = 8.2 Hz, 2H), 6.73 (t, *J* = 5.9 Hz, 1H), 6.64 (d, *J* = 8.2 Hz, 2H), 5.49 (dd, *J* = 10.6 Hz, *J* = 4.3 Hz, 1H), 5.36 (t, *J* = 5.7 Hz, 1H), 4.87 (d, *J* = 14.5 Hz, 1H), 4.45 (m, 2H), 4.3 (m, 1H), 3.49 (m, 2H), 3.37 (d, *J* = 5.5 Hz, 2H), 3.31 (dd, *J* = 11.7 Hz, *J* = 4.3 Hz, 1H), 3.16 (d, *J* = 16.8 Hz, 1H), 2.68 (s, 3H); ^{13}C NMR (100 MHz, $CDCl_3$) δ 166.4, 162.7, 156.2, 155.7, 138.7, 135.7, 130.7, 129.0, 128.2, 127.7, 127.0, 115.8, 80.4, 56.9, 55.5, 50.7, 50.6, 45.7, 44.3, 35.9; LRMS (ESI) *m/z* calcd for $C_{29}H_{32}N_5O_3$+ $[M+H]^+$: 514.24, Found: 514.06.

Crude LC-MS data

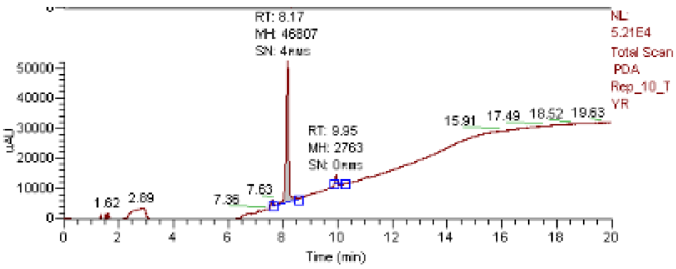

Mass spectrum

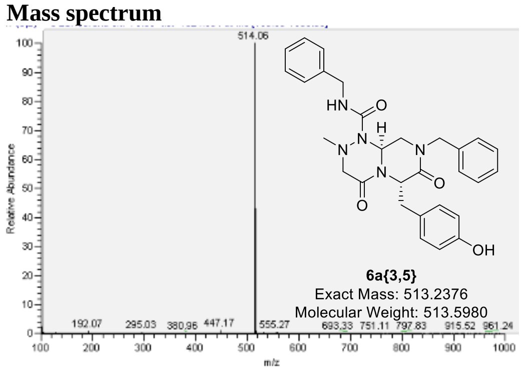

Crude ^{1}H NMR

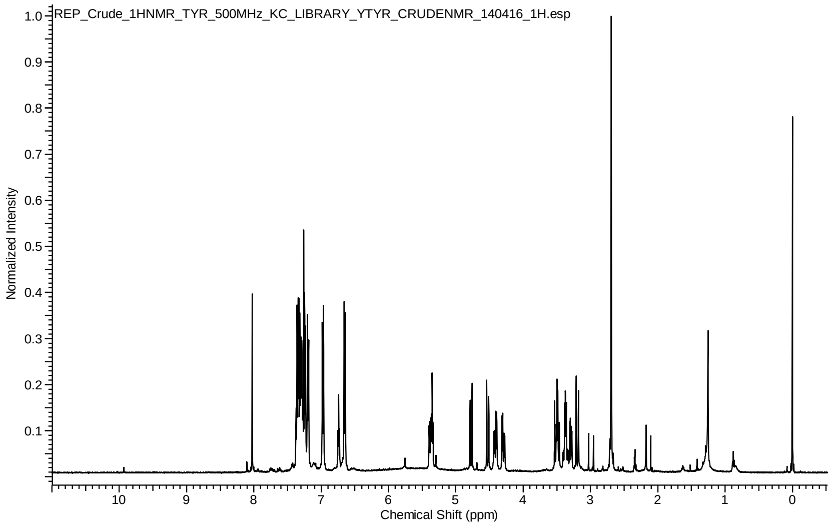

Chrial HPLC

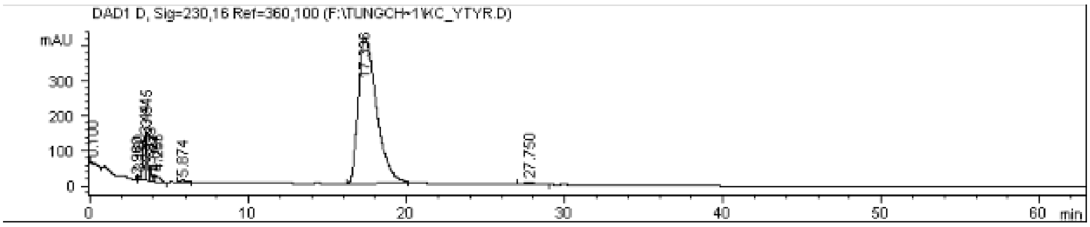

gCOSY

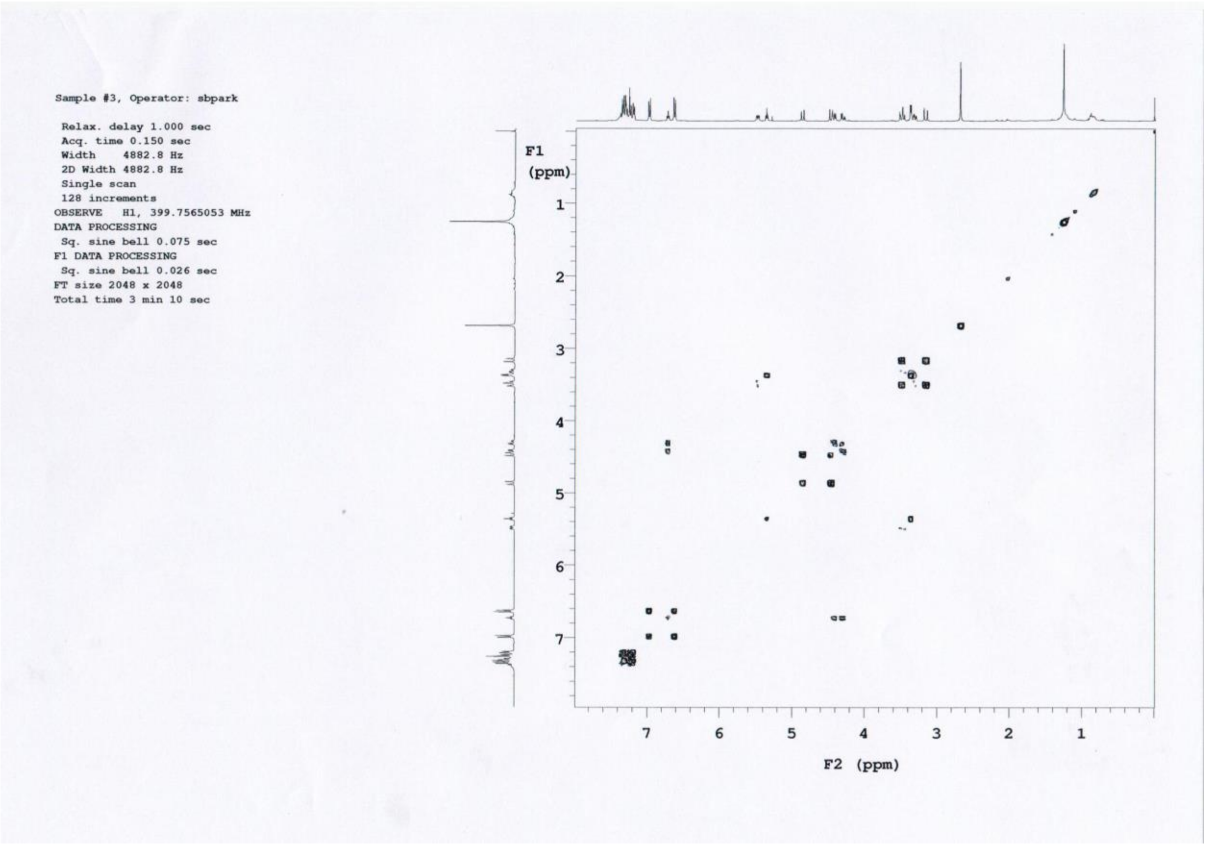

IV. Principal Component Analysis (PCA) of pyrazinotriazinedione library

PCA was performed against 162 independent molecules using 8 major molecular descriptors [molecular weight, topological PSA, 2D VDW volume, 2D VSA hydrophobic surface, 2D VSA polar surface, 2D VSA Hbond donors, 2D VSA Hbond acceptors, AlogP98 value]. Molecular descriptors were calculated using PreADMET 2.0 software [BMDRC, Seoul, Korea] and PCA was executed using SAS 9.3 software [SAS Institute Inc., Cary, NC, USA]. Three principal components (Prin1, Prin2, and Prin3) represent 99.6% of the total variance in molecular descriptors. Prin1 factor, which explains 95.9% of the total variance, is mainly constituted by molecular weight (MW), 2D van der Waals (VDW) volume and AlogP98 value. Prin2 factor, which explains 3.0% of the total variance, is influenced by topological polar surface area (PSA), 2D van der Waals (VDW) polar surface, 2D van der Waals surface area (VSA) Hbond donors and 2D van der Waals surface area (VSA) Hbond acceptors. Prin3 factor, accounting for 0.6% of the total variance, includes topological polar surface area and 2D VSA hydrophobic. The eigenvalues of the covariance matrix and eigenvectors are presented in Table S1 and S2, respectively.

Table S1. Eigenvalues of the covariance matrix

Eigenvalues of the covariance matrix				
	**Eigenvalue**	**Difference**	**Proportion**	**Cumulative**
**Prin 1**	20356.7478	19711.3138	0.9590	0.9590
**Prin 2**	645.4338	514.3846	0.0304	0.9894
**Prin 3**	131.0492	52.9487	0.0062	0.9955

Table S2. Eigenvectors in principal component analysis

Eigenvectors			
	**Prin1**	**Prin2**	**Prin3**
**Molecular_weight**	0.671133	0.158384	-.597872
**Topological_PSA**	0.189649	0.580146	0.494505
**2D_VDW_volume**	0.565977	-.131462	0.056570
**2D_VSA_hydrophobic**	0.403183	-.581978	0.565279
**2D_VSA_polar**	0.143184	0.421638	0.237825

Eigenvectors			
	**Prin1**	**Prin2**	**Prin3**
**2D_VSA_Hbond_donor**	0.076611	0.251406	0.097630
**2D_VSA_Hbond_acceptor**	0.065710	0.201759	0.093448
**AlogP98_value**	0.006236	-.025961	-.021187

Fig S2. (A) 3-D visualization of chemical space of tetra-substituted pyrazinotriazinedione library differentiated by R_1 substituents. (B) 3-D visualization of chemical space differentiated by R_2 substituents (C) 3-D visualization of chemical space differentiated by R_3 substituents (D) 3-D visualization of chemical space differentiated by R_4 substituents [PreADMET, V_{conf} Interface, SAS 9.3, Spotfire Decision site]

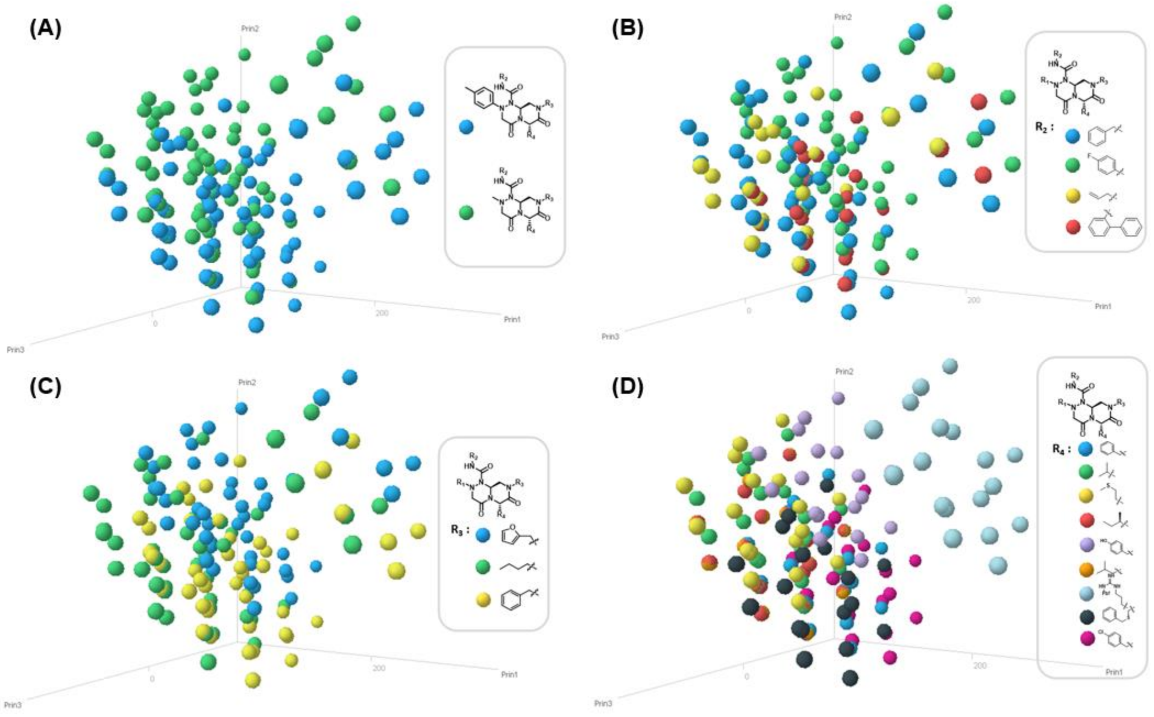

VI. Copies of ^{1}H and ^{13}C NMR spectra

1. Acid coupling partners

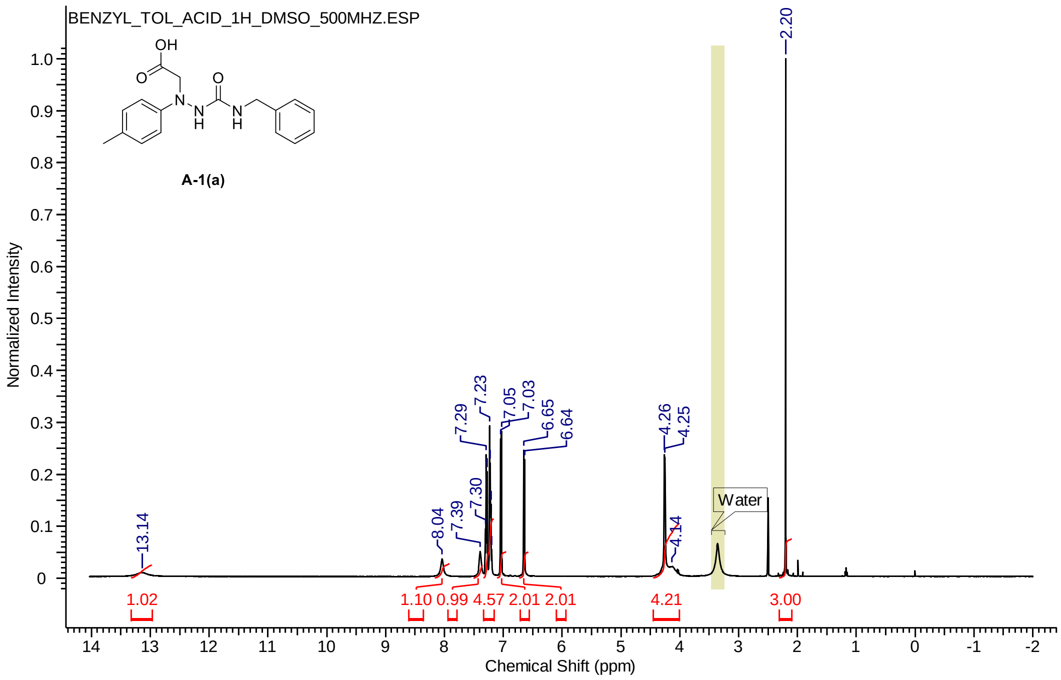

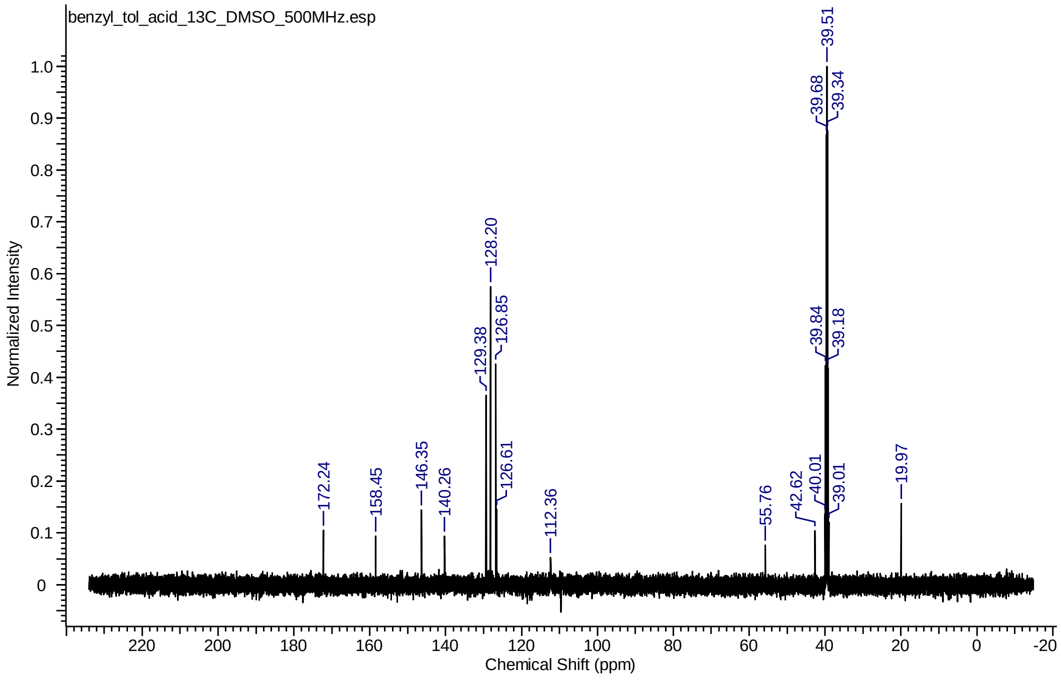

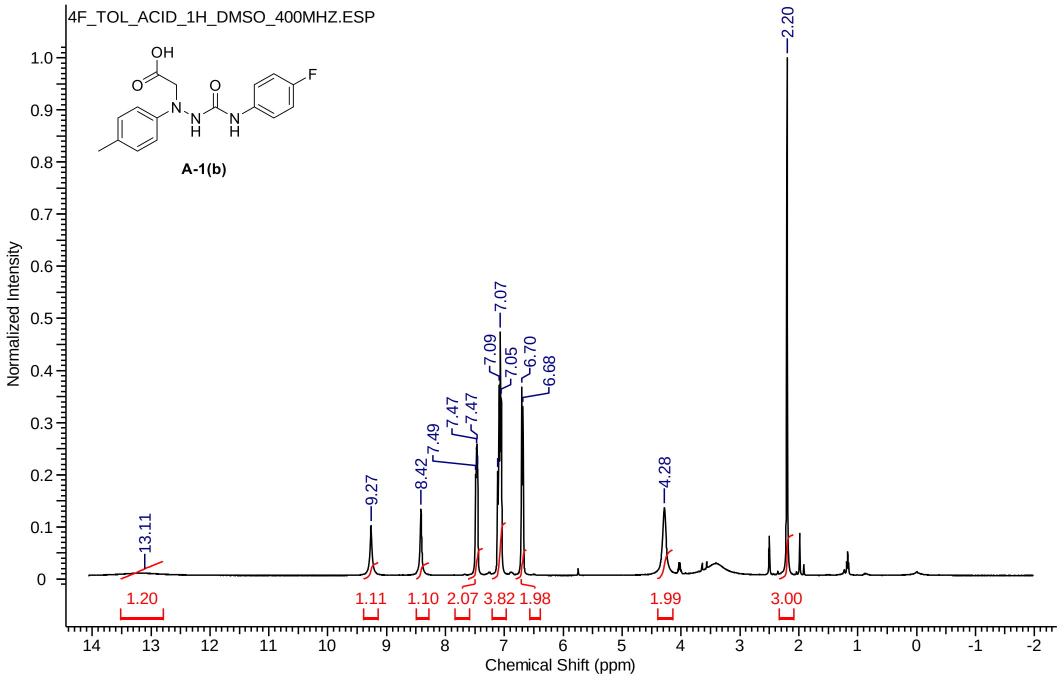
4F_TOL_ACID_1H_DMSO_400MHZ.ESP
A-1(b)
13.11
9.27
8.42
7.49
7.47
7.47
7.09
7.07
7.05
6.70
6.68
4.28
2.20
1.20
1.11
1.10
2.07
3.82
1.98
1.99
3.00
Normalized Intensity
Chemical Shift (ppm)

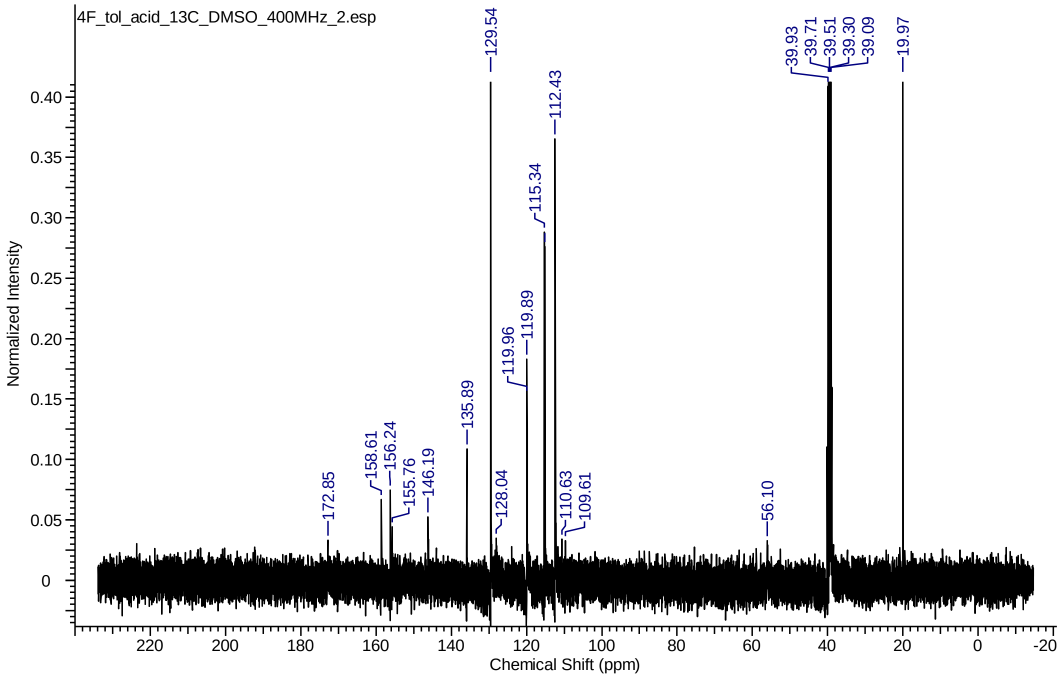
4F_tol_acid_13C_DMSO_400MHz_2.esp
172.85
158.61
156.24
155.76
146.19
135.89
129.54
128.04
119.96
119.89
115.34
112.43
110.63
109.61
56.10
39.93
39.71
39.51
39.30
39.09
19.97
Normalized Intensity
Chemical Shift (ppm)

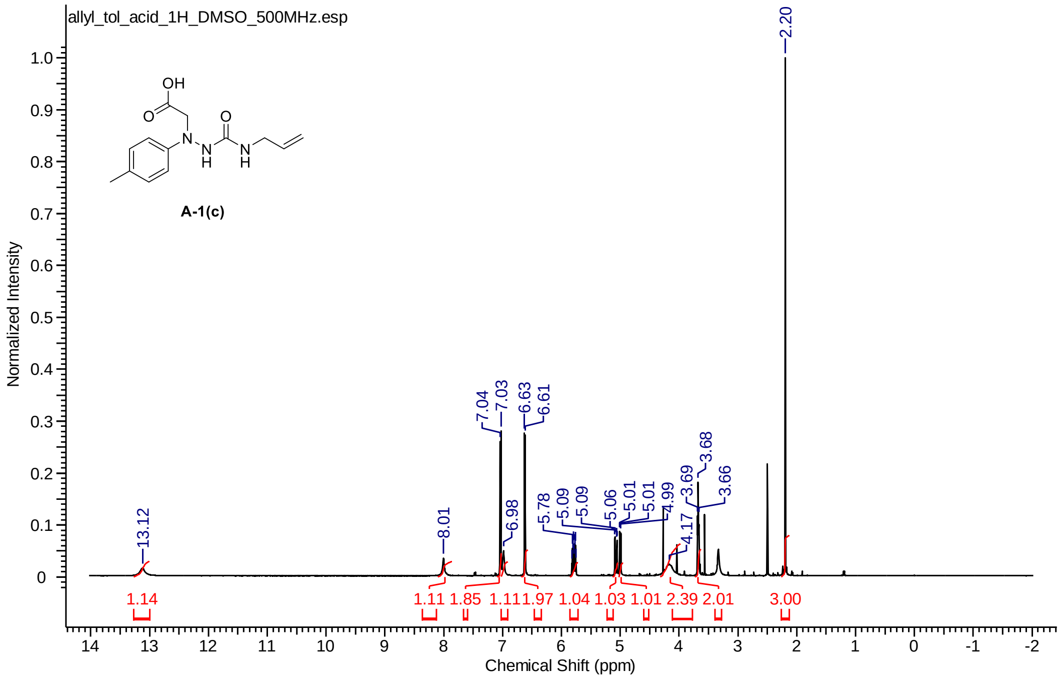
allyl_tol_acid_1H_DMSO_500MHz.esp
A-1(c)
Normalized Intensity
Chemical Shift (ppm)
13.12
8.01
7.04
7.03
6.98
6.63
6.61
5.78
5.09
5.09
5.06
5.01
5.01
4.99
4.17
3.69
3.68
3.66
2.20
1.14
1.11
1.85
1.11
1.97
1.04
1.03
1.01
2.39
2.01
3.00

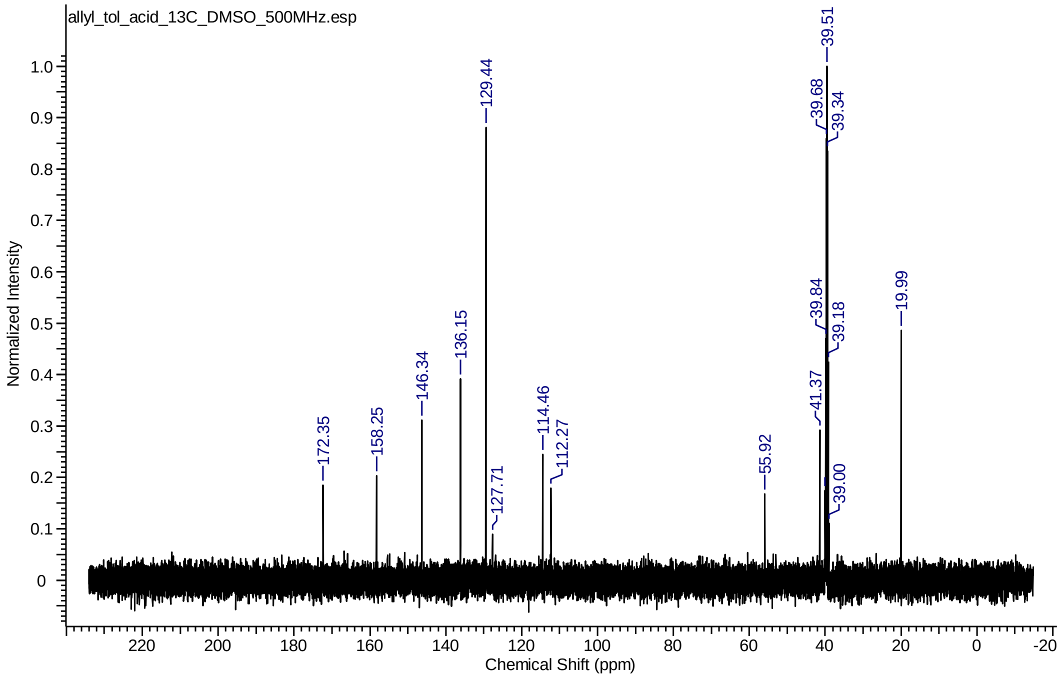
allyl_tol_acid_13C_DMSO_500MHz.esp
Normalized Intensity
Chemical Shift (ppm)
172.35
158.25
146.34
136.15
129.44
127.71
114.46
112.27
55.92
41.37
39.84
39.68
39.51
39.34
39.18
39.00
19.99

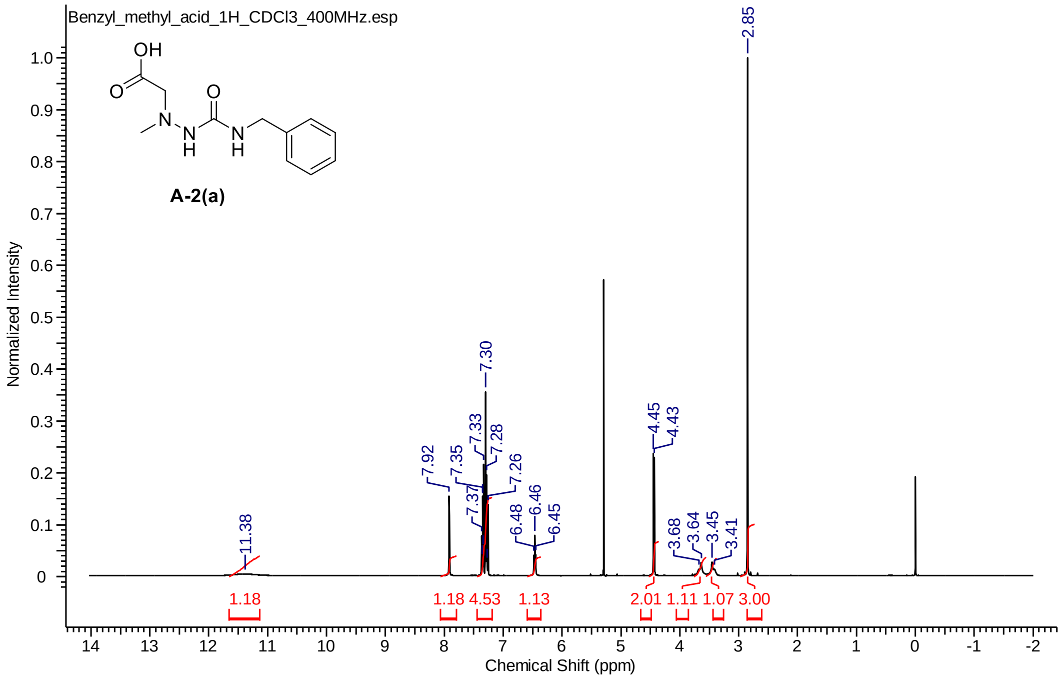
Benzyl_methyl_acid_1H_CDCl3_400MHz.esp
A-2(a)
Normalized Intensity
Chemical Shift (ppm)
11.38
7.92
7.35
7.37
7.33
7.30
7.28
7.26
6.48
6.46
6.45
4.45
4.43
3.68
3.64
3.45
3.41
2.85
1.18 1.18 4.53 1.13 2.01 1.11 1.07 3.00

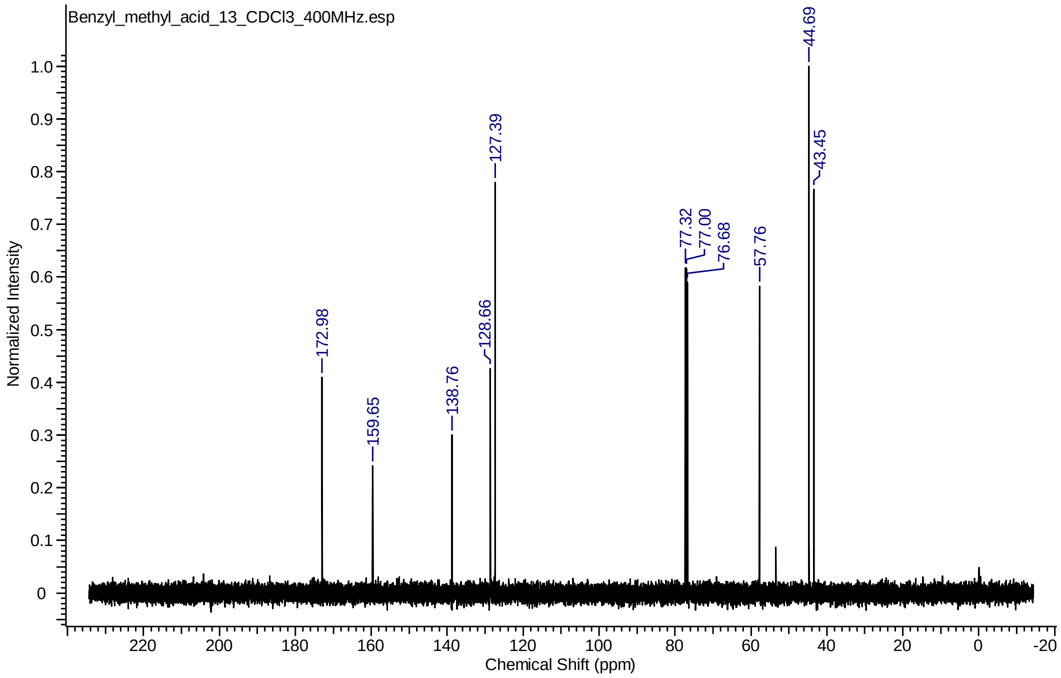
Benzyl_methyl_acid_13_CDCl3_400MHz.esp
Normalized Intensity
Chemical Shift (ppm)
172.98
159.65
138.76
128.66
127.39
77.32
77.00
76.68
57.76
44.69
43.45

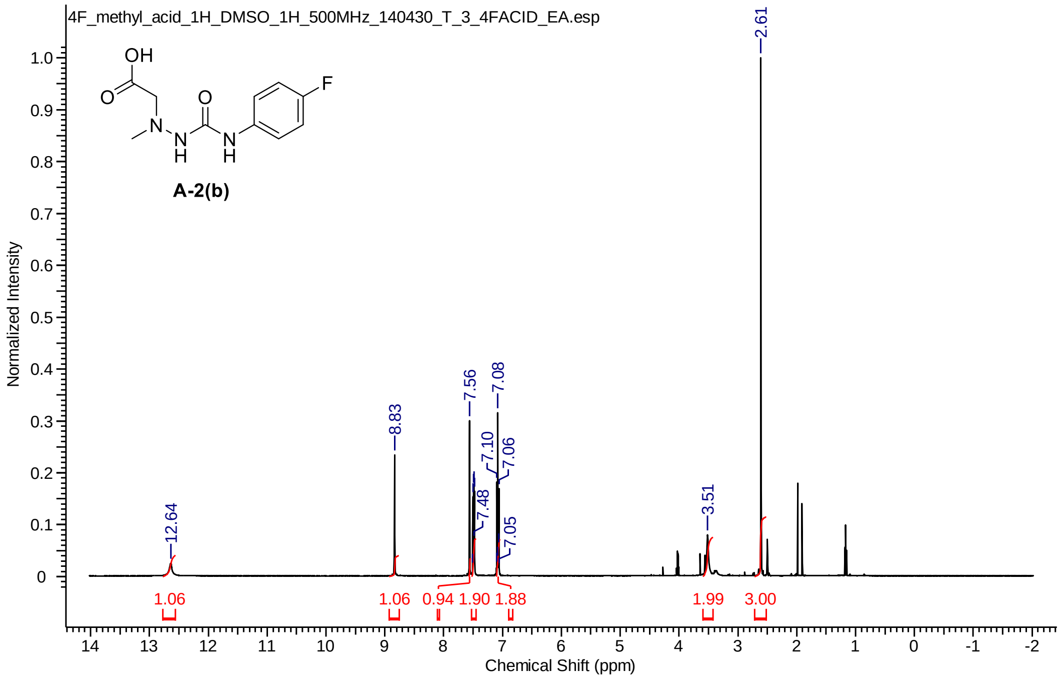
4F_methyl_acid_1H_DMSO_1H_500MHz_140430_T_3_4FACID_EA.esp
A-2(b)
Normalized Intensity
Chemical Shift (ppm)
2.61
8.83
7.56
7.08
7.10
7.06
7.48
7.05
12.64
3.51
1.06
1.06
0.94
1.90
1.88
1.99
3.00

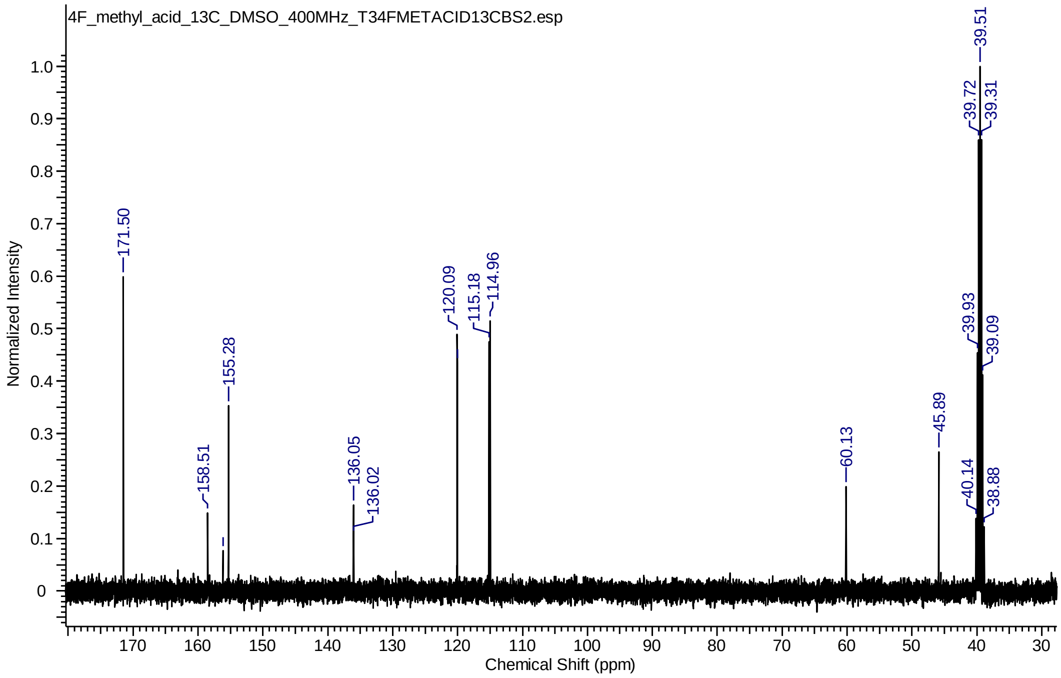
4F_methyl_acid_13C_DMSO_400MHz_T34FMETACID13CBS2.esp
Normalized Intensity
Chemical Shift (ppm)
171.50
158.51
155.28
136.05
136.02
120.09
115.18
114.96
60.13
45.89
39.51
39.72
39.31
39.93
39.09
40.14
38.88

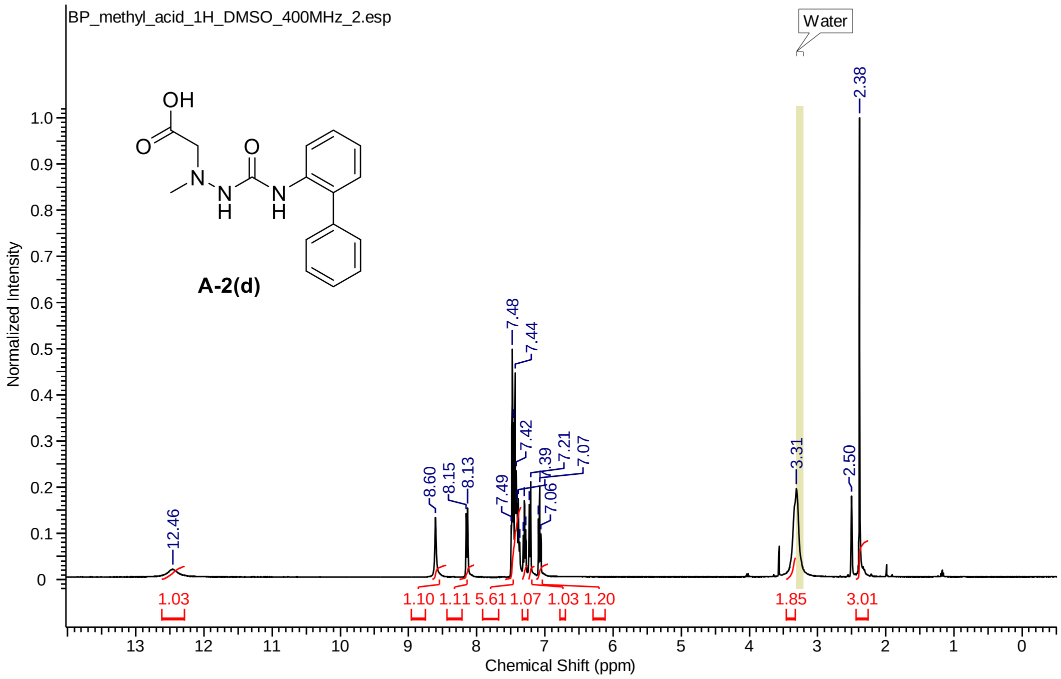
BP_methyl_acid_1H_DMSO_400MHz_2.esp
Water
A-2(d)
OH
O
O
N
N
H
N
H
12.46
8.60
8.15
8.13
7.49
7.48
7.44
7.42
7.39
7.21
7.07
7.06
3.31
2.50
2.38
1.03
1.10
1.11
5.61
1.07
1.03
1.20
1.85
3.01
Normalized Intensity
Chemical Shift (ppm)

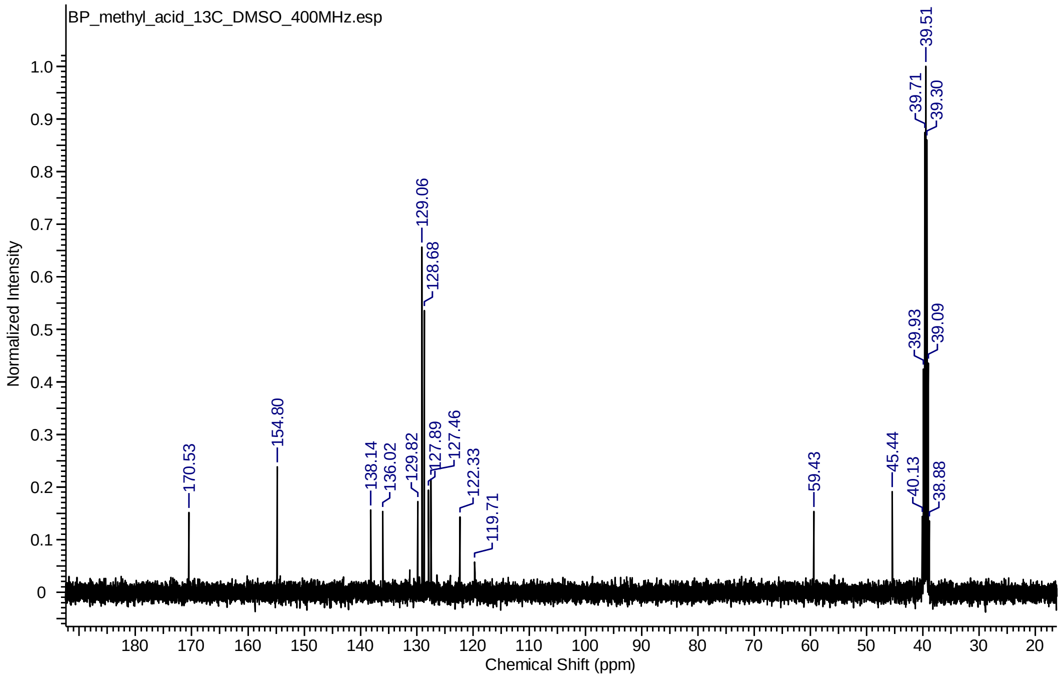
BP_methyl_acid_13C_DMSO_400MHz.esp
170.53
154.80
138.14
136.02
129.82
129.06
128.68
127.89
127.46
122.33
119.71
59.43
45.44
40.13
39.93
39.71
39.51
39.30
39.09
38.88
Normalized Intensity
Chemical Shift (ppm)

2. Representative compounds

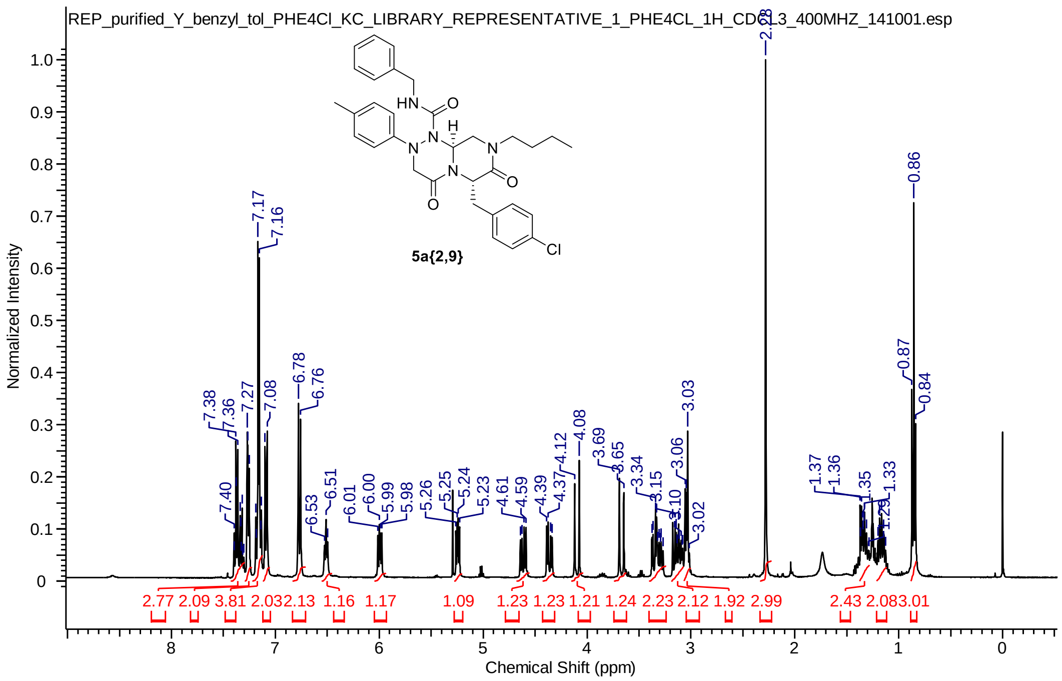

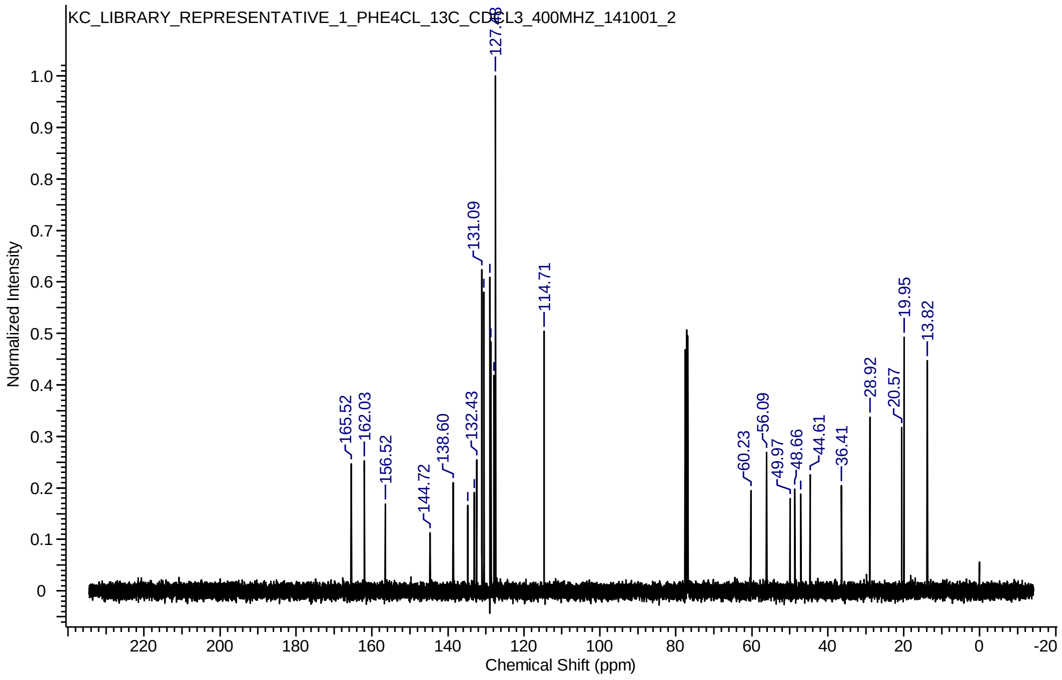

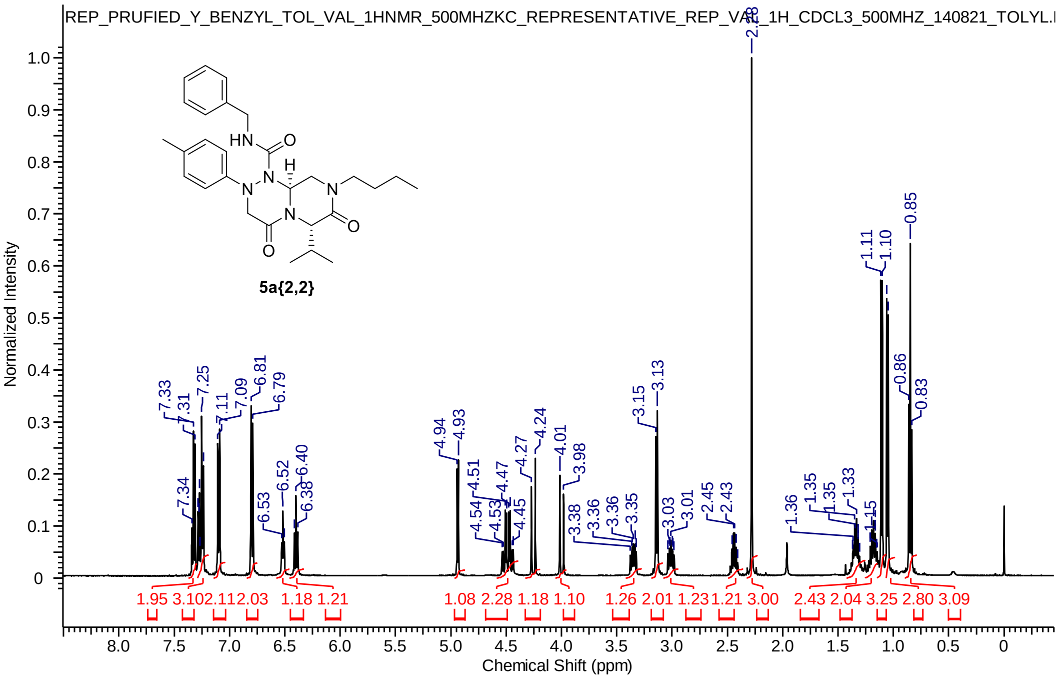
REP_PRUFIED_Y_BENZYL_TOL_VAL_1HNMR_500MHZKC_REPRESENTATIVE_REP_VAL_1H_CDCL3_500MHZ_140821_TOLYL.I
Normalized Intensity
Chemical Shift (ppm)
HN
O
N
N
H
N
N
O
N
O
5a{2,2}
1.95 3.10 2.11 2.03 1.18 1.21 1.08 2.28 1.18 1.10 1.26 2.01 1.23 1.21 3.00 2.43 2.04 3.25 2.80 3.09

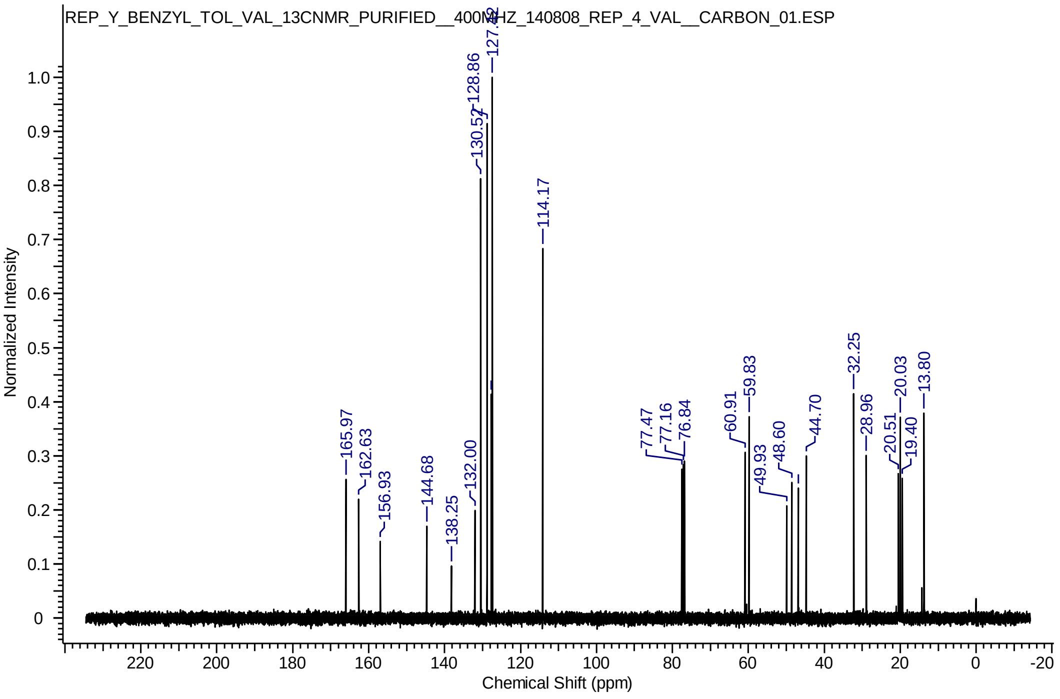
REP_Y_BENZYL_TOL_VAL_13CNMR_PURIFIED__400MHZ_140808_REP_4_VAL__CARBON_01.ESP
Normalized Intensity
Chemical Shift (ppm)
165.97
162.63
156.93
144.68
138.25
132.00
130.52
128.86
127.42
114.17
77.47
77.16
76.84
60.91
59.83
49.93
48.60
44.70
32.25
28.96
20.51
20.03
19.40
13.80

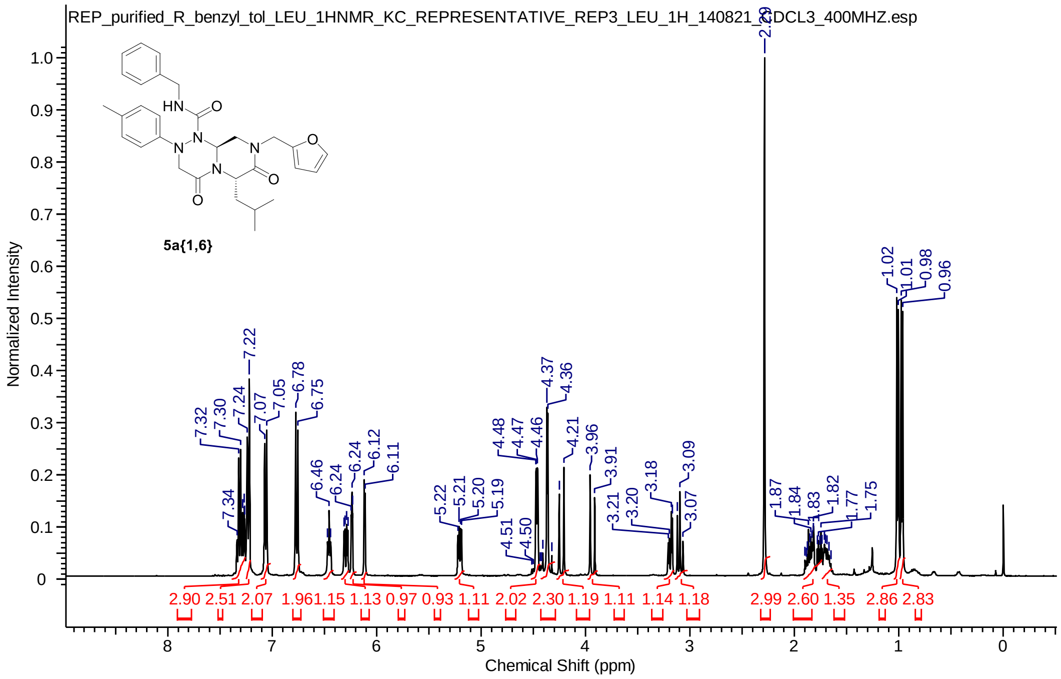
REP_purified_R_benzyl_tol_LEU_1HNMR_KC_REPRESENTATIVE_REP3_LEU_1H_140821_CDCL3_400MHZ.esp
5a{1,6}
HN
O
N
N
N
N
O
O
O
Normalized Intensity
Chemical Shift (ppm)
7.34 7.32 7.30 7.24 7.22 7.07 7.05 6.78 6.75 6.46 6.24 6.24 6.12 6.11 5.22 5.21 5.20 5.19 4.51 4.50 4.48 4.47 4.46 4.37 4.36 4.21 3.96 3.91 3.21 3.20 3.18 3.09 3.07 2.29 1.87 1.84 1.83 1.82 1.77 1.75 1.02 1.01 0.98 0.96
2.90 2.51 2.07 1.96 1.15 1.13 0.97 0.93 1.11 2.02 2.30 1.19 1.11 1.14 1.18 2.99 2.60 1.35 2.86 2.83

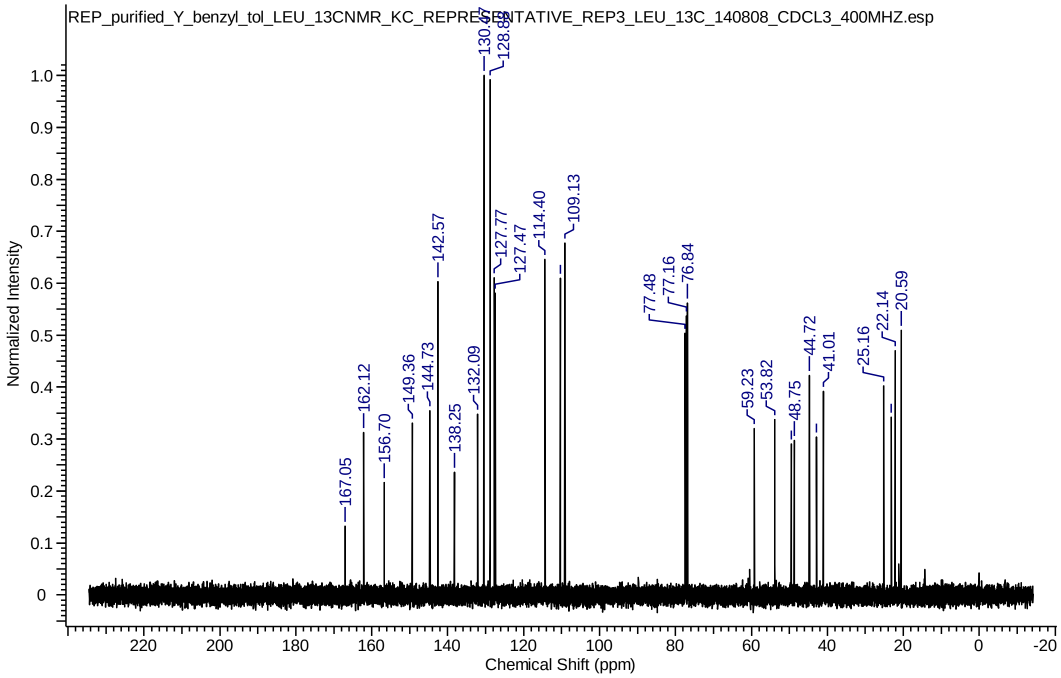
REP_purified_Y_benzyl_tol_LEU_13CNMR_KC_REPRESENTATIVE_REP3_LEU_13C_140808_CDCL3_400MHZ.esp
Normalized Intensity
Chemical Shift (ppm)
167.05 162.12 156.70 149.36 144.73 142.57 138.25 132.09 130.47 128.89 127.77 127.47 114.40 109.13 77.48 77.16 76.84 59.23 53.82 48.75 44.72 41.01 25.16 22.14 20.59

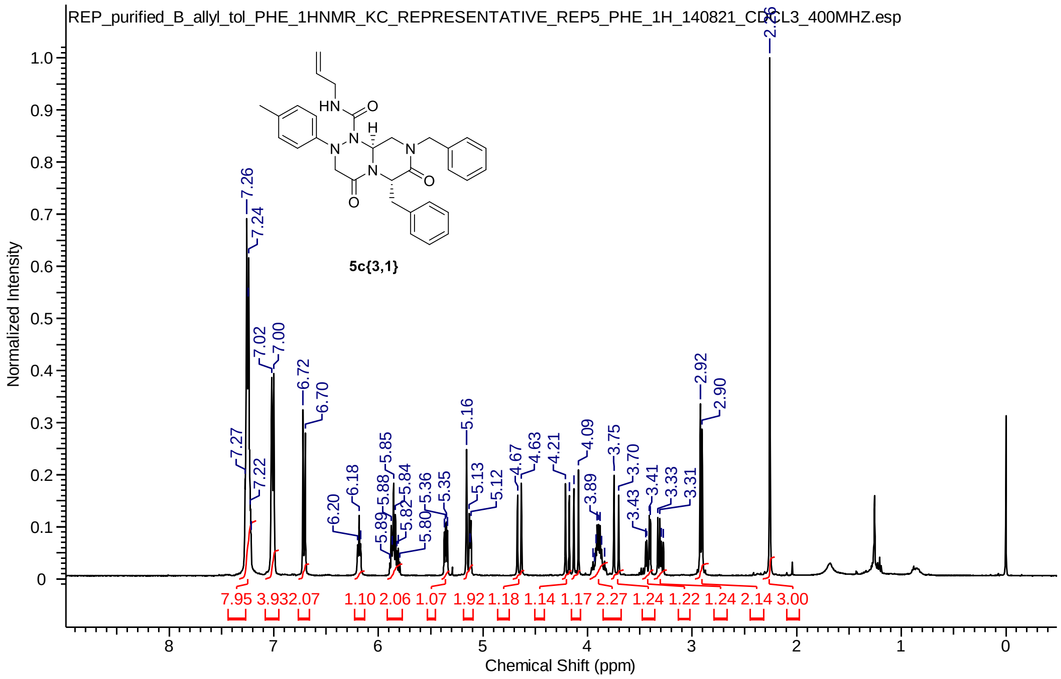
REP_purified_B_allyl_tol_PHE_1HNMR_KC_REPRESENTATIVE_REP5_PHE_1H_140821_CDCL3_400MHZ.esp
5c{3,1}
Normalized Intensity
Chemical Shift (ppm)

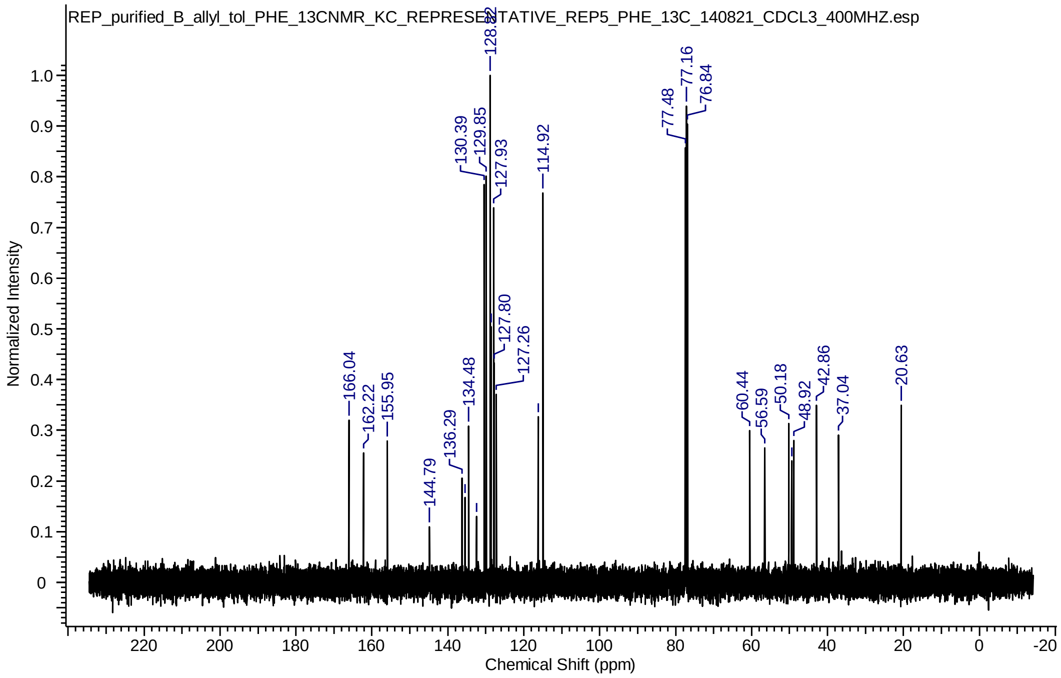
REP_purified_B_allyl_tol_PHE_13CNMR_KC_REPRESENTATIVE_REP5_PHE_13C_140821_CDCL3_400MHZ.esp
Normalized Intensity
Chemical Shift (ppm)

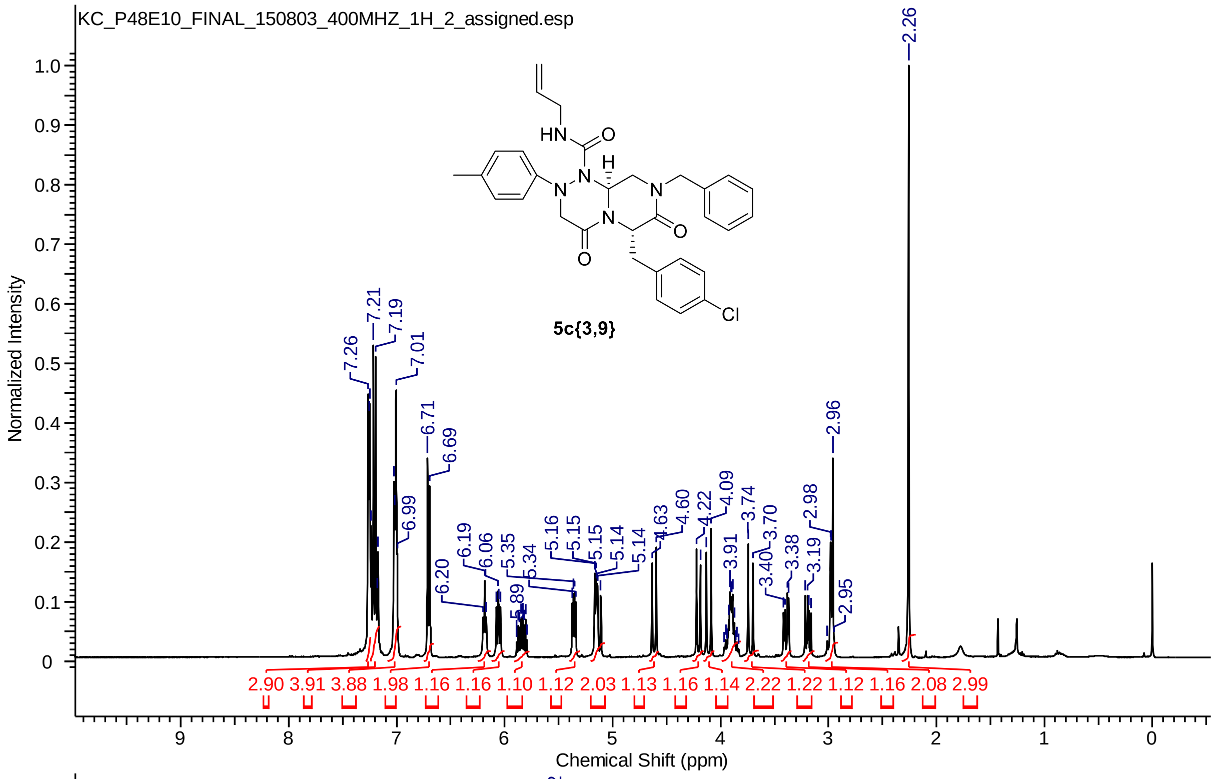
KC_P48E10_FINAL_150803_400MHZ_1H_2_assigned.esp
5c{3,9}
HN
O
N
N
H
N
N
O
O
Cl
7.26 7.21 7.19 7.01 6.99 6.71 6.69 6.20 6.19 6.06 5.89 5.35 5.34 5.16 5.15 5.15 5.14 5.14 4.63 4.60 4.22 4.09 3.91 3.74 3.70 3.40 3.38 3.19 2.98 2.96 2.95 2.26
2.90 3.91 3.88 1.98 1.16 1.16 1.10 1.12 2.03 1.13 1.16 1.14 2.22 1.22 1.12 1.16 2.08 2.99
Normalized Intensity
Chemical Shift (ppm)

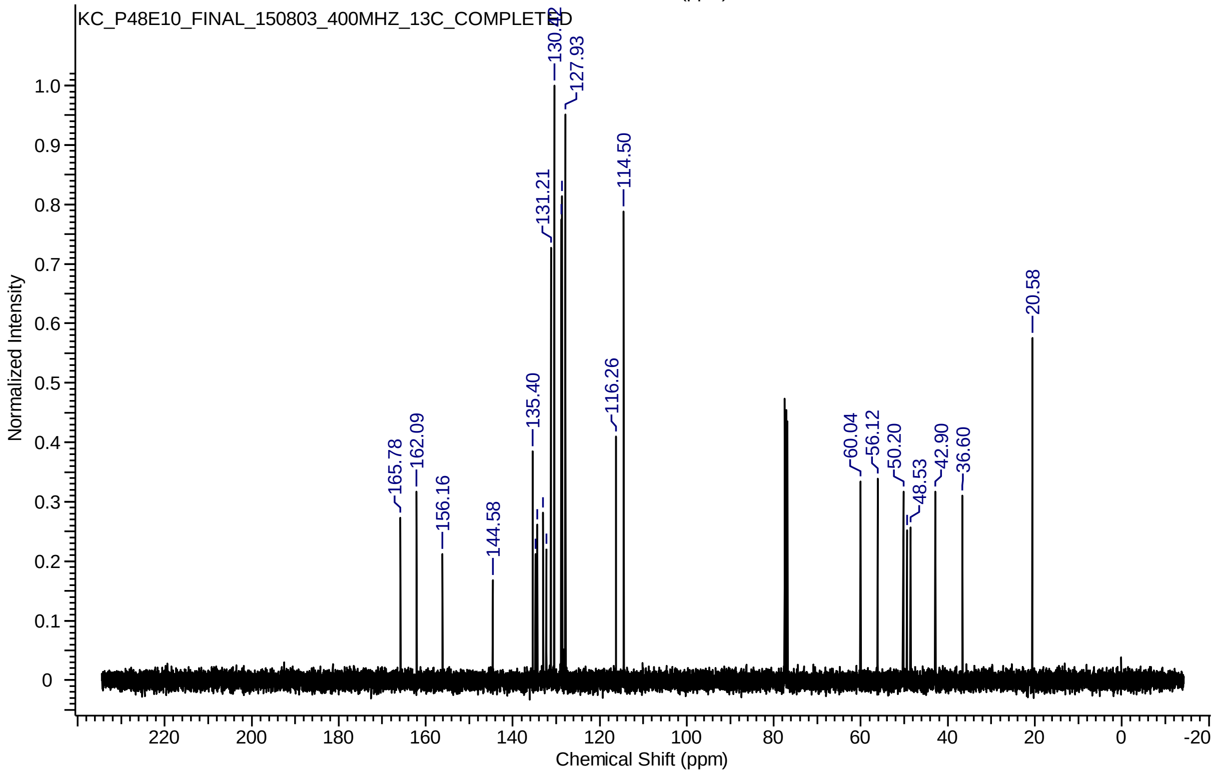
KC_P48E10_FINAL_150803_400MHZ_13C_COMPLETED
165.78 162.09 156.16 144.58 135.40 131.21 130.42 127.93 116.26 114.50 60.04 56.12 50.20 48.53 42.90 36.60 20.58
Normalized Intensity
Chemical Shift (ppm)

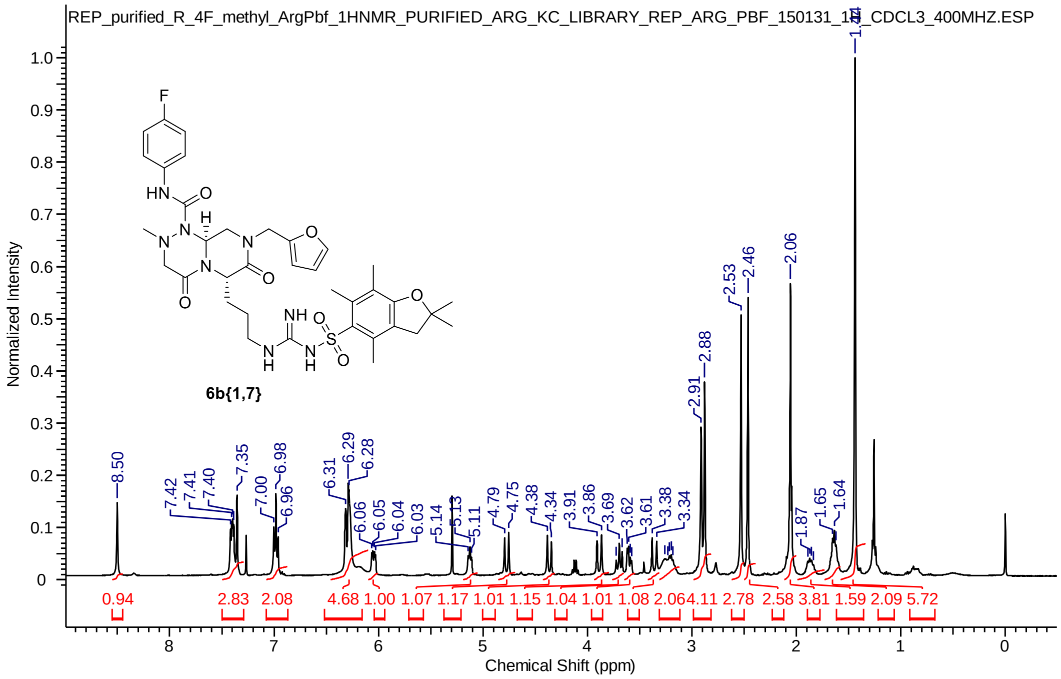
REP_purified_R_4F_methyl_ArgPbf_1HNMR_PURIFIED_ARG_KC_LIBRARY_REP_ARG_PBF_150131
CDCL3_400MHZ.ESP
Normalized Intensity
Chemical Shift (ppm)
6b{1,7}
8.50
7.42
7.41
7.40
7.35
7.00
6.98
6.96
6.31
6.29
6.28
6.06
6.05
6.04
6.03
5.14
5.13
5.11
4.79
4.75
4.38
4.34
3.91
3.86
3.69
3.62
3.61
3.38
3.34
2.91
2.88
2.53
2.46
2.06
1.87
1.65
1.64
0.94
2.83
2.08
4.68
1.00
1.07
1.17
1.01
1.15
1.04
1.01
1.08
2.06
4.11
2.78
2.58
3.81
1.59
2.09
5.72

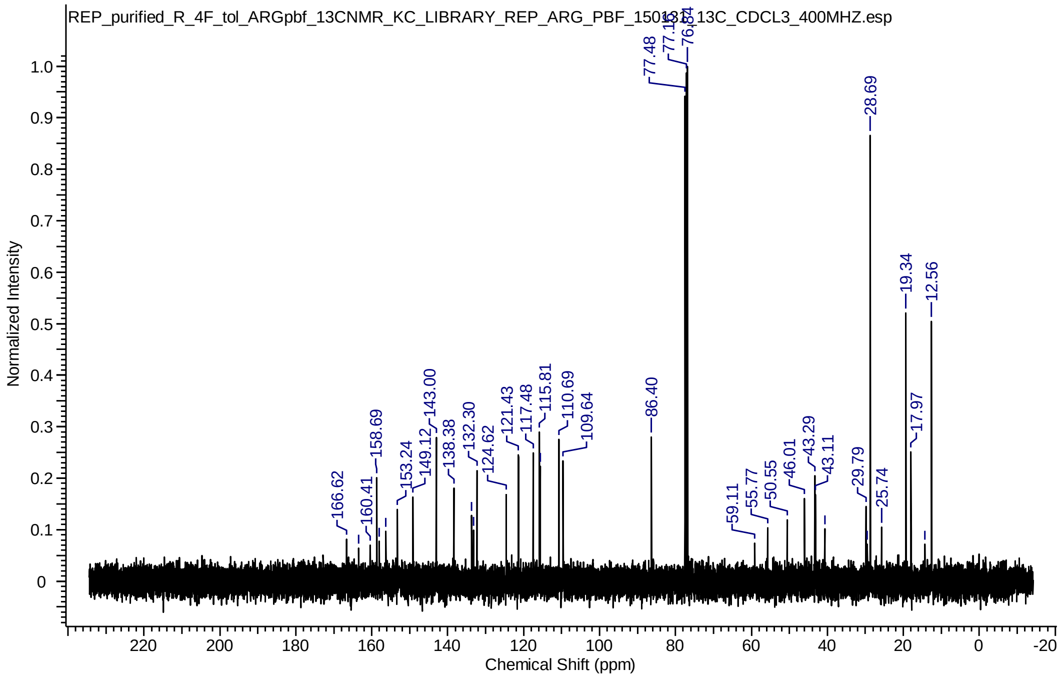
REP_purified_R_4F_tol_ARGpbf_13CNMR_KC_LIBRARY_REP_ARG_PBF_15013
13C_CDCL3_400MHZ.esp
Normalized Intensity
Chemical Shift (ppm)
77.48
86.40
28.69
19.34
12.56
17.97
166.62
160.41
158.69
153.24
149.12
143.00
138.38
132.30
124.62
121.43
117.48
115.81
110.69
109.64
59.11
55.77
50.55
46.01
43.29
43.11
29.79
25.74

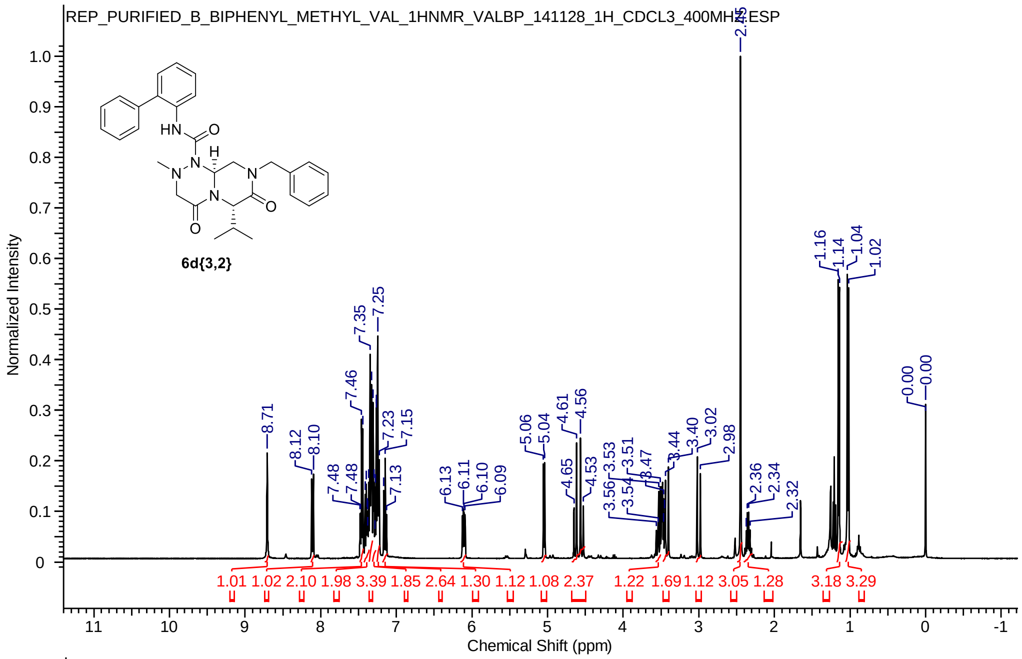
REP_PURIFIED_B_BIPHENYL_METHYL_VAL_1HNMR_VALBP_141128_1H_CDCL3_400MHZ.ESP
6d{3,2}
Normalized Intensity
Chemical Shift (ppm)

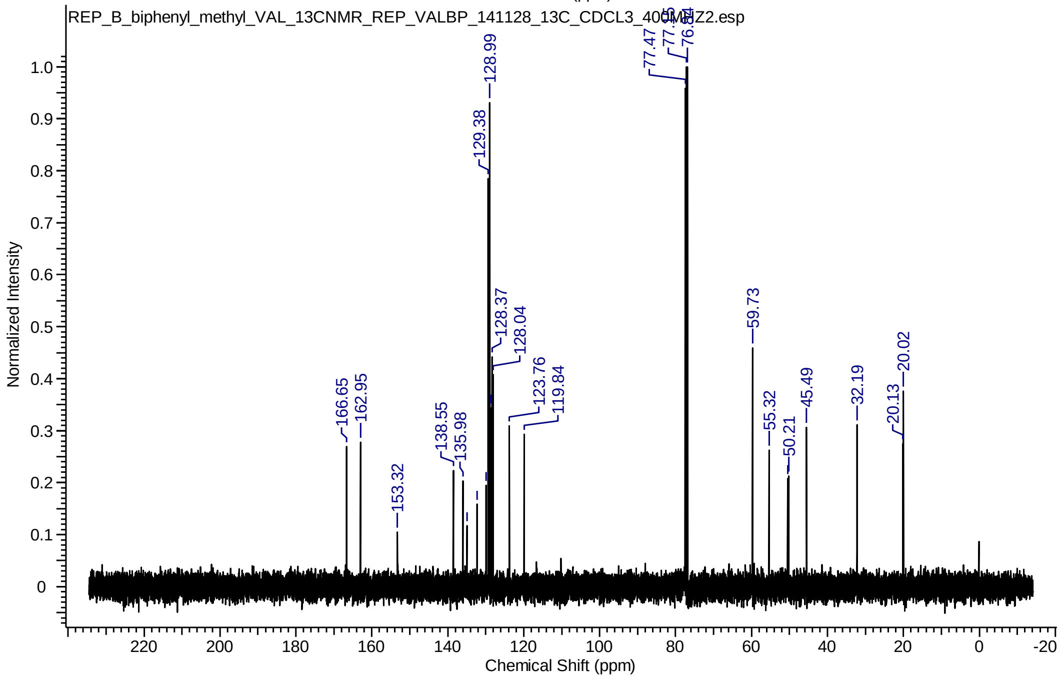
REP_B_biphenyl_methyl_VAL_13CNMR_REP_VALBP_141128_13C_CDCL3_400MHZ2.esp
Normalized Intensity
Chemical Shift (ppm)

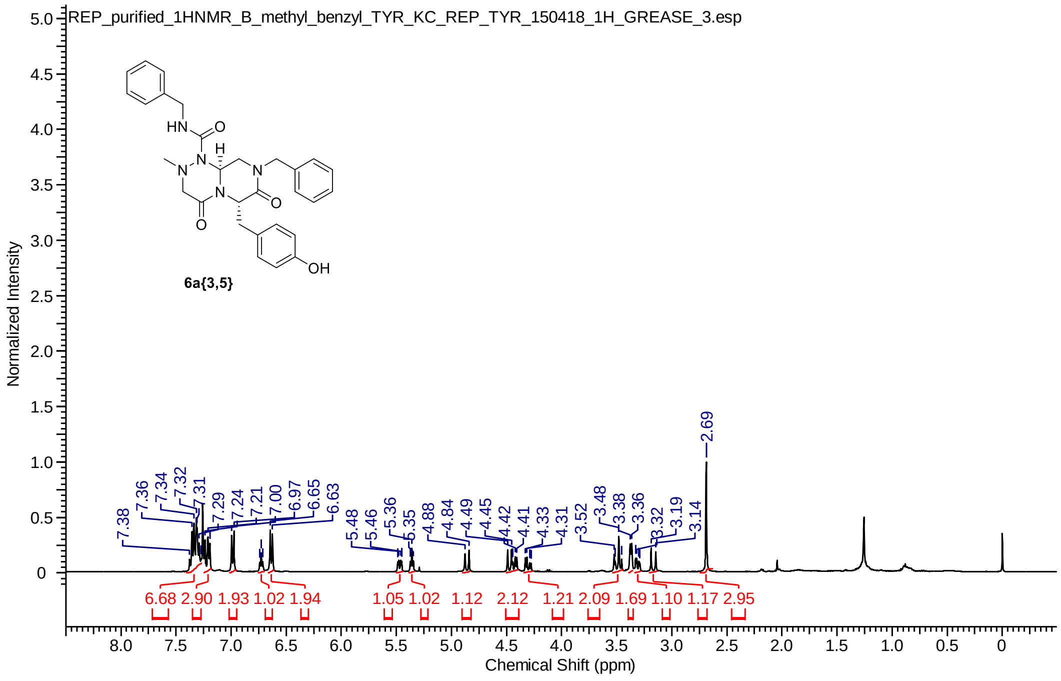
REP_purified_1HNMR_B_methyl_benzyl_TYR_KC_REP_TYR_150418_1H_GREASE_3.esp
6a{3,5}
Normalized Intensity
Chemical Shift (ppm)
7.38 7.36 7.34 7.32 7.31 7.29 7.24 7.21 7.00 6.97 6.65 6.63 5.48 5.46 5.36 5.35 4.88 4.84 4.49 4.45 4.42 4.41 4.33 4.31 3.52 3.48 3.38 3.36 3.32 3.19 3.14 2.69
6.68 2.90 1.93 1.02 1.94 1.05 1.02 1.12 2.12 1.21 2.09 1.69 1.10 1.17 2.95

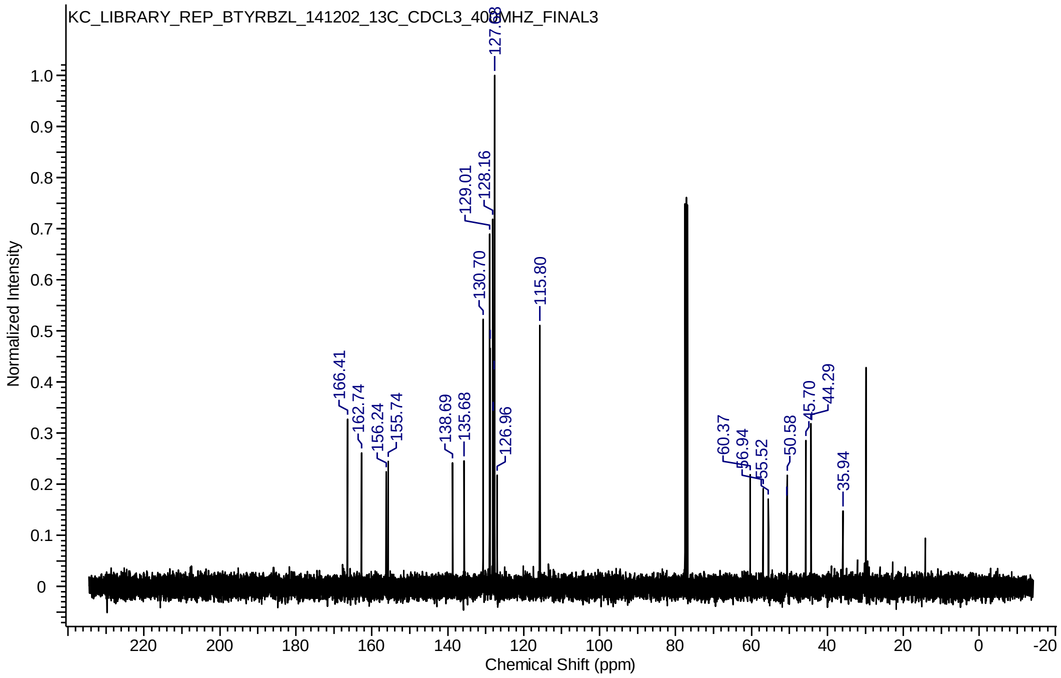
KC_LIBRARY_REP_BTYRBZL_141202_13C_CDCL3_400MHZ_FINAL3
Normalized Intensity
Chemical Shift (ppm)
166.41 162.74 156.24 155.74 138.69 135.68 130.70 129.01 128.16 126.96 115.80 60.37 56.94 55.52 50.58 45.70 44.29 35.94

VII. PDA-based LC/MS analysis data for library compounds

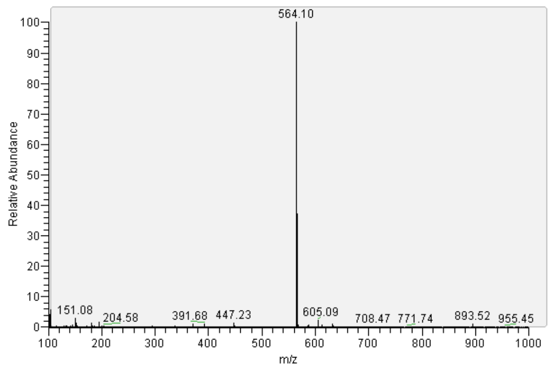

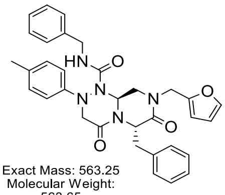

Exact Mass: 563.25
Molecular Weight:
563.65

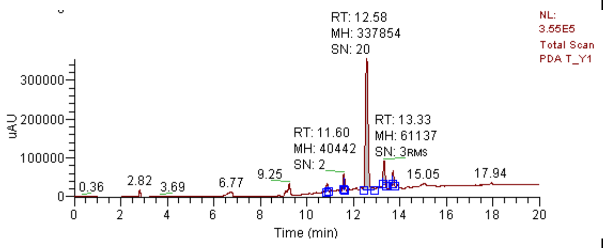

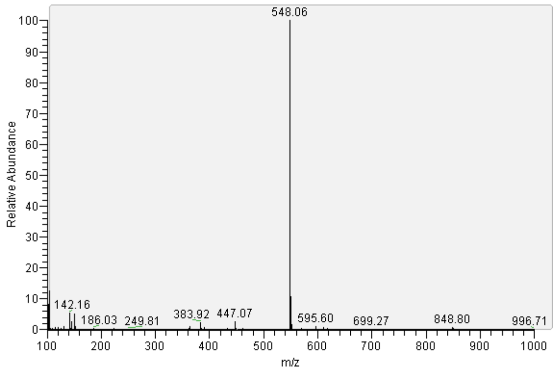

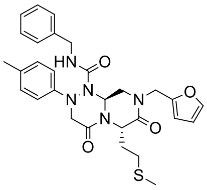

Exact Mass: 547.23
Molecular Weight: 547.67

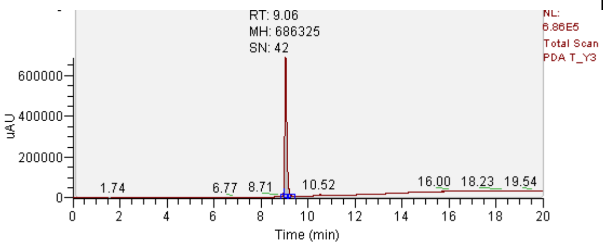

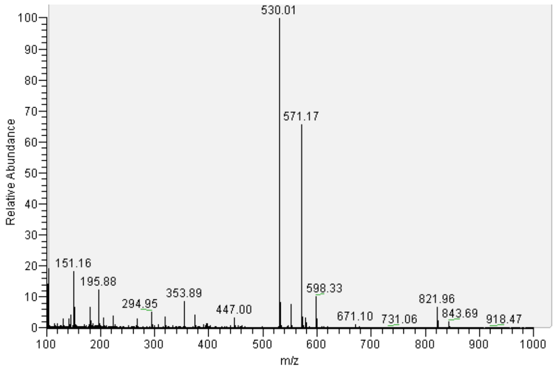

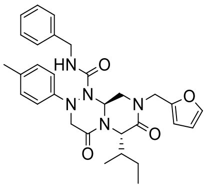

Exact Mass: 529.27
Molecular Weight: 529.63

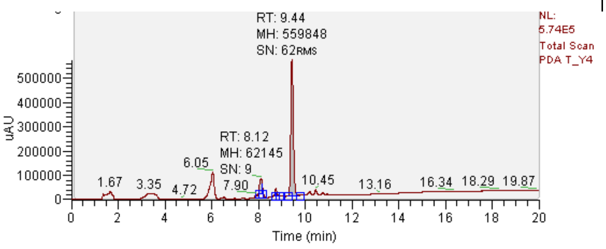

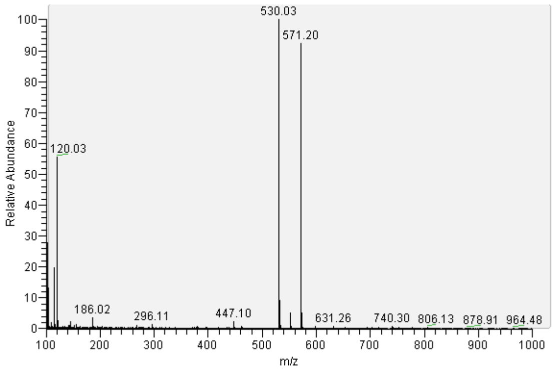

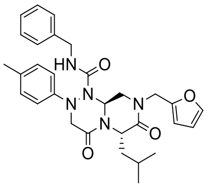

Exact Mass: 529.27
Molecular Weight: 529.63

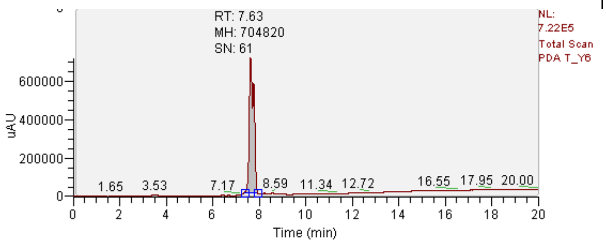

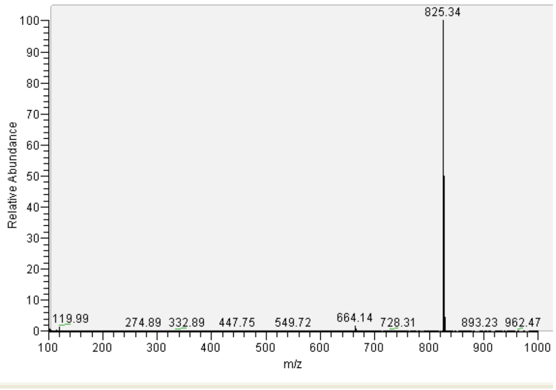

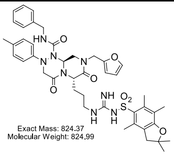

Exact Mass: 824.37
Molecular Weight: 824.99

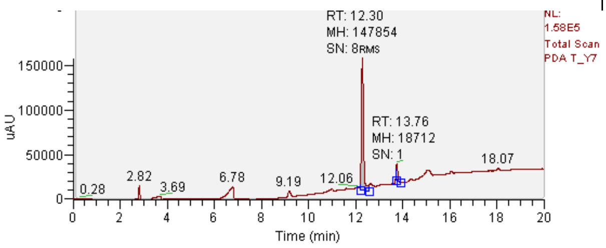

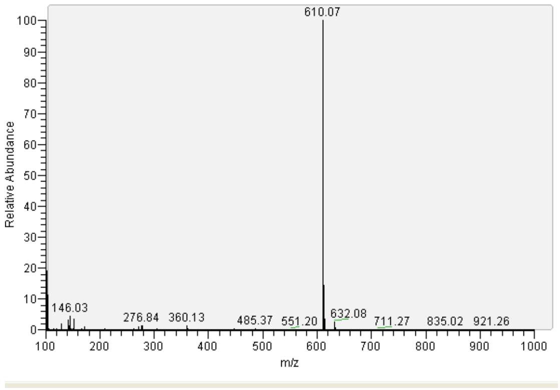

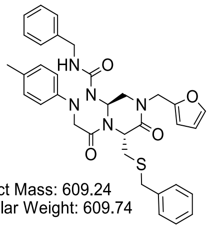

Exact Mass: 609.24
Molecular Weight: 609.74

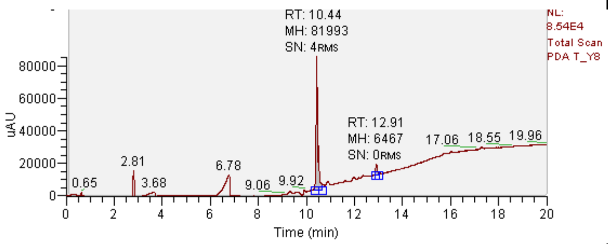

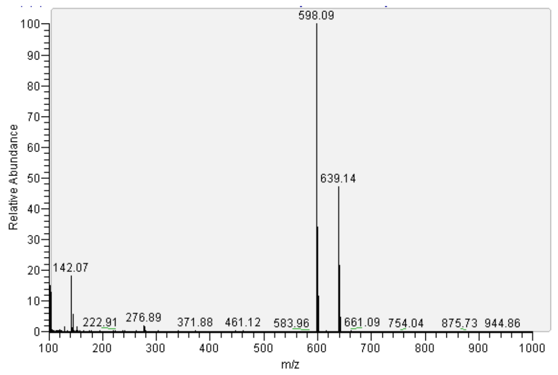

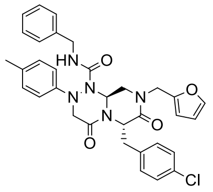

Exact Mass: 597.21
Molecular Weight: 598.09

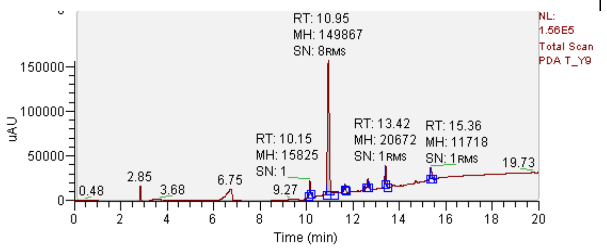

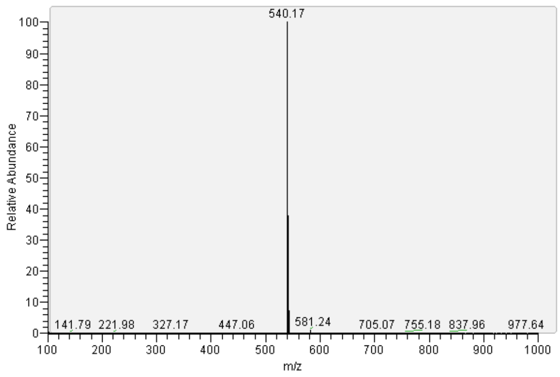

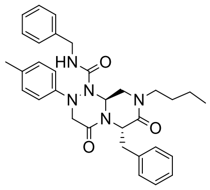

Exact Mass: 539.29
Molecular Weight: 539.67

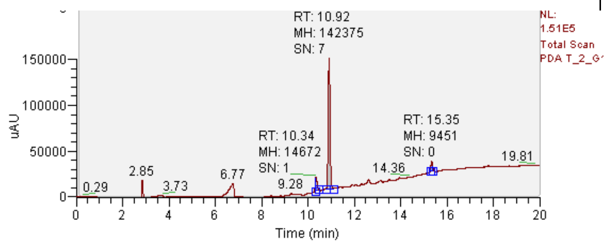

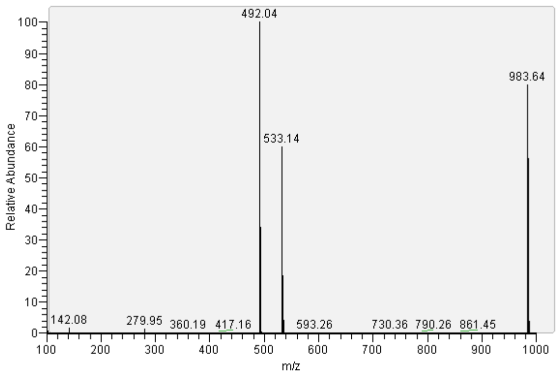

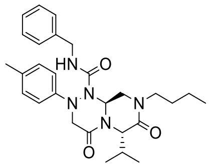

Exact Mass: 491.29
Molecular Weight: 491.63

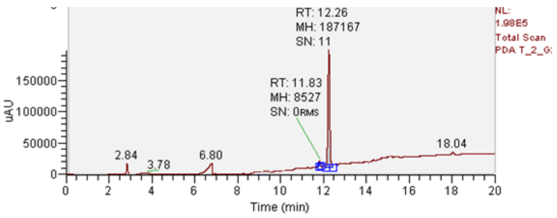

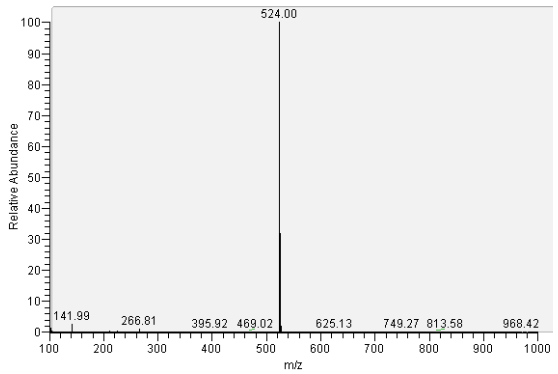

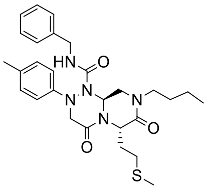

Exact Mass: 523.26
Molecular Weight: 523.69

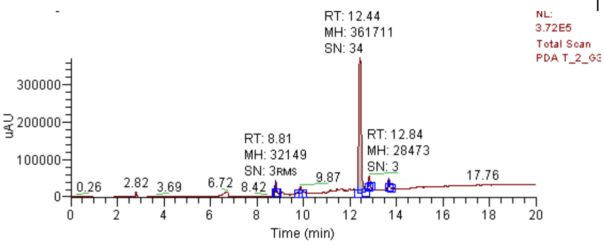

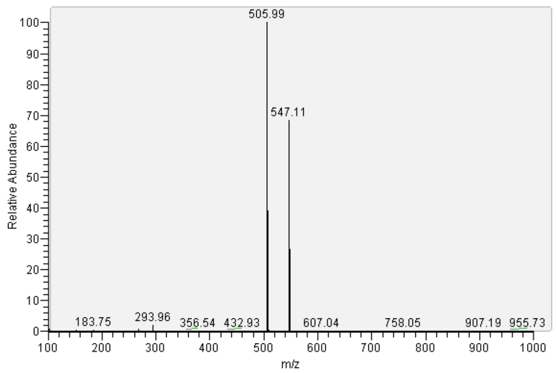

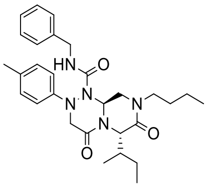

Exact Mass: 505.31
Molecular Weight: 505.65

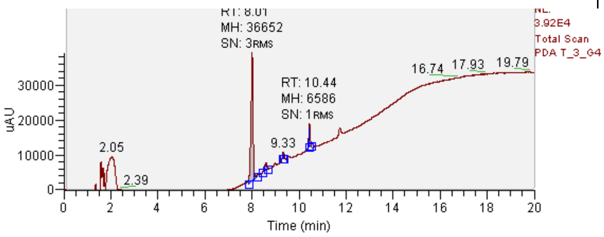

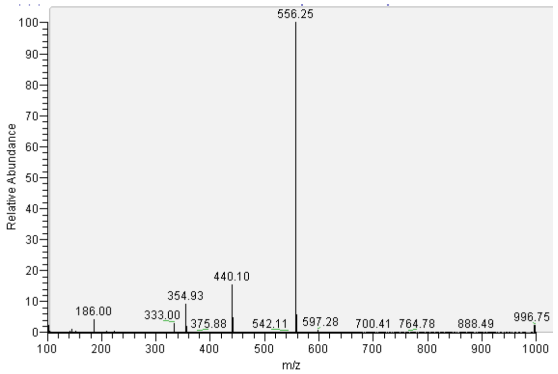

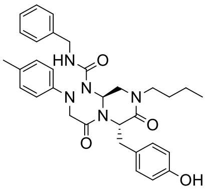

Exact Mass: 555.28
Molecular Weight: 555.67

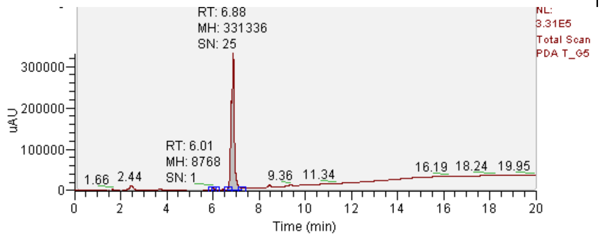

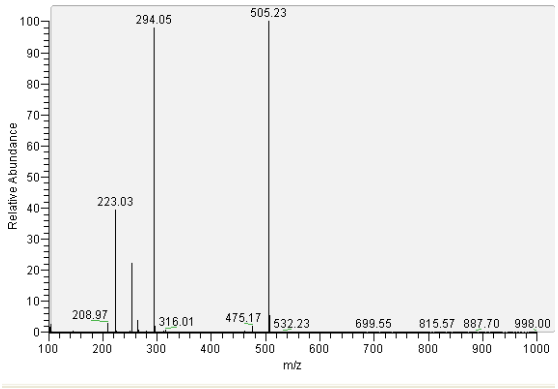
Relative Abundance
m/z
294.05
505.23
223.03

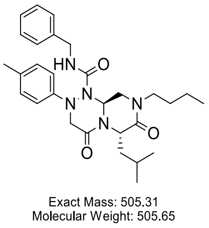
Exact Mass: 505.31
Molecular Weight: 505.65

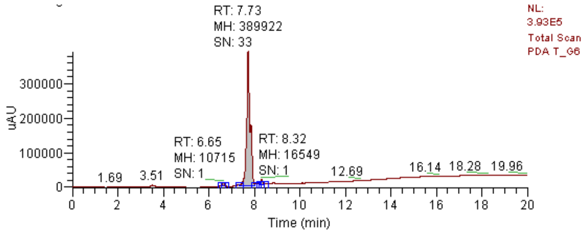
RT: 7.73
MH: 389922
SN: 33
NL: 3.93E5 Total Scan PDA T_G6
uAU
Time (min)

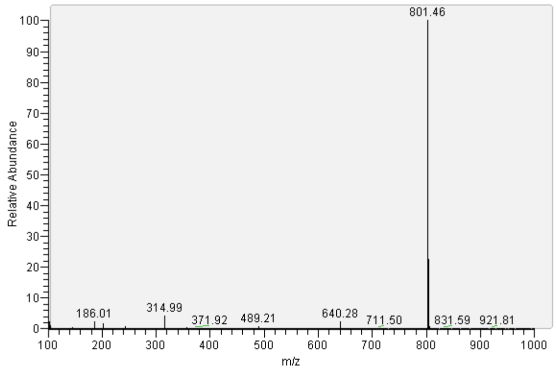
Relative Abundance
m/z
801.46

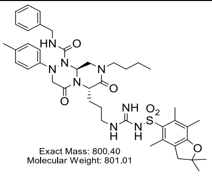
Exact Mass: 800.40
Molecular Weight: 801.01

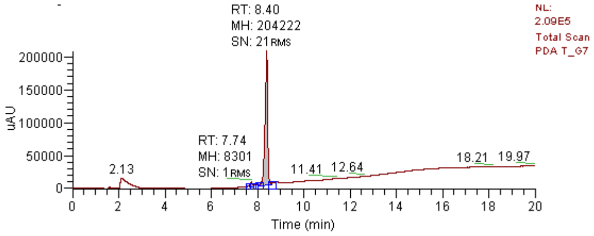
RT: 8.40
MH: 204222
SN: 21RMS
NL: 2.09E5 Total Scan PDA T_G7
uAU
Time (min)

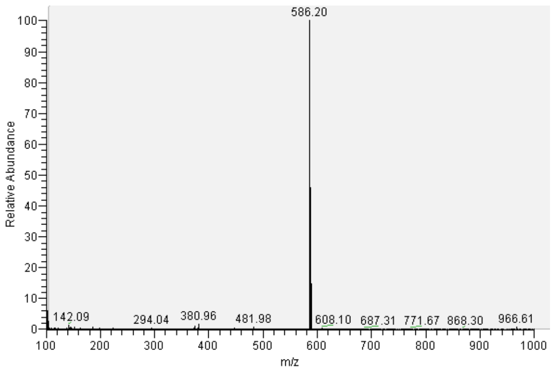
Relative Abundance
m/z
586.20

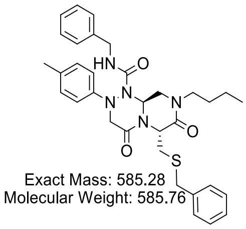
Exact Mass: 585.28
Molecular Weight: 585.76

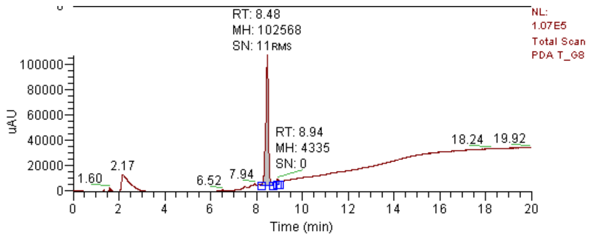
RT: 8.48
MH: 102568
SN: 11RMS
NL: 1.07E5 Total Scan PDA T_G8
uAU
Time (min)

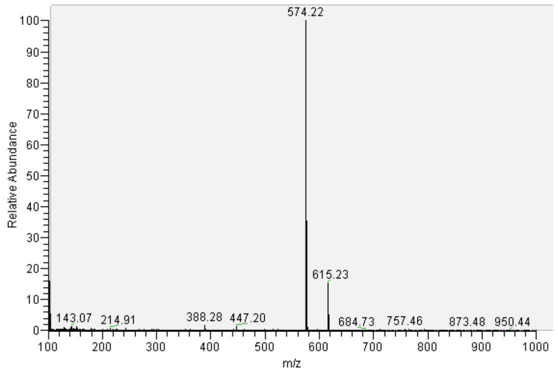

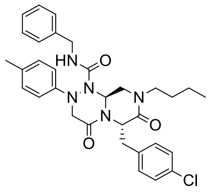

Exact Mass: 573.25
Molecular Weight: 574.11

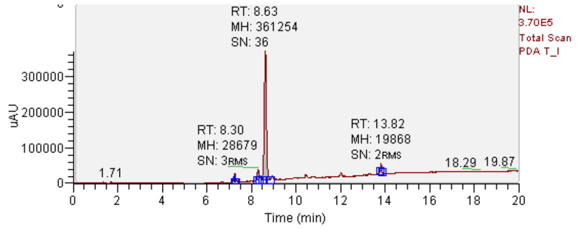

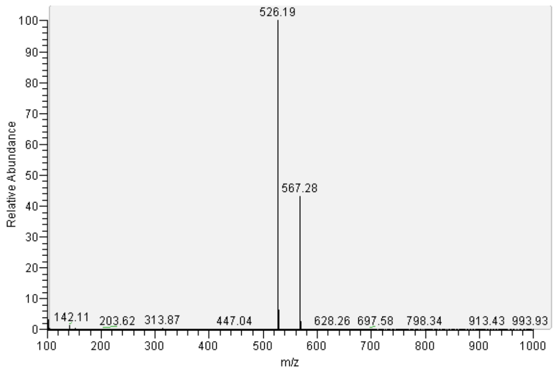

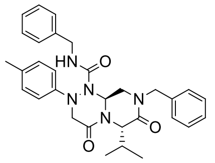

Exact Mass: 525.27
Molecular Weight: 525.64

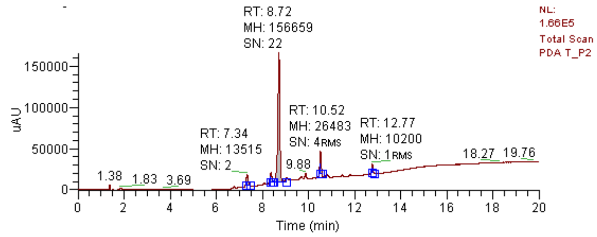

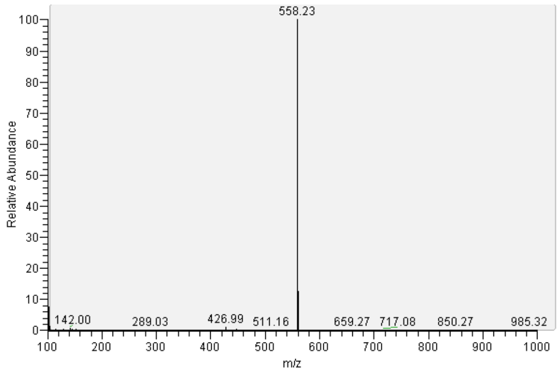

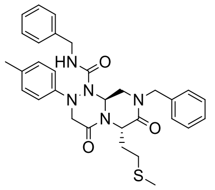

Exact Mass: 557.25
Molecular Weight: 557.71

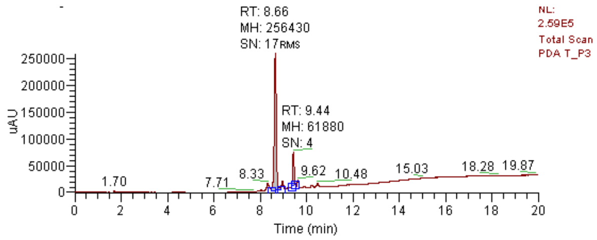

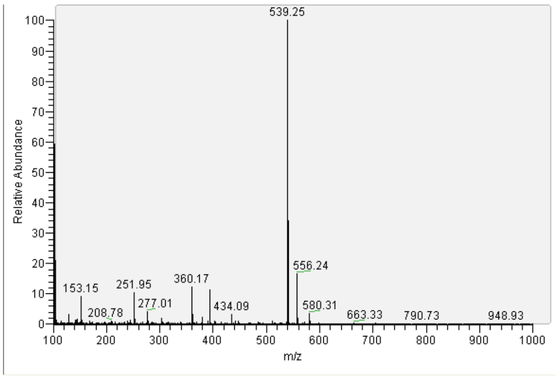

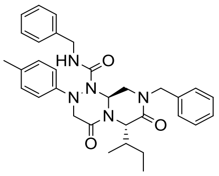

Exact Mass: 539.29
Molecular Weight: 539.67

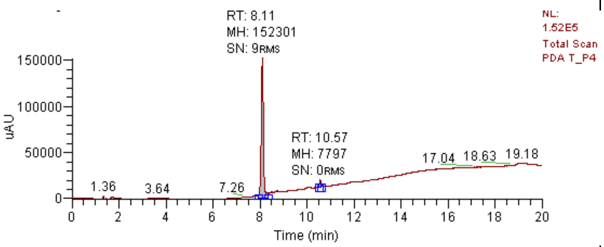

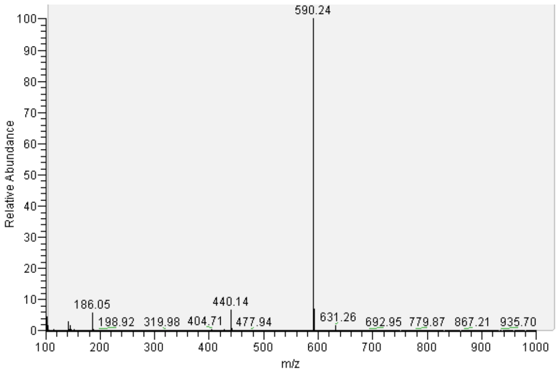

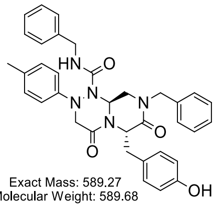

Exact Mass: 589.27
Molecular Weight: 589.68

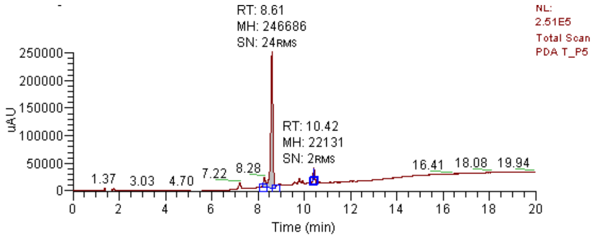

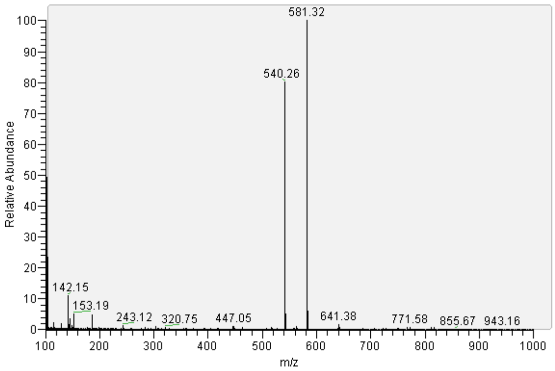

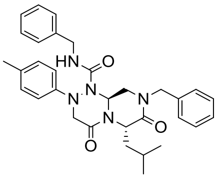

Exact Mass: 539.29
Molecular Weight: 539.67

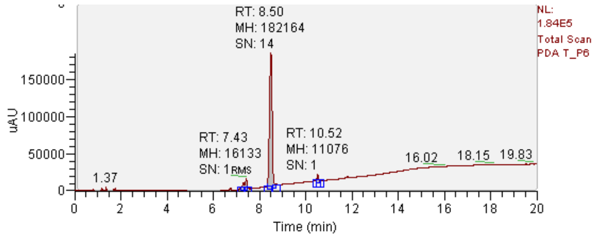

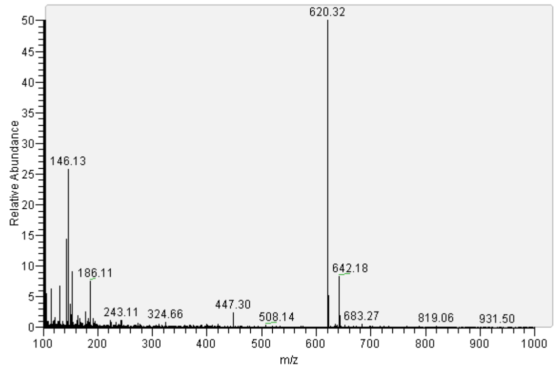

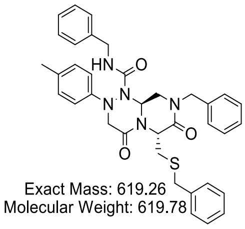

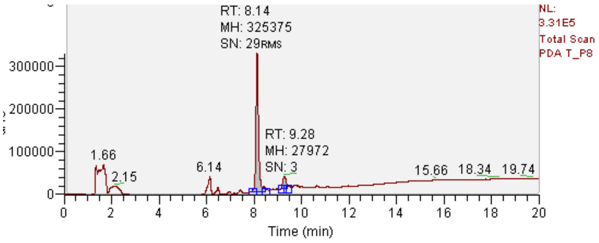

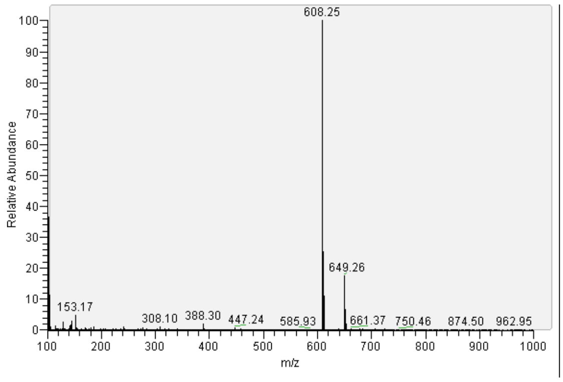

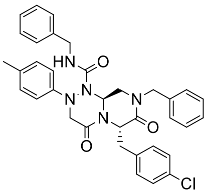

Exact Mass: 607.24
Molecular Weight: 608.13

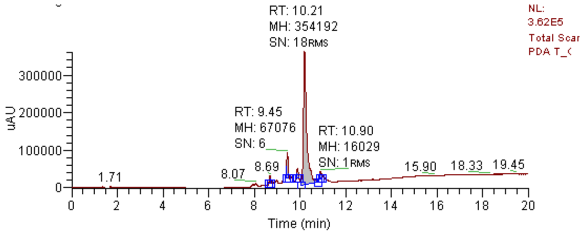

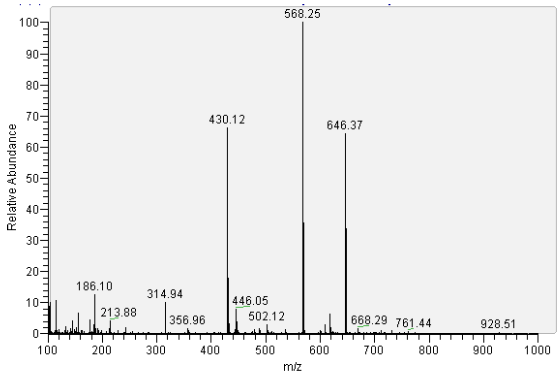

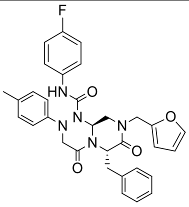

Exact Mass: 567.23
Molecular Weight: 567.61

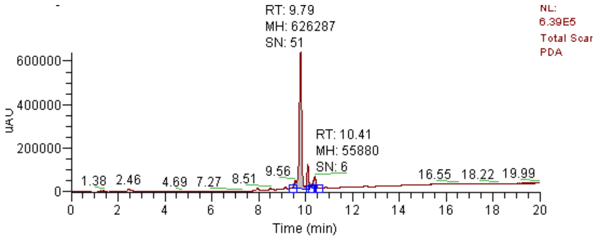

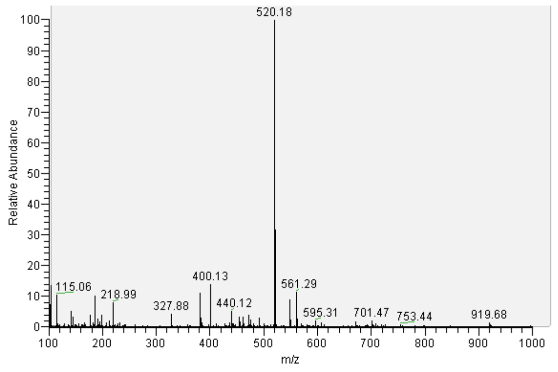

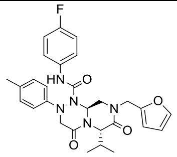

Exact Mass: 519.23
Molecular Weight: 519.57

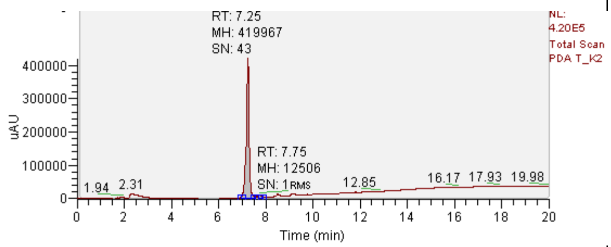

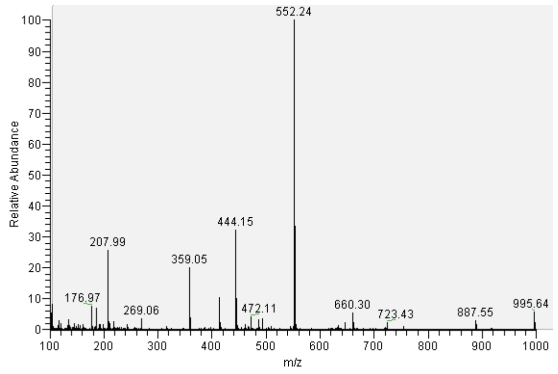

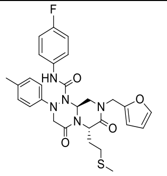

Exact Mass: 551.20
Molecular Weight: 551.63

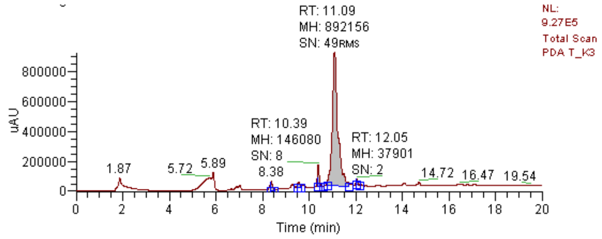

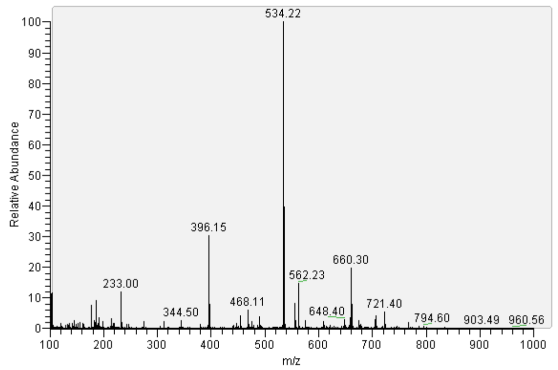

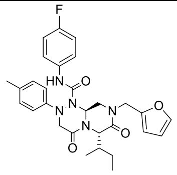

Exact Mass: 533.24
Molecular Weight: 533.59

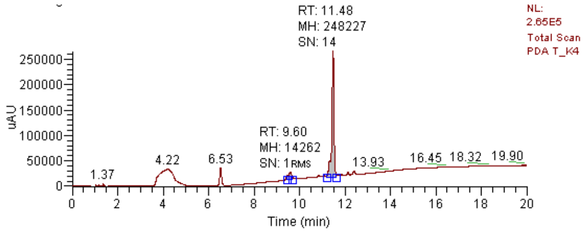

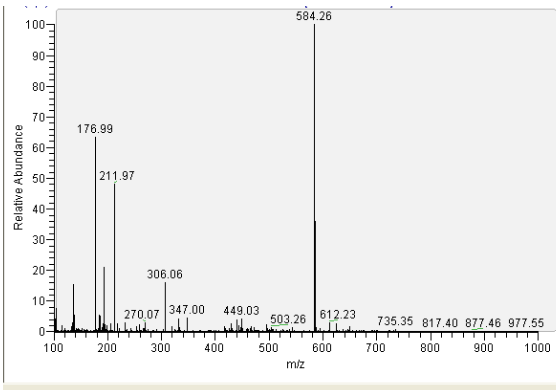

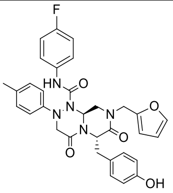

Exact Mass: 583.22
Molecular Weight: 583.61

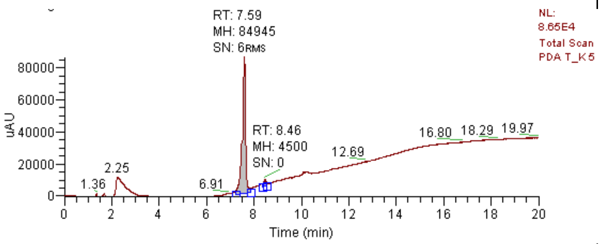

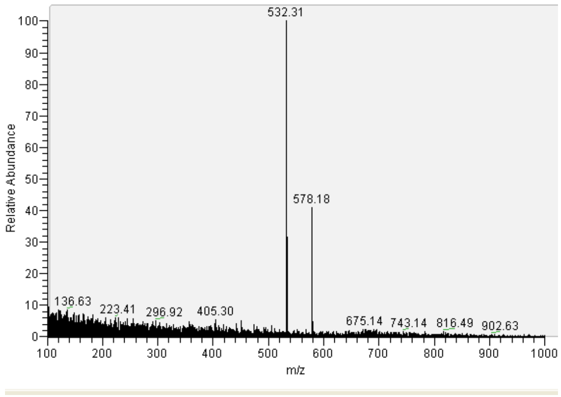

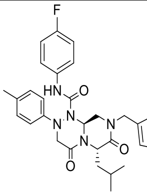

Exact Mass: 533.24
Molecular Weight: 533.59

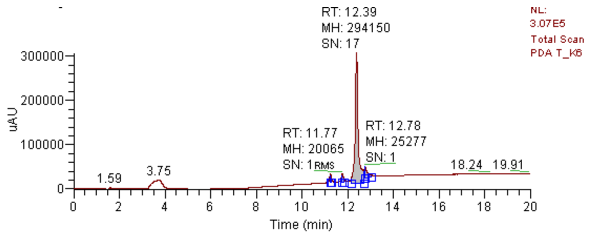

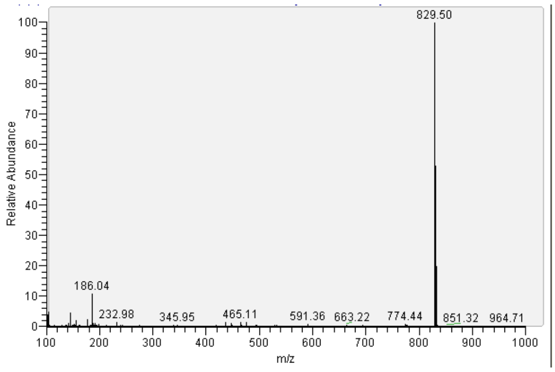

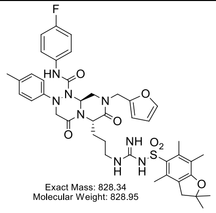

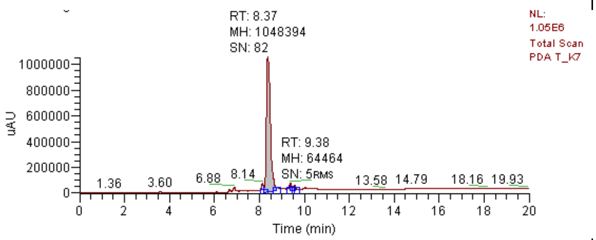

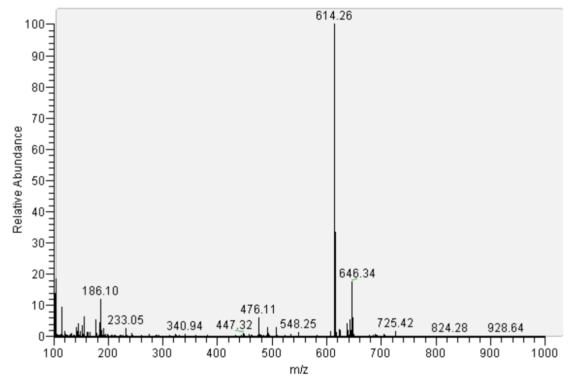
614.26
646.34
186.10
233.05
340.94
447.32
476.11
548.25
725.42
824.28
928.64
Relative Abundance
m/z

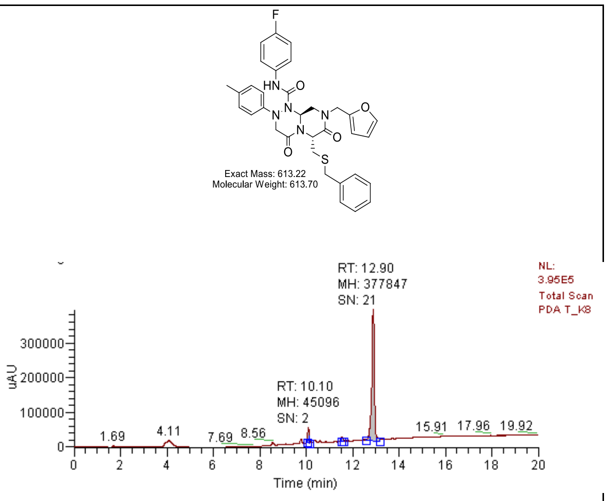
F
HN
O
N
N
N
O
O
S
Exact Mass: 613.22
Molecular Weight: 613.70
RT: 12.90
MH: 377847
SN: 21
RT: 10.10
MH: 45096
SN: 2
NL:
3.95E5
Total Scan
PDA T_K8
uAU
Time (min)

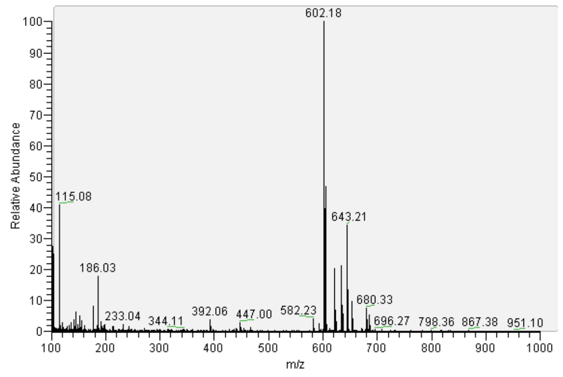
602.18
643.21
680.33
115.08
186.03
233.04
344.11
392.06
447.00
582.23
696.27
798.36
867.38
951.10
Relative Abundance
m/z

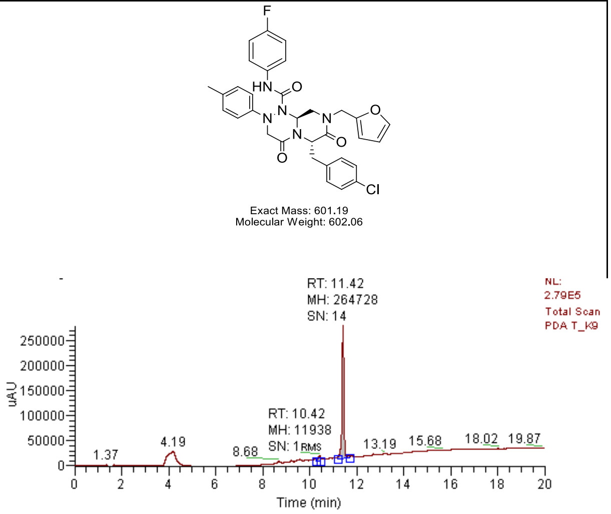
F
HN
O
N
N
N
O
O
Cl
Exact Mass: 601.19
Molecular Weight: 602.06
RT: 11.42
MH: 264728
SN: 14
RT: 10.42
MH: 11938
SN: 1RMS
NL:
2.79E5
Total Scan
PDA T_K9
uAU
Time (min)

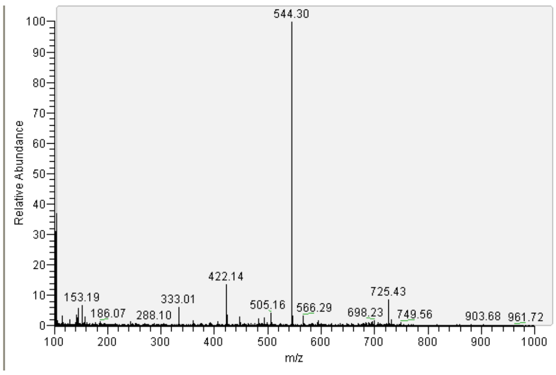
544.30
422.14
153.19
186.07
288.10
333.01
505.16
566.29
698.23
725.43
749.56
903.68
961.72
Relative Abundance
m/z

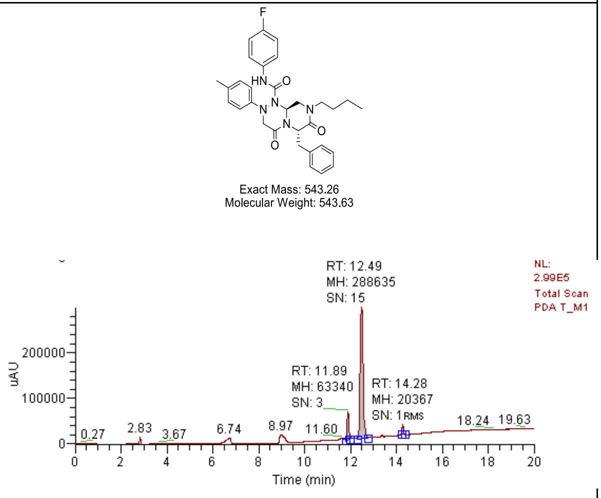
F
HN
O
N
N
N
O
O
Exact Mass: 543.26
Molecular Weight: 543.63
RT: 12.49
MH: 288635
SN: 15
RT: 11.89
MH: 63340
SN: 3
RT: 14.28
MH: 20367
SN: 1RMS
NL:
2.99E5
Total Scan
PDA T_M1
uAU
Time (min)

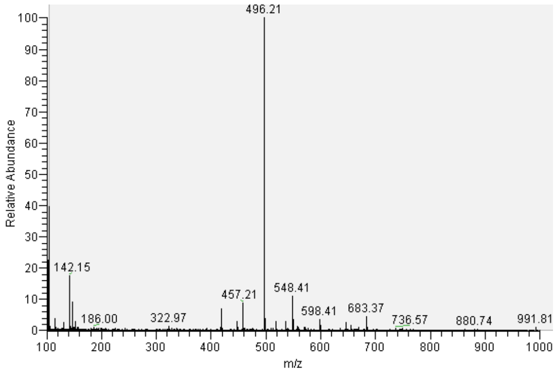

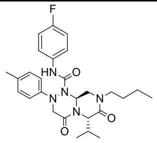

Exact Mass: 495.26
Molecular Weight: 495.59

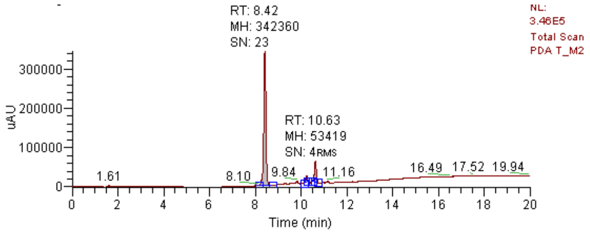

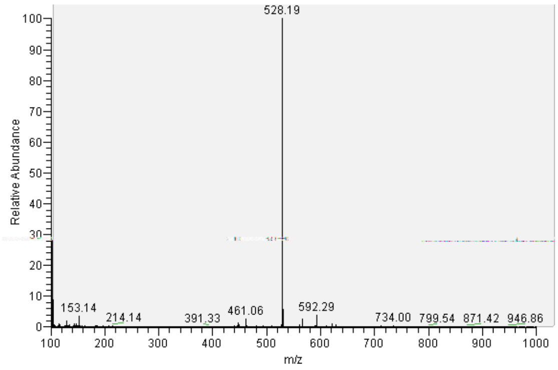

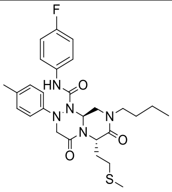

Exact Mass: 527.24
Molecular Weight: 527.65

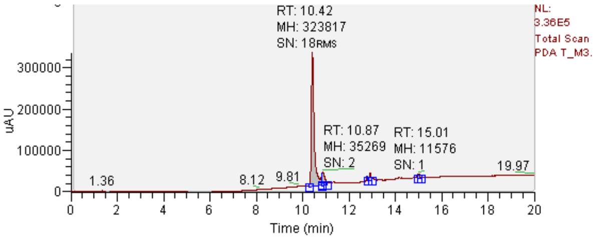

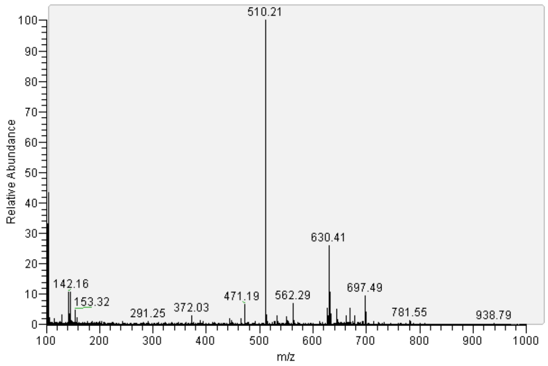

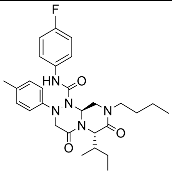

Exact Mass: 509.28
Molecular Weight: 509.62

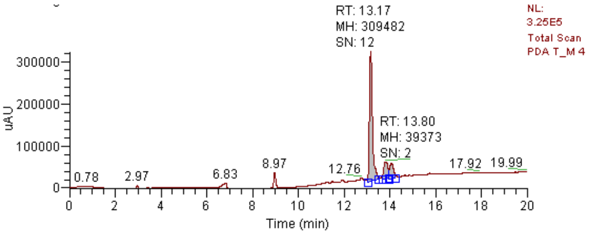

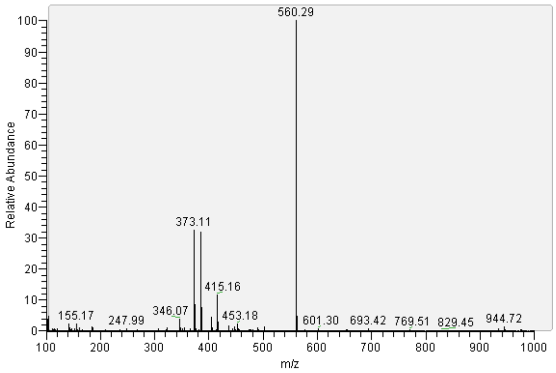

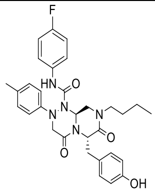

Exact Mass: 559.26
Molecular Weight: 559.63

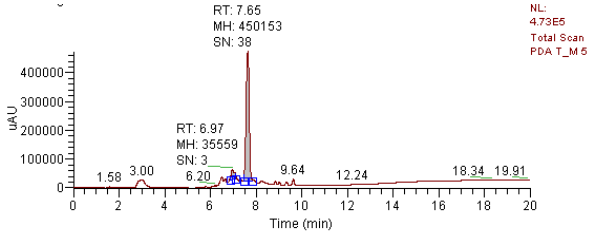

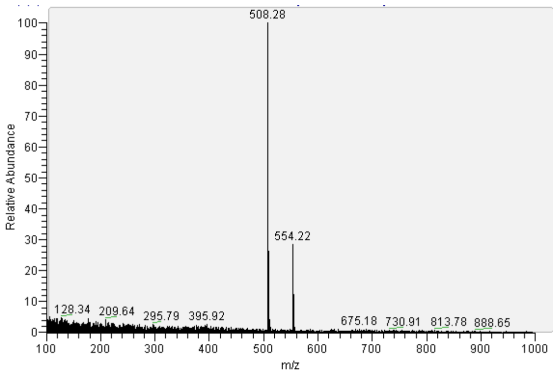

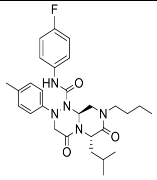

Exact Mass: 509.28
Molecular Weight: 509.62

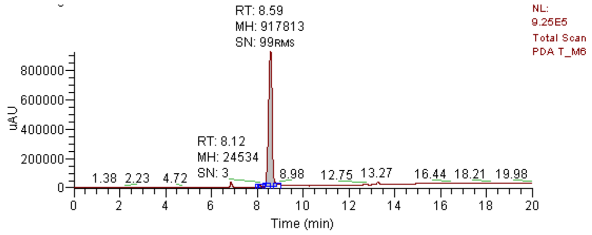

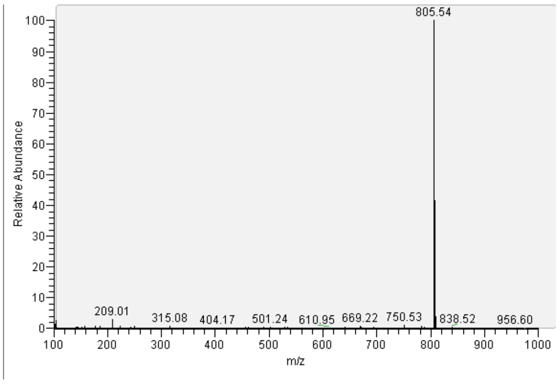

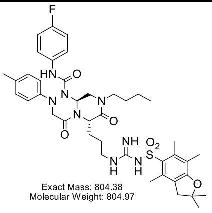

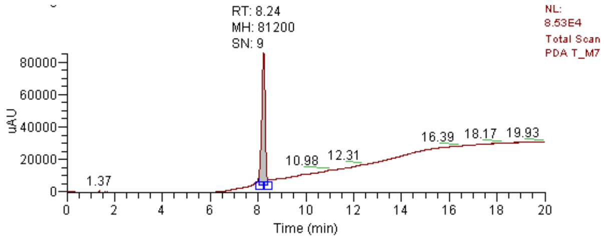

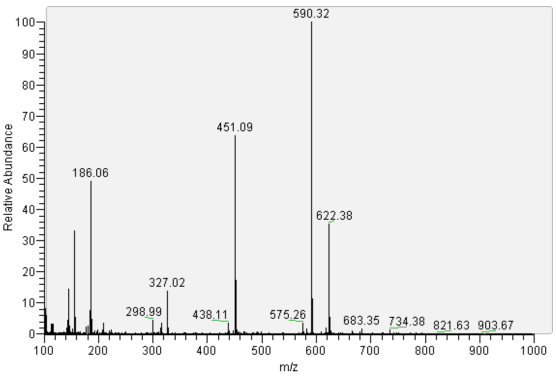

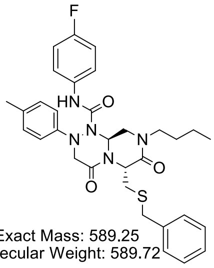

Exact Mass: 589.25
Molecular Weight: 589.72

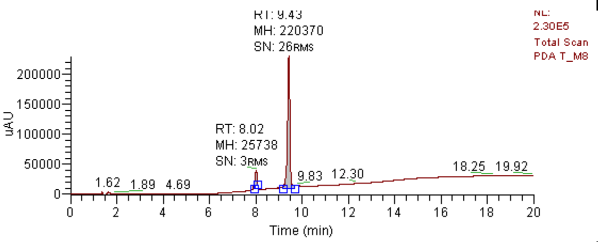

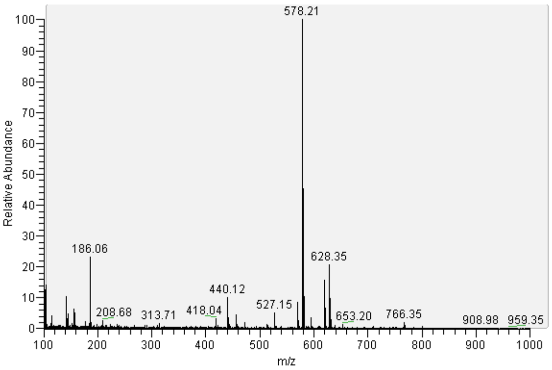

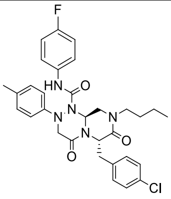

Exact Mass: 577.23
Molecular Weight: 578.08

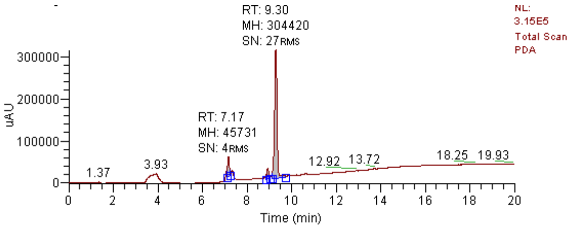

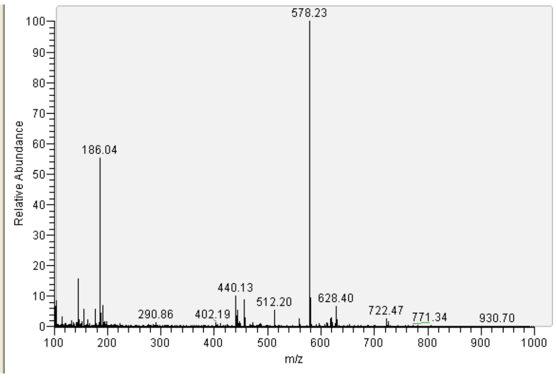

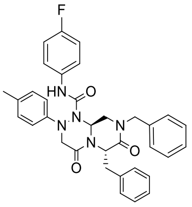

Exact Mass: 577.25
Molecular Weight: 577.65

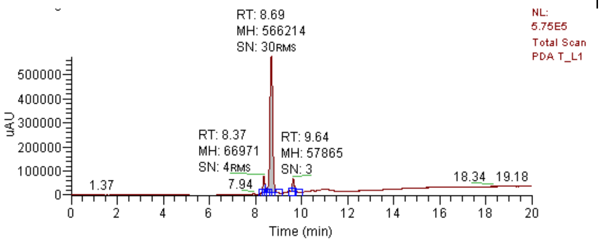

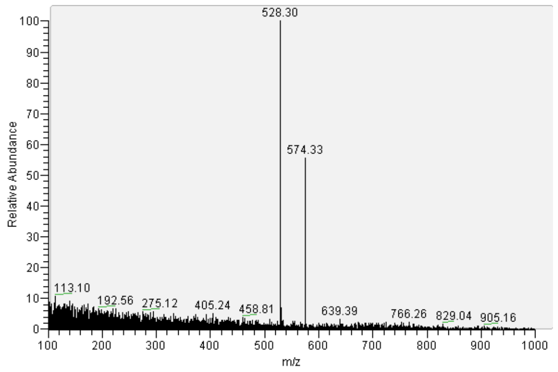

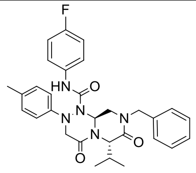

Exact Mass: 529.25
Molecular Weight: 529.61

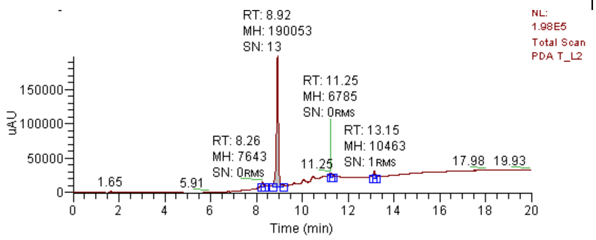

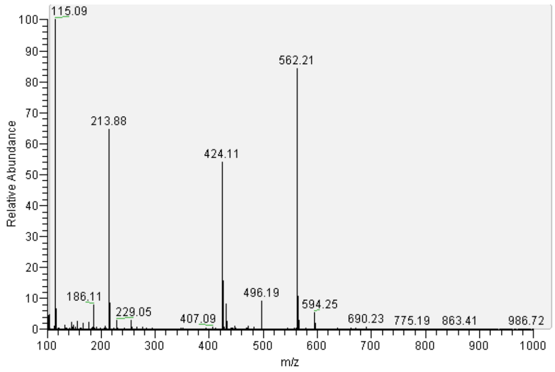

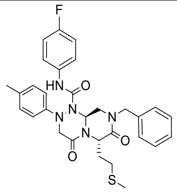

Exact Mass: 561.22
Molecular Weight: 561.67

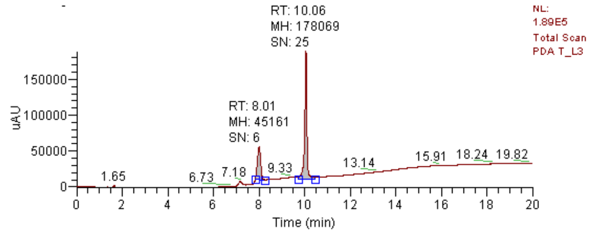

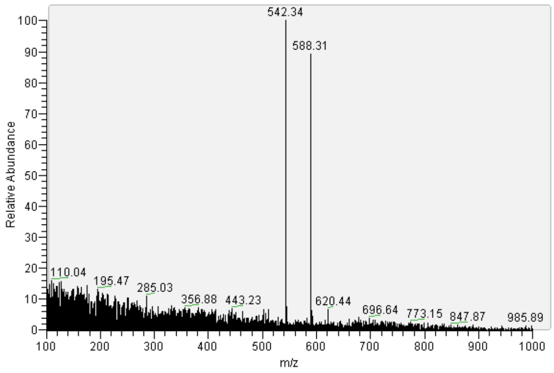

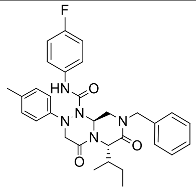

Exact Mass: 543.26
Molecular Weight: 543.63

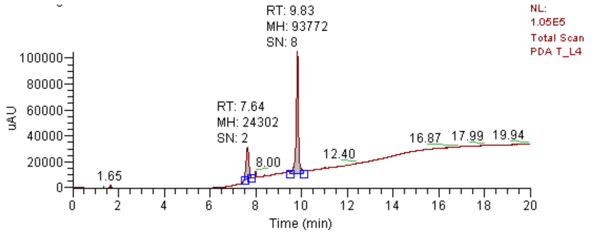

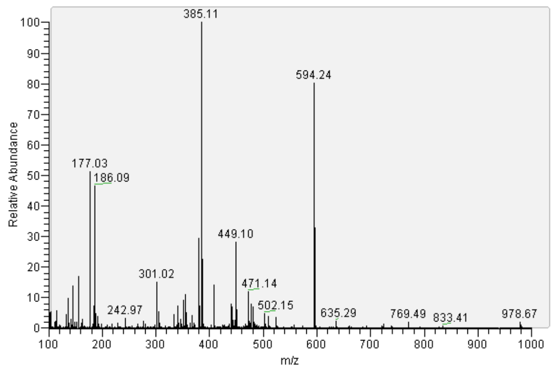

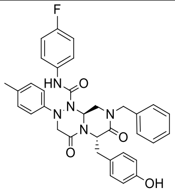

Exact Mass: 593.24
Molecular Weight: 593.65

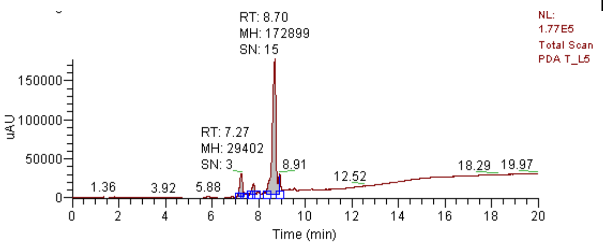

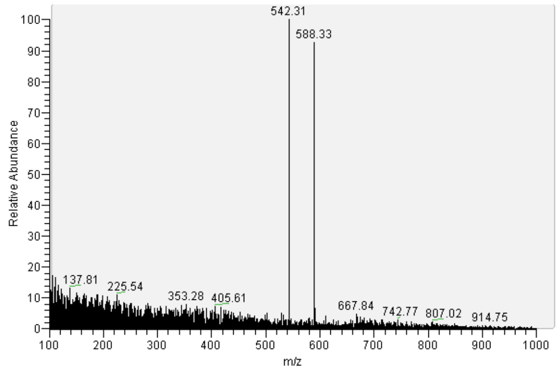

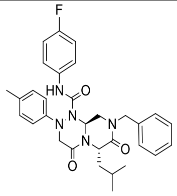

Exact Mass: 543.26
Molecular Weight: 543.63

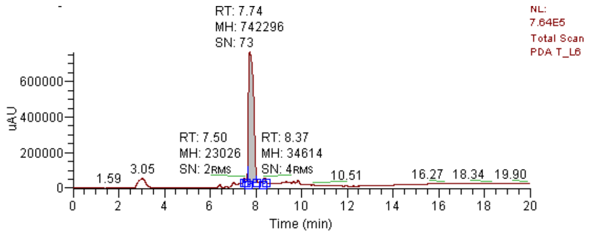

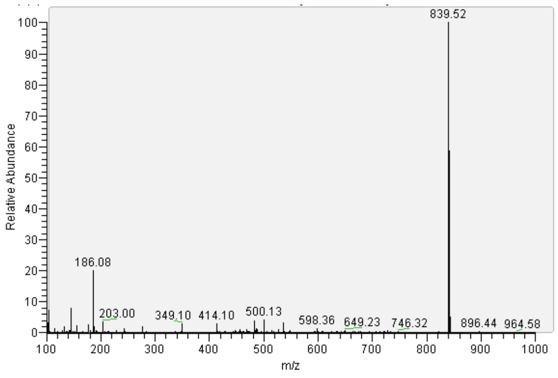

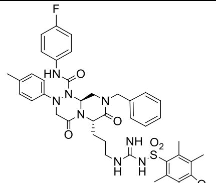

Exact Mass: 838.36
Molecular Weight: 838.99

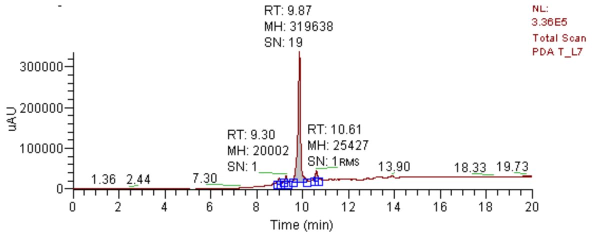

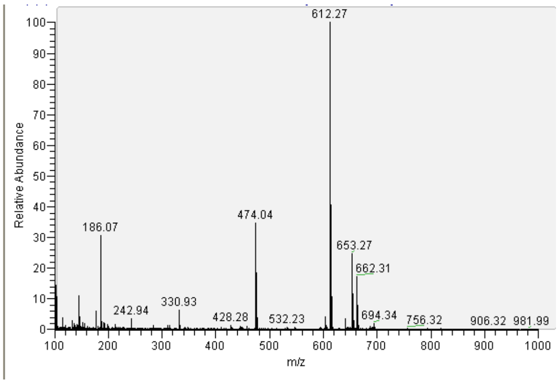

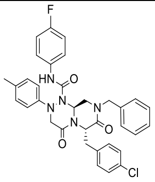

Exact Mass: 611.21
Molecular Weight: 612.09

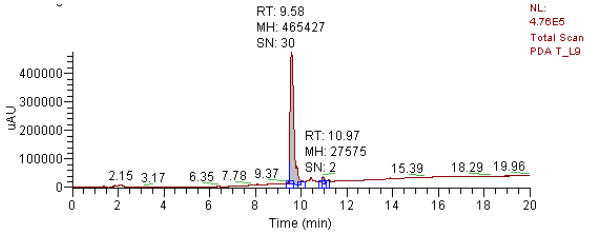

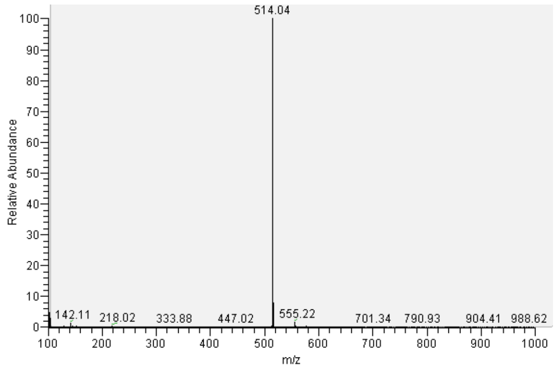

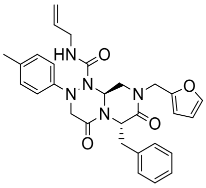

Exact Mass: 513.24
Molecular Weight: 513.59

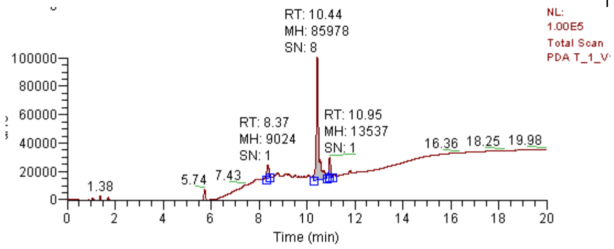

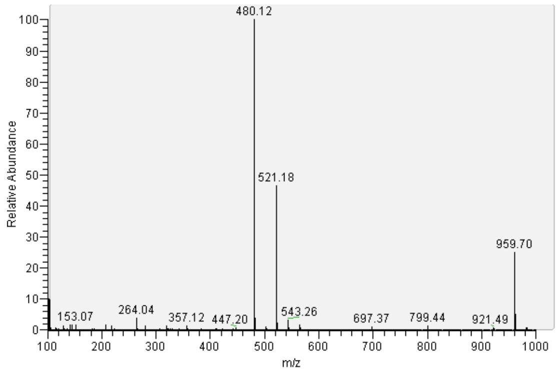

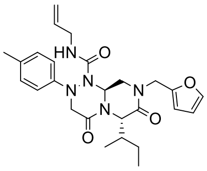

Exact Mass: 479.25
Molecular Weight: 479.57

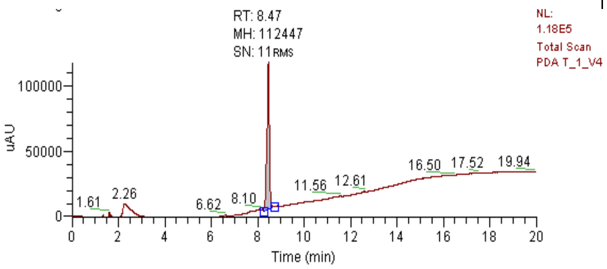

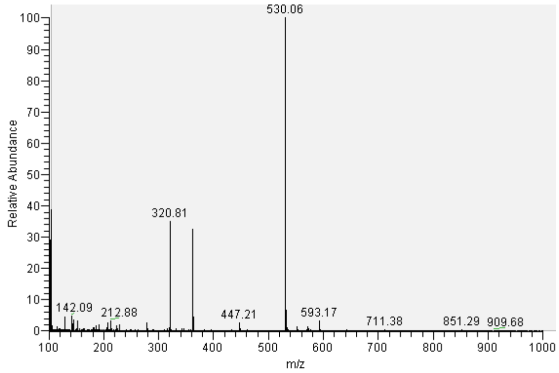

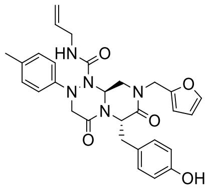

Exact Mass: 529.23
Molecular Weight: 529.59

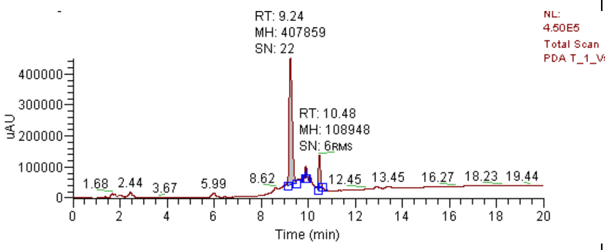

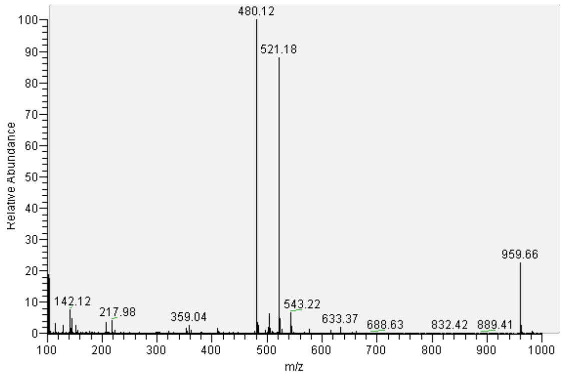

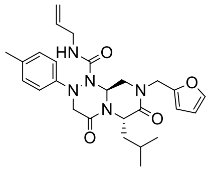

Exact Mass: 479.25
Molecular Weight: 479.57

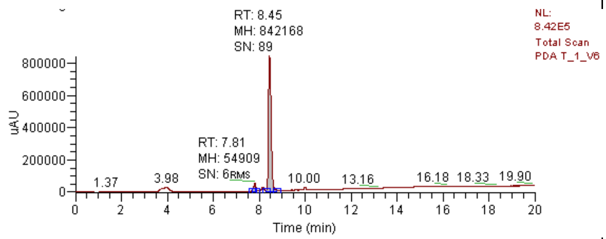

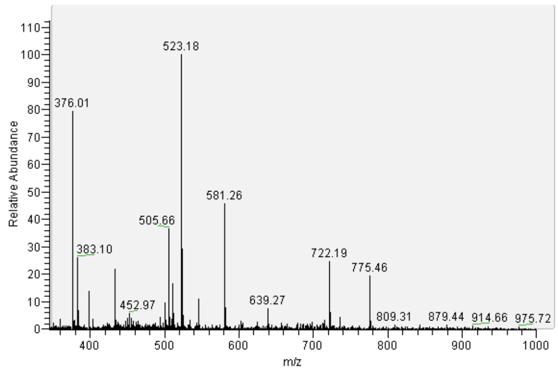

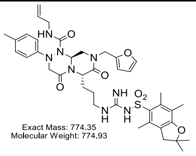

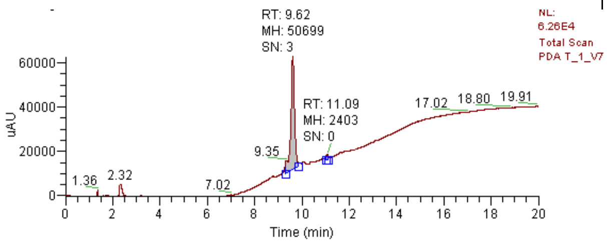

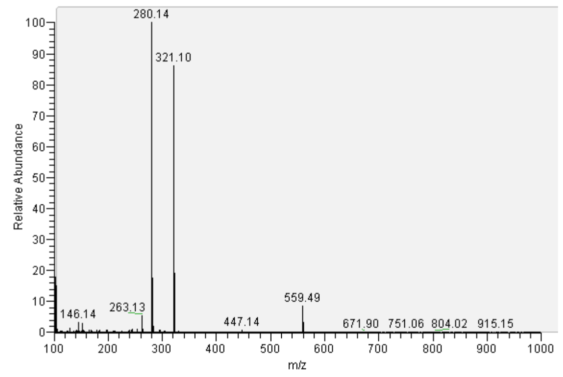

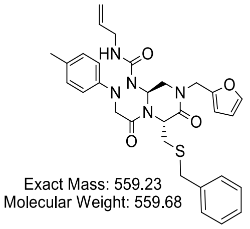

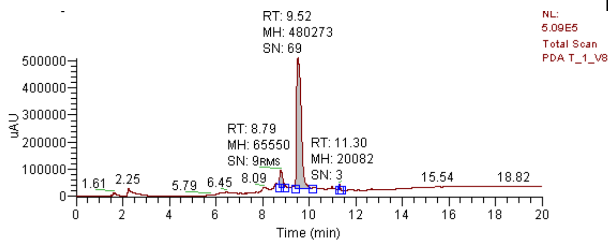

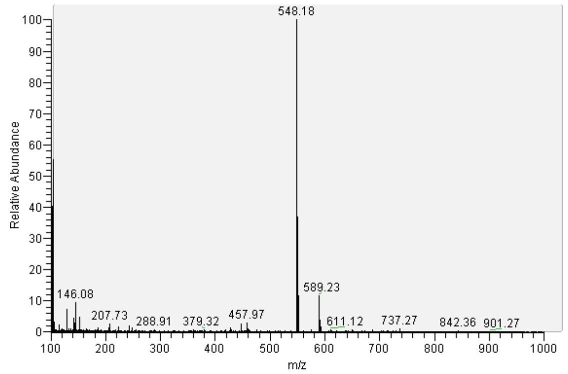

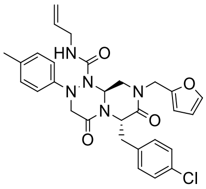

Exact Mass: 547.20
Molecular Weight: 548.03

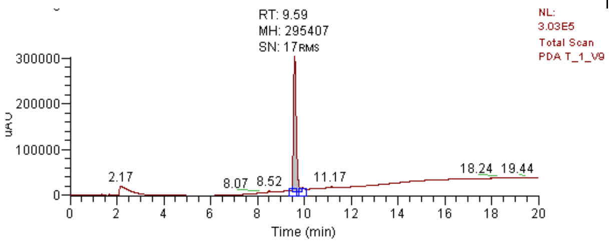

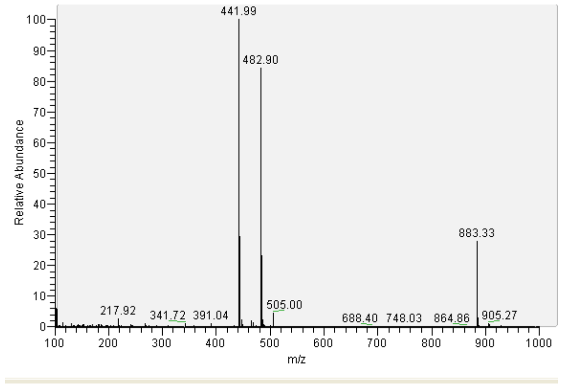

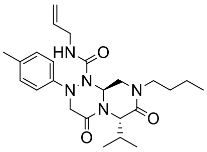

Exact Mass: 441.27
Molecular Weight: 441.57

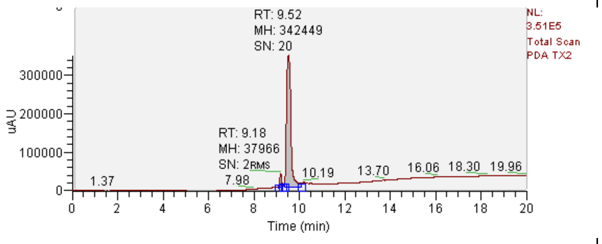

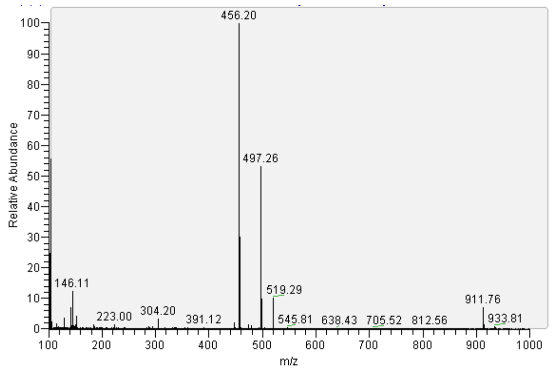

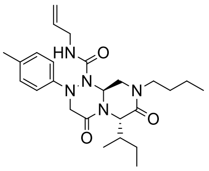

Exact Mass: 455.29
Molecular Weight: 455.59

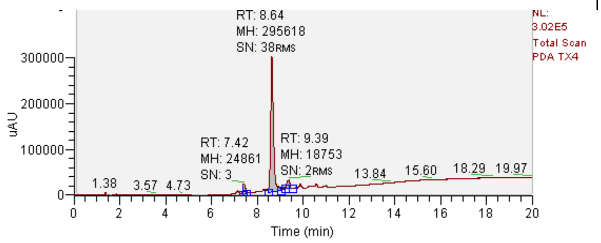

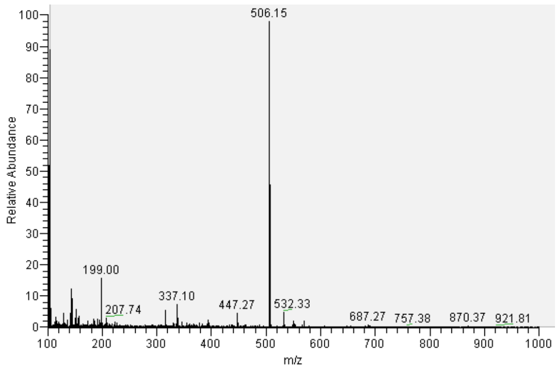

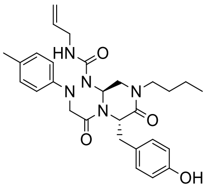

Exact Mass: 505.27
Molecular Weight: 505.61

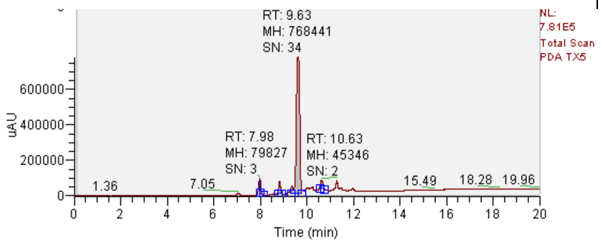

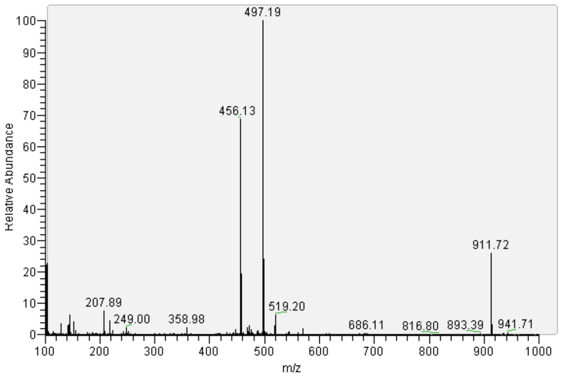

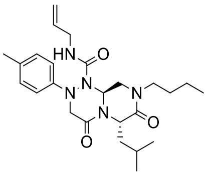

Exact Mass: 455.29
Molecular Weight: 455.59

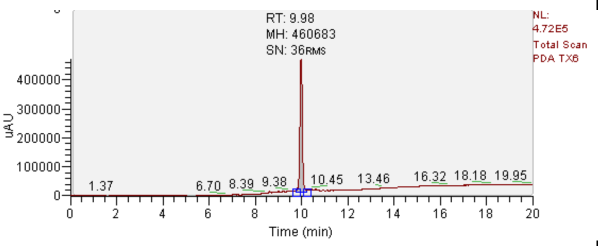

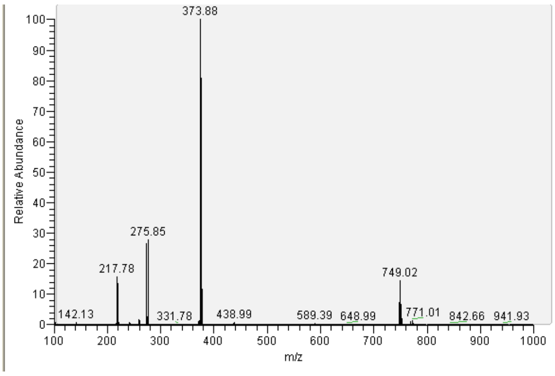

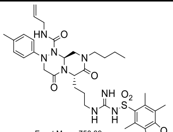
Exact Mass: 750.39
Molecular Weight: 750.95

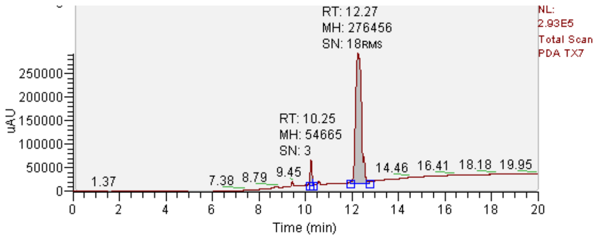

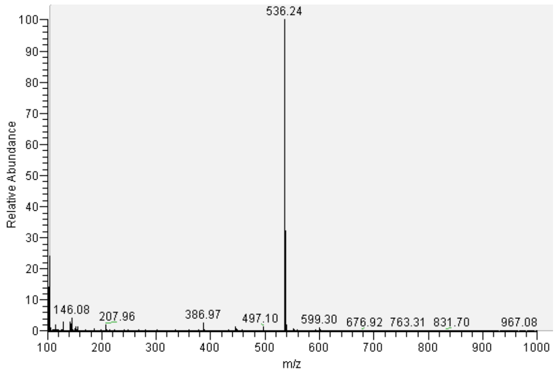

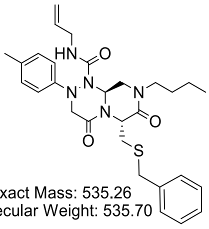
Exact Mass: 535.26
Molecular Weight: 535.70

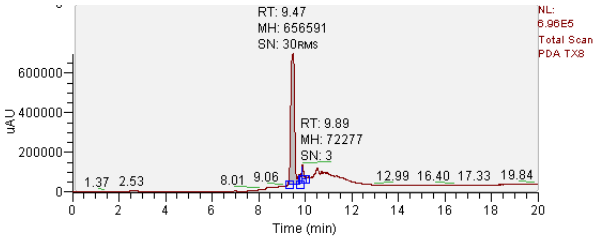

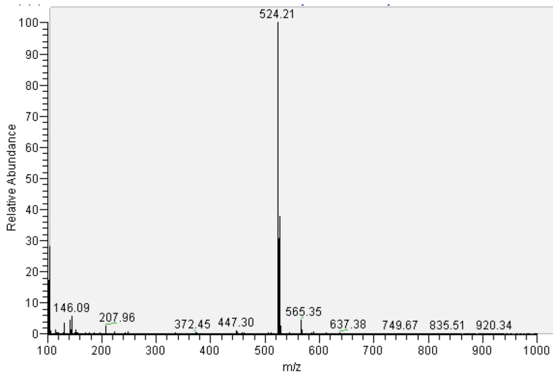

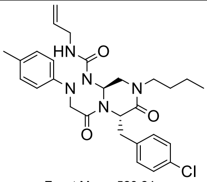
Exact Mass: 523.24
Molecular Weight: 524.05

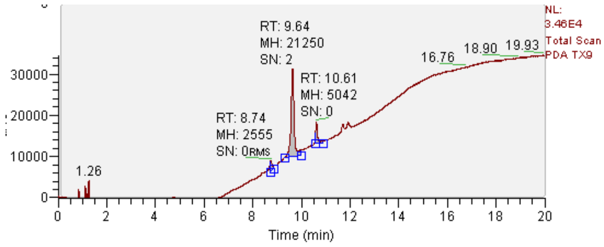

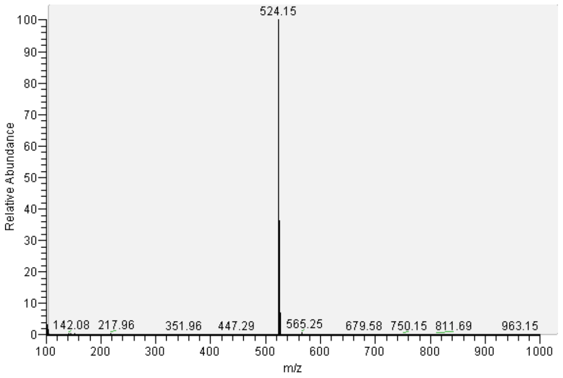

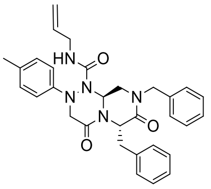

Exact Mass: 523.26
Molecular Weight: 523.63

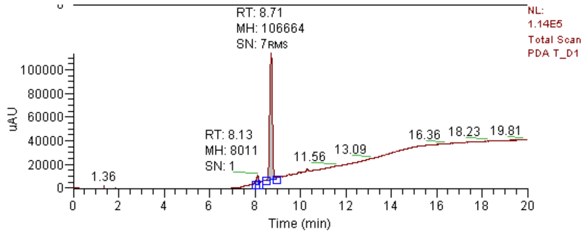

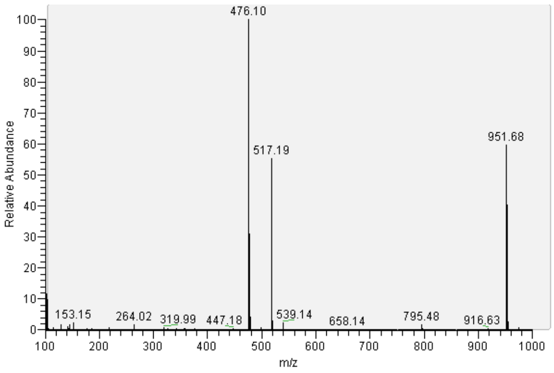

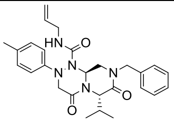

Exact Mass: 475.26
Molecular Weight: 475.58

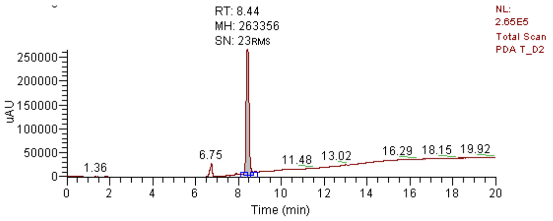

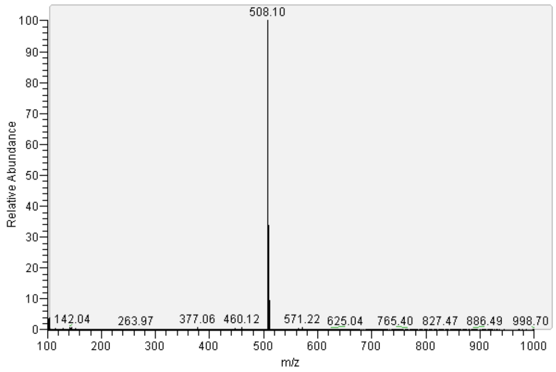

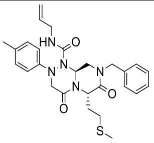

Exact Mass: 507.23
Molecular Weight: 507.65

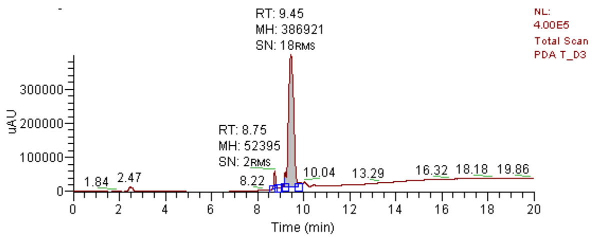

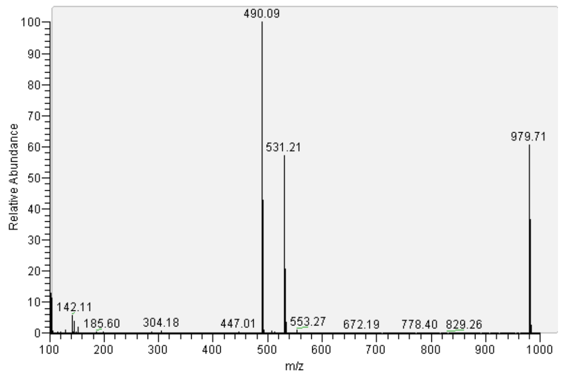

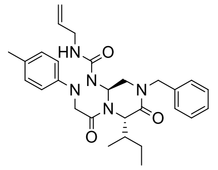

Exact Mass: 489.27
Molecular Weight: 489.61

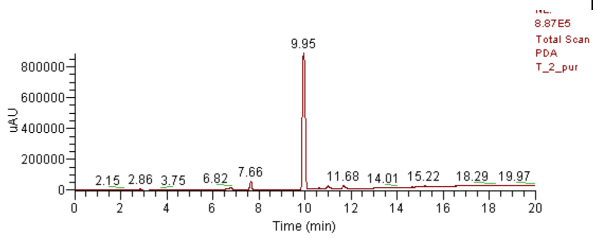

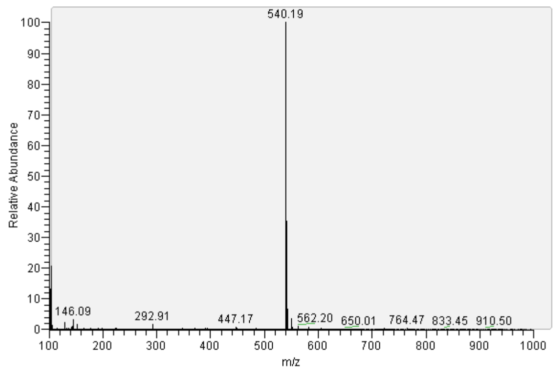

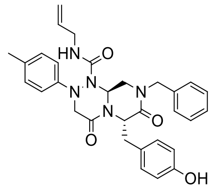

Exact Mass: 539.25
Molecular Weight: 539.62

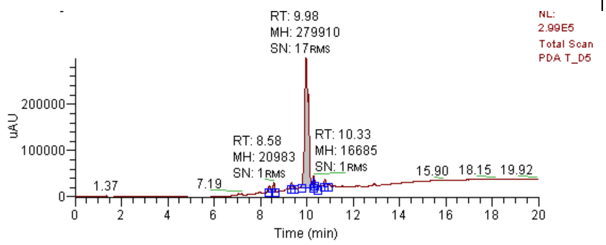

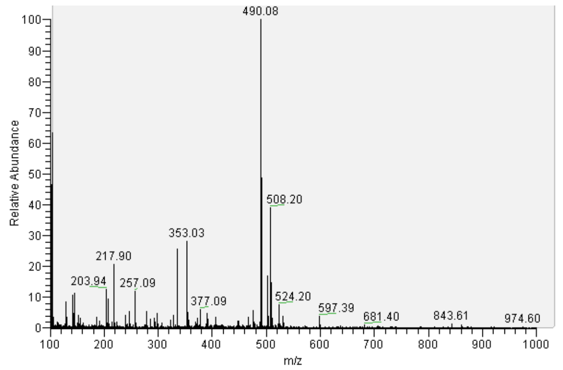

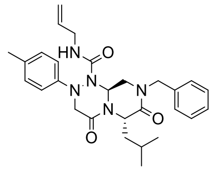

Exact Mass: 489.27
Molecular Weight: 489.61

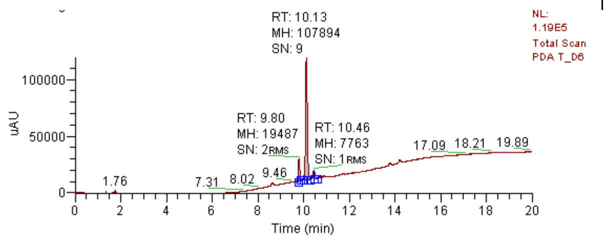

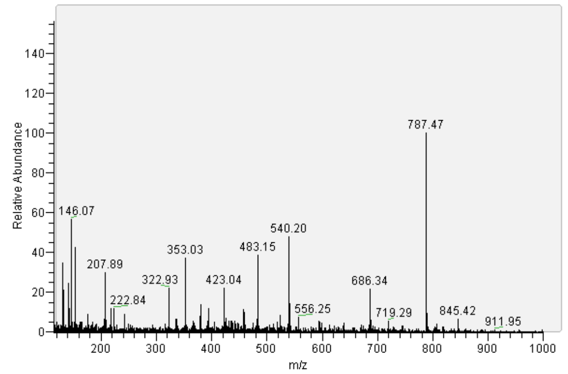
787.47
146.07
207.89
353.03
483.15
540.20
322.93
423.04
222.84
686.34
556.25
719.29
845.42
911.95
Relative Abundance
m/z

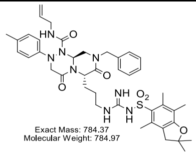
Exact Mass: 784.37
Molecular Weight: 784.97

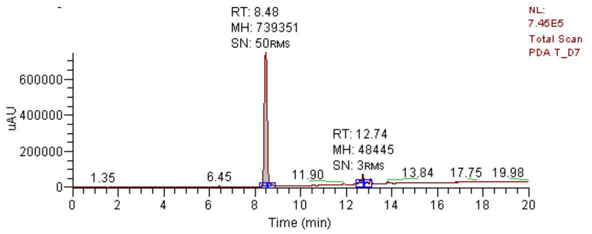
RT: 8.48
MH: 739351
SN: 50RMS
NL:
7.45E5
Total Scan
PDA T_D7
RT: 12.74
MH: 48445
SN: 3RMS
1.35
6.45
11.90
13.84
17.75
19.98
uAU
Time (min)

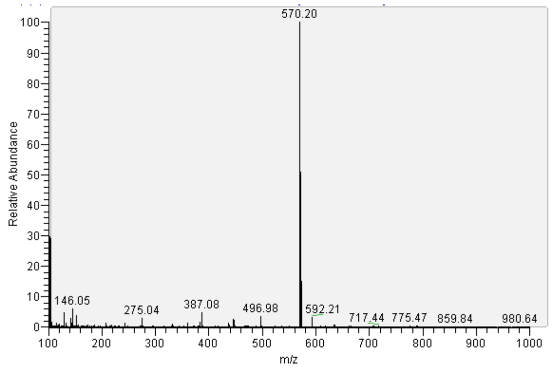
570.20
146.05
275.04
387.08
496.98
592.21
717.44
775.47
859.84
980.64
Relative Abundance
m/z

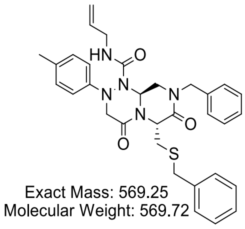
Exact Mass: 569.25
Molecular Weight: 569.72

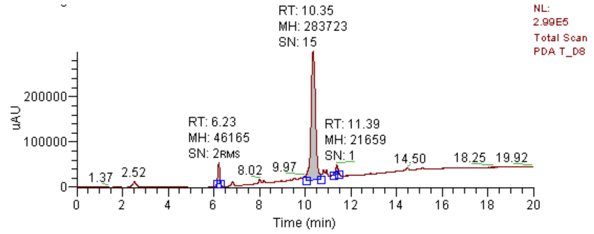
RT: 10.35
MH: 283723
SN: 15
NL:
2.99E5
Total Scan
PDA T_D8
RT: 6.23
MH: 46165
SN: 2RMS
RT: 11.39
MH: 21659
SN: 1
1.37
2.52
8.02
9.97
14.50
18.25
19.92
uAU
Time (min)

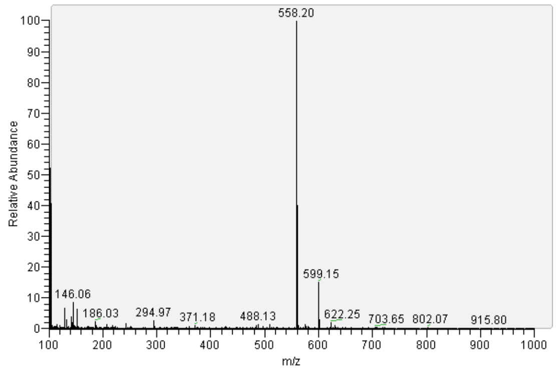
558.20
599.15
146.06
186.03
294.97
371.18
488.13
622.25
703.65
802.07
915.80
Relative Abundance
m/z

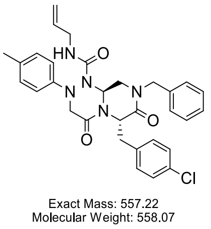
Exact Mass: 557.22
Molecular Weight: 558.07

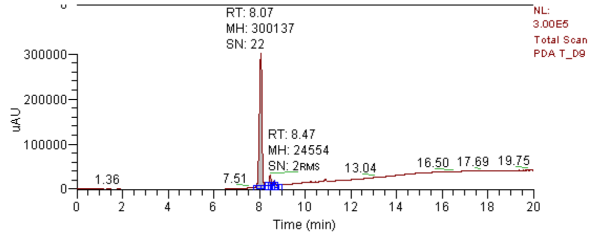
RT: 8.07
MH: 300137
SN: 22
NL:
3.00E5
Total Scan
PDA T_D9
RT: 8.47
MH: 24554
SN: 2RMS
1.36
7.51
13.04
16.50
17.69
19.75
uAU
Time (min)

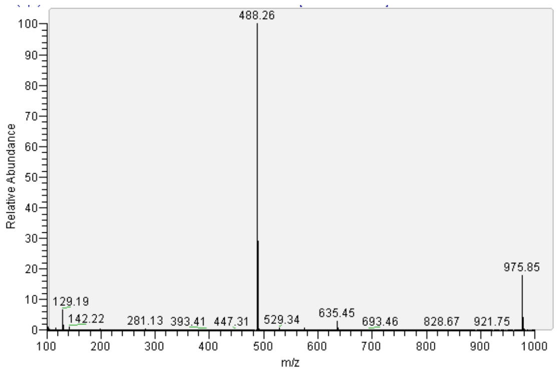

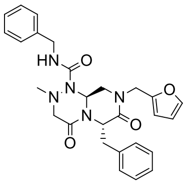

Exact Mass: 487.22
Molecular Weight: 487.55

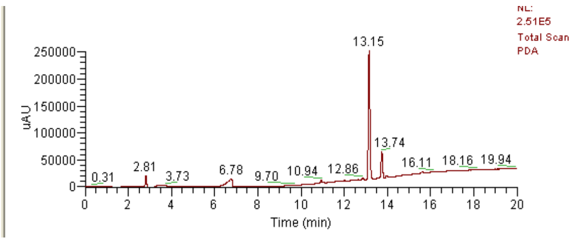

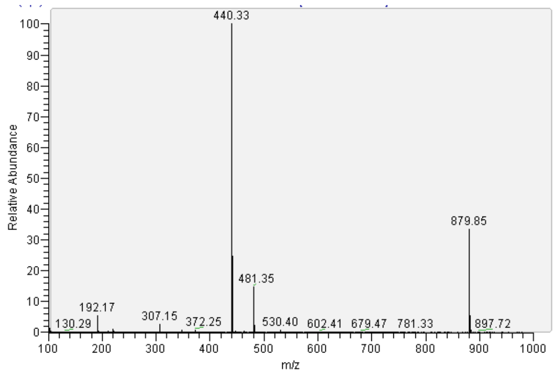

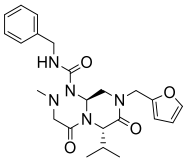

Exact Mass: 439.22
Molecular Weight: 439.51

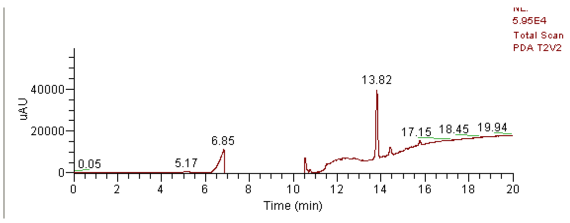

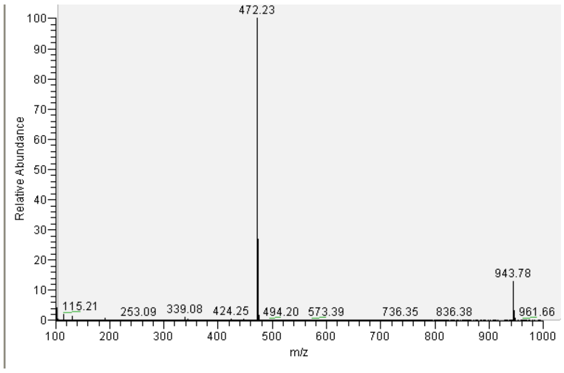

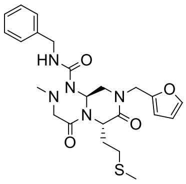

Exact Mass: 471.19
Molecular Weight: 471.57

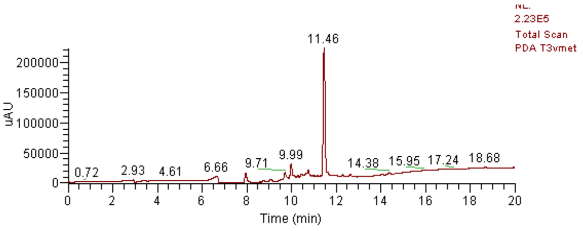

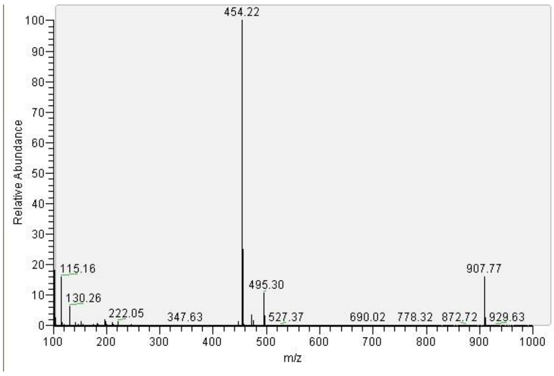

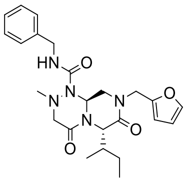

Exact Mass: 453.24
Molecular Weight: 453.53

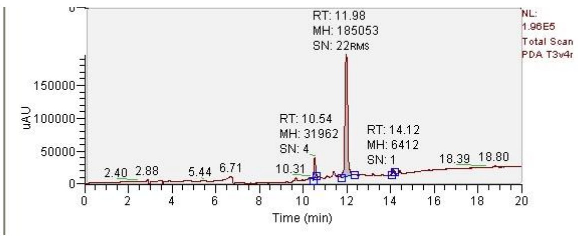

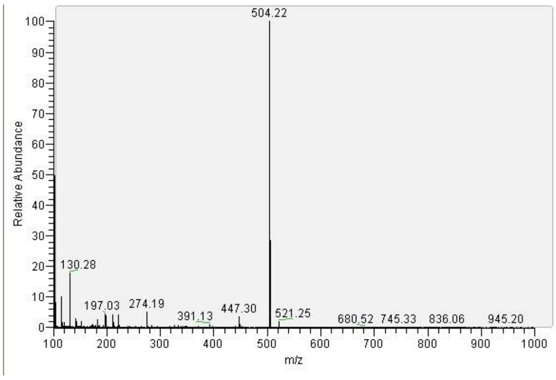

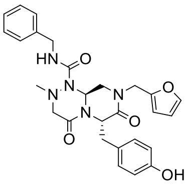

Exact Mass: 503.22
Molecular Weight: 503.55

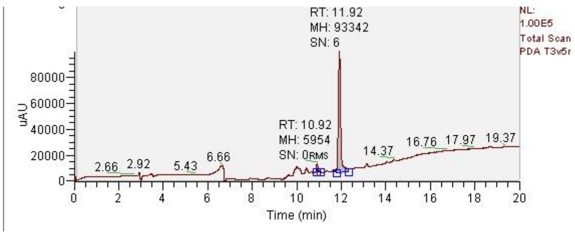

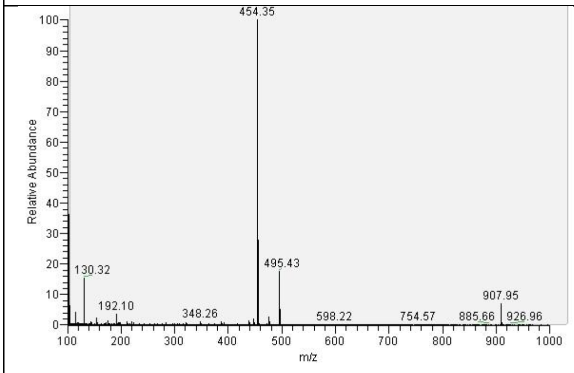

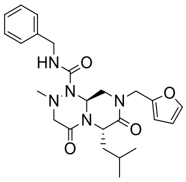

Exact Mass: 453.24
Molecular Weight: 453.53

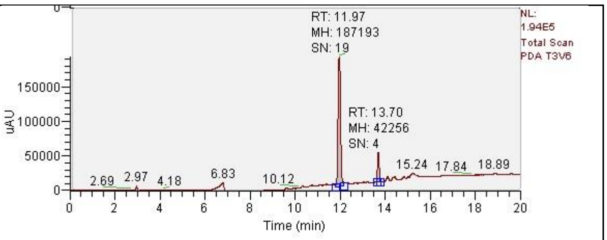

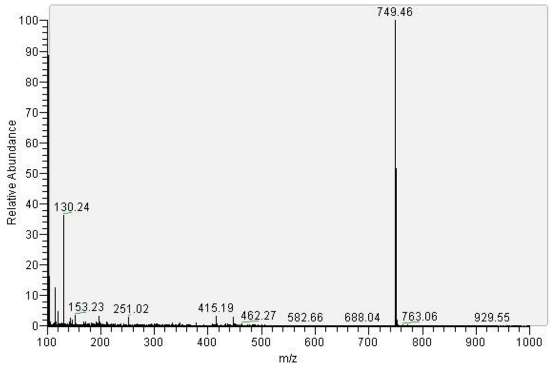

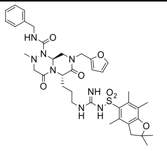

Exact Mass: 748.34
Molecular Weight: 748.89

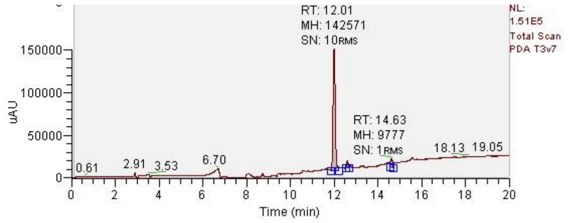

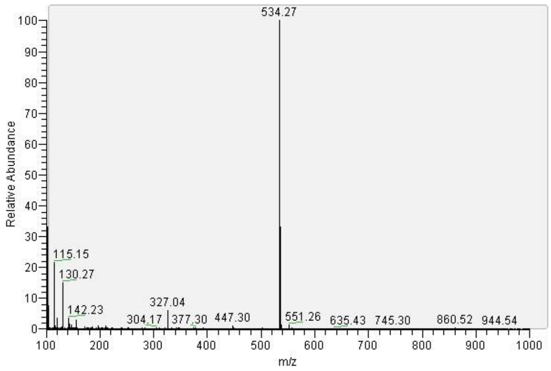

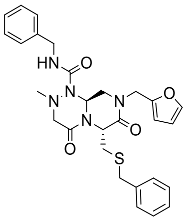

Exact Mass: 533.21
Molecular Weight: 533.64

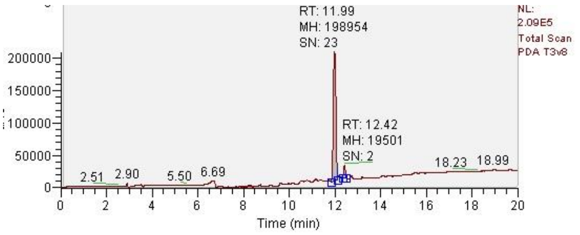

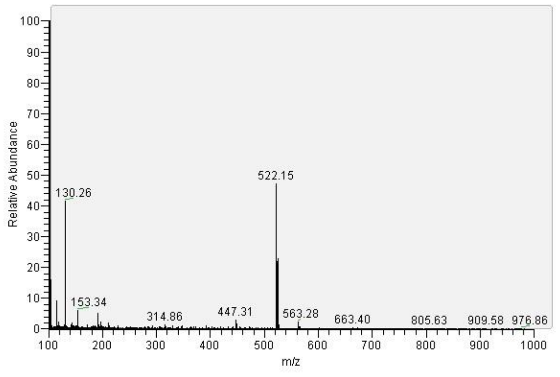	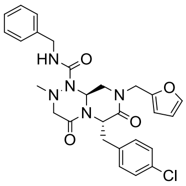<br>Exact Mass: 521.18<br>Molecular Weight: 522.00<br>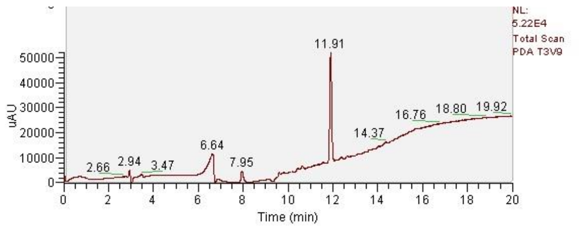
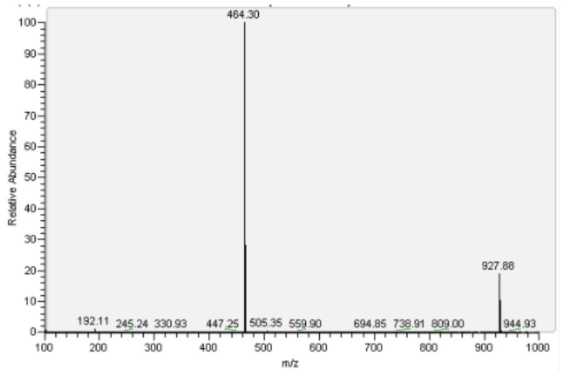	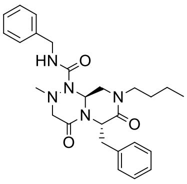<br>Exact Mass: 463.26<br>Molecular Weight: 463.57<br>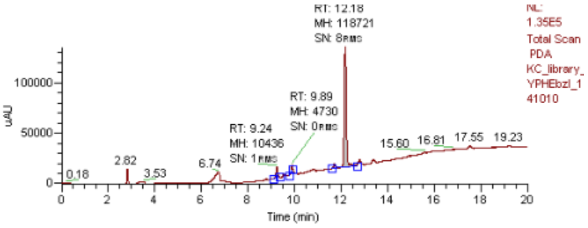
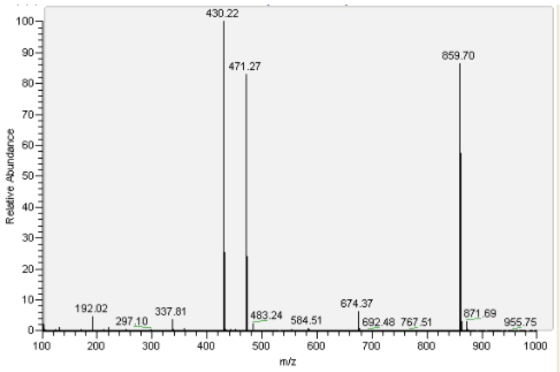	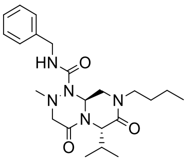<br>Exact Mass: 415.26<br>Molecular Weight: 415.53<br>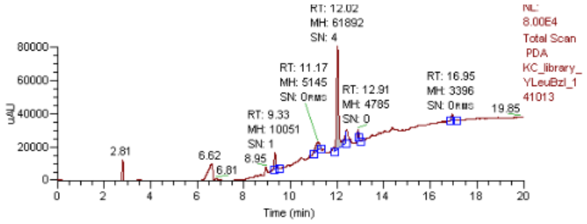

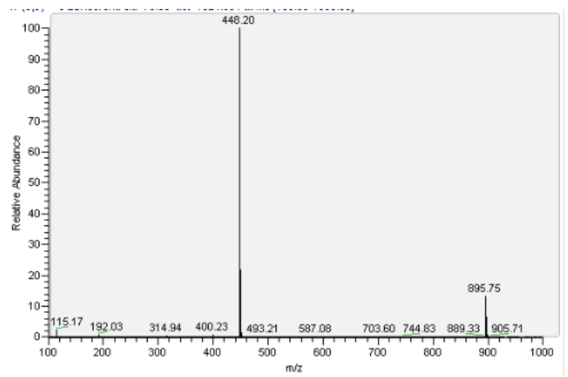

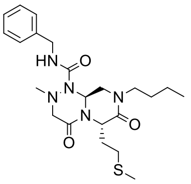

Exact Mass: 447.23
Molecular Weight: 447.59

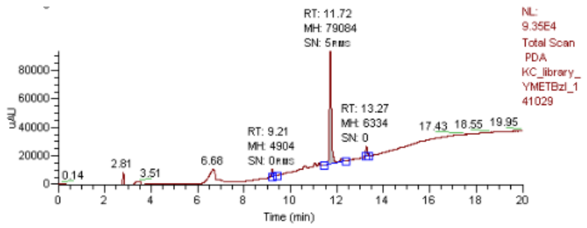

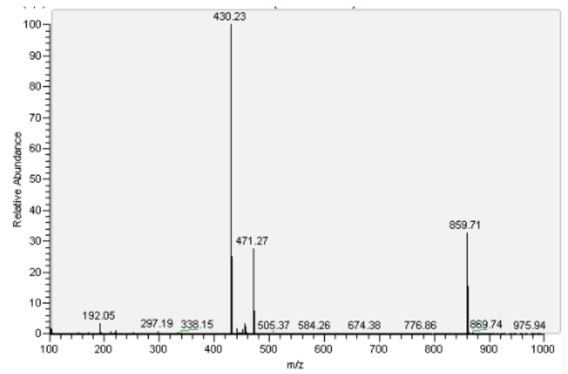

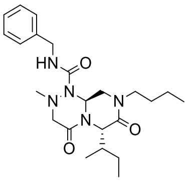

Exact Mass: 429.27
Molecular Weight: 429.56

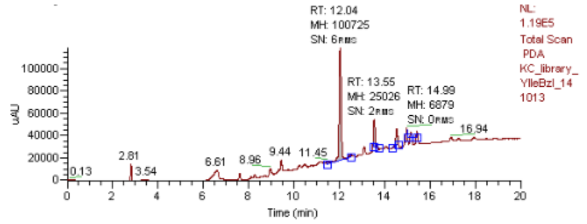

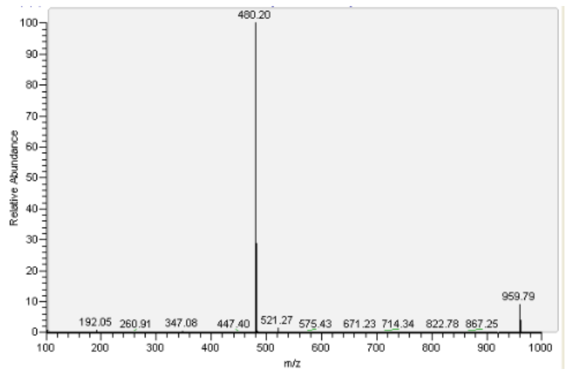

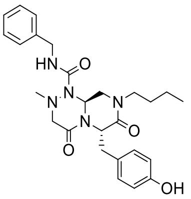

Exact Mass: 479.25
Molecular Weight: 479.57

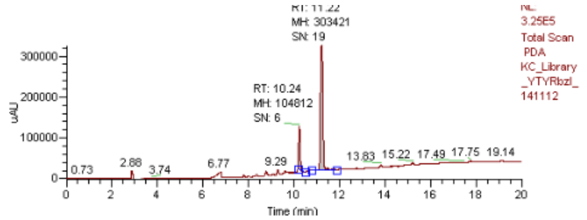

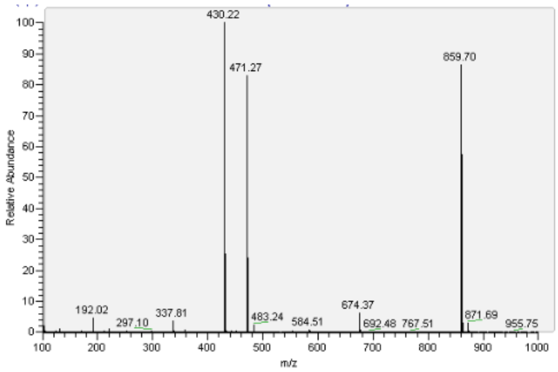

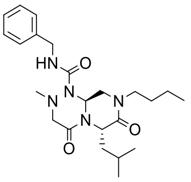

Exact Mass: 429.27
Molecular Weight: 429.56

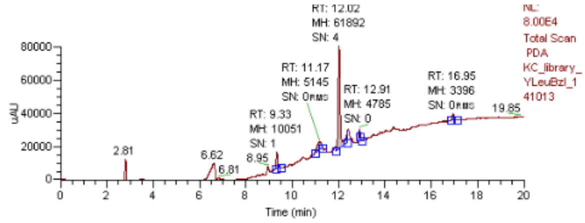

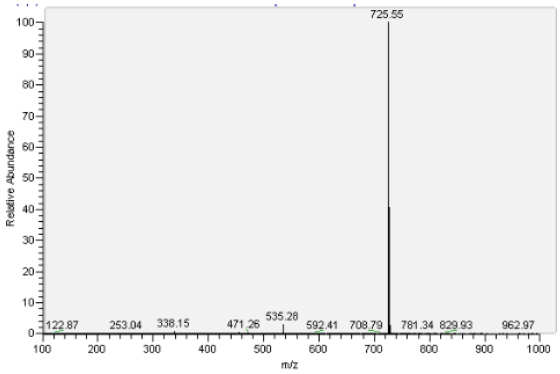

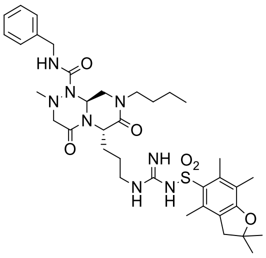

Exact Mass: 724.37
Molecular Weight: 724.91

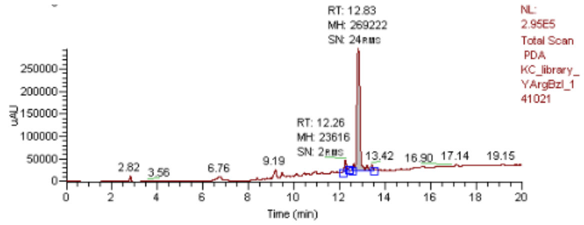

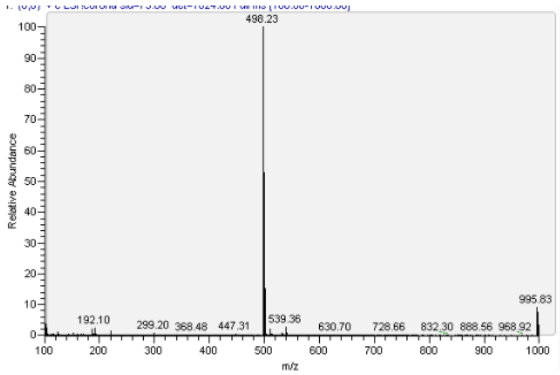

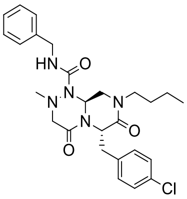

Exact Mass: 497.22
Molecular Weight: 498.02

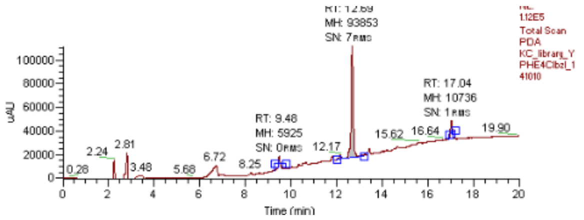

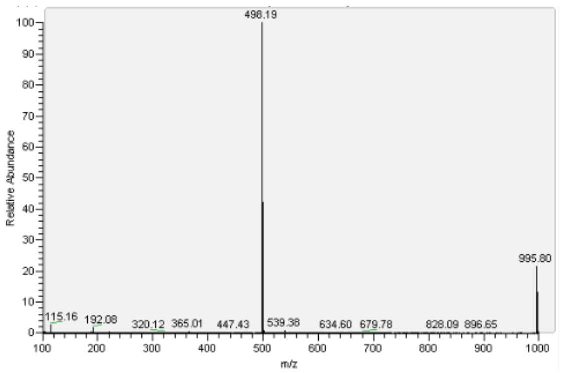

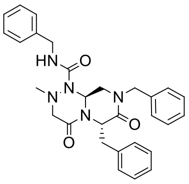

Exact Mass: 497.24
Molecular Weight: 497.59

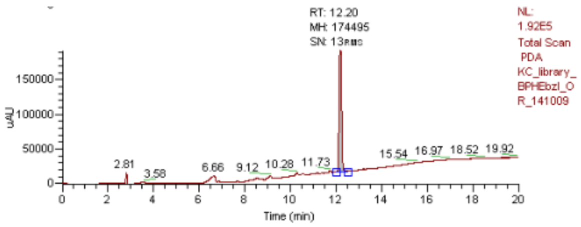

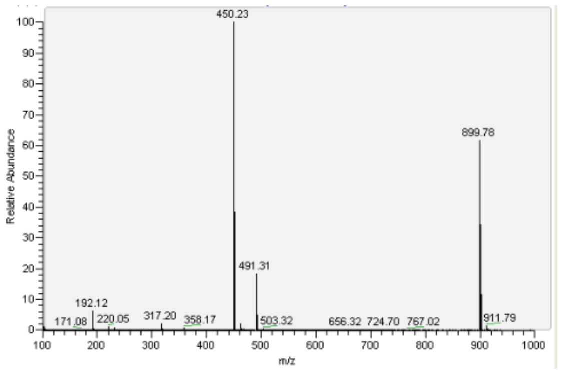

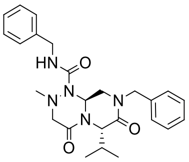

Exact Mass: 449.24
Molecular Weight: 449.55

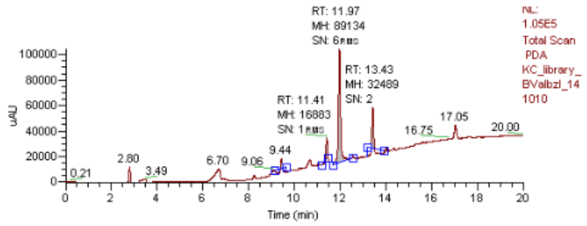

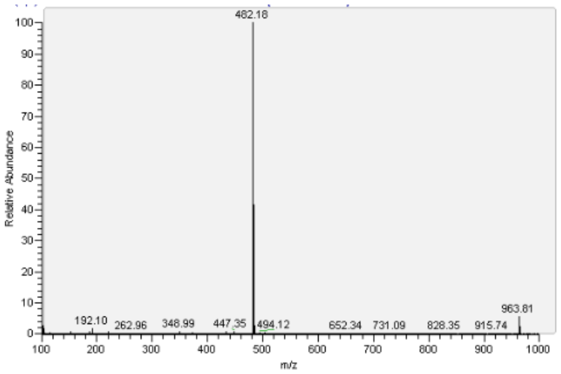

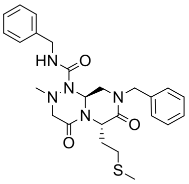

Exact Mass: 481.21
Molecular Weight: 481.61

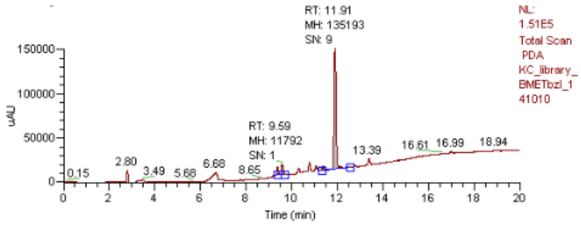

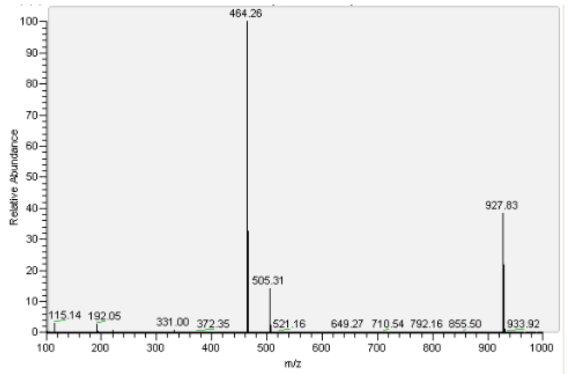

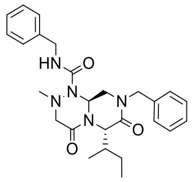

Exact Mass: 463.26
Molecular Weight: 463.57

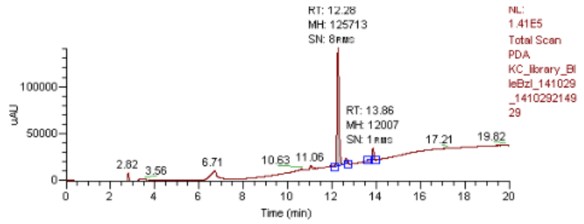

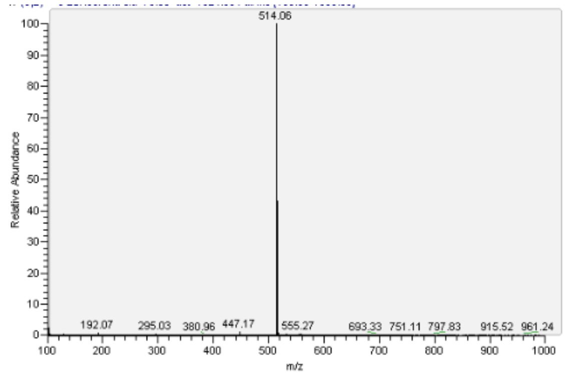

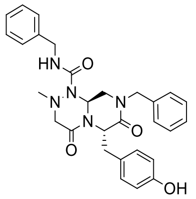

Exact Mass: 513.24
Molecular Weight: 513.59

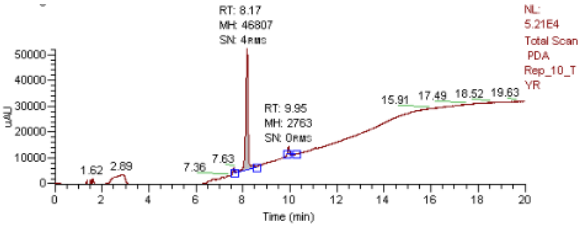

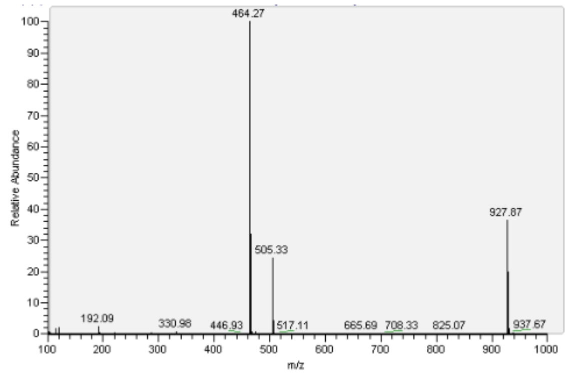

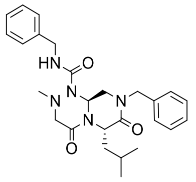

Exact Mass: 463.26
Molecular Weight: 463.57

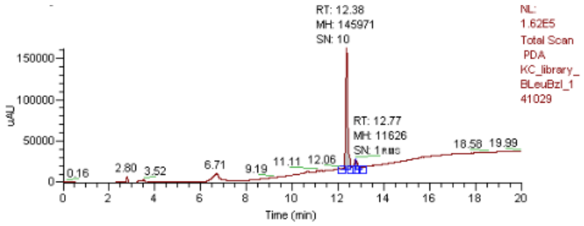

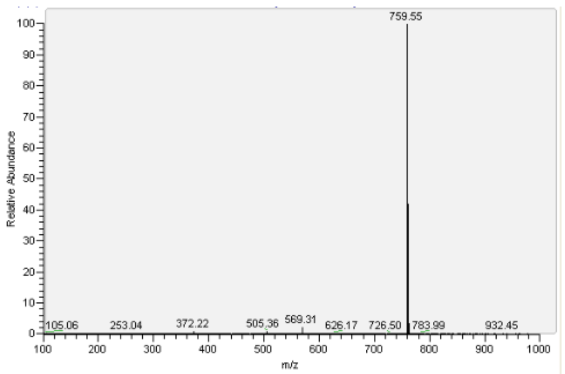

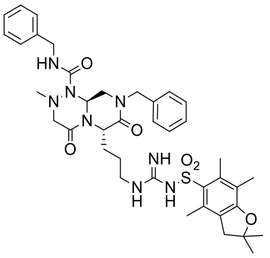

Exact Mass: 758.36
Molecular Weight: 758.93

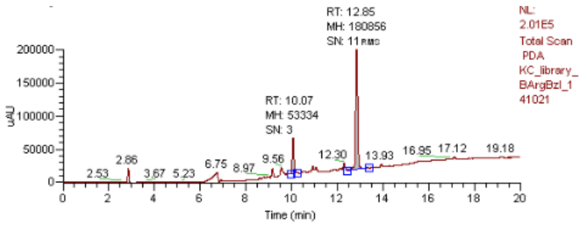

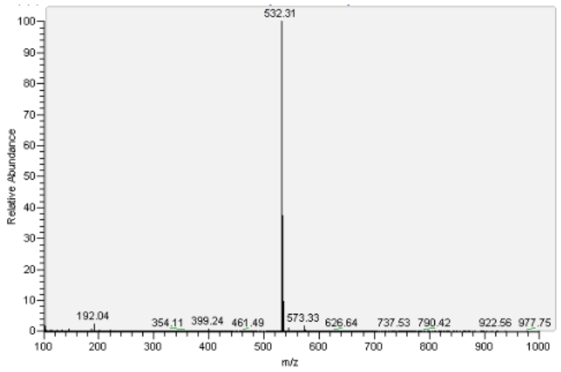

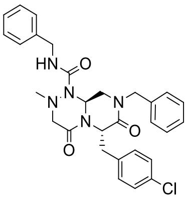

Exact Mass: 531.20
Molecular Weight: 532.03

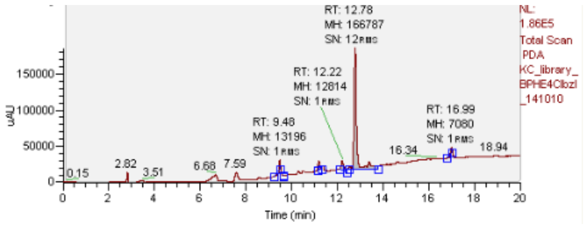

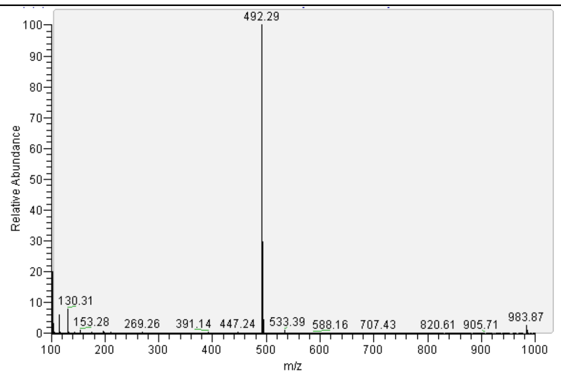

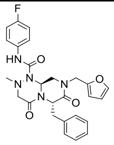

Exact Mass: 491.20
Molecular Weight: 491.51

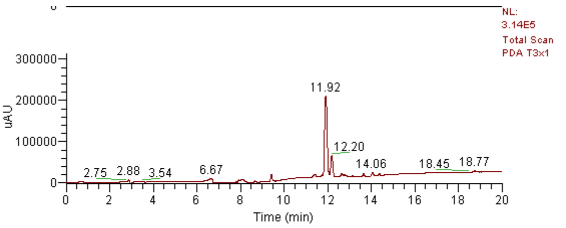

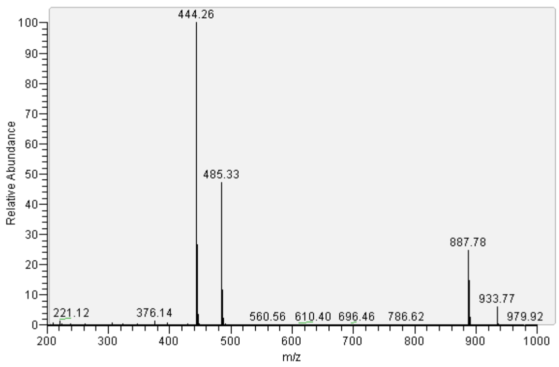

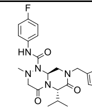

Exact Mass: 443.20
Molecular Weight: 443.47

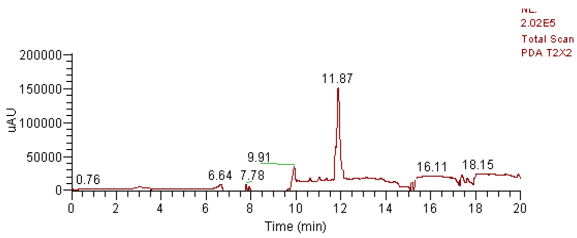

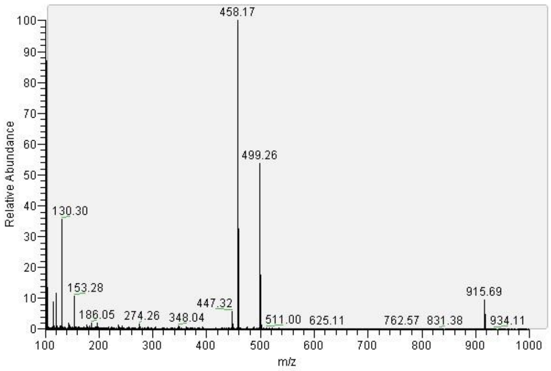

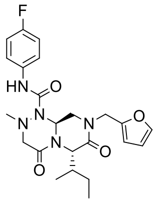

Exact Mass: 457.21
Molecular Weight: 457.50

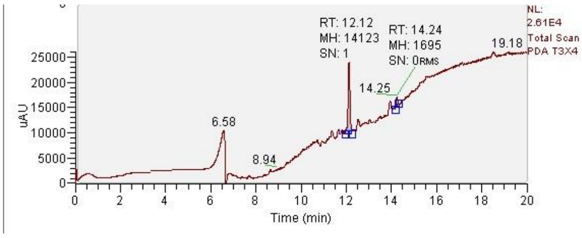

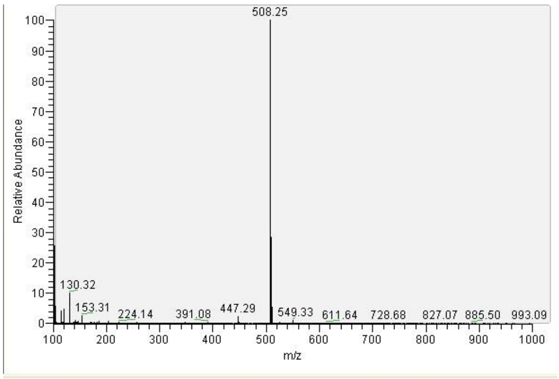

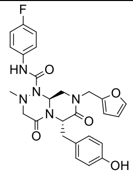

Exact Mass: 507.19
Molecular Weight: 507.51

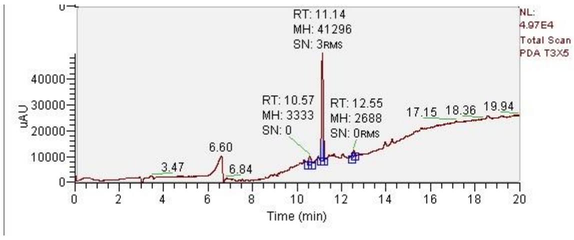

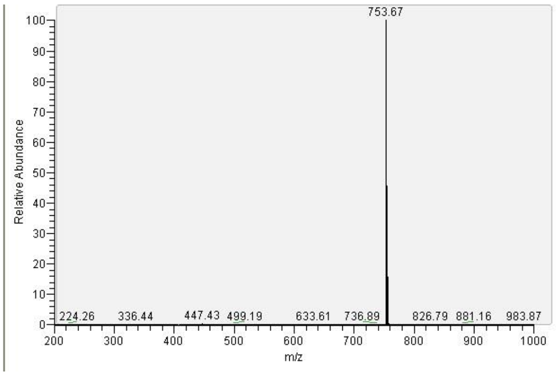

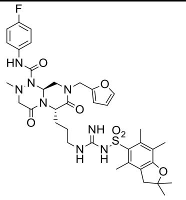

Exact Mass: 752.31
Molecular Weight: 752.86

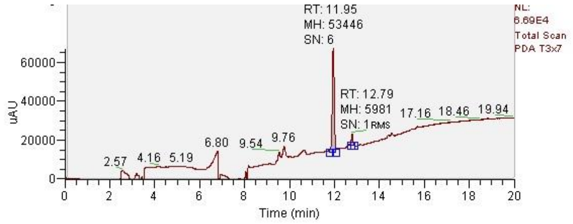

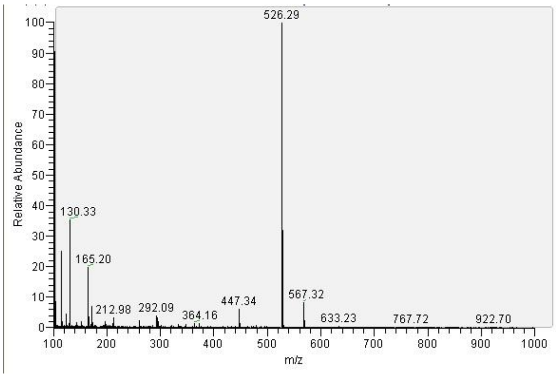

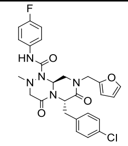

Exact Mass: 525.16
Molecular Weight: 525.96

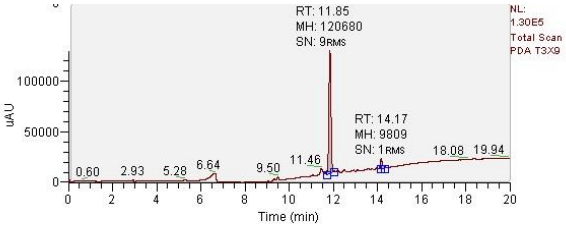

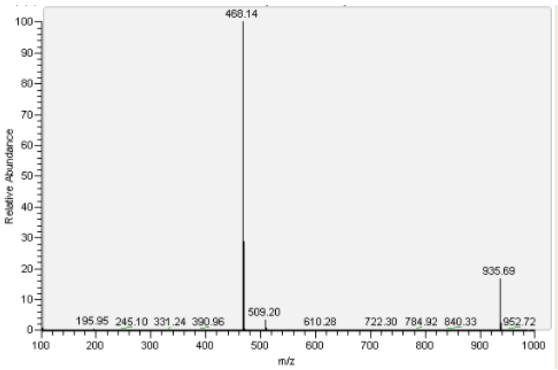

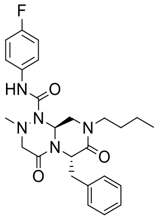

Exact Mass: 467.23
Molecular Weight: 467.54

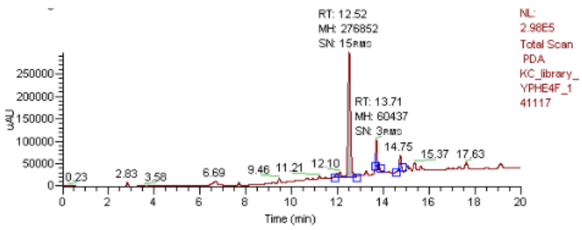

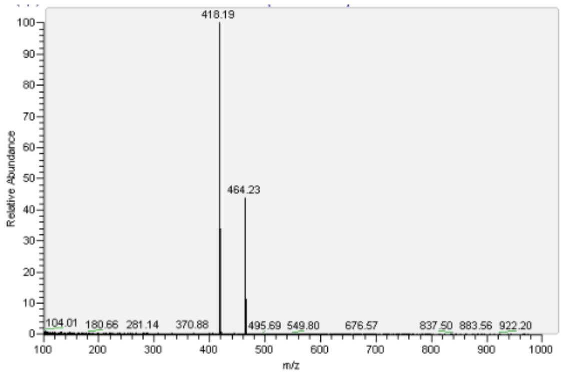

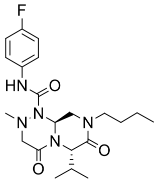

Exact Mass: 419.23
Molecular Weight: 419.49

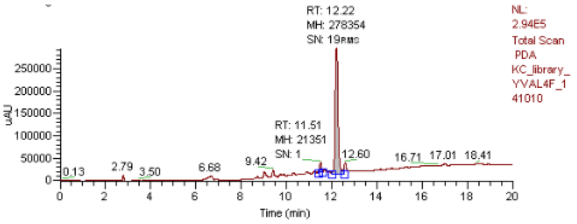

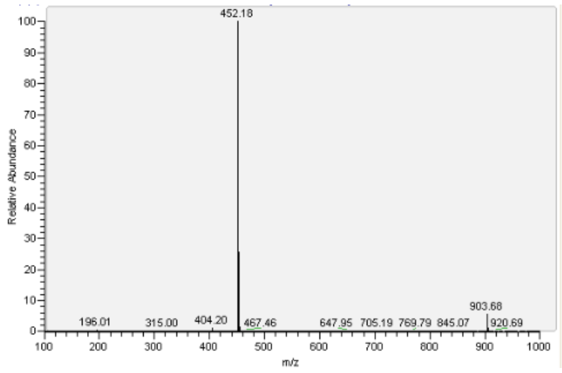

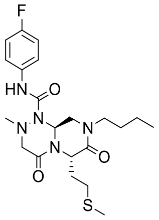

Exact Mass: 451.21
Molecular Weight: 451.56

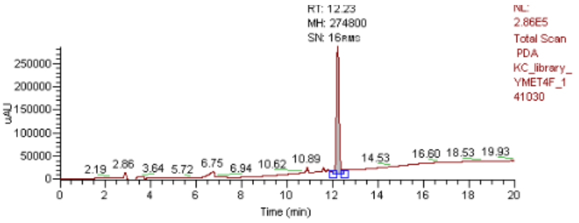

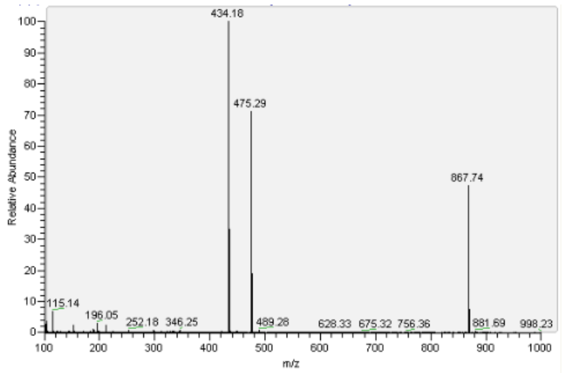

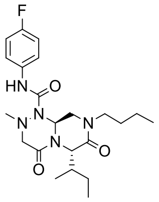

Exact Mass: 433.25
Molecular Weight: 433.52

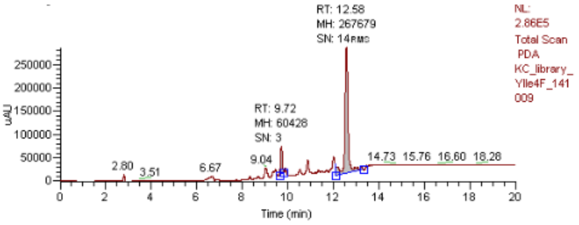

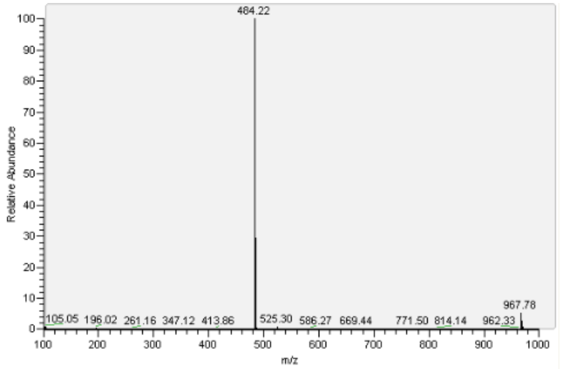

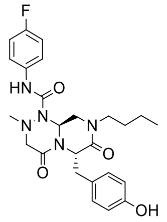

Exact Mass: 483.23
Molecular Weight: 483.54

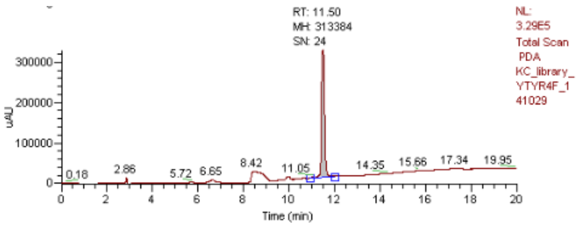

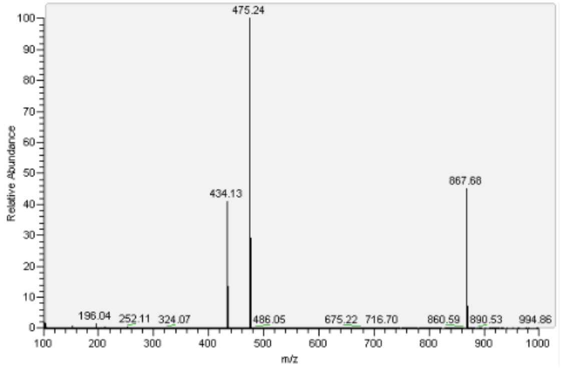

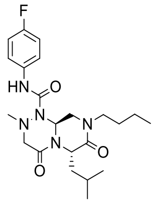

Exact Mass: 433.25
Molecular Weight:
433.52

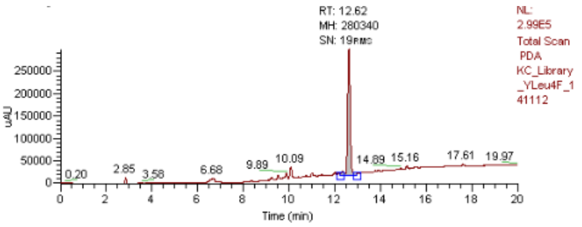

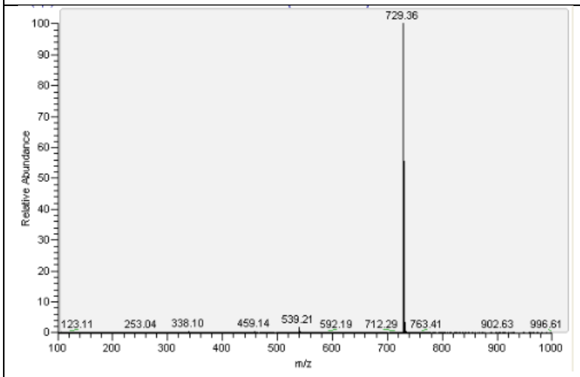

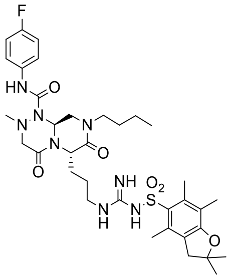

Exact Mass: 728.35
Molecular Weight: 728.88

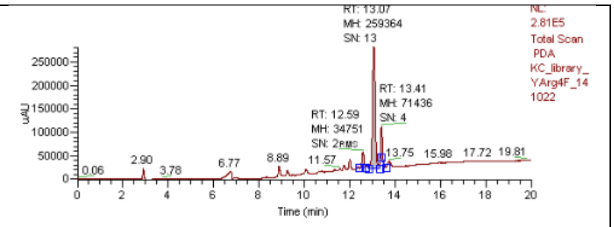

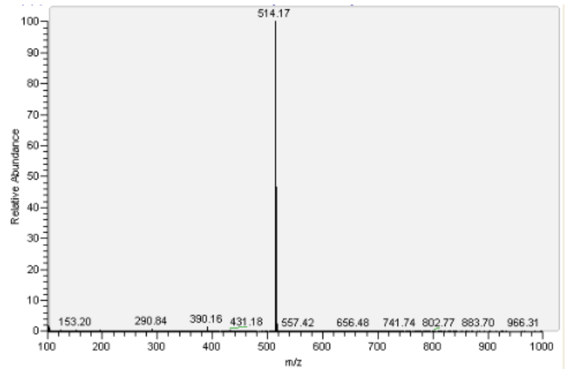

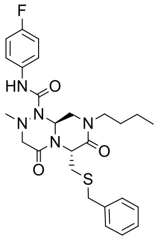

Exact Mass: 513.22
Molecular Weight: 513.63

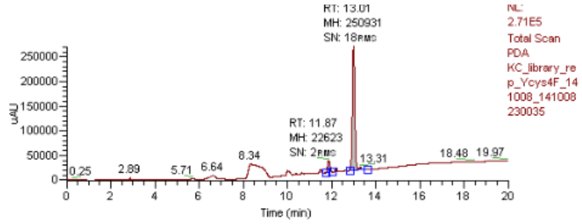

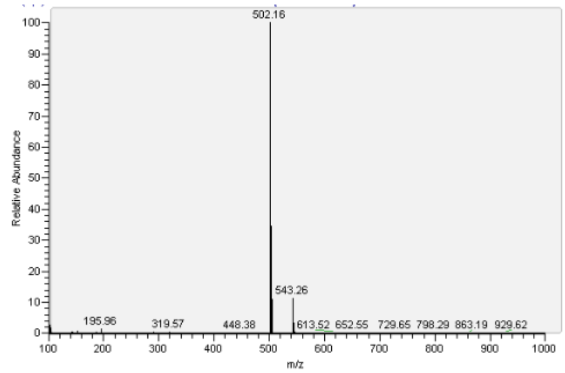

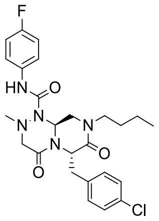

Exact Mass: 501.19
Molecular Weight: 501.98

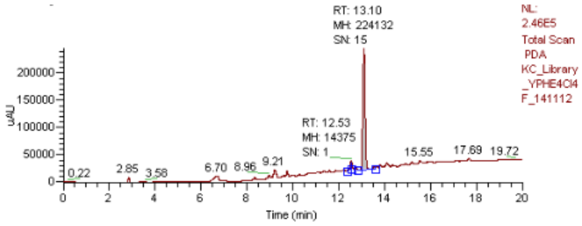

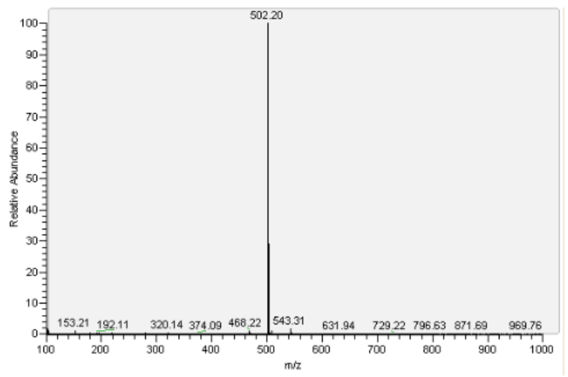

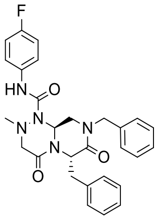

Exact Mass: 501.22
Molecular Weight: 501.55

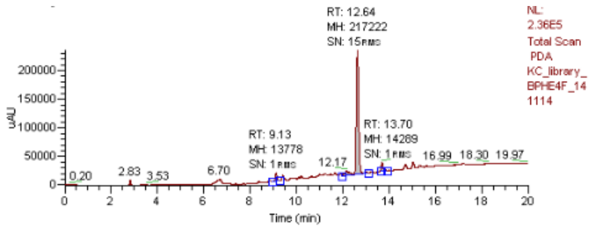

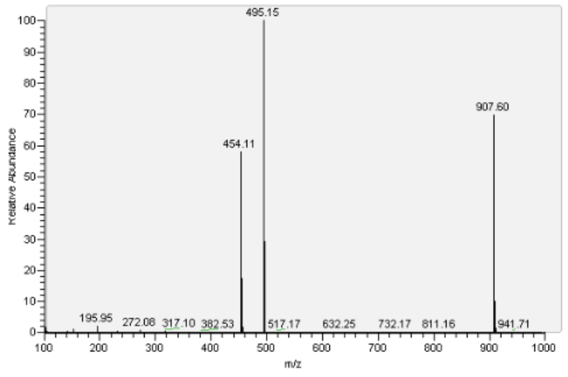

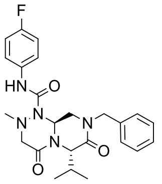

Exact Mass: 453.22
Molecular Weight: 453.51

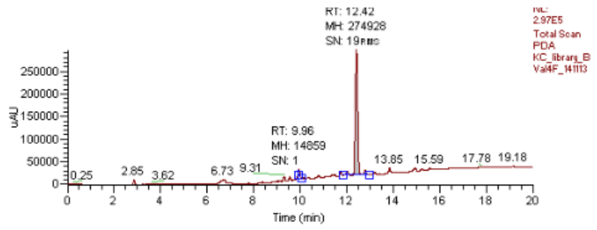

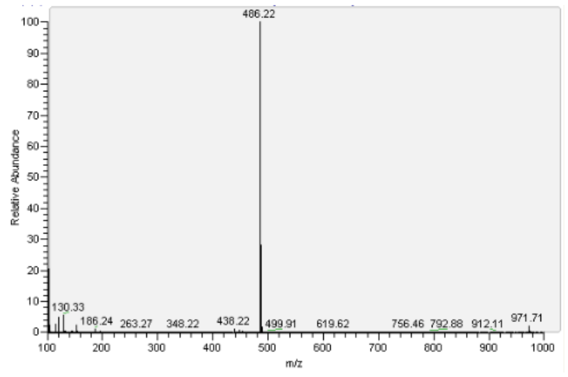

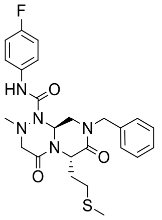

Exact Mass: 485.19
Molecular Weight: 485.57

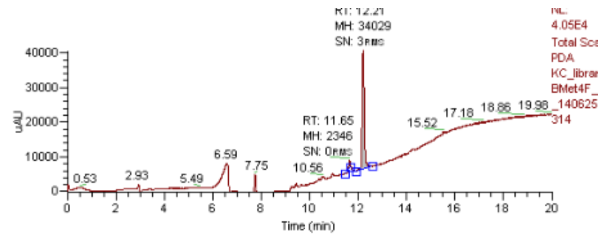

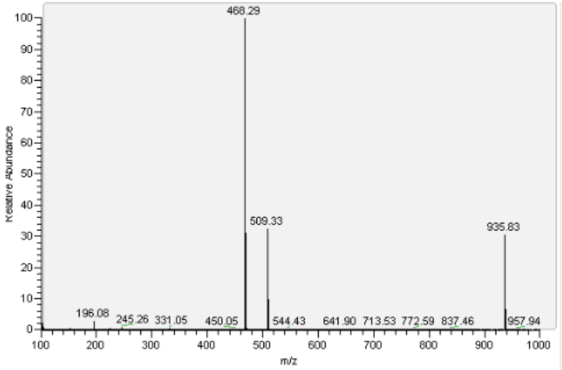

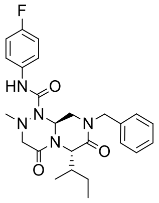

Exact Mass: 467.23
Molecular Weight: 467.54

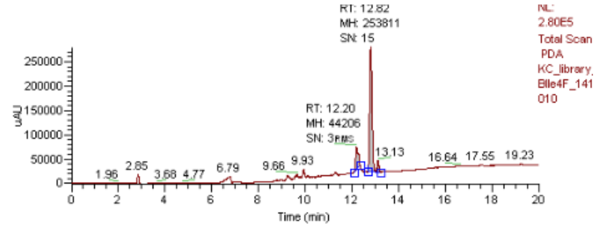

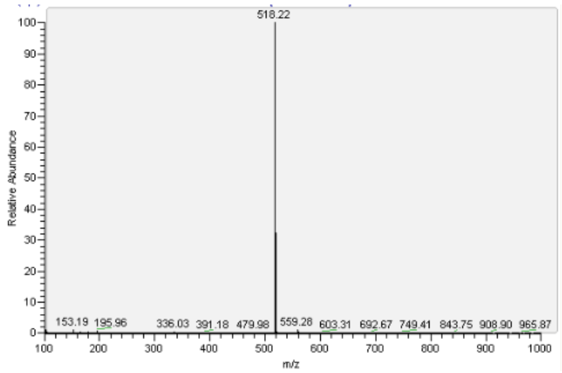

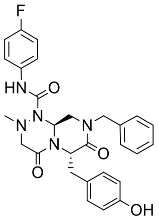

Exact Mass: 517.21
Molecular Weight: 517.55

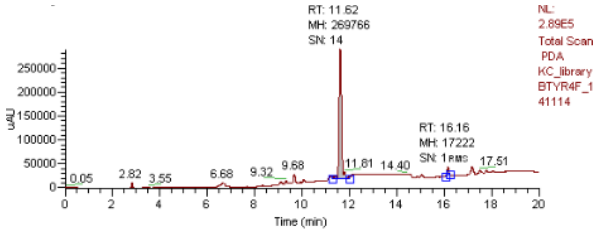

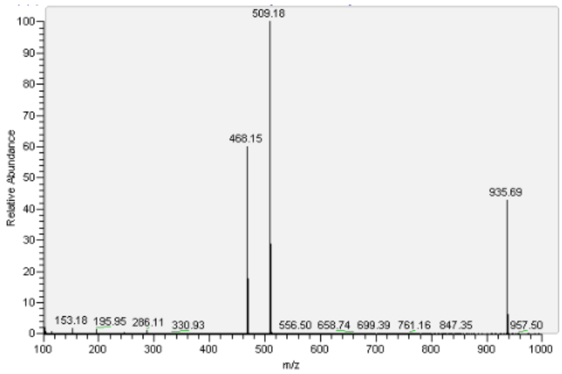

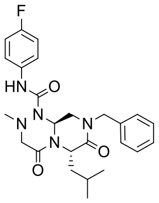

Exact Mass: 467.23
Molecular Weight: 467.54

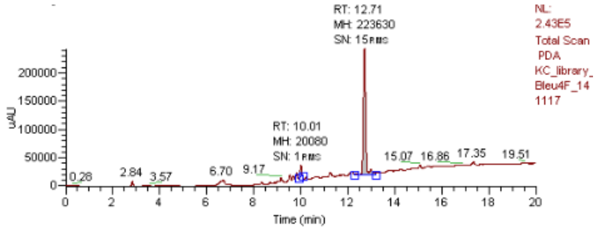

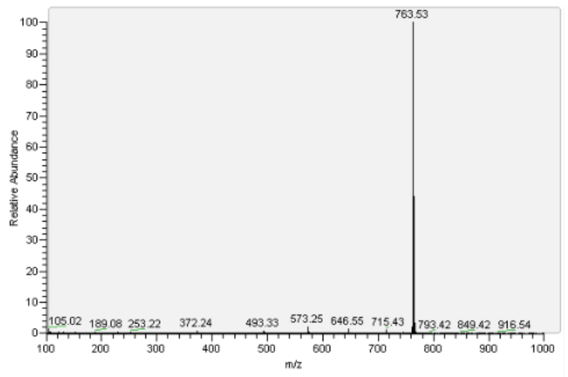

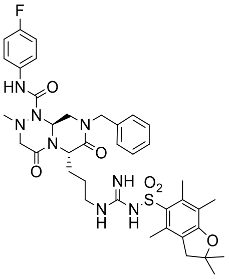

Exact Mass: 762.33
Molecular Weight: 762.89

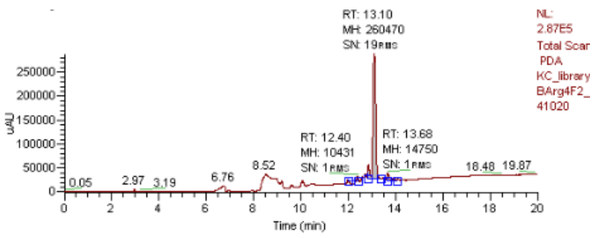

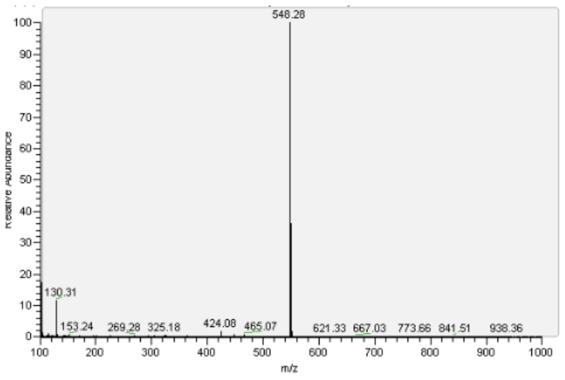

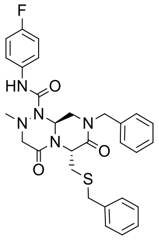

Exact Mass: 547.21
Molecular Weight: 547.64

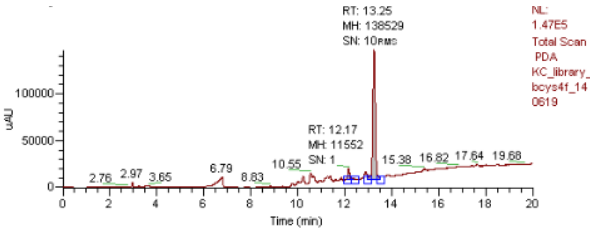

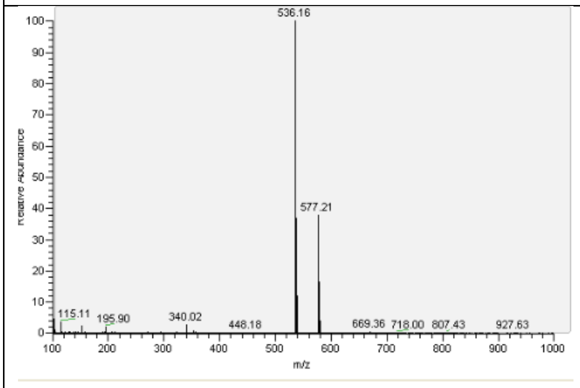

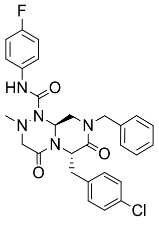

Exact Mass: 535.18
Molecular Weight: 536.00

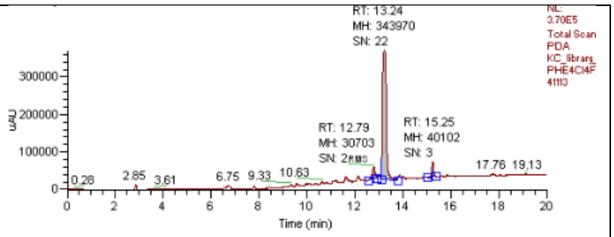

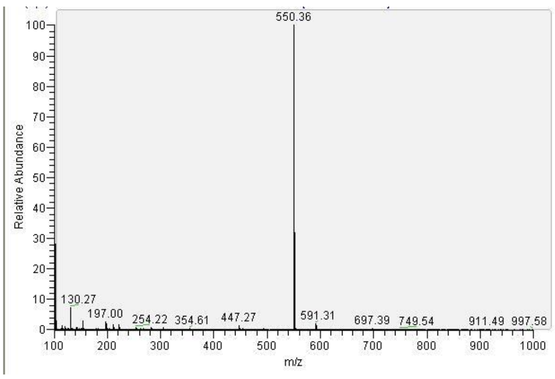

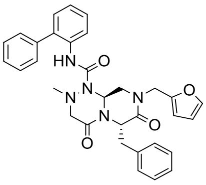

Exact Mass: 549.24
Molecular Weight: 549.62

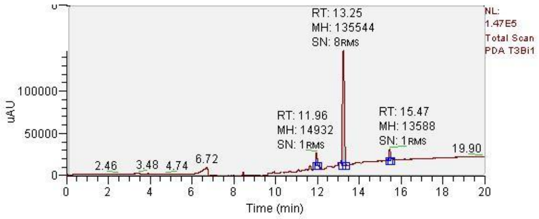

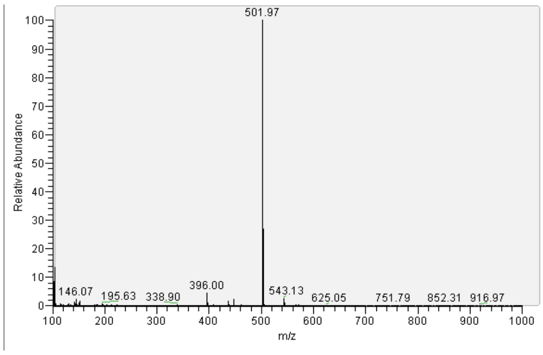

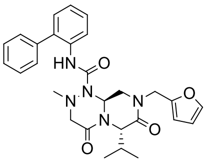

Exact Mass: 501.24
Molecular Weight: 501.58

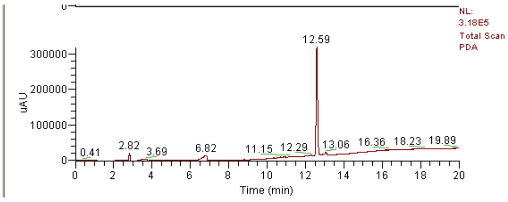

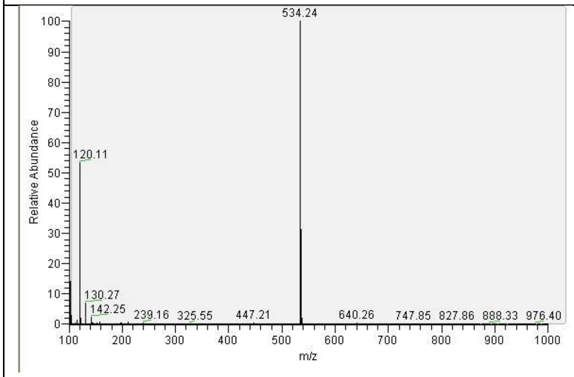

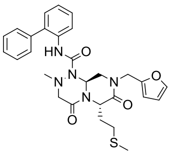

Exact Mass: 533.21
Molecular Weight: 533.64

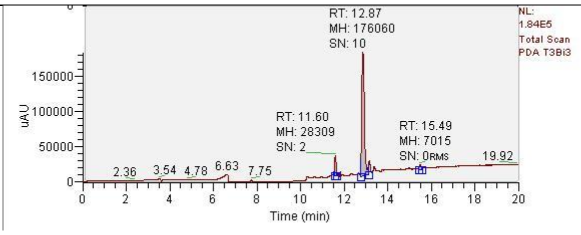

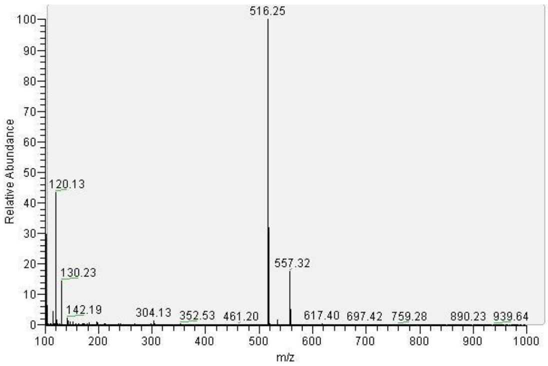

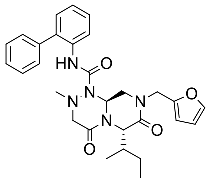

Exact Mass: 515.25
Molecular Weight: 515.60

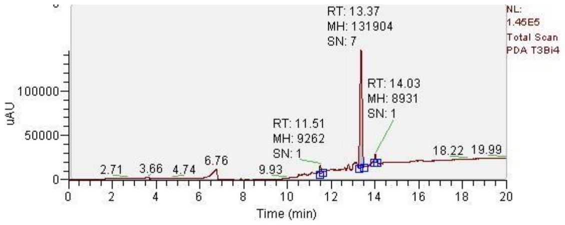

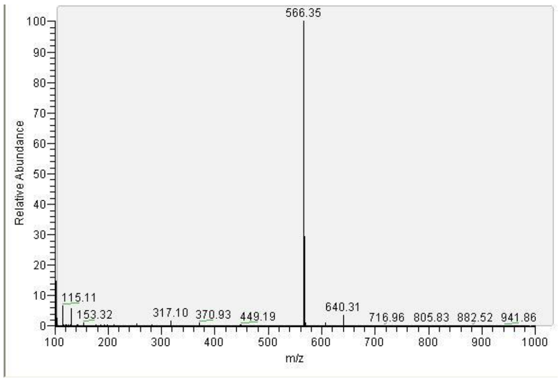

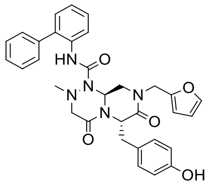

Exact Mass: 565.23
Molecular Weight: 565.62

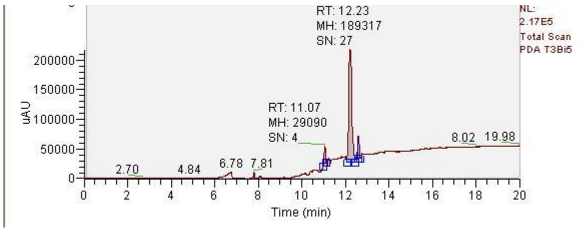

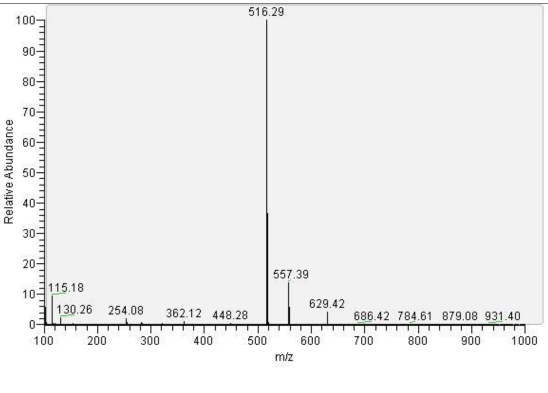

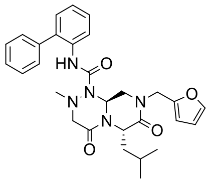

Exact Mass: 515.25
Molecular Weight: 515.60

	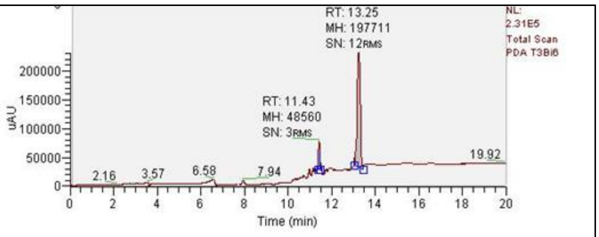
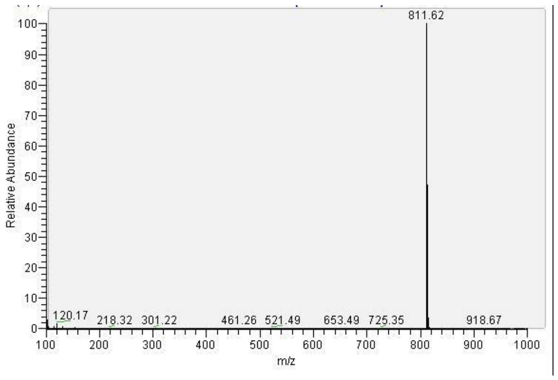	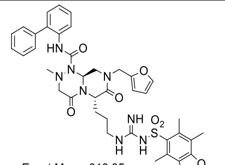 Exact Mass: 810.35<br>Molecular Weight: 810.96<br>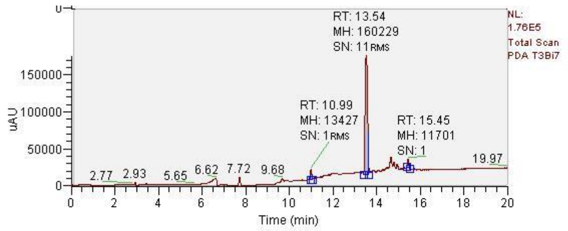
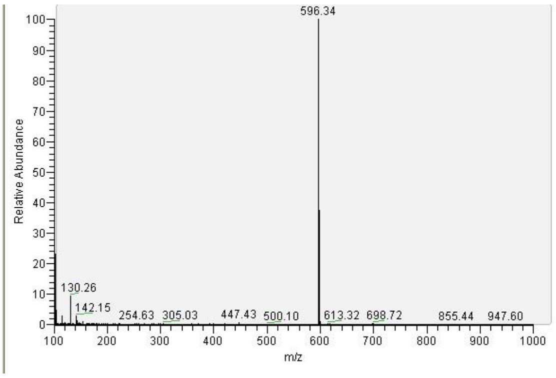	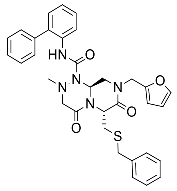 Exact Mass: 595.23<br>Molecular Weight: 595.71<br>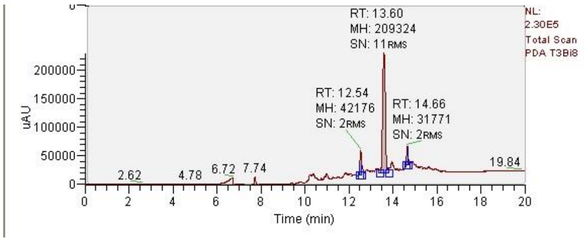
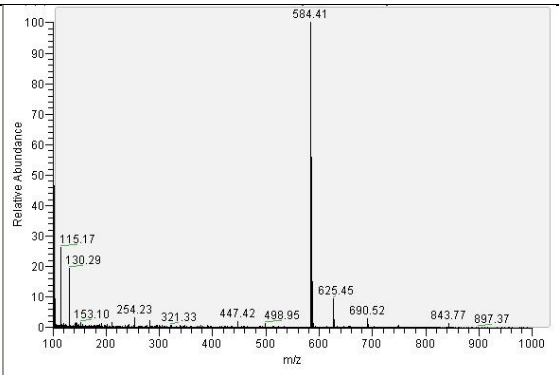	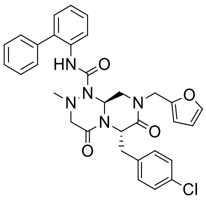 Exact Mass: 583.20<br>Molecular Weight: 584.06

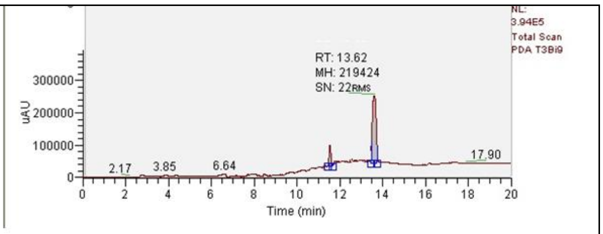

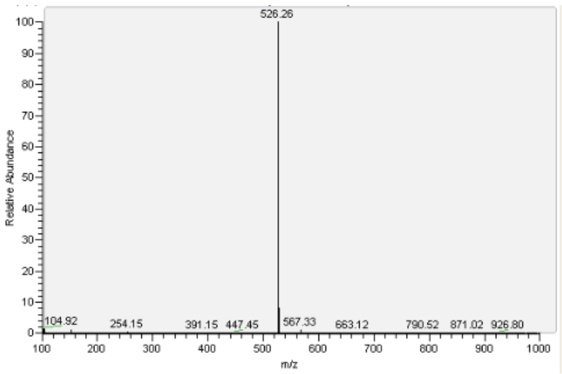

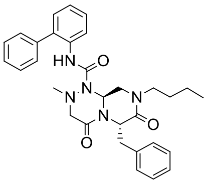

Exact Mass: 525.27
Molecular Weight: 525.64

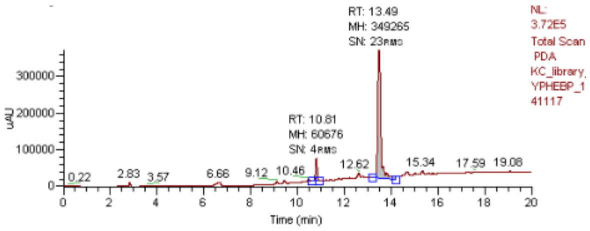

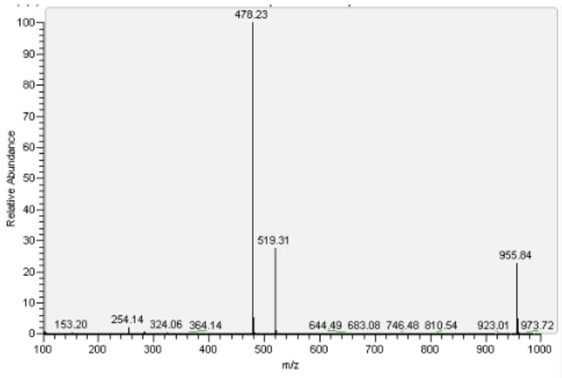

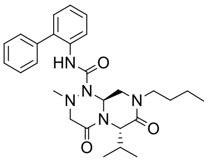

Exact Mass: 477.27
Molecular Weight: 477.60

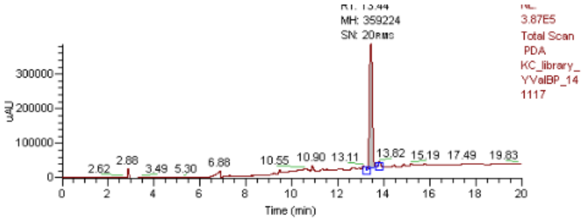

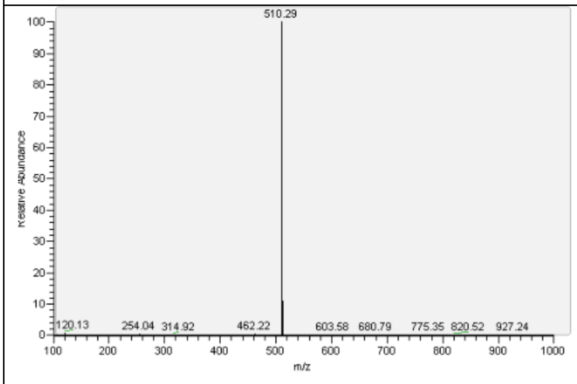

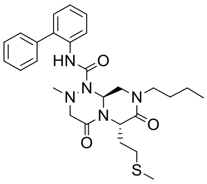

Exact Mass: 509.25
Molecular Weight: 509.66

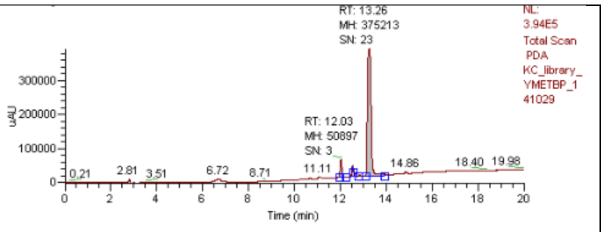

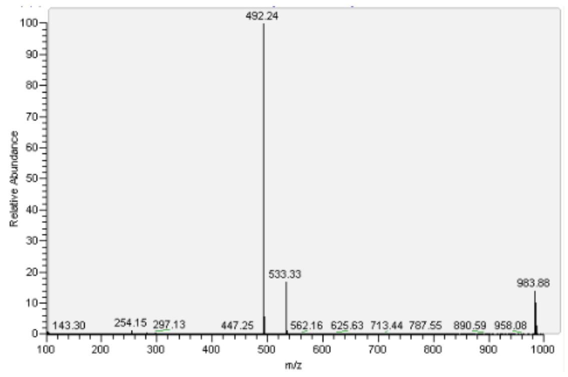

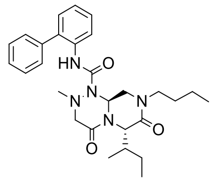

Exact Mass: 491.29
Molecular Weight: 491.63

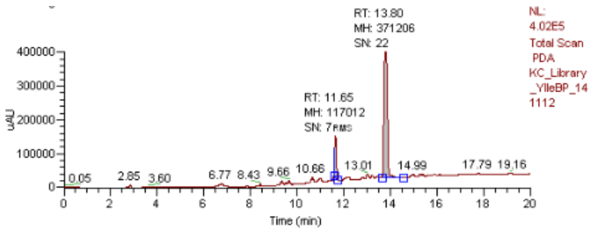

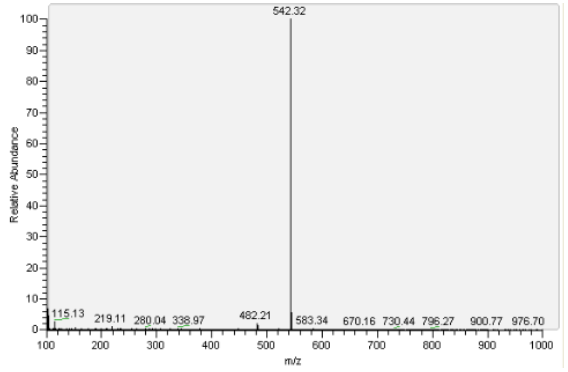

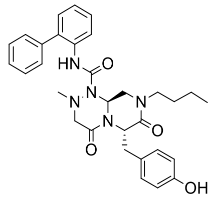

Exact Mass: 541.27
Molecular Weight: 541.64

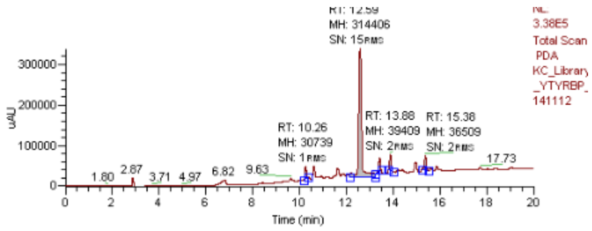

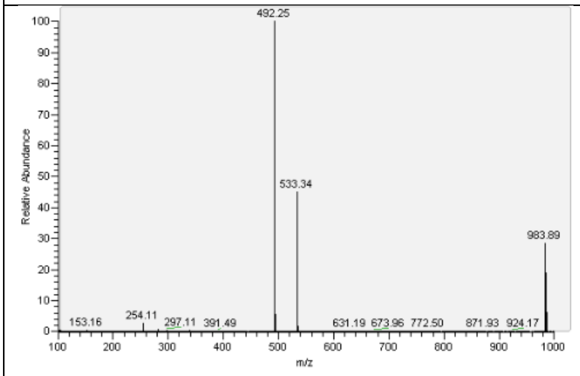

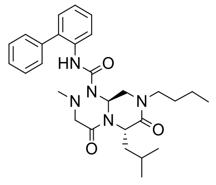

Exact Mass: 491.29
Molecular Weight: 491.63

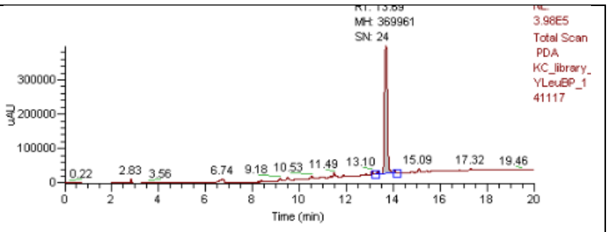

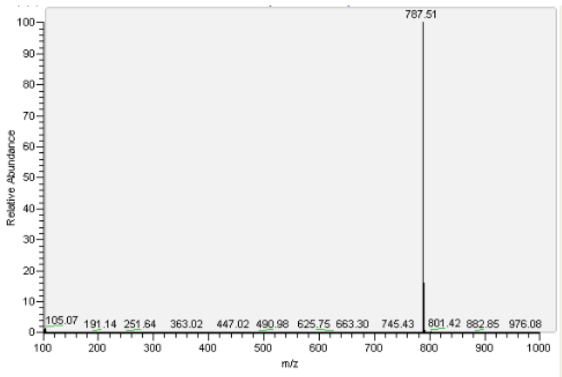

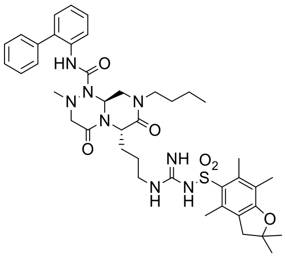

Exact Mass: 786.39
Molecular Weight: 786.98

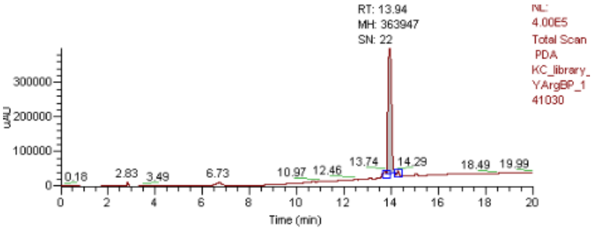

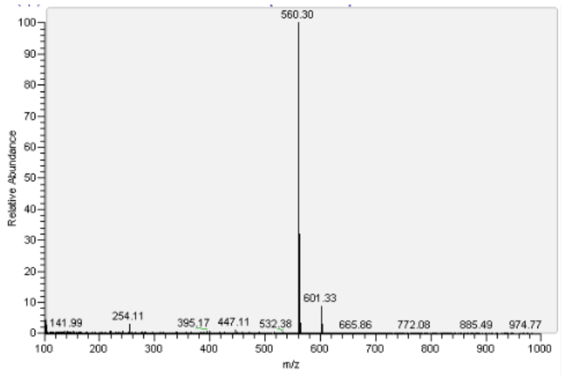

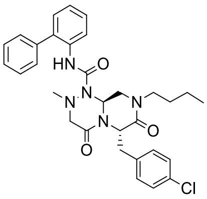

Exact Mass: 559.24
Molecular Weight: 560.09

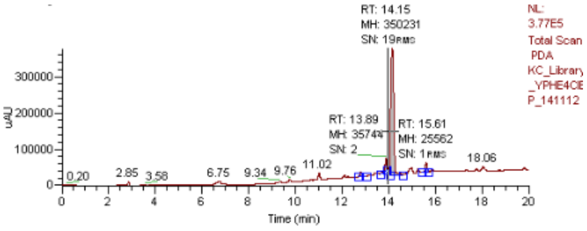

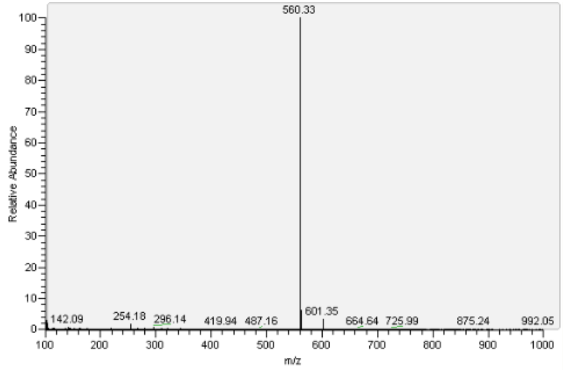

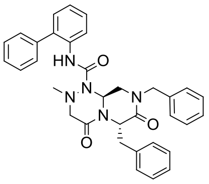

Exact Mass: 559.26
Molecular Weight: 559.66

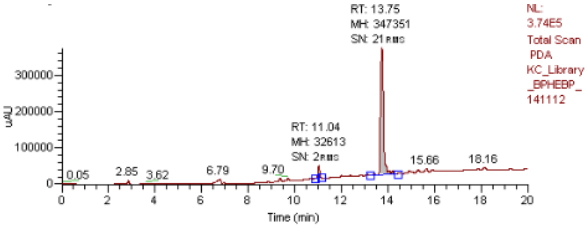

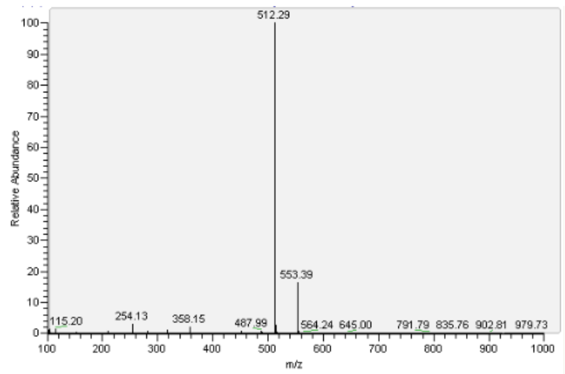

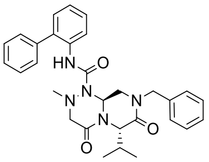

Exact Mass: 511.26
Molecular Weight: 511.61

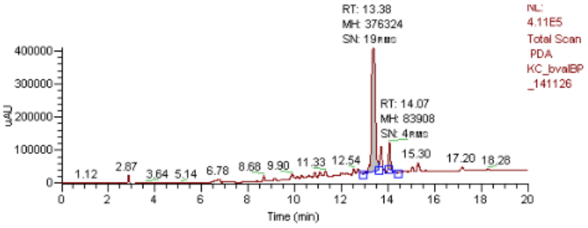

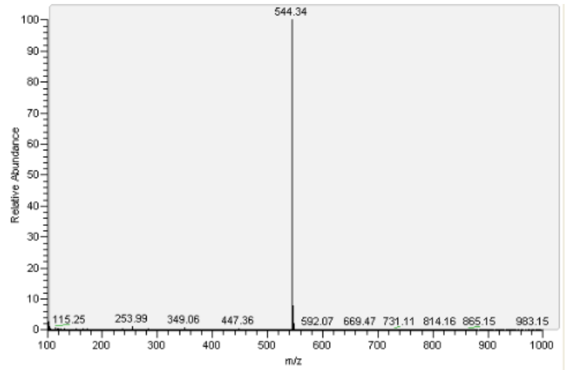

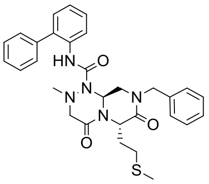

Exact Mass: 543.23
Molecular Weight: 543.68

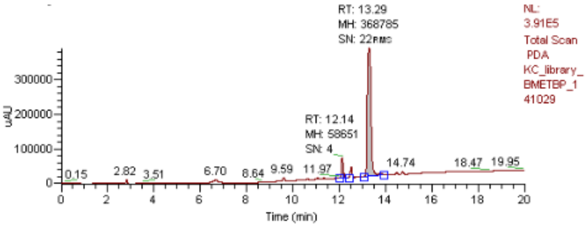

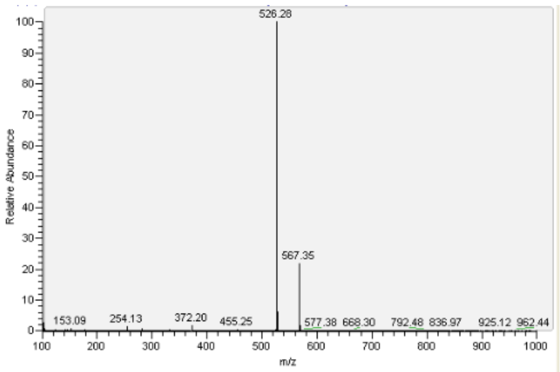

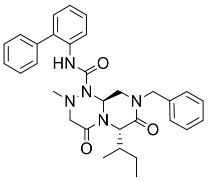

Exact Mass: 525.27
Molecular Weight: 525.64

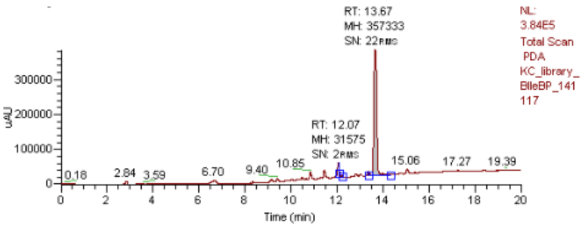

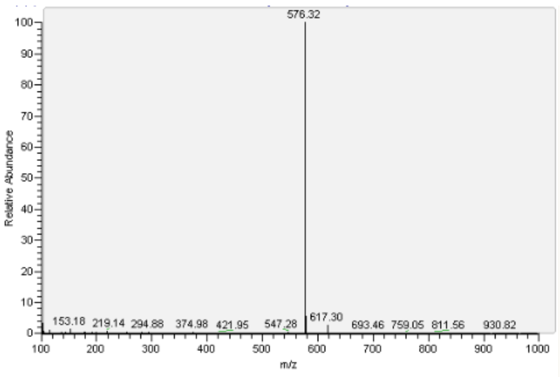

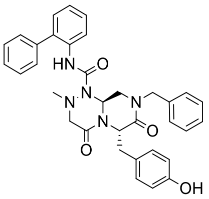

Exact Mass: 575.25
Molecular Weight: 575.66

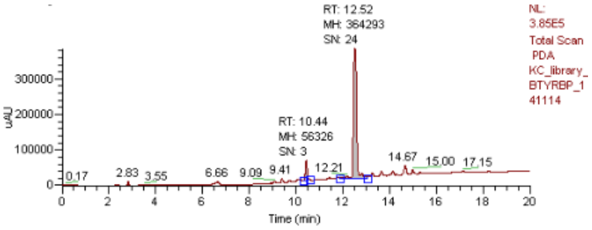

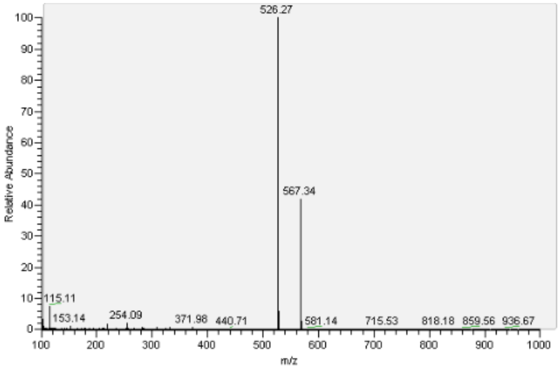

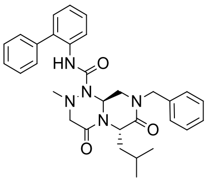

Exact Mass: 525.27
Molecular Weight: 525.64

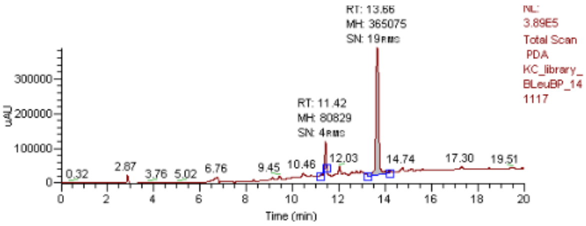

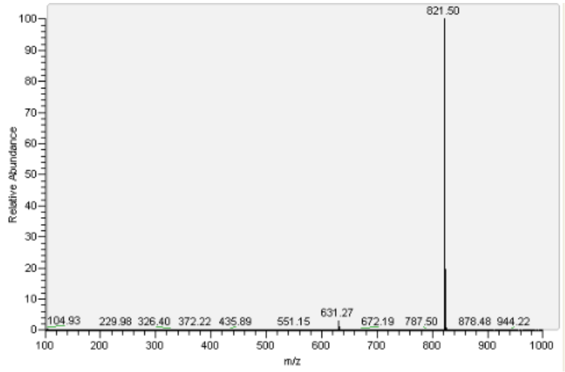

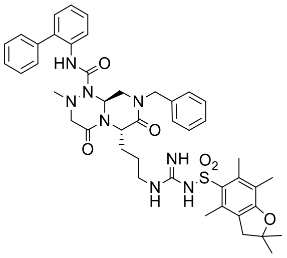

Exact Mass: 820.37
Molecular Weight: 821.00

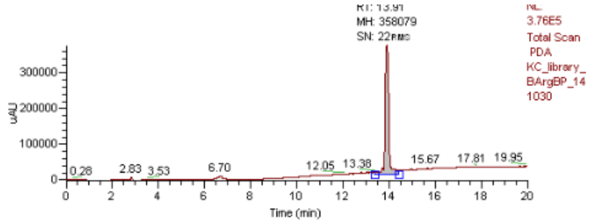

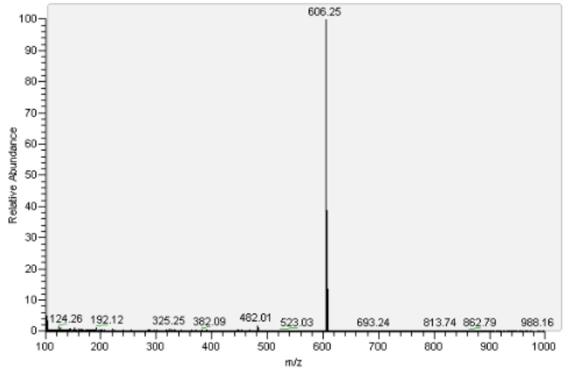

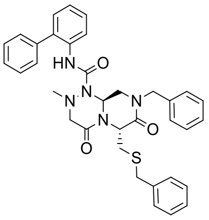

Exact Mass: 605.25
Molecular Weight: 605.75

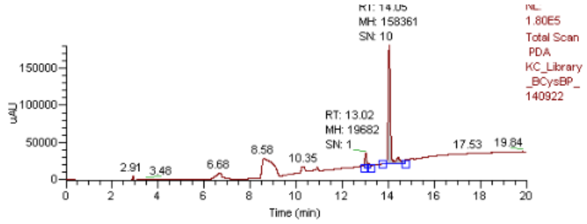

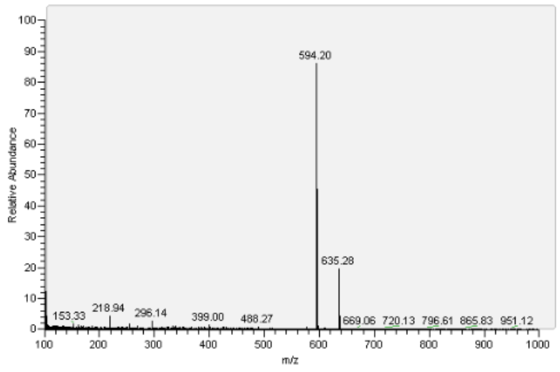

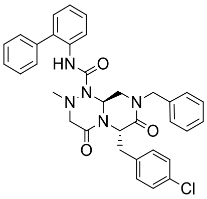

Exact Mass: 593.22
Molecular Weight: 594.10

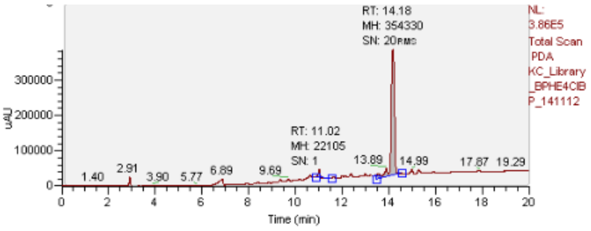

VIII. FRET Imaging

FIG S3. FRET Imaging. Captured fluorescent images within HEK293T cells expressing both LRS-CFP and RagD-YFP (first low), LRS-CFP (second low) and RagD-YFP (third low). Images captured with CFP/CFP (first column), YFP/YFP (second column) and CFP/YFP channel filter sets are shown (excitation/emission). Scale bar, 20 μm.

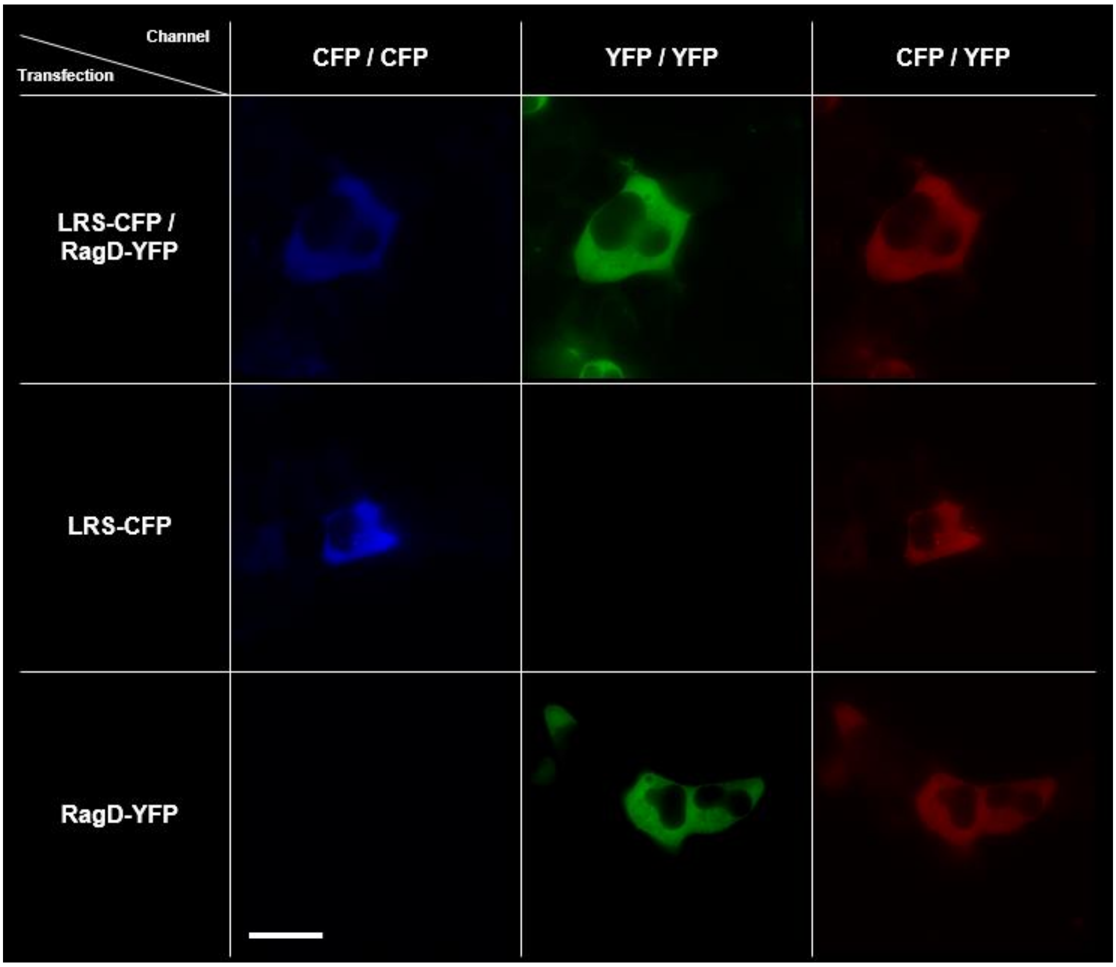

IX. Experimental procedure for biological assay

Cell culture

HEK293T cells were obtained from American Type Culture Collection [ATCC, Manassas, VA, USA]. HEK293T cell lines were cultured in DMEM [GIBCO, Invitrogen] supplemented with heat-inactivated 10% (v/v) fetal bovine serum [GIBCO, Invitrogen] and 1% (v/v) antibiotic-antimycotic solution [GIBCO, Invitrogen]. Cells were maintained in a humidified atmosphere of 5% CO_2 incubator at 37 ℃, and cultured in 100 mm cell culture dish [CORNING].

ELISA-based assay

His-tag-LRS diluted with carbonate buffer (100 mM, pH 9.6) at the 0.5 ng/μL concentration and distribute the solution to each well of the half-bottom 96 well clear plate [CORNING, #3690]. Incubate overnight at 4 ℃ (covered) and remove the coating solution and wash three times with PBST. After blocking step (2 h, 5% BSA in PBS), each well was treated with each β-turn mimetic library member and GST-tagged RagD simultaneously for 3 hours. (GST protein was used for negative control.) Diluted GST antibody in PBS was added to each well and incubate at room temperature for 1 h. After washing with PBST, the enzyme-conjugated secondary antibody was treated and incubated at room temperature for 1 h. For developing, TMB was added to each well. Color should develop in positive wells (blue). The reaction was stopped with stopping reagent, H_3PO_4, and absorbance read later at 450 nm.

Leucine Starvation

For leucine depletion, cells were rinsed with leucine-free DMEM twice, incubated in leucine-free DMEM for 60 min and replaced with and incubated in DMEM.

Western blotting

HEK293T cells were seeded on 6-well plate and incubated in 5% CO_2 incubator at 37 ℃ overnight. HEK293T cells were starved for leucine for 1 h and treated with compounds in leucine-deprived condition. Cells were washed by PBS and harvested. Cell lysates were obtained by 30 min treatment with RIPA cell lysis buffer containing protease inhibitors and phosphatase inhibitors at ice. After the centrifugation of cell lysates at 15,000 rpm and 4 ℃ for 30 min, the protein concentration in the supernatant was measured by BCA assay. The resulting proteome were analyzed by SDS-PAGE and transferred into PVDF membrane, followed by 2% BSA blocking in TBST over 1 h. The samples were subjected to western blotting to detect the S6K1, phospho-S6K1 (T389) or GAPDH with specific primary antibodies, e.g. anti-S6K1 and anti-phopho-S6K1 (T389) [Abcam], GAPDH [Cell Signaling Technology] antibodies for overnight at 4 ℃, followed by washing with TBST for 1 h. The resulting

membrane was exposed into HRP-conjugated secondary antibody for 1 h at room temperature. After washing with TBST, the membrane was developed by ECL prime solution [GE healthcare] and the chemiluminescent signal was measured by ChemiDoc^{TM} MP imaging system.

FRET imaging experiment and analysis

We carried out FRET imaging with DeltaVision Elite imaging system [GE healthcare]. For maintaining live cell condition during experiment, imaging was performed in a CO_2 supporting chamber along with an objective air heater. HEK293T cells were transfected with LRS-CFP (Condition 1), RagD-YFP (Condition 2) and both LRS-CFP and RagD-YFP (Condition 3). Condition 1 & 2 were for calculating crosstalk and condition 3 was for FRET analysis. Images captured with CFP/CFP, YFP/YFP and CFP/YFP filter sets (excitation/emission) and 60× scale. Excitation filter: 438/24 nm and Emission filter 475/24 nm for CFP; Excitation filter: 513/17 nm and Emission filter: 548/22 nm for YFP. In each experiment, images of randomly selected 4 or 5 different cells per individual condition were taken at 10-min intervals over 3.5 h. Ultimate focusing module was operated before imaging during whole imaging process. Compounds were treated in 30 min after live cell imaging. Out of focus light is digitally removed using the SoftWorks deconvolution software. FRET analysis controlled by DeltaVision SoftWorks using the SoftWorks tool "FRET analysis" for excluding false-positive signal such as CFP crosstalk and YFP crosstalk. Using 'FRET analysis' tool, extract crosstalk information from condition 2 & 3. (CFP crosstalk was extracted from condition 2 on whole time point images and YFP crosstalk was extracted from condition 3 on whole time point images.) Net FRET images were developed by applying each crosstalk extracted from each time point. Calculate FRET efficiency within region of interest of developed Net FRET images. Finally, FRET efficiency was converted to FRET efficiency ratio by level at 30 min as a standard. The quantified data are the mean measurements from 3 independent experiments.
